# HER3 targeting augments the efficacy of panobinostat in claudin-low triple-negative breast cancer cells

**DOI:** 10.1038/s41698-023-00422-8

**Published:** 2023-08-03

**Authors:** Hui Lyu, Defu Hou, Hao Liu, Sanbao Ruan, Congcong Tan, Jiande Wu, Chindo Hicks, Bolin Liu

**Affiliations:** 1 Departments of Interdisciplinary Oncology, Louisiana State University (LSU) Health Sciences Center, New Orleans, LA USA; 2 Departments of Genetics, Stanley S. Scott Cancer Center, School of Medicine, Louisiana State University (LSU) Health Sciences Center, New Orleans, LA USA; 3 Department of Biochemistry and Molecular Biology, School of Medicine, Hunan Normal University, Changsha, Hunan China

**Keywords:** Breast cancer, Targeted therapies

## Abstract

Patients with triple-negative breast cancer (TNBC) have a poor prognosis and high relapse rate due to limited therapeutic options. This study was conducted to determine the mechanisms of action of panobinostat, a pan-inhibitor of histone deacetylase (HDAC) and FDA-approved medication for multiple myeloma, in TNBC and to provide a rationale for effective drug combinations against this aggressive disease. RNA sequencing analyses of the claudin-low (CL) TNBC (MDA-MB-231) cells untreated or treated with panobinostat were performed to identify the differentially expressed genes. Adaptive alterations in gene expression were analyzed and validated in additional CL TNBC cells. Tumor xenograft models were used to test the in vivo antitumor activity of panobinostat alone or its combinations with gefitinib, an EGFR-tyrosine kinase inhibitor (TKI). Panobinostat potently inhibited proliferation and induced apoptosis in all TNBC cells tested. However, in CL TNBC cells, this HDAC inhibitor markedly enhanced expression of HER3, which interacted with EGFR to activate both receptors and Akt signaling pathways. Combinations of panobinostat and gefitinib synergistically suppressed CL TNBC cell proliferation and promoted apoptosis in vitro and in vivo. Upregulation of HER3 compromises the efficacy of panobinostat in CL TNBC. Inactivation of HER3 combined with panobinostat represents a practical approach to combat CL TNBC.

## Introduction

Triple-negative breast cancer (TNBC) is defined by lack of ER (estrogen receptor) and PR (progesterone receptor) expression and HER2 (human epidermal growth factor receptor 2) overexpression (or *HER2* gene amplification). TNBC counts for approximately 15% of all breast cancer (BC)^1^. Compared to other BC subtypes, TNBC exhibits higher invasiveness, relapse rates, and worse survival outcome^2^. The lack of valuable biomarkers for targeted therapy represents a significant clinical challenge in the treatment of TNBC patients. Moreover, TNBC’s high heterogeneity is another pivotal factor that impedes the therapeutic efficacy in TNBC. Lehmann et al. classify TNBC into four transcriptome-based subtypes^3^, whereas other reports have defined three or four distinct tumor immune microenvironment (TIME) stratification in TNBC^4^. Apart from these, the claudin-low (CL) TNBC subtype is characterized by low expression of claudin-3, -4, and -7^5,6^. There is an urgent desire to design accurate, personalized therapeutic strategies for patients with a different subtype of TNBC.

Studies in the past decade have highlighted the vital role of epigenetic processes in cancer progression and treatment^7^. Histone deacetylase (HDAC) is dysregulated in human cancers, making them a valuable therapeutic target^8^. HDAC inhibitors (HDACi) exert their anticancer activity mainly by inducing G1 cell cycle arrest, apoptosis, and differentiation^9,10^. It is the first success of epigenetic-based cancer therapy. For example, Food and Drug Administration (FDA) has approved vorinostat (SAHA) as a regimen for cutaneous T-cell lymphoma^11^. Romidepsin and panobinostat have been approved for treating peripheral T-cell lymphoma and multiple myeloma, respectively^12,13^. Unfortunately, HDACis have not yielded promising results as monotherapy in clinical trials for most solid tumors, including BC. A number of studies investigate the combinatorial effects of an HDACi and other anticancer agents on solid tumors^14^. We previously found that entinostat (SNDX-275), a selective class I HDACi, enhanced the efficacy of trastuzumab in HER2-positive BC cells^15^. It has been shown that HDACi, including SAHA, panobinostat, and entinostat, in combination with cytotoxic agents or ionizing radiation obtained encouraging results in TNBC cells^16,17^. However, due to TNBC’s heterogeneity, further understanding of the disease’s biology and adaptive response to HDACi is required for designing effective combinatorial therapy for TNBC. Panobinostat has shown more significant action against TNBC than other BC subtypes^18,19^. In the current study, we analyzed the adaptive response of TNBC cells to panobinostat treatment. Interestingly, we noticed a rapid and dramatic upregulation of HER3 in CL TNBC cells. The increased HER3 became active via interaction with EGFR to provide survival advantage for the particular subtype of TNBC cells, thereby limiting the efficacy of panobinostat. We then performed both in vitro and in vivo studies to determine whether targeting of HER3 or EGFR would significantly enhance the antitumor activity of panobinostat against CL TNBC.

## Results

### Elevated expression of *HDAC1/2/3* was observed in BC and significantly associated with poor prognosis in BC patients

**Fig. 1:**
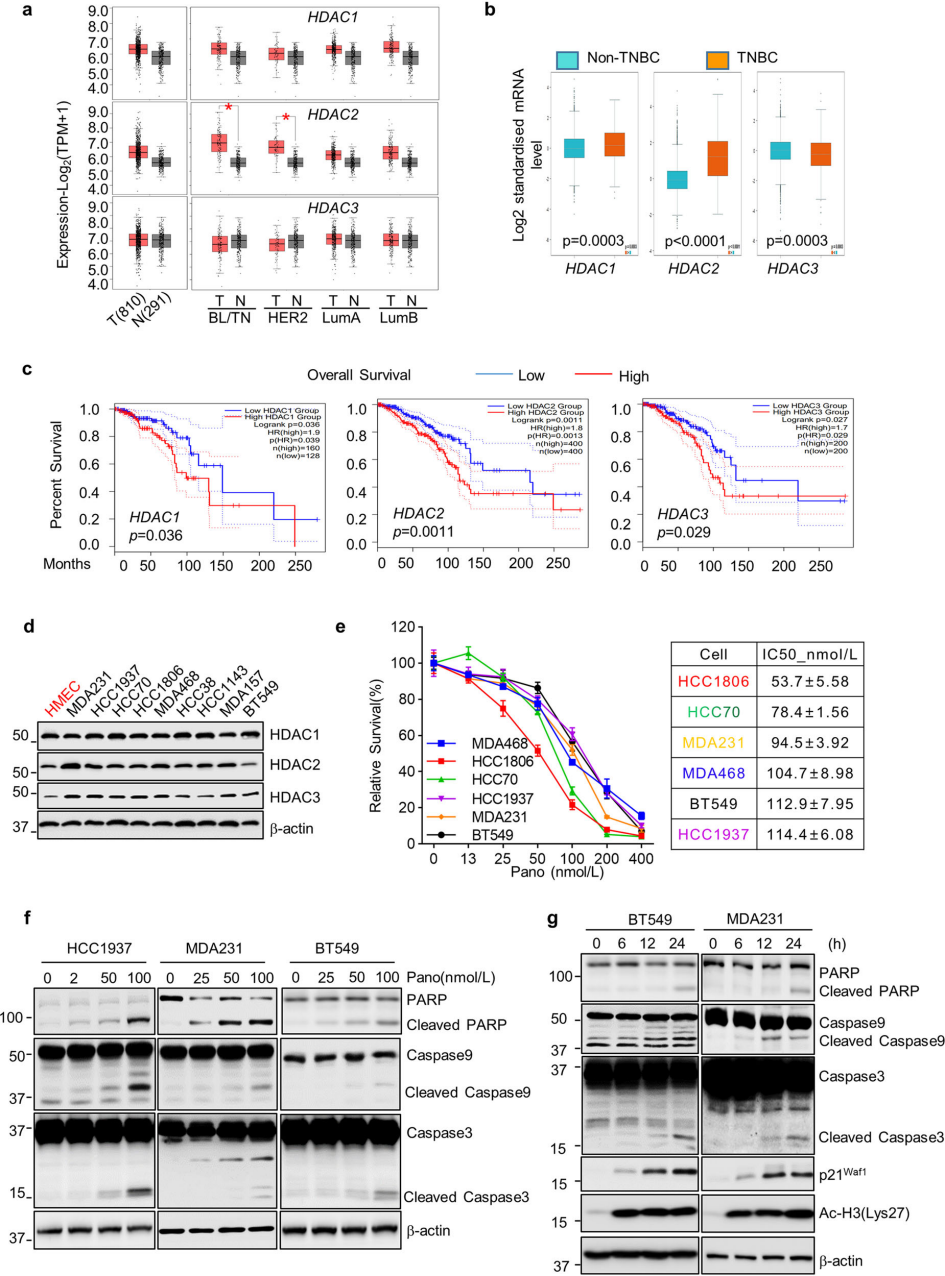
HDAC1, HDAC2, and HDAC3 are frequently overexpressed in TNBC tumors and cell lines and serve as good therapeutic targets. **a** Box plot presentation of *HDAC1, HDAC2, and HDAC3* mRNA expression in BC tumors (red box, *n* = 810) versus normal breast tissues (gray box, *n* = 291) acquired from Gene Expression Profiling Interactive Analysis (GEPIA2) website. The expressions of *HDAC1, HDAC2, and HDAC3* in different BC subtypes (BL/TN basal-like/triple negative, HER2-positive; LumA luminal A, LumB luminal B) was also compared with normal breast tissues. **b** mRNA expression of *HDAC1, HDAC2, and HDAC3* in non-TNBC tumors (blue box, *n* = 4119) versus TNBC tumors (orange box, *n* = 317) acquired from Breast Cancer Gene-Expression Miner v4.7 website. **c** Kaplan–Meier survival curves of overall survival (OS) of BC patients from TCGA datasets. The log-rank tests were used to compare OS of the patients with BC tumors expressing high (red line) versus low (blue line) mRNA levels of *HDAC1, HDAC2*, or *HDAC3*. **d** Western blot analyses of HDAC1, HDAC2, and HDAC3 in human mammary epithelial cells (HMEC) and a series of TNBC cell lines. β-actin was used as the loading control. **e** Examination of cell proliferation/survival in response to panobinostat. TNBC cell lines (4000–8000/well) were seeded onto 96-well plates for culture overnight. Cells were treated with panobinostat at indicated concentrations for 72 h. The percentages of surviving cells from each cell line relative to controls, defined as 100% survival, were determined by MTS assays. Data show a representative of three independent experiments. Bars, SD. **f**, **g** TNBC cells were seeded in 60 mm dishes. The following day, cells were treated with indicated concentrations of panobinostat (nmol/L) for 48 h (**f**) or with 50 nmol/L of panobinostat for indicated hours (**g**). Both adherent and non-adherent cells were collected. An equal amount of total cell lysates was used for western blot assays with specific antibodies directed against PARP, Caspase-9, Caspase-3, p21^waf1^, acetylated histone H3 (Ac-H3), or β-actin. Inducing Ac-H3 and cell cycle inhibitor (p21^waf1^) indicated successful HDAC inhibition.

HDACs have been shown to play an important role in the development of various human cancers, including BC^8^. However, the expression pattern of HDAC family members in BC has not been fully described. We took advantage of the online tool GEPIA2 to examine the mRNA expression of *HDAC1/2/3* in BC and normal breast tissues. We analyzed normal tissues (*n* = 291), Basal-like/TNBC (*n* = 135), HER2-positive (*n* = 66), Luminal A (*n* = 415) and luminal B (*n* = 192) BC from Match TCGA normal and GTEx datasets (http://gepia2.cancer-pku.cn/#index), and observed an elevated expression of *HDAC1/2/3* in all BC as compared to normal breast tissues. Especially, the expression of *HDAC2*, but not *HDAC1/3*, in basal-like (BL/TNBC) and HER2 subtypes was significantly higher than that in normal tissues (*p* < 0.01, Student’s *t* test) (Fig. 1a). When compared TNBC with non-TNBC groups, the expression of *HDAC1/2* in TNBC was significantly higher than that in non-TNBC, whereas *HDAC3* was lower in TNBC BC (Fig. 1b). Moreover, the Kaplan–Meier plotter revealed that high expression of *HDAC1/2/3* mRNA was significantly associated with poor overall survival in all BC patients (Fig. 1c). Higher expression of *HDAC2*, but not *HDAC1/3* also correlated with a worse disease-free survival (Supplementary Fig. 1a and data not shown). Finally, we examined expression of HDAC1/2/3 protein in a series of human TNBC cell lines. Compared with normal human mammary epithelial cells (HMEC), majority of TNBC cell lines seemed to express more HDAC2. The expression of HDAC1 was slightly higher in TNBC cells, whereas HDAC3 appeared no significant difference between TNBC and HMEC cells (Fig. 1d). The mRNA expression of *HDAC1/2/3* is significantly higher in the majority of TNBC cells as compared to HMEC (Supplementary Fig. 1b). Collectively, these data indicate that HDAC1/2/3 could potentially be developed as therapeutic targets for breast cancer patients, particularly those with triple-negative breast cancer (TNBC).

### TNBC cells were sensitive to panobinostat-induced apoptosis

To test the effect of HDACi on TNBC cells, we selected an FDA-approved pan-HDACi panobinostat to perform the subsequent studies since this HDACi was shown to exhibit potent inhibitory effects on TNBC cells^10,18^. Our studies also validated that panobinostat had superior activity as compared to SAHA or entinostat to inhibit TNBC cells (Supplementary Fig. 1c). We first probed the efficacy of panobinostat in 6 TNBC cell lines (HCC1806, MDA-MB-468 (MDA468), HCC70, HCC1937, MDA-MB-231 (MDA231), and BT549). As expected, all the TNBC cells were sensitive to panobinostat treatment with half-maximal inhibitory concentrations (IC50) between 50 and 115 nmol/L (Fig. 1e). Next, we examined apoptosis induced by panobinostat in TNBC cells. HCC1937, MDA231, or BT549 cells were treated with indicated concentrations of panobinostat for 48 h (Fig. 1f) or fixed concentration of panobinostat for 6, 12, or 24 h (Fig. 1g). Western blot assays revealed an enhanced PARP cleavage and increased cleaved forms of caspase-9 and caspase-3. Apoptosis-specific ELISA assays further verified apoptosis provoked by panobinostat treatment (Supplementary Fig. 1d). Collectively, our data demonstrated that panobinostat exhibited profound anti-survival effects on TNBC cells via induction of growth inhibition and apoptosis.

### Upregulation of HER3 significantly compromised the anti-proliferative/anti-survival effects of panobinostat on CL TNBC cells

**Fig. 2:**
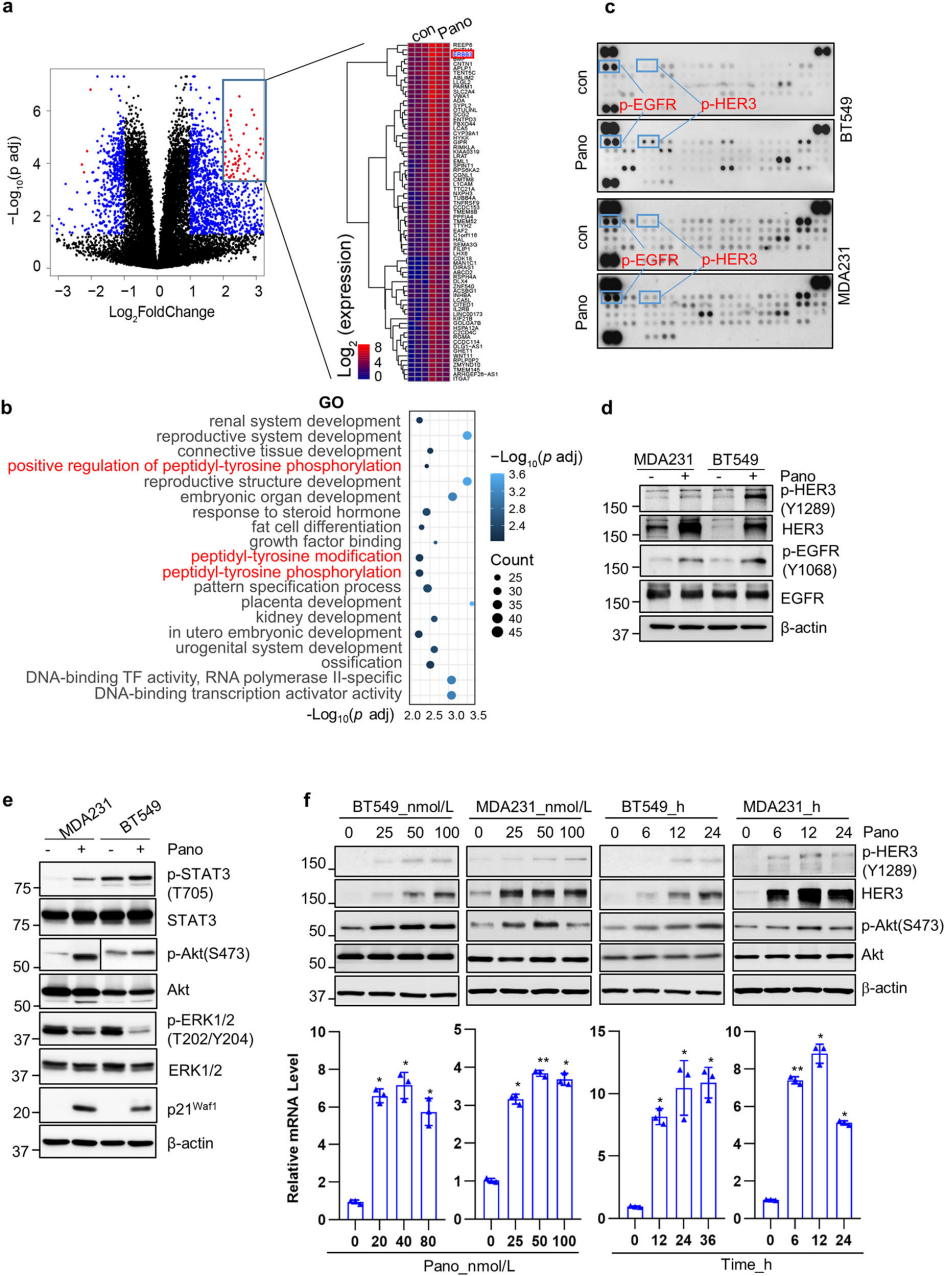
Panobinostat upregulates HER3 at both mRNA and protein levels and induces activation of HER3 and its downstream signaling pathways in CL TNBC cells. **a** Volcano plot presentation of RNA-Seq analyses of the differentially expressed genes in MDA-MB-231 cells treated with vehicle control or panobinostat. Each point represents the average value of one transcript in three replicates. The expression difference is considered significant for a |Log 2FC| > 1 & *p* < 0.05 (blue or red dots). The heat map shows the genes with significantly increased expression in panobinostat (Pano)-treated cells versus the control (con) cells (*p* < 0.0005, Log2FC > 2). **b** Gene Ontology (GO) enrichment analysis for genes upregulated in response to panobinostat. In all, 19 GO terms were represented by gene count >20, with *p* < 0.01. **c**–**e** BT549 or MDA-MB-231 (MDA231) cells were treated with vehicle control (DMSO) or panobinostat (Pano, 25 nmol/L) for 8 h. An equal amount of total cell lysates was subjected to Phospho-RTK arrays. Increased levels of p-HER3 and p-EGFR were observed in panobinostat (Pano)-treated cells as compared to the control (con) cells (**c**). The same batch of the cell lysates was used for western blot analyses of p-HER3, HER3, p-EGFR, EGFR, or β-actin (**d**), or for western blot analyses of p-STAT3, STAT3, p-Akt, Akt, p-ERK1/2, ERK1/2, p21^waf1^, or β-actin (**e**). **f** BT549 or MDA-MB-231 cells were seeded in 60 mm dishes for culture overnight. Cells were then treated with indicated concentrations of panobinostat (Pano) for 24 hours or with 25 nmol/L of panobinostat (Pano) for indicated times (hours). The cells were then collected for western blot analyses of p-HER3, HER3, p-Akt, Akt, or β-actin (top). Total RNAs isolated from the cells were subjected to RT-qPCR measurement of the expression levels of *erbB3* mRNA, which were normalized to *GAPDH* levels (bottom). Data shows a representative of three independent experiments. Bars, SD. *p < 0.05, **p < 0.01, ***p < 0.005 vs untreated control.

Although panobinostat showed potent inhibitory effects on TNBC cells, monotherapy is generally insufficient to combat solid cancers^20,21^. Drug-induced activation of alternative survival pathways frequently occurs, leading to therapeutic resistance. Thus, uncovering the alternative survival pathways activated upon panobinostat treatment may provide a rationale for drug combinations against TNBC. To address it, we first performed RNA sequencing (RNA-Seq) analysis of MDA-MB-231 cells untreated vs treated with panobinostat. As shown in Fig. 2a, Supplementary Table 1 included all significantly upregulated genes (adjust *p* < 0.05) and Supplementary Table 2 showed 73 genes that were significantly upregulated in panobinostat-treated cells (Log FC > 2, *p* < 0.0005). Among them, *erbB3* (or *HER3*) was markedly induced after panobinostat treatment. GO enrichment analysis revealed that 19 GO term were significantly involved in response to panobinostat stimulation (Count > 20, *p* adj < 0.01), with three of them associated with the processes of peptidyl-tyrosine phosphorylation (Fig. 2b and Supplementary Table 3). DEGs in the biological network showed erbB3 involved in 3 processes (Supplementary Fig. 2a). These results suggested that receptor tyrosine kinases (RTKs) might play a role in the adaptive response to panobinostat in the TNBC cells. Then, we utilized the Human Phospho-Receptor Tyrosine Kinase Array to examine the phosphorylation status of RTKs in BT549 and MDA-MB-231 cells untreated or treated with panobinostat. Panobinostat indeed altered the phosphorylation levels of several RTKs (Fig. 2c). Notably, the levels of phosphorylated HER3 (p-HER3) and EGFR (p-EGFR) were clearly increased in both cell lines after panobinostat treatment. Western blot assays confirmed the results of our RNA-Seq analysis and Phospho-RTK array. i.e., panobinostat treatment increased the levels of both p-HER3 and p-EGFR in BT549 and MDA-MB-231 cells. It also enhanced expression of total HER3 but had little effect on EGFR expression (Fig. 2d). We next examined the downstream signaling pathways of HER3. Pathway enrichment analysis indicated significant change of PI3K-Akt signaling pathway upon panobinostat treatment (Supplementary Fig. 2a). We found that the levels of p-STAT3 and p-Akt were increased, whereas the p-ERK1/2 levels were decreased in MDA-MB-231 and BT549 cells upon panobinostat treatment (Fig. 2e). To explore whether panobinostat-induced upregulation of HER3 was a general phenomenon in all TNBC cells, we treated several TNBC cell lines (MDA-MB-468, HCC1806, HCC70, MDA-MB-231, BT549, MDA-MB-436, and Hs578T) with panobinostat and performed both western blot and RT-PCR assays. Upregulation of HER3 was only detected in BT549, MDA-MB-231, MDA-MB-436, Hs578t and MDA-MB-157 cells, which initially have low or undetectable HER3 expression (Fig. 2f and Supplementary Fig. 3a). Interestingly, all the HER3^low/negative^ TNBC cells belong to the CL subtype^22^. We did not observe panobinostat-induced expression of HER3 in non-CL TNBC (MDA-MB-468, HCC1806 and HCC70) cells, which express relatively high levels of HER3 (Supplementary Fig. 3a). Panobinostat treatment of CL TNBC cells also increased the levels of both p-HER3 and p-Akt in a dose- and time-dependent manner (Fig. 2f top). Similar results were obtained from studies of the TNBC-PDX model-derived 4IC cells^23^ that has low HER3 expression (Supplementary Fig. 3b). Flow cytometry analysis revealed a significant right shift of the peak after panobinostat treatment, indicating an increase in HER3 expression on MDA-MB-231 cell membrane (Supplementary Fig. 3c). Taken together, our data indicated that panobinostat induced HER3 expression and activated the HER3/Akt signaling pathway in CL TNBC cells. However, whether elevated HER3 and activation of its downstream signaling contributed to the limited response of panobinostat in the CL TNBC cells remained to be elucidated.

**Fig. 3:**
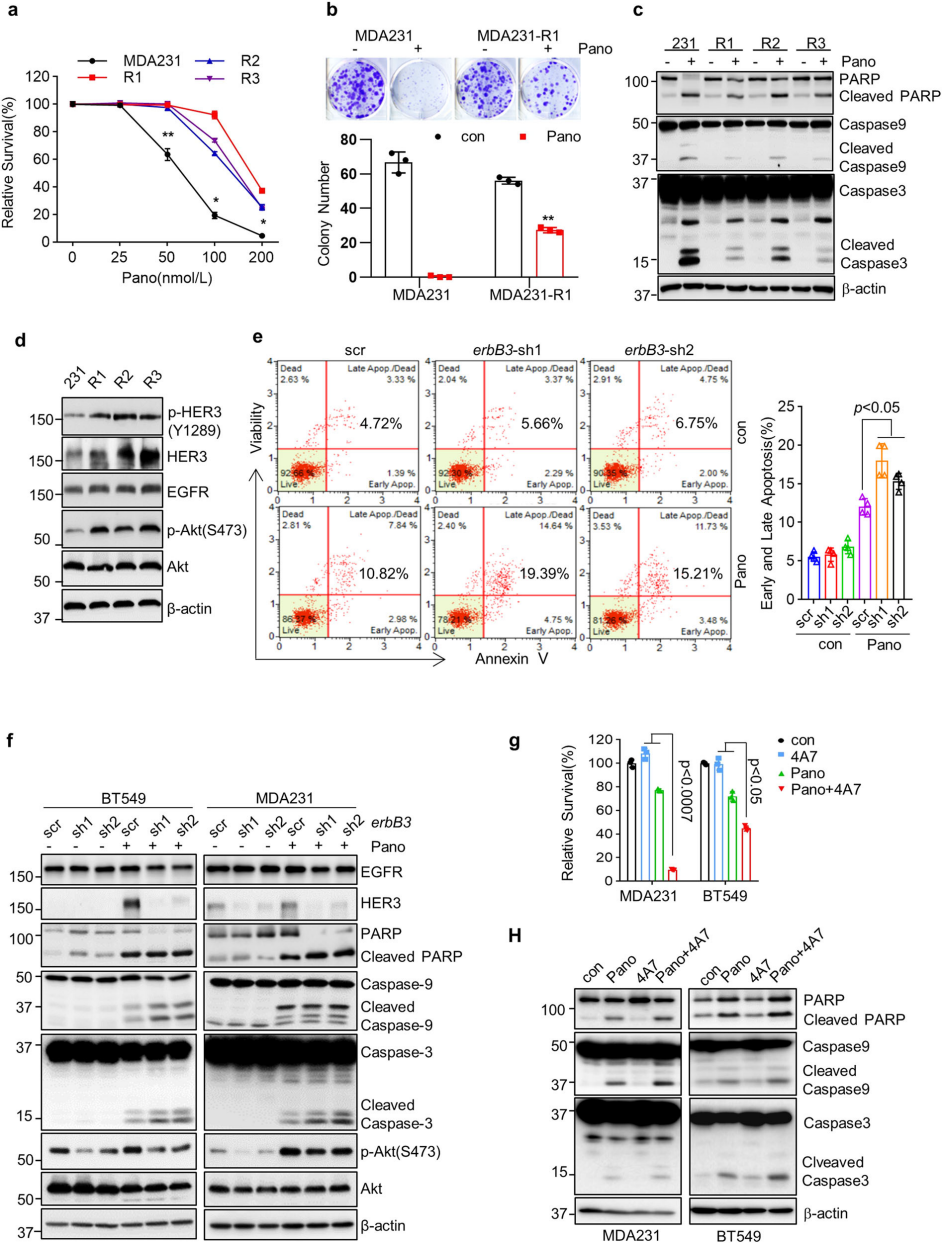
Upregulation of HER3 results in reduced anti-proliferative/anti-survival effects of panobinostat on CL TNBC cells. **a** MDA-MB-231 or its sublines (R1, R2, and R3)-derived through long-term treatment of panobinostat were seeded onto 96-well plates for culture overnight. The cells were then treated with indicated concentrations of panobinostat for 72 h. Cell viability was determined with MTS assays. Data shows a representative of three independent experiments. Bars, SD. *p < 0.05, **p < 0.01. **b** MDA231 or MDA231-R1 cells seeded onto 6-well plates (600 cells/well) were cultured with medium without panobinostat or the same medium containing 25 nmol/L of panobinostat. The culture medium was changed every three days for 11 days. Representative images of the colony formation assays for each cell line were taken by a digital camera on day 11 (upper panel), and quantification of the colony numbers was performed using the Image J Software (lower panel). Bars, SD. **p < 0.01 **c** MDA-MB-231 (231), R1, R2, or R3 cells untreated or treated with 25nmol/L of panobinostat (Pano) for 48 h were collected and examined by western blot assays of PARP, Caspase-9, Caspase-3. or β-actin. **d** MDA-MB-231 (231), R1, R2, or R3 cells under normal culture conditions were collected and examined by western blot assays of p-HER3 (Y1289), HER3, EGFR, p-Akt (S473), Akt, or β-actin. **e** MDA-MB-231-R1 cells were transiently transduced with the lentivirus containing either a scramble (scr) control shRNA or specific shRNA against *erbB3* (*erbB3*-sh1 or *erbB3*-sh2) for 24 h. The cells were then untreated (con) or treated with 50nmol/L of panobinostat (Pano) for another 24 h. Cells were collected and analyzed for early and late apoptosis with Muse™ Annexin V & Dead Cell Assays. Data shows a representative of three independent experiments. Bars, SD. Student’s *t* test, *p < 0.05. **f** BT549 or MDA-MB-231 cells were transduced with the lentivirus containing either a scramble (scr) shRNA or *erbB3* specific shRNA (*erbB3*-sh1 or *erbB3*-sh2) were treated with vehicle (DMSO) control (−) or 50 nmol/L of panobinostat (+ Pano) for another 24 h. Cells were collected and examined by western blot assays. **g** MDA-MB-231 or BT549 cells were seeded onto 96-well plates for growth overnight. Cells were then treated with DMSO (con), 50 nmol/L of panobinostat (Pano), 4A7 (20 μg/ml), or the combinations of panobinostat and 4A7 (Pano+4A7) for 72 h. Cell viability was determined with MTS assays. Data shows a representative of three independent experiments. Bars, SD. **h** MDA-MB-231 or BT549 cells were treated with DMSO (con), 50 nmol/L of panobinostat (Pano), 4A7 (20 μg/ml), or combinations of panobinostat and 4A7 (Pano+4A7) for 48 h. Cells were collected and examined by western blot assays.

To clarify the role of the upregulated HER3 in CL TNBC cells’ response to panobinostat, we cultured MDA-MB-231 cells with gradually increased concentrations of panobinostat for several months and selected three panobinostat-resistant sublines (MDA-MB-231-R1, -R2, and -R3). As compared to the parental MDA-MB-231 cells, R1, R2, and R3 cells were significantly less sensitive to the growth inhibition induced by panobinostat (Fig. 3a). While panobinostat dramatically suppressed colony formation in MDA-MB-231 cells, it was much less effective to decrease the colony numbers in MDA-MB-231-R1 cells (Fig. 3b). Western blot analyses found that panobinostat potently induced PARP cleavage and both caspase-9 and caspase-3 activation in MDA-MB-231 cells, but showed reduced capability to do so in R1, R2, and R3 cells (Fig. 3c). These data indicated that MDA-MB-231-R1, -R2, and -R3 cells were relatively insensitive to panobinostat-induced growth inhibition and apoptosis. Notably, HER3 protein and mRNA were markedly increased in MDA-MB-231-R1, -R2, and -R3 cells (Fig. 3d and Supplementary Fig. 4a). In contrast, the other RTKs, such as EGFR, MET, and IGF-1R, remained unchanged (Supplementary Fig. 4b), suggesting a specific upregulation of HER3 by panobinostat. Next, we sought to determine if the increased HER3 was causal in developing resistance to panobinostat. We infected MDA-MB-231-R1 cells with the lentivirus containing either a scramble (scr) control shRNA or specific shRNA against *erbB3* (*erbB3*-sh1 or *erbB3*-sh2) and followed panobinostat treatment. Flow Cytometry analyses revealed that specific knockdown of *erbB3* expression significantly enhanced panobinostat-induced apoptosis in MDA-MB-231-R1 cells (Fig. 3e). Further studies showed that *erbB3* depletion clearly increased panobinostat-induced PARP cleavage and caspase-9 and caspase-3 activation in both MDA-MB-231 and MDA-MB-231-R1 cells (Supplementary Fig. 4c). Collectively, our data demonstrated that upregulation of HER3 was an adaptive response to panobinostat treatment, which in turn impaired panobinostat’s efficacy in CL TNBC cells.

### Inactivation of HER3 or its downstream Akt kinase augmented panobinostat-induced growth inhibition and apoptosis in CL TNBC cells

Increased levels of both p-HER3 and p-Akt were observed not only in the CL TNBC cells treated with panobinostat (Fig. 2f top), but also in the resistant sublines (MDA-MB-231-R1, -R2, and -R3) (Fig. 3d). These data suggested that panobinostat-induced HER3 expression led to activation of HER3 and its downstream Akt kinase, which inspired us to hypothesize that inactivation of HER3 or Akt would overcome panobinostat resistance and synergistically enhance panobinostat-induced growth inhibition and apoptosis in CL TNBC cells. To this end, we first used specific shRNA (*erbB3*-sh1 or *erbB3*-sh2) to silence *erbB3* expression. BT549 and MDA-MB-231 cells transiently transduced with the lentivirus containing a scramble (scr) control shRNA or an *erbB3*-specific shRNA (*erbB3*-sh1 or *erbB3*-sh2) were untreated or treated with panobinostat. Our data showed that silencing of *erbB3* clearly enhanced panobinostat-induced cleavages of PARP, caspase-9, and caspase-3 in both BT549 and MDA-MB-231 cells (Fig. 3f). Similar results were observed in the studies of the primary 4IC cells (Supplementary Fig. 5). Furthermore, Specific knockdown of HER3 also reduced the levels of p-Akt-induced by panobinostat in both cell lines (Fig. 3f). Then, we utilized our recently developed anti-HER3 monoclonal antibody (4A7, submitted separately) to inhibit HER3. Combinations of 4A7 and panobinostat significantly induced growth inhibition in both MDA-MB-231 and BT549 cells (Fig. 3g). 4A7 in combination with panobinostat also potently induced PARP cleavage and both caspase-9 and caspase-3 activation (Fig. 3h). Moreover, we used a specific inhibitor of Akt (Akti-1/2) to examine its combinatorial effects with panobinostat on CL TNBC cells. The Akti-1/2 (Akti) in combination with panobinostat as compared to single agent, significantly inhibited proliferation of both MDA-MB-231 and BT549 cells (Supplementary Fig. 6a). The combinations were much more effective than either agent alone in inducing apoptosis, evidenced by markedly increased Annexin V-staining cells (Supplementary Fig. 6b), potently induced caspase-9 and caspase-3 activation (Supplementary Fig. 6c) and profound cell death detected by the Live/Dead Cell Imaging (Supplementary Fig. 6d). Thus, inhibition of Akt dramatically intensified panobinostat-mediated anti-proliferative/anti-survival effects on CL TNBC cells.

### Panobinostat in combination with gefitinib, an EGFR-tyrosine kinase inhibitor (TKI) synergistically induced growth inhibition and apoptosis in CL TNBC cells

**Fig. 4:**
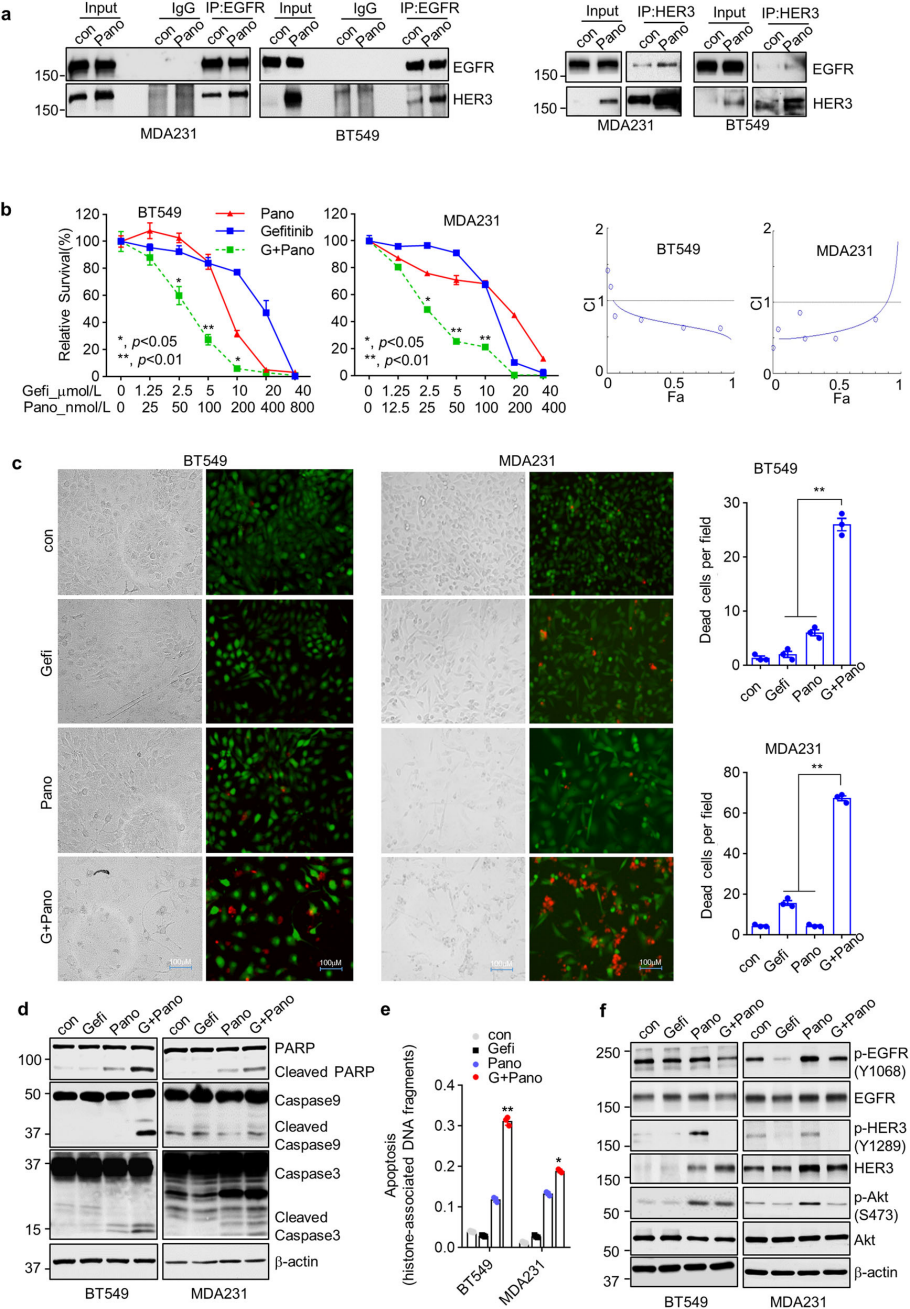
HER3 upregulation increases its interactions with EGFR, conferring synergistic effects between panobinostat and gefitinib to induce growth inhibition and apoptotic cell death in CL TNBC cells. **a** Co-immunoprecipitation of HER3 with EGFR. Left and right, immunoprecipitation (IP) results showed that HER3 interacted with EGFR. MDA-MB-231 or BT549 cells were treated with DMSO (con) or 50nmol/L Panobinostat (Pano) for 8 h. And 800 mg cell lysates were immunoprecipitated with anti-rabbit IgG, anti-EGFR (left), or anti- HER3 antibody (right), then analyzed by western blots with an antibody specific for the precipitated and co-precipitated protein (EGFR and HER3). **b** Combinations of gefitinib and panobinostat synergistically induced growth inhibition of CL TNBC cells. BT549 and MDA-MB-231 cells were seeded in 96-well plates (5000 cells/well) with fresh DMEM/F12 medium. The next day cells were treated with indicated concentrations of Pano or gefitinib (Gefi) or their combinations for 72 h. The percentages of surviving cells from each cell line relative to controls, defined as 100% survival, were determined by MTS assays. Data shows the representative of three independent experiments.(left). The combination index (CI) curves were calculated using Calcusyn software (right). A lower CI, when it is less than 1, indicates stronger evidence in favor of synergy. **c** BT549 or MDA-MB-231 cells were seeded in 6-well plate, treated with DMSO (con), 2 μmol/L of gefitinib (Gefi), 25 nmol/L of panobinostat (Pano), or their combinations (G+Pano), respectively. After 48 h, cells were stained with Live/Dead Cell Imaging Kit. Representative images were presented. The green color indicated live cells and red color showed dead cells (left). Dead cells were counted at three random fields (right). **d**–**f** Combinations of Pano and Gefi significantly enhanced apoptosis and suppressed activation of p-HER3/p-Akt signal in CL TNBC cells. Same treatment as in Live/Dead Cell Imaging assay, cell lysates were subjected to western blot assays with specific antibodies directed against PARP, Caspase-9, Caspase-3, or β-actin (**d**), and apoptosis analysis using a cell death detection ELISA (**e**). BT549 and MDA-MB-231 cells were seeded in 6-well plate, treated with DMSO (con), 2 μmol/L of gefitinib(Gefi), 25 nmol/L of panobinostat (Pano), or their combinations (G+Pano). After 24 h, cells were collected and examined by western blot assays of p-HER3(Y1289), HER3, p-EGFR(Y1068), EGFR, p-Akt(S473), Akt, or β-actin (**f**). All data are presented as means ± S.D. (*n* = 3). *p < 0.05; **p < 0.01; ***p < 0.001.

Because of its lack of or impaired kinase activity, HER3 has to form hetero-dimerization with another receptor to activate signaling cascades promoting cell growth and survival^24^. EGFR and HER2 are the most preferred dimerization partners for HER3 to activate signaling pathways^25^. TNBC lacks overexpression of HER2, but frequently overexpresses EGFR^26^. We wondered whether panobinostat-induced upregulation of HER3 might form dimerization with EGFR in CL TNBC cells. Co-immunoprecipitation (co-IP) assays were performed to examine the interactions of HER3 and EGFR. MDA-MB-231 and BT549 cells untreated or treated with panobinostat were subjected to IP analyses with either a control IgG or a specific Ab against EGFR (Fig. 4a left) or HER3 (Fig. 4a right) and followed by western blot assays of EGFR and HER3. While HER3 and EGFR slightly interacted with each other in untreated cells, the HER3/EGFR interactions markedly increased upon panobinostat treatment in both MDA-MB-231 and BT549 cells. Thus, the increased expression of HER3-induced by panobinostat formed heterodimers with EGFR in CL TNBC cells, providing an alternative option to suppress HER3 signaling via targeting its dimerization partner - EGFR. We then examined the combinatorial effects of gefitinib (an EGFR-TKI) and panobinostat on CL TNBC cells. Gefitinib in combination with panobinostat was significantly more effective than either agent alone in inhibiting proliferation of BT549 and MDA-MB-231 cells. The combinations were synergistic as the combination index (CI) was less than 1 for both cell lines (Fig. 4b). Similar results were also obtained from our studies of the primary 4IC cells (Supplementary Fig. 7a). Moreover, Live/Dead Cell Imaging assays showed much more dead cells upon combinatorial treatment of gefitinib and panobinostat (Fig. 4c). Western blot assays revealed profound apoptosis, evidenced by increased PARP cleavage and active forms of caspase-9 and caspase-3 in the combinatorial treatment (Fig. 4d and Supplementary Fig. 7b). Consistently, apoptosis-specific ELISA confirmed that gefitinib significantly enhanced panobinostat-induced apoptosis in the CL TNBC cells (Fig. 4e). Further studies showed that gefitinib successfully reduced the levels of p-HER3 and p-Akt-induced by panobinostat (Fig. 4f). Collectively, our data indicated that combinations of gefitinib and panobinostat synergistically induced growth inhibition and apoptotic cell death in CL TNBC cells.

### Combinations of panobinostat and gefitinib exerted potent antitumor activity against TNBC in vivo

**Fig. 5:**
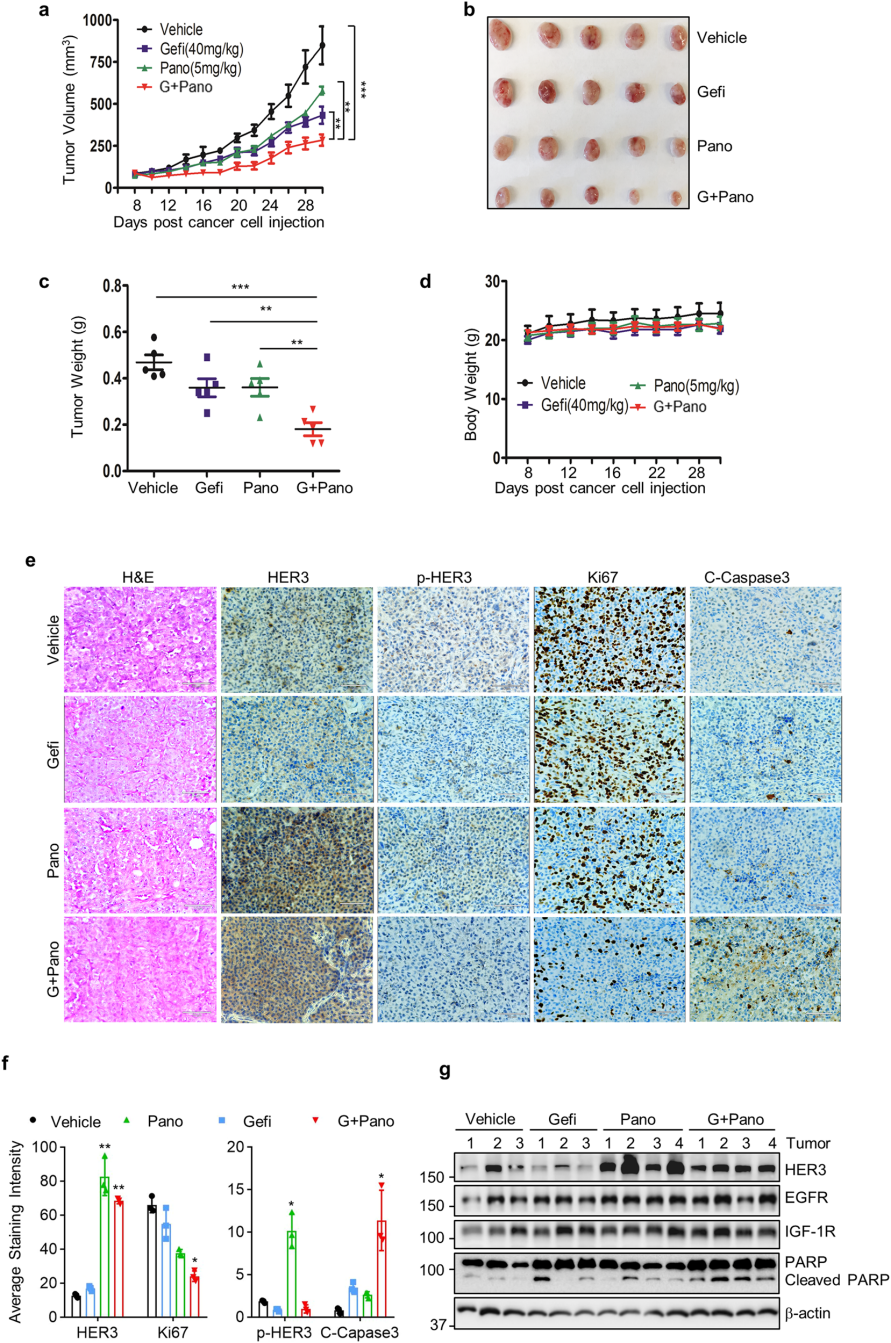
Combinations of panobinostat and gefitinib exhibit potent in vivo antitumor activity in CL TNBC xenograft models. **a** Tumor growth curves were plotted using average tumor volume within each group at the indicated time points. Bars: SD. **b**, **c** At the end of the experiment, tumor-bearing mice from the vehicle, gefitinib (Gefi), panobinostat (Pano), or combination-treated (G+Pano) group were sacrificed. The tumors were dissected, imaged as indicated (**b**) and measured for weight (**c**). **d** The body weight of mice was measured throughout the study. **e**, **f** Formalin-fixed paraffin-embedded sections of xenograft tumors were analyzed with H&E staining. Representative images presented IHC staining for HER3, p-HER3, Ki67, and Cleaved Caspase-3. Scale bar, 70 μm (**e**). Quantification of IHC staining with ImageJ and ImageJ plugin IHC profile (**f**). **g** Cell lysis from the tumors was prepared. Western blot assays were performed to examine the expression of HER3, EGFR, IGF-1R, and apoptotic markers PARP. β-actin was used as a loading control. Two-way ANOVA was used for statistical analysis. All data are presented as means ± S.D. (*n* = 5). *p < 0.05; **p < 0.01; ***p < 0.001.

To determine the antitumor activity of panobinostat and gefitinib combination in vivo, we took advantage of a tumor xenograft model established from MDA-MB-231 cells. When the tumors volume reached around 80 mm^3^, the tumor-bearing mice were treated daily with either vehicle (40% β-cyclodextrin in ddH2O), panobinostat (5 mg/kg), gefitinib (40 mg/kg), or Pano+Gefi via i.p. injection for 21 days. Tumor growth was monitored every two days. We discovered that tumor growth in the mice with combinatorial treatment was significantly inhibited as compared to that in the mice with single agent treatment (Fig. 5a). The tumor growth inhibition was also evidenced by a marked reduction of tumor size and weight (Fig. 5b, c). There was no difference in the mouse body weight among the treatment groups (Fig. 5d). IHC analyses showed that panobinostat induced upregulation of HER3 in vivo. The combinatorial treatment significantly reduced expression of Ki67, increased the tumor cells with positive staining for cleaved caspase-3, and was accompanied by the decrease of p-HER3 (Fig. 5e, f). Western blot analysis of the tumor samples further confirmed that panobinostat treatment increased the expression of HER3, but not EGFR or IGF-1R (Fig. 5g). Collectively, our data indicated that panobinostat and gefitinib combination exerted potent antitumor activity against CL TNBC in vivo, likely via gefitinib suppressing HER3 feedback activation induced by panobinostat.

### Panobinostat seemed to enhance HER3 expression in CL TNBC cells via a mechanism involving in c-Myc inhibition

**Fig. 6:**
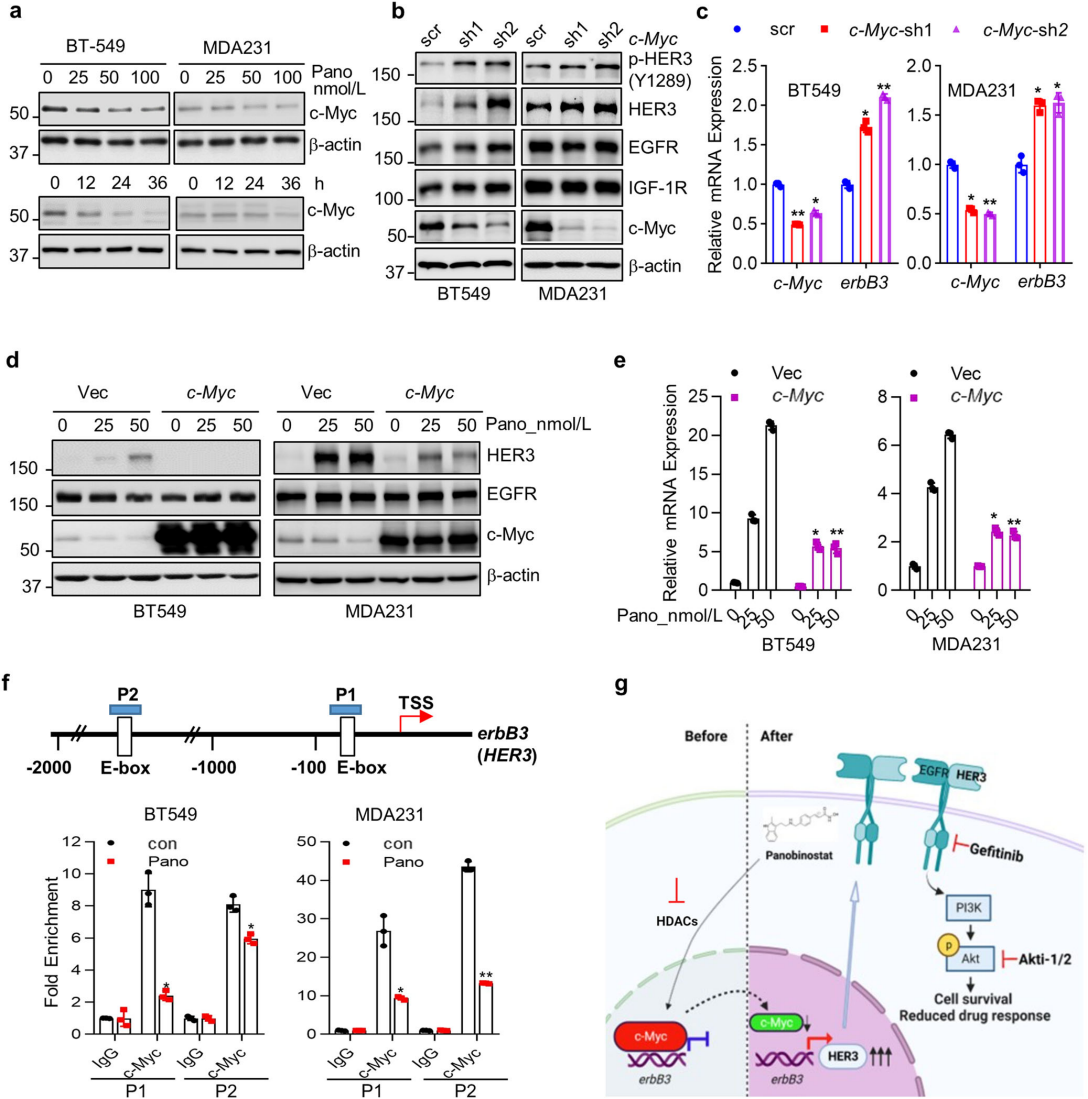
Panobinostat induces upregulation of HER3 via suppression of c-Myc expression in CL TNBC cells. **a** BT549 or MDA-MB-231 cells were seeded in 60 mm dishes for culture overnight. The next day, cells were treated with indicated concentrations of panobinostat (nmol/L) for 24 h or 25 nmol/L of panobinostat for indicated times (h). Then the cells were collected for western blot analyses of c-Myc or β-actin. **b**, **c** BT549 or MDA-MB-231 were transduced with the lentivirus containing either a scramble (scr) control shRNA or specific shRNA against *c-Myc* (*c-Myc*-sh1 or *c-Myc*-sh2) for 48 hours. The cells were then collected for western blot analyses of p-HER3 (Y1289), HER3, EGFR, IGF-1R, c-Myc or β-actin (**b**). Total RNAs isolated from the cells were subjected to RT-qPCR measurement of the expression levels of *c-Myc* and *erbB3* mRNA, normalized to *GAPDH* levels (**c**). **d**, **e** BT549 or MDA-MB-231 cells were transiently transduced with the lentivirus containing either a c-Myc construct (*c-Myc*) or vehicle vector (Vec) for 24 h, then treated with the indicated concentration of Pano for another 24 h and analyzed for western blot analyses with specific antibodies directed against HER3, EGFR, c-Myc, or β-actin (**d**). The same transduction cells treated with the indicated concentration of Pano for 8 h were collected for RT-qPCR measurement of the expression levels of *c-Myc* and *erbB3* mRNA, normalized to *GAPDH* levels (**e**). **f** Schematic diagrams of *erbB3* (*HER3*) and PCR amplicons indicated as P1/P2 used for ChIP-qPCR (upper). BT549 and MDA-MB-231 cells were treated with DMSO (con) or panobinostat (Pano) for 24 h before cross-linking. The chromatin of each sample was immunoprecipitated using IgG or anti-c-Myc antibody. ChIP-qPCR showed a significant reduction of c-Myc binding on the promoter regions of *HER3* upon panobinostat stimulation (lower). **g** Proposed working model. Panobinostat treatment decreased c-Myc protein, releasing its repression of *HER3* gene transcription in CL TNBC cells. Upregulated HER3, interacted with other RTKs (such as EGFR), activated PI3K-Akt signaling, and promoted cell survival, causing the reduced response to panobinostat. Blockade of this compensatory HER3 signaling activation with inhibition of either EGFR via gefitinib or Akt kinase via Akt inhibitor (Akti-1/2) will reinforce the efficacy of panobinostat in CL TNBC. Created with BioRender.com. Data are presented as the average of three replicates ±SD *p < 0.05, **p < 0.01.

To understand the underly mechanism of HER3 upregulation post-panobinostat treatment in CL TNBC cells, we thoroughly reviewed the literature and found that c-Myc had been implicated in the adaptive reprogramming of breast cancer kinome. Losing the c-Myc function contributed to the upregulation of several RTKs in TNBC cells^27^. HDACi, including SAHA and entanostat have been shown to be involved in c-Myc downregulation^28,29^. We wondered whether c-Myc might play a role in panobinostat-induced upregulation of HER3 in CL TNBC cells. First, we found panobinostat decreased c-Myc protein expression in a dose- and time-dependent manner in both MDA-MB-231 and BT549 cells (Fig. 6a). Next, we utilized two specific shRNA sequences to silence *c-Myc* expression in MDA-MB-231 and BT549 cells. Our data showed that specific knockdown of *c-Myc* led to a marked upregulation of HER3 at both protein and mRNA levels (Fig. 6b, c), suggesting that downregulation of c-Myc mimicked the action of panobinostat to induce HER3 expression in CL TNBC cells. To further define the role of c-Myc in panobinostat-induced upregulation of HER3, we conducted rescue assays with ectopic expression of *c-Myc* via a cDNA vector transfection. Panobinostat-induced upregulation of HER3 at both protein and mRNA levels was significantly impaired upon overexpression of c-Myc in both MAD-MB-231 and BT549 cells (Fig. 6d, e). To substantiate the role of c-Myc on repressing *erbB3* gene transcription, chromatin immunoprecipitation (ChIP) assays were performed to detect the binding of c-Myc to *erbB3* promoter. As shown in Fig. 6f, interactions of c-Myc with the chromatin fragments corresponding to the two putative c-Myc binding sites (P1& P2) on *erbB3* promoter were dramatically decreased in panobinostat-treated MAD-MB-231 and BT549 cells. Thus, our data indicated that inhibition of c-Myc contributed at least in part to panobinostat-induced upregulation of HER3 in CL TNBC cells.

## Discussion

HDACi elicit cancer cell‐specific cytotoxicity and exert potent antitumor activity in various human cancers, including TNBC^30,31^. In this study, we observed dysregulated expression of *HDAC1/2/3* in BC and found that high mRNA expression of *HDAC1/2/3* associated with poor prognosis in BC patients. In particular, the expression of *HDAC2* was significantly higher in TNBC than that in non-TNBC. These data provided a rational support for applying HDACi to treat TNBC. Compared to other subtypes of BC, TNBC showed higher sensitivity to panobinostat^18^. In agreement, our data confirmed that panobinostat at nanomolar range exhibited profound anti-proliferative/anti-survival effects on TNBC cells. Panobinostat also shows less toxicity to normal cells and more safety than other HDACi^32^ and inhibits TNBC metastasis via repression of epithelial-to-mesenchymal transition (EMT) regulators^33^. Thus, our analyses and others strongly support the notion that panobinostat is likely an effective therapeutics in combination with other anticancer drugs for TNBC treatment.

To better understand the mechanisms of the response of TNBC to panobinostat, we performed RNA-Seq analysis to compare the differentially expressed genes prior vs. post panobinostat treatment of MDA-MB-231 cells and discovered a significant increase of *HER3* expression upon panobinostat treatment. Additional studies confirmed that panobinostat markedly upregulated HER3 expression at both protein and mRNA levels and activated the downstream Akt kinase, especially in the TNBC cells with low or undetectable HER3 expression. Interestingly, these particular TNBC cells belong to claudin-low (CL) TNBC^22^, which is a novel molecular subtype, generally characterized by the low expression of genes involved in tight junctions and cell-cell adhesion and enriched for genes associated with EMT and breast cancer initiating cells^34,35^. CL TNBC was found scattered within the large basal-like group of tumors based on their expression of the immune and/or stromal gene clusters^6^. Panobinostat-induced HER3 expression/activation appeared to create a compensatory bypass pathway on which CL TNBC cells relied to proliferate and survive. We believe that the adaptive upregulation of HER3 opens a new avenue for combining panobinostat with therapeutics targeting HER3 against CL TNBC.

HER3 is unique among the HER family members, as it has no or impaired kinase activity^36^. HER3 must interact with another receptor to activate downstream signaling pathways, thereby promoting drug resistance and tumor metastasis^25,37^. Effective inhibition of HER3 is thought to be required to overcome the resistance and enhance therapeutic efficacy^38^. However, there is currently no FDA-approved HER3-targeted therapy for cancer treatment. Targeting HER3 with a blocking Ab is the only strategy examined in preclinical and clinical studies^39^. Several anti-HER3 Abs have been reported to exhibit antitumor activity and show promising as cancer therapeutics^25^. Recent clinical trials with the patritumab deruxtecan (HER3-Dxd; an Ab-drug conjugate (ADC) consisting of an anti-HER3 Ab and topoisomerase I inhibitor payload) have produced impressive results in non-small cell lung cancer (NSCLC) patients with EGFR-TKI resistance^40,41^. It would be interesting to test if panobinostat in combination with HER3-Dxd exerts significant efficacy in CL TNBC.

In addition to directly inhibiting HER3, we also sought to block HER3 activation via targeting its critical dimerization partner. TNBC lacks overexpression of HER2, but frequently overexpress EGFR. Elevated expression of EGFR significantly correlates with poor prognosis in TNBC patients. Thus, EGFR has been considered as an attractive target for TNBC^42^. Unfortunately, EGFR-targeted therapies, including monoclonal Abs and TKIs show limited efficacy for TNBC treatment in both clinical and preclinical settings^43^. The disappointing data suggest that EGFR expression per se without considering its activation may not predict which TNBC patients benefit from EGFR-target therapy. Our current data indicated that in CL TNBC cells, panobinostat-induced upregulation of HER3 interacted with EGFR, enabling the cells acquiring ‘addiction’ to HER3-EGFR dimerization/activation. This idea is supported by a recent report showing that combined HER3-EGFR high score performs better than considering the receptors individually to predict worse clinical outcomes in TNBC patients^44^. We, therefore, hypothesized that administration of the EGFR-TKI gefitinib would significantly enhance the anti-proliferative/anti-survival effects of panobinostat on CL TNBC cells. Indeed, panobinostat in combination with gefitinib synergistically induced CL TNBC cell growth inhibition and apoptosis in vitro and exerted profound antitumor activity in vivo. Our data provide a foundation to revive the efficacy of EGFR-TKIs in TNBC.

Investigation with ChIP assays revealed that c-Myc bound to the promoter of *HER3* gene, thereby repressing its transcription. These findings are consistent with a previous report indicating that c-Myc exhibits a suppressive effect on RTKs, such as PDGFRβ and VEGFR2^27^. However, it is currently unclear if c-Myc-mediated repression on *HER3* gene transcription in TNBC cells requires a co-repressor. In terms of the molecular mechanism through panobinostat downregulates c-Myc in TNBC cells, our current data suggest that it may involve transcriptional regulation and/or protein degradation. This idea is supported by previous studies showing that SAHA and entinostat downregulated c-Myc at both mRNA and protein levels in AML cells^28^. Regardless, targeting of c-Myc may lead to upregulation of several RTKs, including HER3 (our studies) and PDGFRβ and VEGFR2^27^, thereby activating multiple signaling pathways to promote cell growth and survival. Thus, it is important to note that targeting c-Myc for therapeutic purposes can be complicated. Additional caution and careful consideration should be taken into account when developing c-Myc-targeted therapies for cancer treatment.

Recent studies show that HDACi, including panobinostat, induces STAT3 activation via upregulation of Leukemia Inhibitory Factor Receptor (LIFR) in BC cells, thereby limiting the efficacy of HDACi^45^. Consistent with these findings, our RNA-Seq analyses also discovered an increase of *LIFR* mRNA in panobinostat-treated MDA-MB-231 cells (Supplementary Table 1 and Supplementary Fig. 2a). However, the induction of *LIFR* was less significant than that of *erbB3*, and the increased p-STAT3 levels were not observed in all cells we tested (data not shown). In contrast, HER3 upregulation and Akt activation (evidenced by increased p-Akt) were consistently detected (Figs. 2e, f and 3d, f). Given that HER3 has been implicated as a major cause of treatment failure in various human cancers^25,37^, we believe that upregulation of HER3 and activation of its downstream Akt kinase critically contribute to the reduced cytotoxicity of panobinostat toward CL TNBC cells.

In summary, we demonstrated that panobinostat induced a quick and marked upregulation of HER3 in CL TNBC cells. The increased HER3 interacted with EGFR to activate both receptors and the downstream signaling pathways, on which CL TNBC cells relied to proliferate and survive. Targeting HER3 with our blocking Ab (4A7) or EGFR with its TKI (gefitinib) significantly enhanced the antitumor activity of panobinostat against CL TNBC. Thus, the feedback upregulation of HER3 confers new therapeutic approaches for CL TNBC. Our data suggest that panobinostat, combined with the therapeutics targeting HER3 or its dimerization partner (EGFR), may benefit patients with CL TNBC.

## Methods

### Antibodies, reagents, and chemicals

Antibodies used for western blots were as follows: HER3(cat#12708, 1:1000 dilution), p-HER3(Y1289) (cat#4791, 1:1000 dilution), PARP(cat#9542, 1:1000 dilution), Caspase-9(cat#9502, 1:1000 dilution), Caspase-3(cat#9665, 1:1000 dilution), EGFR(cat#4267, 1:1000 dilution), p-EGFR(Y1068) (cat#3777, 1:1000 dilution), MET(cat#8198, 1:1000 dilution), IGF-1R(cat#9750, 1:1000 dilution), p-Akt (S473) (cat#9271, 1:1000 dilution), Akt(cat#9272, 1:1000 dilution), p-STAT3(T705) (cat#9145, 1:1000 dilution), STAT3(cat#9139, 1:1000 dilution), p-ERK1/2(T202/Y204) (cat#9101, 1:1000 dilution), ERK1/2(cat#9102, 1:1000 dilution), c-Myc(cat#18583, 1:1000 dilution), p21^Waf1^(cat#2947, 1:1000 dilution), Ac-Histone H3 (Lys27) (cat#8173, 1:1000 dilution), Ki67(cat#9027, 1:400 dilution), HDAC1/2/3 (cat#34589; 57156; 85057, 1:1000 dilution) (Cell Signaling Technology, Inc., Beverly, MA, USA), and β-actin(A3853, 1:10000 dilution) (Sigma Chemical Co., St. Louis, MO). Panobinostat, gefitinib, and Akti-1/2 were brought from Selleck Chemicals. All other reagents were purchased from Sigma unless otherwise specified.

### Cell culture

Human TNBC cell lines (MDA-MB-468, MDA-MB-231, BT549, HCC1937, HCC70, HCC1806, HCC38, MDA-MB-157, MDA-MB-436, Hs578t, and HCC1143) were obtained from the American Type Culture Collection (ATCC). Human Mammary Epithelial Cells (HMEC) were kindly provided by Dr. Qiang Shen (Stanley S. Scott Cancer Center, School of Medicine, LSU Health Sciences Center, New Orleans, LA). TU-BcX-4IC primary cell line (4IC) established from the TNBC patient-derived xenograft (PDX) tumor was kindly provided by Dr. Matthew E. Burow^23^ (Section of Hematology & Medical Oncology, Department of Medicine, Tulane University School of Medicine, New Orleans, LA). All cell lines were maintained in DMEM/F-12 (1:1) medium (Sigma Chemical Co., St. Louis, MO, USA) containing 10% FBS (Sigma Chemical Co., St. Louis, MO, USA) and cultured in a 37 °C humidified atmosphere containing 95% air and 5% CO_2_ and split twice a week. Cells were free of mycoplasma contamination, determined by the MycoAlert™ Mycoplasma Detection Kit (Lonza Group Ltd., Basel, Switzerland).

### Cell proliferation assay

The MTS assay was performed using CellTiter 96® AQueous One Solution (Promega, Madison, WI, USA) following the manufacturer’s protocol. Measure the absorbance by a Synergy LX Multi-Mode Reader (Biotek, Winooski, VT, USA). Synergy was evaluated using CompuSyn software^46^.

### Quantification of apoptosis

Cell Death Detection ELISA Kit (Roche Diagnostics Corp., Indianapolis, IN, USA) was used to detect apoptotic cell death induced by different types of drugs as previously described^47^. In brief, cells treated with/without Panobinostat were lysed and centrifuged to produce a nucleosome-containing supernatant according to the manufacture’s protocol. 5ug total protein was used in each tested sample. The amount of histone-coupled DNA was quantified by measuring the absorbance.

### Western blot analysis

Cells were lysed in RIPA lysis buffer and sonicated at 4 °C. Protein concentration in extracts was measured using Bradford reagent. 30μg total cell lysates were subjected to western blot assays as described previously^47^. Protein samples were resolved by SDS-PAGE, 35 V overnight transferred onto PVDF membranes, and blocked with TBST containing 5% nonfat dry milk at RT for 30 min. The membranes were probed with the primary antibodies described in the figure legends.

### Reverse transcription (RT)-PCR and quantitative real-time (qRT)-PCR

Total RNA was extracted using RNeasy Mini Kit (QIAGEN, Hilden, Germany). First-strand cDNA was generated using High-Capacity cDNA Reverse Transcription Kit (Applied Biosystems, Foster City, CA, USA). 1 μg total RNA was used for cDNA reverse. mRNA expressions were examined using SYBR™ Green PCR Master Mix (Thermo Fisher Scientific Inc., Waltham, MA, USA).

### RNA sequencing and analysis

All the analysis was done by the Linux system. Raw sequence data coming from high throughput sequencing pipelines were checked for quality by FastQC (Version 0.11.9), and results were aggregated with MultiQC. The Reads from each sample were mapped to the reference genome with HISAT2. The mapped reads were further sorted, indexed, and compressed by samtools (https://github.com/samtools/samtools). The tool of GfeatureCounts was used to generate the gene counts. Differentially expressed genes analysis: R version 4.0.4 was used in the analysis. DESeq2 R package was used to identify differentially expressed genes (DEGs) between treatment and control groups. The cutoff values are|log2 fold change (FC)| > 1 and FDR < 0.01. Gene Ontology (GO) enrichment analysis of DEGs was done by "clusterProfiler" R package. Volcano plot of DEGs and bubble chart of significant pathways were generated with R package "ggplot2".

### Flow cytometric analysis

Flow cytometric analyses were performed to define the presentation of HER3 on cell membrane. In brief, cells were untreated or treated with panobinostat for 8 h, harvested by trypsinization and resuspended in PBS (1 × 10^7^cell/ml). Then, 100 µl cell suspension was incubated with 5 µl antibody PE-HER3 (Biolegend cat. 324706) or the relative isotype control (Biolegend cat. 400213) on ice in the dark for 30 min. Flow cytometric analyses were performed with a BD FACSymphony flow cytometer (San Jose, CA) and the mean fluorescent intensity of HER3 were calculated by the Flowjo software (FLOWJO, Ashland, OR).

### Detection of human receptor tyrosine kinase (RTK) phosphorylation

The human phospho-RTK array kit was purchased from R&D Systems (R&D Systems, Inc. Minneapolis, MN, USA). Cells were treated with DMSO or 25 nmol/L panobinostat for 8 h. 200 μg cell lysates were diluted and incubated with the Human Phospho-RTK Array for each experiment following the manufacturer’s introductions.

### Colony formation assay

Cells were plated in six-well plates (600 cells/well) and continuously incubated with/without panobinostat. Medium changed every three days. After 20 days, cells were fixed with methanol for 15 min and stained with 0.5% crystal violet for 15 min at room temperature. Cell colonies were counted using Image J software.

### Annexin-V apoptosis assay

Muse™ Annexin V & Dead Cell Assay was performed to define apoptosis. In brief, cells grown in 60-mm culture dishes were dissociated to obtain single-cell suspensions, and 100 μL of these suspensions was added to each tube together with 100 μL of the Muse™ Annexin V & Dead Cell Reagent (Luminex Corporate, Austin, TX, USA). The samples were mixed thoroughly and stained before being analyzed with the Guava® Muse® Cell Analyzer (Luminex Corporate, Austin, TX, USA).

### Co-immunoprecipitation

Cells were lysed with Pierce^TM^ IP Lysis buffer (Thermo Fisher Scientific Inc., Waltham, MA). The immunoprecipitations were performed overnight at 4 °C with a specific Ab or IgG, then incubated with protein A-agarose (Amersham Biosciences Corp., Amersham, UK) for 2 h. The protein A-agarose beads were washed with lysis buffer, and the immune complexes were detected by western blot assays as described above^48^.

### Live/Dead cell staining

Cells were stained with a Live/Dead Imaging kit (Life Technologies Carlsbad, CA, USA) following the manufacturer’s instructions as described previously^49^. In brief, cells were seeded in 6-well plate. Cells were stained with reagent provided by kit in RT for 15 min and then observed under EVOS FLoid Cell Imaging Station (Life Technologies Carlsbad, CA, USA).

### shRNA lentivirus production and infection

Lentiviruses containing shRNAs targeting *erbB3*(*HER3*) or *c-Myc* were used for *HER3* or *c-Myc* knockdown, respectively. Two specific lentiviruses shRNAs were purchased from Sigma (Sigma Chemical Co., St. Louis, MO, USA) and validated for specificity and efficiency (*erbB3*: TRCN0000219020 and TRCN0000009835; *c-Myc*: TRCN0000000136 and TRCN0000000138). The lentivirus production in HEK293T cells and transduction of targeted cells were carried out as described previously^50^. In brief, DAY1: Seed 4-5×106 293T cell /10 cm dish; DAY2: In 15 ml conical tube, mix DNA plus serum free DMEM (Expression vector DNA: 10 μg, Packaging DNA: 7.5 μg psPAX, 2.5 μg pMD2.G, SERUM FREE DMEM: 2.4 ml), then add 60 μl of PEI (1 μg/μl). Mix totally, incubate ~15 min at RT. Discard the old medium of 293 T cell and re-feed with 7 ml of 10% FBS DMEM. Add PEI /DNA complex dropwise to 293T cells. Incubate cells with DNA overnight. DAY3: Change transfection medium, add 5 ml 10% FBS DMEM/ DISH DAY4: Harvest viral supernatant at 48 h post-transfection.

### Chromatin immunoprecipitation

For analyzing protein binding to gene promoters, we used the Simple ChIP® plus Kit (Magnetic Bead) (Cell Signaling Technology, Inc., Beverly, MA, USA). Briefly, chromatin proteins were cross-linked to DNA. Cell lysates were subjected to sonication to reduce the size of DNA to approximately 200–1000 base pairs in length. Anti-c-Myc (E5Q6W, #18583, CST) and control rabbit antibody (#2729, CST) were used to immunoprecipitate the DNA-protein complex, followed by real-time PCR with primers targeting the core promoter regions of *erbB3*.

### Xenograft mouse experiments

Athymic nu/nu mice (Charles River Laboratories Inc., Wilmington, MA, USA) were maintained following the Institutional Animal Care and Use Committee (IACUC) procedures and guidelines. 5 × 10^5^ MDA-MB-231 cells were injected subcutaneously in the right flanks of 6 weeks old female athymic nude mice. After one week, tumor-bearing mice (tumor volume around 80 mm^3^) were randomized into four treatment groups (*n* = 5) and treated as follows: (1) vehicle (40% β-cyclodextrin in ddH_2_O, IP), (2) gefitinib (40 mg/kg, IP, once daily), (3) panobinostat(5 mg/kg, IP, once daily) and (4) gefitinib+Pano(G 40 mg/kg+P 5 mg/kg, IP, once daily) 6 days/week for 3 weeks. Tumor volume was calculated by the formula: Length × Width^2^ /2. At the end of the experiment, mice were sacrificed, and tumors were dissected for further experiments.

### Immunohistochemistry (IHC) and scoring of stained slides

IHC assays were performed as we described previously^49^. In brief, paraffin sections were deparaffinized, antigen retrieved and immunohistochemically stained for HER3 (cat# 12708,1:200 dilution), p-HER3(cat# 4791,1:500 dilution); Ki67 rabbit mAb (cat# 9027, 1:400 dilution); Cleaved caspase-3 rabbit pAb (cat# 9661, 1: 400 dilution). Digital images were captured using a Nikon Microscope. Quantification of IHC analysis: Image J and Image J plugin IHC profiler were applied to quantify IHC staining analysis as reported^51^. The mean intensity of indicated protein was measured using Image J. Three fields of each group were assessed.

### Statistical analyses

Values are the mean ± standard deviation (SD) of at least three independent experiments. The unpaired two-tailed Student’s *t* test and two-way ANOVA were done using Graph Pad Prism software (v9.0.0). *p* < 0.05 was considered statistically significant.

### Reporting summary

Further information on research design is available in the Nature Research Reporting Summary linked to this article.

### Supplementary information

REPORTING SUMMARY

Supplementary Information

Data Sets

## Data Availability

RNA sequencing data is available via the Gene Expression Omnibus (https://www.ncbi.nlm.nih.gov/geo/query/acc.cgi?acc=GSE236280). Data generated from online tool GEPIA2 contain Match TCGA normal and GTEx datasets, Breast Cancer Gene-Expression Miner v4.7 database and Kaplan-Meier plotter database are publicly available. (http://gepia2.cancer-pku.cn/#index, https://kmplot.com/analysis/, http://bcgenex.ico.unicancer.fr/BC-GEM/GEM-Accueil.php?js=1) Source data are provided with this paper in the Supplementary Information.

## Code availability

The underlying code is available on request from the corresponding author.
