# Supplementary material for: HER3 targeting augments the efficacy of panobinostat in claudin-low triple-negative breast cancer cells
Source: NPJ Precis Oncol. 2023 Aug 3;7:72. doi: 10.1038/s41698-023-00422-8 (PMC10400567; doi:10.1038/s41698-023-00422-8)
Supplement: Supplementary file 1 — REPORTING SUMMARY [file 41698_2023_422_MOESM1_ESM.pdf]

nature research

Corresponding author(s): DBPR-NPJPRECISIONONCOLOGY-03083R

Last updated by author(s): 03-31-2023

Reporting Summary

Nature Research wishes to improve the reproducibility of the work that we publish. This form provides structure for consistency and transparency in reporting. For further information on Nature Research policies, see our Editorial Policies and the Editorial Policy Checklist.

Statistics

For all statistical analyses, confirm that the following items are present in the figure legend, table legend, main text, or Methods section.

n/a	Confirmed	
☐	☒	The exact sample size (*n*) for each experimental group/condition, given as a discrete number and unit of measurement
☐	☒	A statement on whether measurements were taken from distinct samples or whether the same sample was measured repeatedly
☐	☒	The statistical test(s) used AND whether they are one- or two-sided<br>*Only common tests should be described solely by name; describe more complex techniques in the Methods section.*
☒	☐	A description of all covariates tested
☒	☐	A description of any assumptions or corrections, such as tests of normality and adjustment for multiple comparisons
☐	☒	A full description of the statistical parameters including central tendency (e.g. means) or other basic estimates (e.g. regression coefficient) AND variation (e.g. standard deviation) or associated estimates of uncertainty (e.g. confidence intervals)
☐	☒	For null hypothesis testing, the test statistic (e.g. *F*, *t*, *r*) with confidence intervals, effect sizes, degrees of freedom and *P* value noted<br>*Give P values as exact values whenever suitable.*
☒	☐	For Bayesian analysis, information on the choice of priors and Markov chain Monte Carlo settings
☒	☐	For hierarchical and complex designs, identification of the appropriate level for tests and full reporting of outcomes
☒	☐	Estimates of effect sizes (e.g. Cohen's *d*, Pearson's *r*), indicating how they were calculated

Our web collection on statistics for biologists contains articles on many of the points above.

Software and code

Policy information about availability of computer code

Data collection	QuantStudio 12K Flex Real-Time PCR System; Bio-Rad iQ5 program; BD FACSymphony flow cytometer ; Synergy 2 of BioTeK
Data analysis	Excel 2013; SPSS version 16.0; Prism Graphpad (v9.0); ImageJ software (v2.0.0);Flowjo software ;FastQC (Version 0.11.9),MultiQC and R package

For manuscripts utilizing custom algorithms or software that are central to the research but not yet described in published literature, software must be made available to editors and reviewers. We strongly encourage code deposition in a community repository (e.g. GitHub). See the Nature Research guidelines for submitting code & software for further information.

Data

Policy information about availability of data

All manuscripts must include a data availability statement. This statement should provide the following information, where applicable:

- Accession codes, unique identifiers, or web links for publicly available datasets
- A list of figures that have associated raw data
- A description of any restrictions on data availability

All data supporting the findings of our studies are available within this article, the Supplementary Information file, and the Reporting Summary of this article.

Field-specific reporting

Please select the one below that is the best fit for your research. If you are not sure, read the appropriate sections before making your selection.

☒ Life sciences ☐ Behavioural & social sciences ☐ Ecological, evolutionary & environmental sciences

For a reference copy of the document with all sections, see nature.com/documents/nr-reporting-summary-flat.pdf

Life sciences study design

All studies must disclose on these points even when the disclosure is negative.

Sample size	Sample size was determined based on published paper and previous experience. For in vitro studies, a sample size of n=3 would allow for adequate analysis to reach meaningful conclusions of the data. For in vivo studies, a bigger sample size (n=5) was used to compensate for the higher natural variance in vivo.
Data exclusions	No data was excluded.
Replication	Data showed a representative of three independent experiments. All the experimental findings were reliably reproduced.
Randomization	All mice were randomly allocated into experimental groups. All cells used throughout the study were differentially treated and analyzed in parallel to minimize experimental variation.
Blinding	Blinding was used in in vivo data analysis, including immunohistochemical result analysis.

Reporting for specific materials, systems and methods

We require information from authors about some types of materials, experimental systems and methods used in many studies. Here, indicate whether each material, system or method listed is relevant to your study. If you are not sure if a list item applies to your research, read the appropriate section before selecting a response.

Materials & experimental systems

n/a	Involved in the study	
☐	☒	Antibodies
☐	☒	Eukaryotic cell lines
☒	☐	Palaeontology and archaeology
☐	☒	Animals and other organisms
☒	☐	Human research participants
☒	☐	Clinical data
☒	☐	Dual use research of concern

Methods

n/a	Involved in the study	
☒	☐	ChIP-seq
☐	☒	Flow cytometry
☒	☐	MRI-based neuroimaging

Antibodies

Antibodies used	Rabbit anti-HER3 Antibody (Cell Signaling Technology, Cat. #12708, 1:1000 for western blot, 1:100 for immunohistochemistry),Rabbit anti-Phospho-HER3(Y1289) (Cell Signaling Technology, Cat. #4791, 1:1000 for western blot, 1:80 for immunohistochemistry) Rabbit anti-EGFR (Cell Signaling Technology, Cat. #4267, 1:1000 for western blot), Rabbit anti-Phospho-EGFR (Y1068)(Cell Signaling Technology, Cat. #2234, 1:1000 for western blot), Rabbit anti-Akt (Cell Signaling Technology, Cat. #4691, 1:1000 for western blot), Rabbit anti-Phospho-Akt (S473) (Cell Signaling Technology, Cat. #4060, 1:1000 for western blot), Rabbit anti-HDAC1(Cell Signaling Technology, Cat. #34589, 1:1000 for western blot), Rabbit anti-HDAC2(Cell Signaling Technology, Cat. #57156, 1:1000 for western blot), Rabbit anti-HDAC3(Cell Signaling Technology, Cat. #85057, 1:1000 for western blot), Rabbit anti-p21 (Cell Signaling Technology, Cat. #2947, 1:1000 for western blot), Rabbit anti-Acetyl-Histone H3 (Lys27)(Cell Signaling Technology, Cat. #8137, 1:1000 for western blot)Rabbit anti-PARP (Cell Signaling Technology, Cat. #9532, 1:1000 for western blot),Rabbit anti-Caspase-9 (Cell Signaling Technology, Cat. #9502, 1:1000 for western blot),Rabbit anti-Caspase-3 (Cell Signaling Technology, Cat. #9662, 1:1000 for western blot),Rabbit anti-Cleaved-Caspase-3 (Cell Signaling Technology, Cat. #9661, 1:350 for immunohistochemistry), Rabbit anti-Cleaved-Caspase-3 (Cell Signaling Technology, Cat. #9661, 1:350 for immunohistochemistry), Rabbit anti-Ki67 (Cell Signaling Technology, Cat. #9027, 1:400 for immunohistochemistry), Rabbit anti-c-Myc (Cell Signaling Technology, Cat. #18583, 1:1000 for western blot, 1:100 for Chromatin immunoprecipitation), Mouse anti-Stat3 (Cell Signaling Technology, Cat. #9139, 1:1000 for western blot), Rabbit anti-Phospho-Stat3(T705) (Cell Signaling Technology, Cat. #52075, 1:1000 for western blot), Rabbit anti-ERK1/2 (Cell Signaling Technology, Cat. #4695, 1:1000 for western blot), Rabbit anti-Phospho-ERK1/2 (T202/Y204) (Cell Signaling Technology, Cat. #4370, 1:1000 for western blot) and Mouse anti-β-actin (Sigma, Cat. #A5316, 1:5000 for western blot), Goat anti-Rabbit IgG (H+L) Secondary Antibody, HRP (Thermo Scientific, Cat. #31460, 1:5000 for western blot), Goat anti-Mouse IgG (H+L) Secondary Antibody, HRP (Thermo Scientific, Cat. #31430, 1:5000 for western blot).
Validation	All antibodies used in this study were obtained from commercial sources and validated according to manufacturers' instruction.

Eukaryotic cell lines

Policy information about cell lines

Cell line source(s)	Human TNBC cell lines (MDA-MB-468, MDA-MB-231, BT549, HCC1937, HCC70, HCC1806, HCC38, MDA-MB-157, MDA-MB-436, Hs578t, and HCC1143) were obtained from the American Type Culture Collection (ATCC). Human Mammary Epithelial Cells (HMEC) were kindly provided by Dr. Qiang Shen (Stanley S. Scott Cancer Center, School of Medicine, LSU Health Sciences Center, New Orleans, LA). TU-BcX-4IC primary cell line (4IC) established from the TNBC patient-derived xenograft (PDX) tumor was described previously20. The 4IC cells were kindly provided by Dr. Matthew E. Burow (Section of Hematology & Medical Oncology, Department of Medicine, Tulane University School of Medicine, New Orleans, LA).
Authentication	Cells were authenticated using Short Tandem Repeat (STR) analysis with PowerPlex® 18D System from Promega (Madison, WI, USA).
Mycoplasma contamination	Cells were free of mycoplasma contamination, determined by the MycoAlert™ Mycoplasma Detection Kit (Lonza Group Ltd., Basel, Switzerland) every three months.
Commonly misidentified lines (See ICLAC register)	No commonly misidentified cell lines were used in this study

Animals and other organisms

Policy information about studies involving animals; ARRIVE guidelines recommended for reporting animal research

Laboratory animals	Female 4 to 6-week-old Athymic nu/nu mice were purchased from Charles River Laboratories Inc.(Wilmington, MA, USA). All animal procedures were conducted under the approval of the Institutional Animal Care and Use Committee (IACUC) of Louisiana State University.
Wild animals	No wild animals were used in the study.
Field-collected samples	At experimental endpoint, tumors were excised and formalin-fixed for paraffin embedding later.
Ethics oversight	The animal studies were approved by the Institutional Animal Care and Use Committee (IACUC) of Louisiana State University.

Note that full information on the approval of the study protocol must also be provided in the manuscript.

Flow Cytometry

Plots

Confirm that:

- [x] The axis labels state the marker and fluorochrome used (e.g. CD4-FITC).
- [x] The axis scales are clearly visible. Include numbers along axes only for bottom left plot of group (a 'group' is an analysis of identical markers).
- [x] All plots are contour plots with outliers or pseudocolor plots.
- [x] A numerical value for number of cells or percentage (with statistics) is provided.

Methodology

Sample preparation	Flow cytometric analyses were performed to define the presentation of HER3 on cell membrane. In brief, cells were untreated or treated with panobinostat for 8 hours, harvested by trypsinization and resuspended in PBS. Then, 100 µl cell suspension was incubated with 5 µl antibody PE-HER3 (Biolegend cat. 324706) or the relative isotype control (Biolegend cat. 400213) on ice in the dark for 30 min. Flow cytometric analyses were performed with a BD FACSymphony flow cytometer (San Jose, CA) and the mean fluorescent intensity of HER3 were calculated by the Flowjo software (FLOWJO, Ashland, OR).
Instrument	BD FACSymphony flow cytometer (San Jose, CA)
Software	Flowjo software (FLOWJO, Ashland, OR)
Cell population abundance	1x10^7 cells/ml in PBS suspension. cell number and viability was determined by cell counter
Gating strategy	Intact cells were gated according to the FSC-A and SSC-A. Doublets were excluded by FSC-H and FSC-A. Fluorescent intensity were measured for all single cells.

- [ ] Tick this box to confirm that a figure exemplifying the gating strategy is provided in the Supplementary Information.
