# Supplementary material for: HER3 targeting augments the efficacy of panobinostat in claudin-low triple-negative breast cancer cells
Source: NPJ Precis Oncol. 2023 Aug 3;7:72. doi: 10.1038/s41698-023-00422-8 (PMC10400567; doi:10.1038/s41698-023-00422-8)
Supplement: Supplementary file 2 — Supplementary Information [file 41698_2023_422_MOESM2_ESM.pdf]

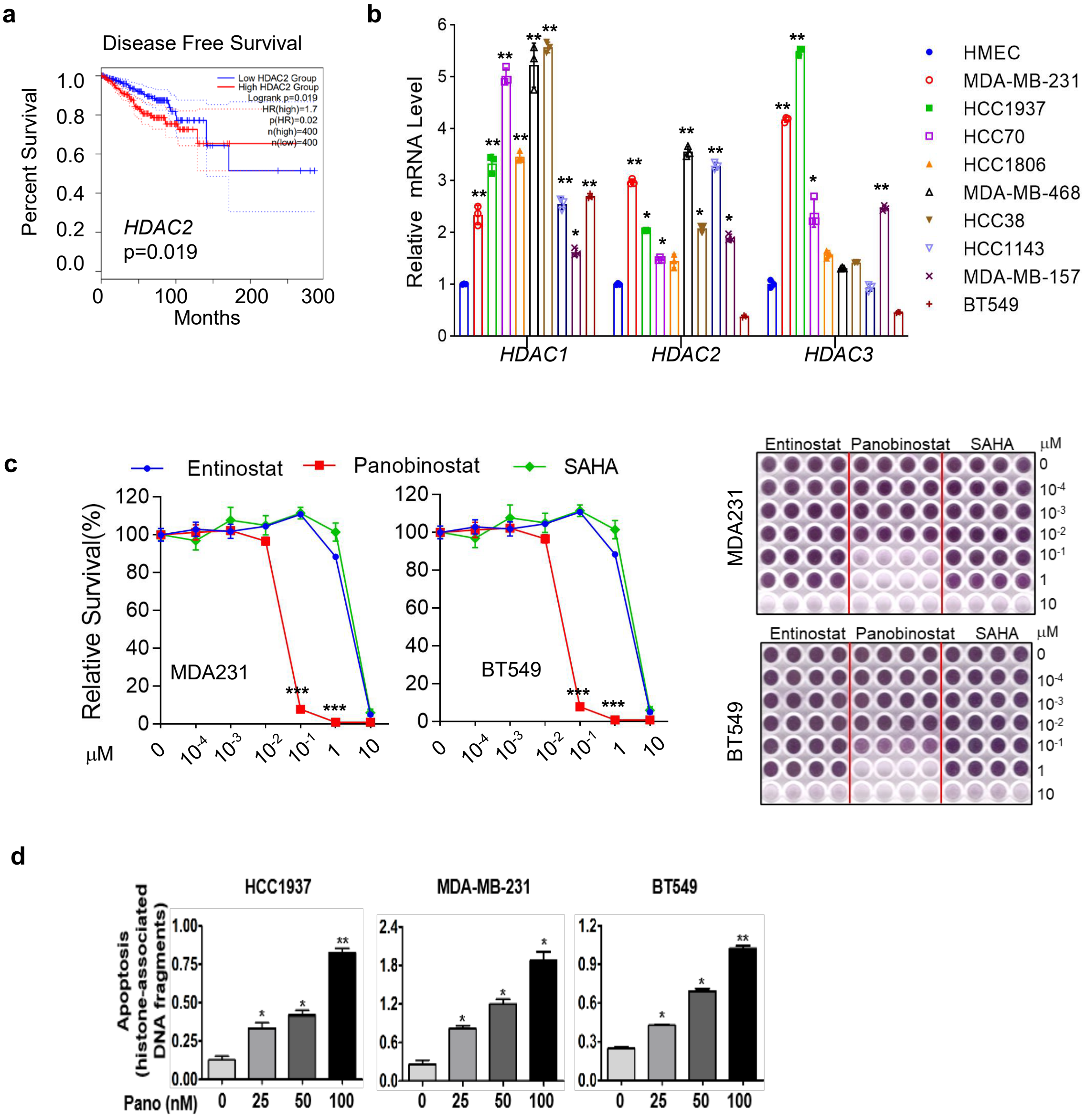

a, Kaplan-Meier survival curves for Disease Free Survival (DFS) of breast cancer patients from TCGA dataset, correlated to *HDAC2* mRNA level. The log rank test was used to compare survival curve for high (red) and low (blue) *HDAC2*. **b**, Total RNAs isolated from HMEC and TNBC cells were subjected to RT-qPCR measurement of the expression levels of HDAC1/2/3 mRNA, which were normalized to GAPDH levels. Data shows a representative of three independent experiments. Bars, SD. *, $p<0.05$, **, $p<0.01$ vs HMEC). **c,** Examination of cell proliferation/survival in response to HDACis (Entinostat, Panobinostat and SAHA), MDA231 and BT549 cells were treated with HDACi at indicated concentrations for 72 hours. The percentages of surviving cells were determined by MTS assays. Data shows a representative of three independent experiments. Bars, SD. ***, $p<0.005$ vs Entinostat and SAHA. **d**, TNBC cells (HCC1937, MDA-MB-231 and BT549) were seeded in 60mm dishes for culture overnight with DMEM/F12 medium containing 10% FBS. Cells were then treated with indicated concentrations of panobinostat (Pano_nmol/L) for 48 hours. Both adherent and non-adherent cells were collected, and cell lysates were subjected to apoptosis ELISA. Data shows a representative of three independent experiments. Bars, SD. *, $p<0.05$, **, $p<0.01$ vs untreated control, unpaired two-tailed Student's t-test.

Supplementary Figure 1

a

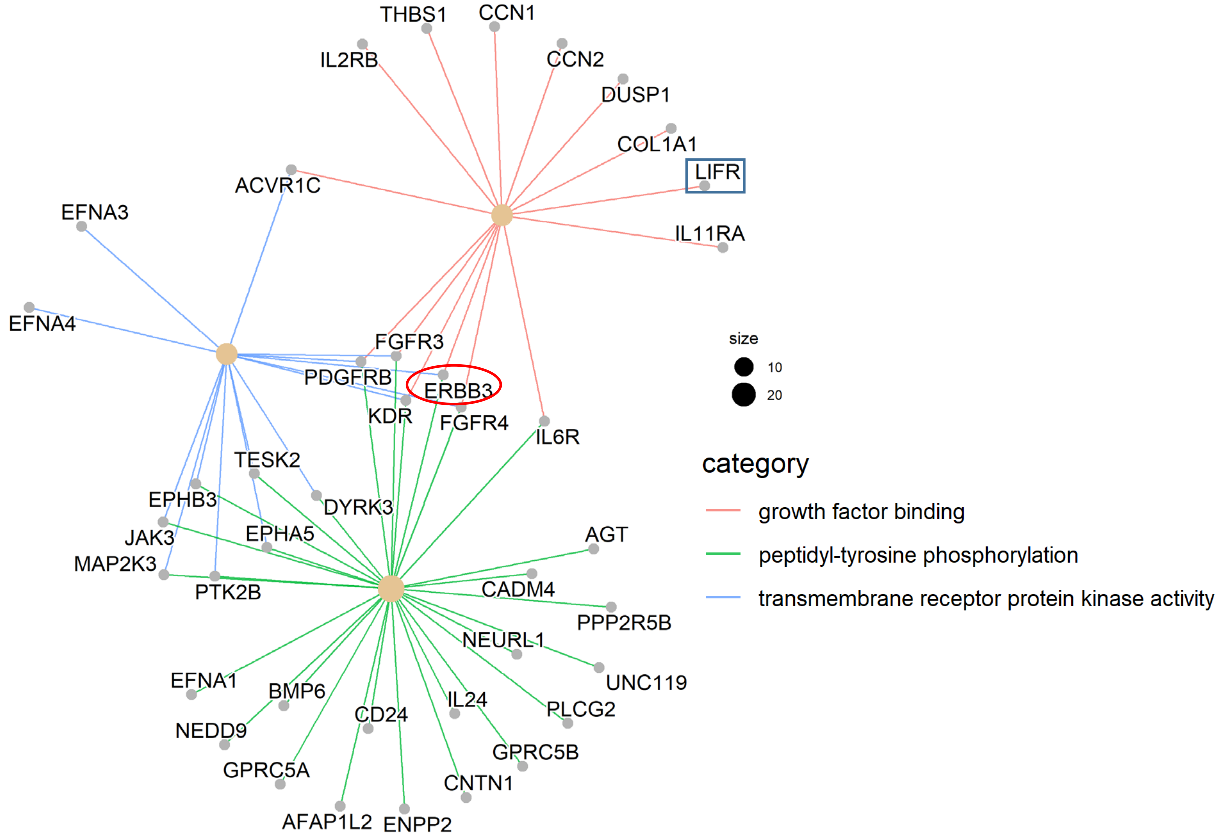

b

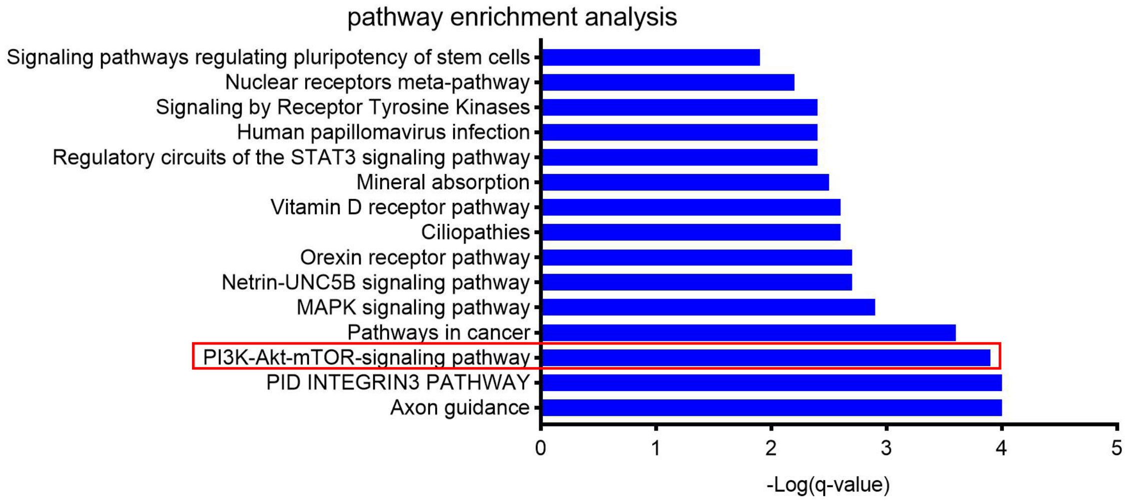

a, DEGs in the GO term of peptidyl-tyrosine phosphorylation(GO:0018108), transmembrane receptor protein tyrosine kinase activity (GO:0004714) and growth factor binding (GO:0019838) . **b,** Pathway enrichment analysis, top 15 clusters with their representative enriched terms.

Supplementary Figure 2

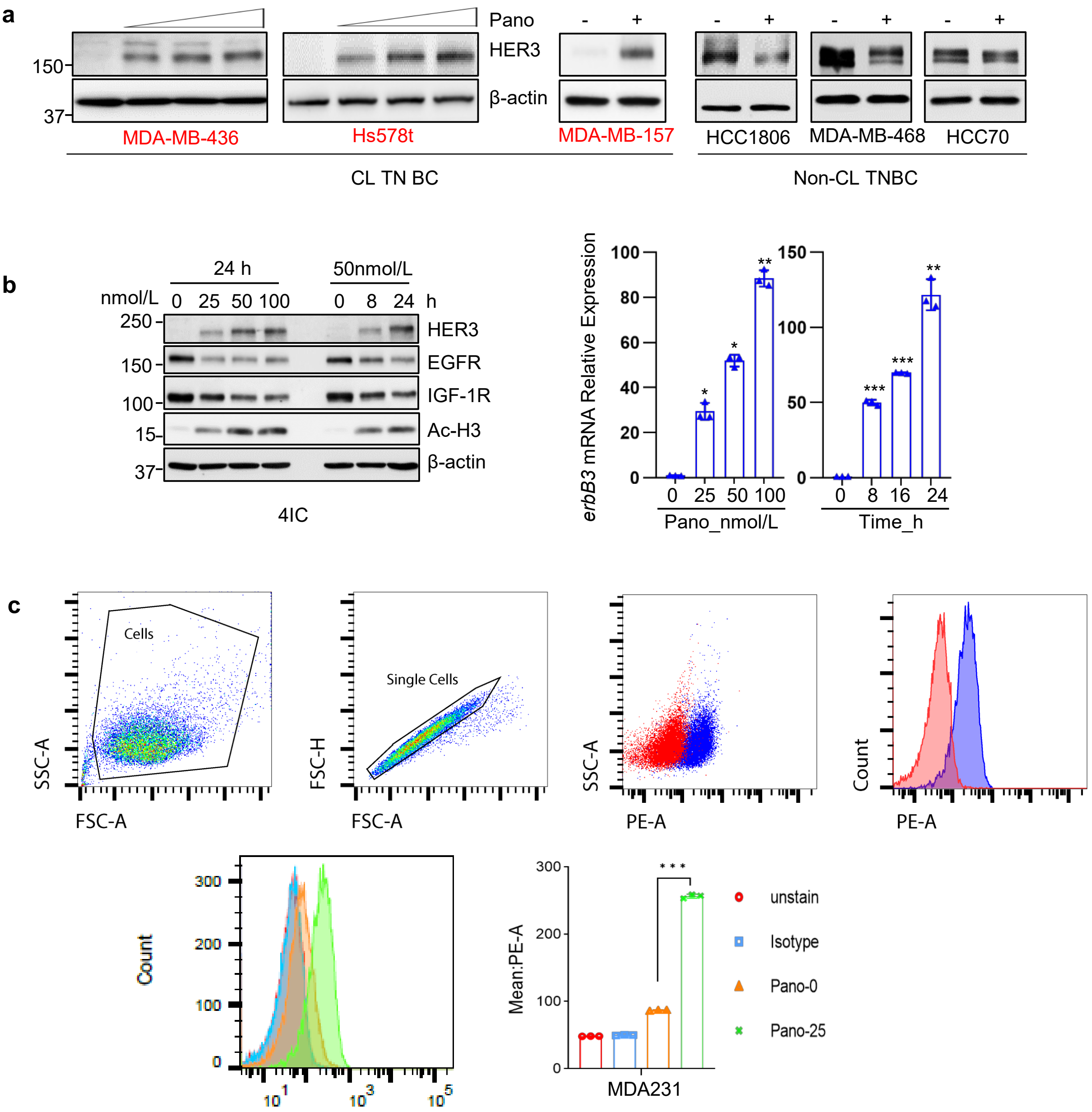

a, CL TNBC cells (MDA-MB-436, Hs578t, MDA-MB-157) and non-CL TNBC cells (HCC1806, MDA-MB-468 and HCC70) were seeded in 60mm dishes with DMEM/F12 medium containing 10% FBS. The following day, cells were treated with increased or fixed concentrations of panobinostat (Pano) for 24 hours. The cells were then collected for western blot analyses of HER3, β-actin. **b,** TNBC PDX 4IC cells were seeded in 60mm dishes. The following day, cells were treated with indicated concentrations of panobinostat (Pano) for 24 hours or with 50 nmol/L of panobinostat for indicated hours. Cells were collected. 30μg protein from whole-cell lysates was used for western blot assays with specific antibodies directed against HER3, EGFR, IGF-1R, Ac-H3 or β-actin (**Right**). Total RNAs isolated from the cells were subjected to RT-qPCR measurement of the expression levels of *erbB3* mRNA, which were normalized to *GAPDH* levels. Data shows a representative of three independent experiments. Bars, SD. *, $p<0.05$, **, $p<0.01$, ***, $p<0.005$ vs untreated control (**Left**). **c,** Flow cytometric analysis of HER3 on cell membrane of MDA231 cells with or without panobinostat treatment. Cells were stained with PE-conjugated HER3 or with isotype-matched IgG control. Gating strategies(**Upper**). Data shows a representative of three independent experiments with similar results. The mean fluorescent intensity of HER3 was quantified. Values were expressed as the mean ± SD of three independent experiments. $p<0.005$ vs untreated control.(**Lower**)

Supplementary Figure 3

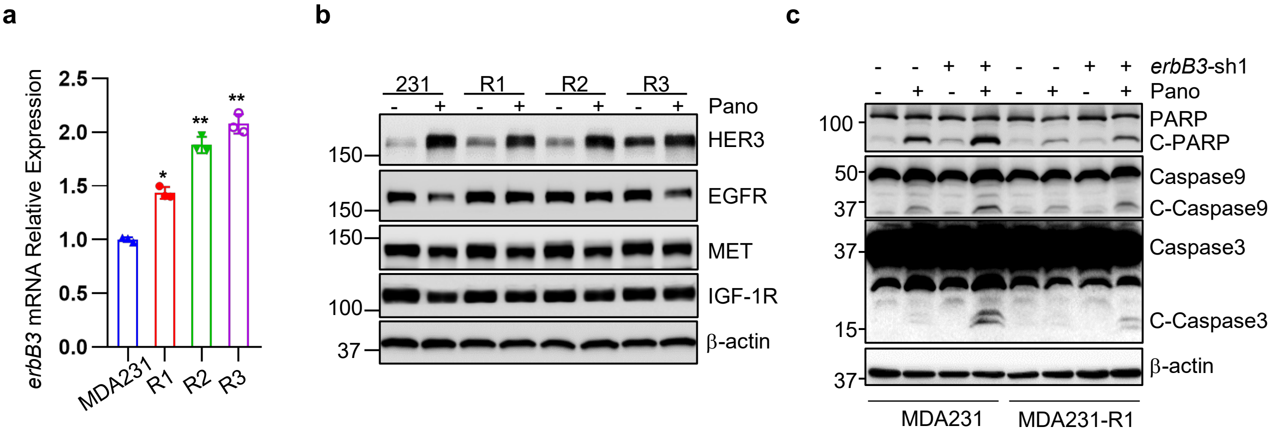

a, MDA-MB-231 parental cell(MDA231) and its panobinostat resistant sublines (R1, R2, and R3)-derived through long-term treatment of panobinostat were collected. Total mRNA was isolated from the cells and 1 μg mRNA was used in RT reaction to obtain cDNA. *erbB3* mRNA levels were analyzed by qPCR and normalized to *GAPDH* levels. All data are presented as means ± S.D. *, $p<0.05$, **, $p<0.01$, vs. control group. **b,** MDA-MB-231 parental cell (231) and its panobinostat resistant sublines were treated with 25nmol/L of panobinostat (Pano) for 24 hours. Cells were collected and examined by western blot assays of HER3, EGFR, MET and IGF-1R, or β-actin. **c,** MDA-MB-231 parental cell (MDA231) and panobinostat resistant subline (MDA231-R1) cell were transiently transduced with shRNA sequences targeting *HER3* (*erbB3*) construct (*erbB3*-sh1) or scramble control vector (src) for 24 hours, following treated with or without Pano (50nmol/L) for 48 hours. Cell lysates were subjected to western blot assay with specific antibodies directed against PARP, Caspase-9, and Caspase-3. β-actin was used as the loading control.

Supplementary Figure 4

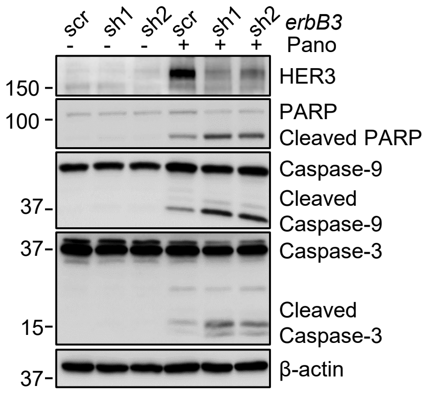

TNBC PDX 4IC cells transiently transduced the lentivirus containing either a scramble (scr) shRNA or erbB3 specific shRNA (sh1 or sh2) were treated with vehicle (DMSO) control (-) or 50nmol/L of panobinostat (+ Pano) for another 48 hours. Cells were collected and examined by western blot assays of HER3, PARP, Caspase-9, Caspase-3 or β-actin.

Supplementary Figure 5

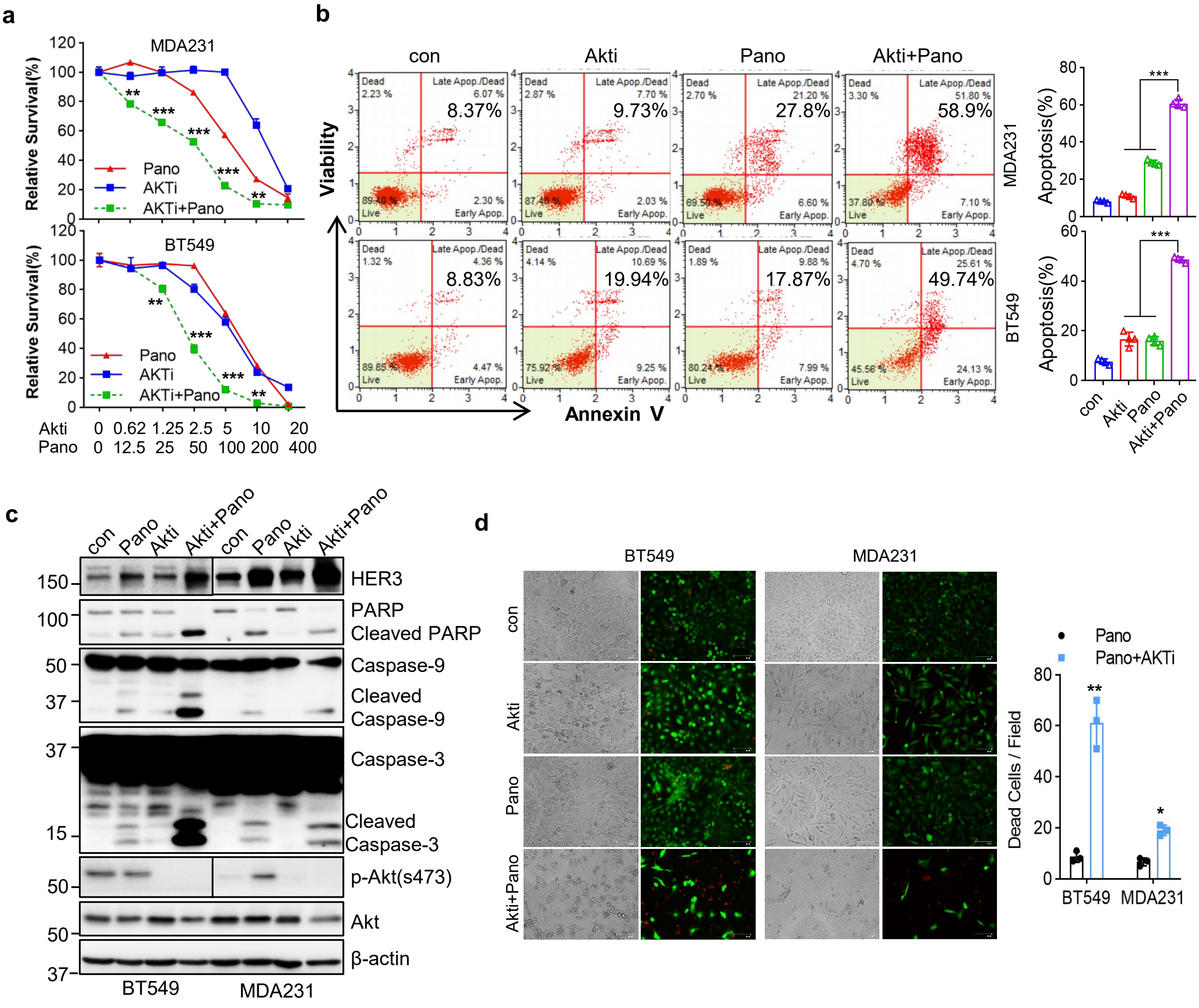

a, BT549 and MDA-MB-231(MDA231) cells were seeded in 96-well plates with fresh DMEM/F12 medium, the following day cells were treated with indicated concentrations of panobinostat (Pano), Akt inhibitor (Akti) or their combination (Akti+Pano) for 72 hours, DMSO was used as control treatment. The percentages of surviving cells from each cell line relative to controls, defined as 100% survival were determined by MTS assays. Data shows the representative of three independent experiments. **b,** BT549 and MDA-MB-231(MDA231) cells were treated with DMSO, Pano, Akti or their combination for 48 hours, and analyzed for cell apoptosis through Muse™ Annexin V & Dead Cell Assay. **c,** BT549 and MDA-MB-231 (MDA231)cells were seeded in 6-well plate, treated with DMSO (con), Panobinostat (Pano), Akt inhibitor (Akti), or their combination (Akti+Pano), respectively. After 48 hours, cells were subjected to western blot analyses with specific antibodies directed against HER3, PARP, Caspase-9, Caspase-3, p-Akt, Akt or β-actin. **d,** BT549 and MDA-MB-231 (MDA231) cells were seeded in 6-well plate, treated with DMSO, Pano, Akti or their combination, respectively. After 48 hours, cells were subjected to the LIVE/DEAD Cell Imaging. Green color indicated live cells; red color indicated dead cells. Dead cells was counted at three random fields. All data are presented as means ± S.D. $*p < 0.05$; $**p < 0.01$; $***p < 0.001$.

Supplementary Figure 6

a

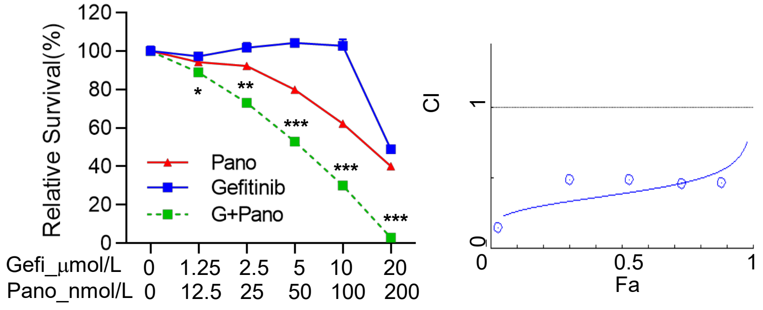

b

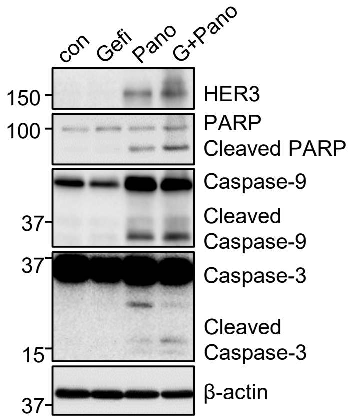

a, TNBC PDX 4IC cells were seeded in 96-well plates (5000 cells/well) with fresh DMEM/F12 medium, the following day cells were treated with indicated concentrations of panobinostat (Pano) or gefitinib (Gefi) or their combination(G+Gefi) for 72 hours. The percentages of surviving cells from each cell line relative to controls, defined as 100% survival were determined by MTS assays. Data shows the representative of three independent experiments. *, p<0.05; **, p<0.01, ***, p<0.005 *vs* each single agent . The combination index (CI) curves were calculated using Calcusyn software. A lower CI, when it is less than 1, indicates stronger evidence in favor of synergy. **b,** TNBC PDX 4IC cells were seeded in 6-well plate, treated with DMSO (con), 2μmol/L gefitinib (Gefi), 50nmol/L of panobinostat (Pano) or their combination(G+Pano), respectively. After 48 hours cells were collected and examined by western blot assays of HER3, PARP, Caspase-9, Caspase-3 or β-actin.

Supplementary Figure 7

**q-PCR primer**	
GAPDH-F	5′- GTCTCCTCTGACTTCAACAGCG-3‘
GAPDH-R	5′-ACCACCCTGTTGCTGTAGCCAA-3‘
erbB3-F	5′-CTATGAGGCGATACTTGGAACGG-3‘
erbB3-R	5′-GCACAGTTCCAAAGACACCCGA-3‘
c-Myc-F	5′-AAAGGCCCCCAAGGTAGTTA-3‘
c-Myc-R	5′-GCACAAGAGTTCCGTAGCTG-3‘
HDAC1-F	5'-CCAGTATTCGATGGCCTGTT-3'
HDAC1-R	5'-CTCGGACTTCTTTGCATGGT-3'
HDAC2-F	5´-ACCTCCGATTCCGAGCTTT- 3´
HDAC2-R	5´-CCGCTCACCGTCGTAGTAGT- 3´
HDAC3-F	5'-GGGACATTATTGGCAGTG-3'
HDAC3-R	5'-GGATTCAGGTGTTAGGGAG-3'
**ChIP primer**	
erbb3-P1-F	5′-TTCCTCCCTCCCTCTCTCTC-3‘
erbb3-P1-R	5′-TAGGACATCGAGGCAAGAGC-3‘
erbb3-P2-F	5′-AAGGGATTTGAAATGCAAGG-3‘
erbb3-P2-R	5′-GACGCGGAGAGGACACTAGA-3‘
**shRNA**	
erbb3-shRNA	Target Sequence
TRCN0000219020	GACTAGACATCAAGCATAATC
TRCN0000009835	AGGTTAGGAGTAGATATTGA
c-Myc-shRNA	Target Sequence
TRCN0000000136	CGAGCAGATGAACTTAACAAT
TRCN0000000138	GAACCTGATGAGAGAAAGAAT

Supplementary Table 1					
id	log2FoldChange	pvalue	padj	SYMBOL	ENTREZID
ENSG00000127995	-1.770903118	4.74E-09	2.66E-05	**CASD1**	64921
ENSG00000172977	-1.200206538	5.01E-09	2.66E-05	**KAT5**	10524
ENSG00000174151	0.882586977	2.54E-09	2.66E-05	**CYB561D1**	284613
ENSG00000066827	-1.378449979	6.75E-09	2.69E-05	**ZFAT**	57623
ENSG00000100023	-0.88439947	9.10E-09	2.78E-05	**PPIL2**	23759
ENSG00000119772	-0.992845086	1.40E-08	2.78E-05	**DNMT3A**	1788
ENSG00000131899	0.580582777	1.35E-08	2.78E-05	**LLGL1**	3996
ENSG00000178104	0.851439972	1.23E-08	2.78E-05	**PDE4DIP**	9659
ENSG00000170776	0.697072763	1.66E-08	2.95E-05	**AKAP13**	11214
ENSG00000124664	-1.389854613	2.55E-08	3.74E-05	**SPDEF**	25803
ENSG00000204084	1.083949717	2.59E-08	3.74E-05	**INPP5B**	3633
ENSG00000211455	0.964512601	2.89E-08	3.74E-05	**STK38L**	23012
ENSG00000234602	-2.004390535	3.05E-08	3.74E-05	**MCIDAS**	345643
ENSG00000030110	0.820428279	3.55E-08	4.04E-05	**BAK1**	578
ENSG00000130749	-1.139535671	4.47E-08	4.46E-05	**ZC3H4**	23211
ENSG00000174804	1.164720517	4.37E-08	4.46E-05	**FZD4**	8322
ENSG00000085719	0.76324661	5.44E-08	4.74E-05	**CPNE3**	8895
ENSG00000121743	1.030753483	6.06E-08	4.74E-05	**GJA3**	2700
ENSG00000127328	0.997334726	6.01E-08	4.74E-05	**RAB3IP**	117177
ENSG00000161010	0.875067663	5.88E-08	4.74E-05	**MRNIP**	51149
ENSG00000165868	3.701369646	6.24E-08	4.74E-05	**HSPA12A**	259217
ENSG00000010319	2.674476889	8.27E-08	6.00E-05	**SEMA3G**	56920
ENSG00000056558	-0.663432414	9.38E-08	6.51E-05	**TRAF1**	7185
ENSG00000101928	0.989120508	9.84E-08	6.54E-05	**MOSPD1**	56180
ENSG00000059145	-0.84847929	1.08E-07	6.92E-05	**UNKL**	64718
ENSG00000090989	0.530939252	1.17E-07	7.15E-05	**EXOC1**	55763
ENSG00000168497	0.814459697	1.23E-07	7.29E-05	**CAVIN2**	8436
ENSG00000006459	1.533871828	1.42E-07	7.43E-05	**KDM7A**	80853
ENSG00000111424	1.445260034	1.39E-07	7.43E-05	**VDR**	7421
ENSG00000134313	0.707632796	1.56E-07	7.43E-05	**KIDINS220**	57498
ENSG00000149636	-0.533076961	1.53E-07	7.43E-05	**DSN1**	79980
ENSG00000153879	-0.685528222	1.58E-07	7.43E-05	**CEBPG**	1054
ENSG00000179943	-1.109068982	1.47E-07	7.43E-05	**FIZ1**	84922
ENSG00000213563	-0.925888416	1.45E-07	7.43E-05	**C8orf82**	414919
ENSG00000141664	0.981730188	1.70E-07	7.75E-05	**ZCCHC2**	54877
ENSG00000105576	-0.814651716	1.84E-07	8.15E-05	**TNPO2**	30000
ENSG00000008405	0.677388819	1.99E-07	8.59E-05	**CRY1**	1407
ENSG00000114859	1.271900764	2.08E-07	8.66E-05	**CLCN2**	1181
ENSG00000136436	0.645176248	2.12E-07	8.66E-05	**CALCOCO2**	10241
ENSG00000163328	1.67978097	2.20E-07	8.76E-05	**GPR155**	151556
ENSG00000170214	1.727843391	2.97E-07	0.000116	**ADRA1B**	147
ENSG00000119899	0.974556118	3.48E-07	0.00013	**SLC17A5**	26503
ENSG00000165322	0.586706828	3.51E-07	0.00013	**ARHGAP12**	94134
ENSG00000085840	-0.747666761	3.91E-07	0.000139	**ORC1**	4998
ENSG00000253522	0.569675506	3.85E-07	0.000139	**MIR3142HG**	1.07E+08
ENSG00000099625	1.531770692	4.37E-07	0.000152	**CBARP**	255057
ENSG00000183576	0.579897108	4.58E-07	0.000156	**SETD3**	84193
ENSG00000054598	-1.03360025	4.78E-07	0.000159	**FOXC1**	2296

ENSG00000123416	-0.472599893	5.09E-07	0.000161	**TUBA1B**	10376
ENSG00000137819	0.969867391	5.05E-07	0.000161	**PAQR5**	54852
ENSG00000143479	1.271420262	5.15E-07	0.000161	**DYRK3**	8444
ENSG00000160818	-0.839307282	5.27E-07	0.000161	**GPATCH4**	54865
ENSG00000181035	1.433383196	5.35E-07	0.000161	**SLC25A42**	284439
ENSG00000196227	-1.022917641	5.67E-07	0.000167	**FAM217B**	63939
ENSG00000071242	2.657289956	5.87E-07	0.00017	**RPS6KA2**	6196
ENSG00000066629	2.614303413	6.27E-07	0.000179	**EML1**	2009
ENSG00000100055	2.141339394	6.40E-07	0.000179	**CYTH4**	27128
ENSG00000048471	1.20355235	6.70E-07	0.000183	**SNX29**	92017
ENSG00000197483	-1.081480229	6.75E-07	0.000183	**ZNF628**	89887
ENSG00000109654	0.984755343	6.87E-07	0.000183	**TRIM2**	23321
ENSG00000114573	0.992912091	7.28E-07	0.000185	**ATP6V1A**	523
ENSG00000183508	1.919301696	7.16E-07	0.000185	**TENT5C**	54855
ENSG00000213903	0.803677067	7.31E-07	0.000185	**LTB4R**	1241
ENSG00000040933	0.739694766	7.56E-07	0.000185	**INPP4A**	3631
ENSG00000122707	1.010877662	7.49E-07	0.000185	**RECK**	8434
ENSG00000087053	-0.616822306	7.94E-07	0.000189	**MTMR2**	8898
ENSG00000135631	0.719561798	8.07E-07	0.000189	**RAB11FIP5**	26056
ENSG00000187098	0.737381193	7.84E-07	0.000189	**MITF**	4286
ENSG00000240849	0.827095897	8.17E-07	0.000189	**TMEM189**	387521
ENSG00000104081	2.036408595	8.38E-07	0.000191	**BMF**	90427
ENSG00000138078	0.773573121	8.51E-07	0.000191	**PREPL**	9581
ENSG00000105662	-0.777600461	9.30E-07	0.000193	**CRTC1**	23373
ENSG00000157259	-0.948265964	9.30E-07	0.000193	**GATAD1**	57798
ENSG00000162384	-0.76436872	9.04E-07	0.000193	**CZIB**	54987
ENSG00000163946	-0.907574228	9.14E-07	0.000193	**TASOR**	23272
ENSG00000166503	-0.954852541	9.27E-07	0.000193	**HDGFL3**	50810
ENSG00000273812	-1.130961211	8.93E-07	0.000193	**NA**	NA
ENSG00000061273	-1.085173654	9.54E-07	0.000193	**HDAC7**	51564
ENSG00000197608	0.889762386	9.57E-07	0.000193	**ZNF841**	284371
ENSG00000143379	-0.764220766	9.76E-07	0.000195	**SETDB1**	9869
ENSG00000060491	-0.560805407	1.05E-06	0.000202	**OGFR**	11054
ENSG00000115993	0.718083717	1.03E-06	0.000202	**TRAK2**	66008
ENSG00000131979	0.728225898	1.06E-06	0.000202	**GCH1**	2643
ENSG00000169193	1.406182516	1.04E-06	0.000202	**CCDC126**	90693
ENSG00000119979	0.704854272	1.10E-06	0.000207	**DENND10**	404636
ENSG00000081181	0.894883633	1.12E-06	0.000208	**ARG2**	384
ENSG00000105245	1.098838406	1.18E-06	0.000217	**NUMBL**	9253
ENSG00000007376	0.635501197	1.29E-06	0.000231	**RPUSD1**	113000
ENSG00000117479	0.656844397	1.28E-06	0.000231	**SLC19A2**	10560
ENSG00000169857	-1.086306661	1.32E-06	0.000234	**AVEN**	57099
ENSG00000169372	1.164509709	1.38E-06	0.000241	**CRADD**	8738
ENSG00000013583	0.668216555	1.41E-06	0.000243	**HEBP1**	50865
ENSG00000188266	2.078196128	1.43E-06	0.000243	**HYKK**	123688
ENSG00000244754	-0.708257314	1.43E-06	0.000243	**N4BP2L2**	10443
ENSG00000137942	1.105590247	1.48E-06	0.000248	**FNBP1L**	54874
ENSG00000067064	0.923720048	1.54E-06	0.000254	**IDI1**	3422
ENSG00000149503	-0.701837975	1.54E-06	0.000254	**INCENP**	3619
ENSG00000179271	-0.46785958	1.57E-06	0.000256	**;ADD45GIP**	90480

ENSG00000077238	-1.128686054	1.62E-06	0.000261	**IL4R**	3566
ENSG00000149257	-1.077384053	1.65E-06	0.000263	**SERPINH1**	871
ENSG00000144580	-0.563017091	1.67E-06	0.000263	**CNOT9**	9125
ENSG00000111665	-0.746665836	1.70E-06	0.000265	**CDCA3**	83461
ENSG00000111231	0.619489717	1.78E-06	0.000266	**GPN3**	51184
ENSG00000130119	-0.786747288	1.73E-06	0.000266	**GNL3L**	54552
ENSG00000141905	-0.94044884	1.76E-06	0.000266	**NFIC**	4782
ENSG00000163517	1.716639511	1.74E-06	0.000266	**HDAC11**	79885
ENSG00000235106	0.788524022	1.77E-06	0.000266	**BRD3OS**	266655
ENSG00000101255	-1.062222822	1.91E-06	0.000274	**TRIB3**	57761
ENSG00000103642	0.617965568	1.91E-06	0.000274	**LACTB**	114294
ENSG00000110429	0.955050087	1.89E-06	0.000274	**FBXO3**	26273
ENSG00000159921	-0.855112943	1.87E-06	0.000274	**GNE**	10020
ENSG00000101384	-0.473216159	1.93E-06	0.000275	**JAG1**	182
ENSG00000111206	-0.74858766	1.97E-06	0.000276	**FOXM1**	2305
ENSG00000162627	-0.566799259	1.97E-06	0.000276	**SNX7**	51375
ENSG00000145431	-0.761253967	2.00E-06	0.000277	**PDGFC**	56034
ENSG00000163536	1.400173032	2.03E-06	0.000279	**SERPINI1**	5274
ENSG00000122140	-0.520306484	2.07E-06	0.000282	**MRPS2**	51116
ENSG00000132906	1.097727738	2.12E-06	0.000287	**CASP9**	842
ENSG00000177733	-0.643534119	2.16E-06	0.00029	**HNRNPA0**	10949
ENSG00000101871	0.87406011	2.24E-06	0.000291	**MID1**	4281
ENSG00000132953	-0.612158857	2.20E-06	0.000291	**XPO4**	64328
ENSG00000163814	-0.892114981	2.22E-06	0.000291	**CDCP1**	64866
ENSG00000184363	-1.14304187	2.23E-06	0.000291	**PKP3**	11187
ENSG00000087903	0.98527979	2.33E-06	0.000295	**RFX2**	5990
ENSG00000137124	0.758842823	2.35E-06	0.000295	**ALDH1B1**	219
ENSG00000148680	-0.901724249	2.33E-06	0.000295	**HTR7**	3363
ENSG00000163249	1.06280995	2.34E-06	0.000295	**CCNYL1**	151195
ENSG00000068971	1.307833681	2.38E-06	0.000295	**PPP2R5B**	5526
ENSG00000170396	-1.582345402	2.39E-06	0.000295	**ZNF804A**	91752
ENSG00000018236	2.048990744	2.51E-06	0.000302	**CNTN1**	1272
ENSG00000142655	-0.891686486	2.52E-06	0.000302	**PEX14**	5195
ENSG00000175305	-1.009452993	2.49E-06	0.000302	**CCNE2**	9134
ENSG00000177181	2.456484847	2.50E-06	0.000302	**RIMKLA**	284716
ENSG00000126461	-0.587836702	2.58E-06	0.000303	**SCAF1**	58506
ENSG00000162419	-0.837236311	2.59E-06	0.000303	**GMEB1**	10691
ENSG00000162437	-1.118437008	2.57E-06	0.000303	**RAVER2**	55225
ENSG00000135823	0.750671892	2.62E-06	0.000304	**STX6**	10228
ENSG00000164970	0.736634068	2.63E-06	0.000304	**FAM219A**	203259
ENSG00000148331	0.768591134	2.67E-06	0.000306	**ASB6**	140459
ENSG00000067900	-0.38177365	2.70E-06	0.000308	**ROCK1**	6093
ENSG00000134755	-1.017963579	2.74E-06	0.00031	**DSC2**	1824
ENSG00000065054	-0.846202187	2.77E-06	0.00031	**SLC9A3R2**	9351
ENSG00000077684	-1.087890789	2.78E-06	0.00031	**JADE1**	79960
ENSG00000179627	1.507650715	2.80E-06	0.00031	**ZBTB42**	1E+08
ENSG00000171109	0.776479955	2.87E-06	0.000316	**MFN1**	55669
ENSG00000165282	-0.608319683	2.92E-06	0.000319	**PIGO**	84720
ENSG00000033627	0.985152893	3.00E-06	0.000321	**ATP6V0A1**	535
ENSG00000070061	0.570576333	3.00E-06	0.000321	**ELP1**	8518

ENSG00000104852	-0.403476103	3.02E-06	0.000321	**SNRNP70**	6625
ENSG00000144036	1.11181583	2.96E-06	0.000321	**EXOC6B**	23233
ENSG00000117152	1.265475463	3.05E-06	0.000323	**RGS4**	5999
ENSG00000065809	0.665694171	3.11E-06	0.000326	**FAM107B**	83641
ENSG00000165480	-0.7847526	3.13E-06	0.000326	**SKA3**	221150
ENSG00000064393	-1.424757143	3.29E-06	0.00033	**HIPK2**	28996
ENSG00000142920	1.293040254	3.29E-06	0.00033	**AZIN2**	113451
ENSG00000143179	-0.710601971	3.23E-06	0.00033	**UCK2**	7371
ENSG00000151353	-1.019219614	3.21E-06	0.00033	**TMEM18**	129787
ENSG00000169991	0.56961406	3.27E-06	0.00033	**IFFO2**	126917
ENSG00000176542	-0.970723721	3.27E-06	0.00033	**USF3**	205717
ENSG00000112699	0.601953008	3.32E-06	0.000331	**GMDS**	2762
ENSG00000157483	0.875637937	3.38E-06	0.000335	**MYO1E**	4643
ENSG00000140465	1.364765588	3.42E-06	0.000337	**CYP1A1**	1543
ENSG00000107551	1.500130758	3.52E-06	0.000345	**RASSF4**	83937
ENSG00000214530	1.060675978	3.55E-06	0.000345	**STARD10**	10809
ENSG00000008256	0.667640777	3.64E-06	0.000346	**CYTH3**	9265
ENSG00000126107	0.809052112	3.62E-06	0.000346	**HECTD3**	79654
ENSG00000189266	-0.729294799	3.58E-06	0.000346	**PNRC2**	55629
ENSG00000280202	-1.682998651	3.65E-06	0.000346	**NA**	NA
ENSG00000135686	0.525085624	3.68E-06	0.000347	**KLHL36**	79786
ENSG00000170581	0.844148979	3.70E-06	0.000347	**STAT2**	6773
ENSG00000075218	-0.755860668	3.75E-06	0.000347	**GTSE1**	51512
ENSG00000076382	-0.81835317	3.86E-06	0.000347	**SPAG5**	10615
ENSG00000079150	1.0292486	4.01E-06	0.000347	**FKBP7**	51661
ENSG00000090861	-0.45697118	3.89E-06	0.000347	**AARS1**	16
ENSG00000104419	1.187470003	3.99E-06	0.000347	**NDRG1**	10397
ENSG00000105699	1.824108159	3.97E-06	0.000347	**LSR**	51599
ENSG00000115392	-0.927979745	3.76E-06	0.000347	**FANCL**	55120
ENSG00000148200	1.300403295	3.80E-06	0.000347	**NR6A1**	2649
ENSG00000151502	0.436992989	3.88E-06	0.000347	**VPS26B**	112936
ENSG00000153982	1.497516134	3.84E-06	0.000347	**GDPD1**	284161
ENSG00000160447	-0.891276969	3.95E-06	0.000347	**PKN3**	29941
ENSG00000172667	0.564712835	3.96E-06	0.000347	**ZMAT3**	64393
ENSG00000180573	0.879842852	3.82E-06	0.000347	**H2AC6**	8334
ENSG00000183814	-1.17326482	4.03E-06	0.000347	**LIN9**	286826
ENSG00000184675	-0.894169471	3.96E-06	0.000347	**AMER1**	139285
ENSG00000115947	0.738926497	4.11E-06	0.000352	**ORC4**	5000
ENSG00000134324	0.666847086	4.13E-06	0.000352	**LPIN1**	23175
ENSG00000107984	0.656877436	4.21E-06	0.000353	**DKK1**	22943
ENSG00000109805	-0.798855692	4.17E-06	0.000353	**NCAPG**	64151
ENSG00000168575	-0.637928565	4.23E-06	0.000353	**SLC20A2**	6575
ENSG00000171824	-1.024305979	4.19E-06	0.000353	**EXOSC10**	5394
ENSG00000078237	0.802780364	4.33E-06	0.000355	**TIGAR**	57103
ENSG00000110852	-1.282542769	4.40E-06	0.000355	**CLEC2B**	9976
ENSG00000119720	-1.151831746	4.40E-06	0.000355	**NRDE2**	55051
ENSG00000136897	-0.532040317	4.39E-06	0.000355	**MRPL50**	54534
ENSG00000185163	-0.678649355	4.37E-06	0.000355	**DDX51**	317781
ENSG00000198492	-0.416833426	4.34E-06	0.000355	**YTHDF2**	51441
ENSG00000231298	-1.126807311	4.36E-06	0.000355	**MANCR**	1E+08

ENSG00000185324	-0.675510213	4.45E-06	0.000357	**CDK10**	8558
ENSG00000143514	0.513021222	4.79E-06	0.000381	**TP53BP2**	7159
ENSG00000172915	1.162113663	4.82E-06	0.000381	**NBEA**	26960
ENSG00000177042	-1.254902045	4.80E-06	0.000381	**TMEM80**	283232
ENSG00000144730	0.888315553	4.93E-06	0.000387	**IL17RD**	54756
ENSG00000113966	1.132491562	5.07E-06	0.000389	**ARL6**	84100
ENSG00000117758	0.914529939	5.10E-06	0.000389	**STX12**	23673
ENSG00000135046	0.639191044	5.04E-06	0.000389	**ANXA1**	301
ENSG00000146063	-0.561573182	5.09E-06	0.000389	**TRIM41**	90933
ENSG00000198612	0.599559825	5.07E-06	0.000389	**COPS8**	10920
ENSG00000205937	-0.384245628	4.99E-06	0.000389	**RNPS1**	10921
ENSG00000158555	1.056581218	5.17E-06	0.000393	**GDPD5**	81544
ENSG00000144560	-1.035550317	5.22E-06	0.000394	**VGLL4**	9686
ENSG00000160310	-0.649737289	5.27E-06	0.000394	**PRMT2**	3275
ENSG00000163935	-1.3358512	5.24E-06	0.000394	**SFMBT1**	51460
ENSG00000198081	-1.312989526	5.28E-06	0.000394	**ZBTB14**	7541
ENSG00000067248	0.589070562	5.45E-06	0.000394	**DHX29**	54505
ENSG00000102007	-0.547785969	5.41E-06	0.000394	**PLP2**	5355
ENSG00000113318	-0.749547189	5.40E-06	0.000394	**MSH3**	4437
ENSG00000140323	0.783934542	5.48E-06	0.000394	**DISP2**	85455
ENSG00000148229	-0.472346795	5.41E-06	0.000394	**POLE3**	54107
ENSG00000150995	0.913892762	5.46E-06	0.000394	**ITPR1**	3708
ENSG00000163435	1.66422038	5.48E-06	0.000394	**ELF3**	1999
ENSG00000175826	-0.538762632	5.43E-06	0.000394	**CTDNEP1**	23399
ENSG00000102699	-1.032026129	5.62E-06	0.000402	**PARP4**	143
ENSG00000136026	-0.830506551	5.66E-06	0.000402	**CKAP4**	10970
ENSG00000143028	2.03713026	5.69E-06	0.000402	**SYPL2**	284612
ENSG00000198952	-0.717345694	5.69E-06	0.000402	**SMG5**	23381
ENSG00000106665	0.96762258	5.78E-06	0.000406	**CLIP2**	7461
ENSG00000095539	2.016162819	5.84E-06	0.000408	**SEMA4G**	57715
ENSG00000079277	0.942808501	5.97E-06	0.000412	**MKNK1**	8569
ENSG00000173020	-0.429670304	5.97E-06	0.000412	**GRK2**	156
ENSG00000198431	0.412710781	5.93E-06	0.000412	**TXNRD1**	7296
ENSG00000104228	0.762100499	6.06E-06	0.000417	**TRIM35**	23087
ENSG00000166851	-1.139597091	6.11E-06	0.000417	**PLK1**	5347
ENSG00000205302	0.464269589	6.12E-06	0.000417	**SNX2**	6643
ENSG00000034713	0.592830458	6.28E-06	0.000422	**GABARAPL**	11345
ENSG00000107771	0.709321103	6.26E-06	0.000422	**CCSER2**	54462
ENSG00000119950	0.996427003	6.23E-06	0.000422	**MXI1**	4601
ENSG00000103326	-0.719265801	6.32E-06	0.000424	**CAPN15**	6650
ENSG00000156642	0.527267317	6.35E-06	0.000424	**NPTN**	27020
ENSG00000134363	-0.989194674	6.41E-06	0.000424	**FST**	10468
ENSG00000141867	-0.5782873	6.41E-06	0.000424	**BRD4**	23476
ENSG00000181222	-0.602009735	6.58E-06	0.000433	**POLR2A**	5430
ENSG00000089057	0.544713921	6.65E-06	0.000434	**SLC23A2**	9962
ENSG00000132005	-1.062227571	6.65E-06	0.000434	**RFX1**	5989
ENSG00000133398	-0.610069398	6.66E-06	0.000434	**MED10**	84246
ENSG00000102554	0.51389558	6.70E-06	0.000434	**KLF5**	688
ENSG00000125266	0.610926877	6.77E-06	0.000437	**EFNB2**	1948
ENSG00000135956	0.606892218	6.84E-06	0.00044	**TMEM127**	55654

ENSG00000121671	1.158067139	6.87E-06	0.00044	**CRY2**	1408
ENSG00000040633	-0.631983205	7.06E-06	0.00044	**PHF23**	79142
ENSG00000111276	-0.782131902	7.04E-06	0.00044	**CDKN1B**	1027
ENSG00000120913	-0.98196438	7.06E-06	0.00044	**PDLIM2**	64236
ENSG00000162923	0.467860445	7.07E-06	0.00044	**WDR26**	80232
ENSG00000168672	0.889923414	7.04E-06	0.00044	**LRATD2**	157638
ENSG00000169126	-1.128853015	6.98E-06	0.00044	**ARMC4**	55130
ENSG00000198189	-0.837058891	6.93E-06	0.00044	**HSD17B11**	51170
ENSG00000236287	-0.642068101	7.10E-06	0.00044	**ZBED5**	58486
ENSG00000071539	-0.747141195	7.36E-06	0.000441	**TRIP13**	9319
ENSG00000072364	0.324991897	7.15E-06	0.000441	**AFF4**	27125
ENSG00000073711	0.959206674	7.44E-06	0.000441	**PPP2R3A**	5523
ENSG00000075275	-0.508125421	7.49E-06	0.000441	**CELSR1**	9620
ENSG00000108587	-0.624461509	7.52E-06	0.000441	**GOSR1**	9527
ENSG00000110756	0.571539804	7.48E-06	0.000441	**HPS5**	11234
ENSG00000115568	-0.641092165	7.51E-06	0.000441	**ZNF142**	7701
ENSG00000132017	-0.73877003	7.37E-06	0.000441	**DCAF15**	90379
ENSG00000132773	-1.312908558	7.24E-06	0.000441	**TOE1**	114034
ENSG00000136213	0.659499564	7.35E-06	0.000441	**CHST12**	55501
ENSG00000137414	1.125827934	7.43E-06	0.000441	**FAM8A1**	51439
ENSG00000168067	0.938890042	7.29E-06	0.000441	**MAP4K2**	5871
ENSG00000173064	0.591217748	7.46E-06	0.000441	**HECTD4**	283450
ENSG00000196391	1.384950986	7.44E-06	0.000441	**ZNF774**	342132
ENSG00000198961	0.396279247	7.18E-06	0.000441	**PJA2**	9867
ENSG00000101191	-0.743430013	7.61E-06	0.000444	**DIDO1**	11083
ENSG00000145495	-0.597670939	7.62E-06	0.000444	**MARCHF6**	10299
ENSG00000012822	1.167799388	8.80E-06	0.000461	**CALCOCO1**	57658
ENSG00000065029	-1.077219496	8.02E-06	0.000461	**ZNF76**	7629
ENSG00000065615	0.690056203	8.09E-06	0.000461	**CYB5R4**	51167
ENSG00000066279	-0.881734603	8.13E-06	0.000461	**ASPM**	259266
ENSG00000073910	1.263019307	8.68E-06	0.000461	**FRY**	10129
ENSG00000083444	0.401934681	8.80E-06	0.000461	**PLOD1**	5351
ENSG00000084070	1.020056753	8.81E-06	0.000461	**SMAP2**	64744
ENSG00000090520	0.637513126	8.45E-06	0.000461	**DNAJB11**	51726
ENSG00000100219	-1.16411667	8.80E-06	0.000461	**XBP1**	7494
ENSG00000101337	-0.791422573	8.01E-06	0.000461	**TM9SF4**	9777
ENSG00000102003	1.867243505	8.25E-06	0.000461	**SYP**	6855
ENSG00000109534	-0.749653265	8.57E-06	0.000461	**GAR1**	54433
ENSG00000123080	-0.756261362	8.69E-06	0.000461	**CDKN2C**	1031
ENSG00000128581	1.020776618	8.26E-06	0.000461	**IFT22**	64792
ENSG00000131941	0.9232878	8.70E-06	0.000461	**RHPN2**	85415
ENSG00000135632	-0.892600786	8.51E-06	0.000461	**SMYD5**	10322
ENSG00000143079	0.711846822	8.07E-06	0.000461	**CTTNBP2NL**	55917
ENSG00000143458	-1.000477321	8.33E-06	0.000461	**GABPB2**	126626
ENSG00000143612	-0.587816345	8.73E-06	0.000461	**C1orf43**	25912
ENSG00000146670	-0.749706565	8.78E-06	0.000461	**CDCA5**	113130
ENSG00000159063	-0.410739866	8.04E-06	0.000461	**ALG8**	79053
ENSG00000161618	-0.738721491	8.64E-06	0.000461	**ALDH16A1**	126133
ENSG00000165240	1.419646279	8.75E-06	0.000461	**ATP7A**	538
ENSG00000166965	-0.918541436	8.81E-06	0.000461	**RCCD1**	91433

ENSG00000169105	-1.031144958	8.17E-06	0.000461	**CHST14**	113189
ENSG00000179967	-0.717204471	8.78E-06	0.000461	**NA**	NA
ENSG00000184226	1.272087009	8.73E-06	0.000461	**PCDH9**	5101
ENSG00000185920	0.894935607	8.40E-06	0.000461	**PTCH1**	5727
ENSG00000198795	-0.932648467	8.74E-06	0.000461	**ZNF521**	25925
ENSG00000215447	0.874993476	8.10E-06	0.000461	**NA**	NA
ENSG00000261221	-0.659406726	8.58E-06	0.000461	**ZNF865**	1.01E+08
ENSG00000066117	-0.542865101	8.89E-06	0.000463	**SMARCD1**	6602
ENSG00000162402	-0.732665304	8.93E-06	0.000463	**USP24**	23358
ENSG00000196584	-0.903790193	8.94E-06	0.000463	**XRCC2**	7516
ENSG00000053372	-0.755516901	8.97E-06	0.000463	**MRTO4**	51154
ENSG00000151025	-1.716917772	9.05E-06	0.000466	**GPR158**	57512
ENSG00000069011	-1.011125433	9.20E-06	0.000469	**PITX1**	5307
ENSG00000116717	0.794665311	9.19E-06	0.000469	**GADD45A**	1647
ENSG00000151806	-0.445468997	9.20E-06	0.000469	**GUF1**	60558
ENSG00000168397	0.680676281	9.23E-06	0.000469	**ATG4B**	23192
ENSG00000070761	0.46373196	9.33E-06	0.00047	**CFAP20**	29105
ENSG00000114933	-1.783643131	9.37E-06	0.00047	**INO80D**	54891
ENSG00000143669	1.115870071	9.31E-06	0.00047	**LYST**	1130
ENSG00000155744	0.903163804	9.35E-06	0.00047	**FAM126B**	285172
ENSG00000120756	0.782579154	9.46E-06	0.000471	**PLS1**	5357
ENSG00000135049	0.613059221	9.47E-06	0.000471	**AGTPBP1**	23287
ENSG00000137288	-0.609533895	9.42E-06	0.000471	**UQCC2**	84300
ENSG00000133138	0.839014907	9.51E-06	0.000471	**TBC1D8B**	54885
ENSG00000007047	1.467774668	9.67E-06	0.000477	**MARK4**	57787
ENSG00000124102	1.636232913	9.68E-06	0.000477	**PI3**	5266
ENSG00000125354	0.823985841	9.73E-06	0.000478	**SEPTIN6**	23157
ENSG00000125388	1.003111699	9.77E-06	0.000478	**GRK4**	2868
ENSG00000243742	2.851378416	9.82E-06	0.000479	**RPLP0P2**	113157
ENSG00000164181	0.978075249	9.93E-06	0.000483	**ELOVL7**	79993
ENSG00000053501	-0.906804202	1.00E-05	0.000486	**USE1**	55850
ENSG00000112208	-1.017217423	1.01E-05	0.00049	**BAG2**	9532
ENSG00000171388	1.111146968	1.02E-05	0.00049	**APLN**	8862
ENSG00000136943	1.329468598	1.02E-05	0.000491	**CTSV**	1515
ENSG00000115935	-0.898159273	1.04E-05	0.000492	**WIPF1**	7456
ENSG00000131584	0.90735264	1.04E-05	0.000492	**ACAP3**	116983
ENSG00000171792	-0.775040189	1.03E-05	0.000492	**RHNO1**	83695
ENSG00000198331	-1.012527014	1.03E-05	0.000492	**HYLS1**	219844
ENSG00000198900	0.474784972	1.04E-05	0.000492	**TOP1**	7150
ENSG00000265972	0.802746987	1.03E-05	0.000492	**TXNIP**	10628
ENSG00000072163	-0.841602085	1.07E-05	0.000496	**LIMS2**	55679
ENSG00000100139	-0.710502083	1.09E-05	0.000496	**MICALL1**	85377
ENSG00000101972	-0.971907826	1.08E-05	0.000496	**STAG2**	10735
ENSG00000105290	1.961551864	1.09E-05	0.000496	**APLP1**	333
ENSG00000113643	0.426688379	1.08E-05	0.000496	**RARS1**	5917
ENSG00000120306	1.033158415	1.08E-05	0.000496	**CYSTM1**	84418
ENSG00000120709	0.760391064	1.07E-05	0.000496	**FAM53C**	51307
ENSG00000120709	0.760391064	1.07E-05	0.000496	**)C1001289**	1E+08
ENSG00000137497	-0.372071152	1.08E-05	0.000496	**NUMA1**	4926
ENSG00000159348	1.055762856	1.08E-05	0.000496	**CYB5R1**	51706

ENSG00000163686	1.057672964	1.08E-05	0.000496	**ABHD6**	57406
ENSG00000170385	0.472833497	1.07E-05	0.000496	**SLC30A1**	7779
ENSG00000172164	-1.187807659	1.07E-05	0.000496	**SNTB1**	6641
ENSG00000175592	-1.077409651	1.06E-05	0.000496	**FOSL1**	8061
ENSG00000124882	0.819868846	1.10E-05	0.0005	**EREG**	2069
ENSG00000175556	-0.897891415	1.11E-05	0.000502	**LONRF3**	79836
ENSG00000063046	-0.680563986	1.12E-05	0.000505	**EIF4B**	1975
ENSG00000070018	0.475751962	1.13E-05	0.000505	**LRP6**	4040
ENSG00000087077	-0.811175545	1.13E-05	0.000505	**TRIP6**	7205
ENSG00000163661	1.015494641	1.13E-05	0.000506	**PTX3**	5806
ENSG00000229689	-1.136265364	1.14E-05	0.00051	**NA**	NA
ENSG00000173950	-0.46399384	1.15E-05	0.000512	**XXYLT1**	152002
ENSG00000181873	-1.367160793	1.16E-05	0.000512	**IBA57**	200205
ENSG00000260565	1.020142672	1.16E-05	0.000512	**NA**	NA
ENSG00000167034	1.112121502	1.17E-05	0.000515	**NKX3-1**	4824
ENSG00000102471	0.580365101	1.19E-05	0.000516	**NDFIP2**	54602
ENSG00000105810	-0.870369257	1.18E-05	0.000516	**CDK6**	1021
ENSG00000107821	-1.06512832	1.18E-05	0.000516	**KAZALD1**	81621
ENSG00000129351	-0.633918734	1.19E-05	0.000516	**ILF3**	3609
ENSG00000133316	-0.865909711	1.18E-05	0.000516	**WDR74**	54663
ENSG00000146674	0.982260447	1.19E-05	0.000516	**IGFBP3**	3486
ENSG00000196839	2.027685739	1.19E-05	0.000516	**ADA**	100
ENSG00000011485	-0.517570838	1.20E-05	0.000517	**PPP5C**	5536
ENSG00000145882	1.141833322	1.20E-05	0.000517	**PCYOX1L**	78991
ENSG00000123983	0.831496597	1.21E-05	0.000521	**ACSL3**	2181
ENSG00000129810	-0.762556644	1.22E-05	0.000521	**SGO1**	151648
ENSG00000265415	-0.739418129	1.22E-05	0.000521	**NA**	NA
ENSG00000276980	1.476749945	1.22E-05	0.000521	**NA**	NA
ENSG00000131051	-1.006655996	1.23E-05	0.000521	**RBM39**	9584
ENSG00000070614	-0.49904755	1.27E-05	0.000529	**NDST1**	3340
ENSG00000125538	0.863090346	1.28E-05	0.000529	**IL1B**	3553
ENSG00000128283	-0.94081155	1.26E-05	0.000529	**CDC42EP1**	11135
ENSG00000142166	-0.728278374	1.25E-05	0.000529	**IFNAR1**	3454
ENSG00000148484	0.588437217	1.27E-05	0.000529	**RSU1**	6251
ENSG00000164951	1.002961928	1.26E-05	0.000529	**PDP1**	54704
ENSG00000168040	-0.708587198	1.28E-05	0.000529	**FADD**	8772
ENSG00000187908	1.538978485	1.27E-05	0.000529	**DMBT1**	1755
ENSG00000198901	-1.015469898	1.27E-05	0.000529	**PRC1**	9055
ENSG00000101940	-0.686266519	1.30E-05	0.000535	**WDR13**	64743
ENSG00000139190	1.211824991	1.30E-05	0.000535	**VAMP1**	6843
ENSG00000166578	1.562756514	1.30E-05	0.000535	**IQCD**	115811
ENSG00000175183	1.297370577	1.31E-05	0.000536	**CSRP2**	1466
ENSG00000145416	-1.250139122	1.33E-05	0.000537	**MARCHF1**	55016
ENSG00000171720	0.782891336	1.32E-05	0.000537	**HDAC3**	8841
ENSG00000181472	-1.599205841	1.33E-05	0.000537	**ZBTB2**	57621
ENSG00000183864	-0.614005692	1.32E-05	0.000537	**TOB2**	10766
ENSG00000187531	0.796069713	1.33E-05	0.000537	**SIRT7**	51547
ENSG00000225439	-2.16663966	1.32E-05	0.000537	**BOLA3-AS1**	1.01E+08
ENSG00000136854	1.147652186	1.34E-05	0.000539	**STXBP1**	6812
ENSG00000149577	0.883261539	1.34E-05	0.000539	**SIDT2**	51092

ENSG00000162614	-1.464624654	1.35E-05	0.000539	**NEXN**	91624
ENSG00000165861	0.789637844	1.35E-05	0.00054	**ZFYVE1**	53349
ENSG00000142197	0.751072181	1.36E-05	0.000541	**DOP1B**	9980
ENSG00000176022	-0.688527956	1.36E-05	0.000541	**B3GALT6**	126792
ENSG00000123609	-1.318979101	1.37E-05	0.000542	**NMI**	9111
ENSG00000141499	0.672631963	1.38E-05	0.000547	**WRAP53**	55135
ENSG00000059804	1.264115871	1.43E-05	0.000563	**SLC2A3**	6515
ENSG00000076356	-0.510570527	1.43E-05	0.000563	**PLXNA2**	5362
ENSG00000156504	0.577634584	1.44E-05	0.000563	**FAM122B**	159090
ENSG00000172889	1.03485975	1.44E-05	0.000563	**EGFL7**	51162
ENSG00000228223	-1.307291102	1.43E-05	0.000563	**HCG11**	493812
ENSG00000137770	-0.492294714	1.45E-05	0.000564	**CTDSPL2**	51496
ENSG00000196743	1.2265076	1.46E-05	0.000566	**GM2A**	2760
ENSG00000132563	1.218638797	1.46E-05	0.000567	**REEP2**	51308
ENSG00000006007	0.70033678	1.47E-05	0.000569	**GDE1**	51573
ENSG00000115758	1.012365014	1.48E-05	0.000569	**ODC1**	4953
ENSG00000130227	-0.57058367	1.48E-05	0.000569	**XPO7**	23039
ENSG00000177675	-0.903137055	1.48E-05	0.00057	**CD163L1**	283316
ENSG00000135119	0.893884129	1.49E-05	0.000572	**RNFT2**	84900
ENSG00000184497	0.804634497	1.49E-05	0.000572	**TMEM255E**	348013
ENSG00000125378	-0.62484373	1.50E-05	0.000573	**BMP4**	652
ENSG00000136159	-0.910000258	1.50E-05	0.000573	**NUDT15**	55270
ENSG00000091039	0.511320143	1.51E-05	0.000574	**OSBPL8**	114882
ENSG00000136783	-0.855226678	1.51E-05	0.000574	**NIPSNAP3A**	25934
ENSG00000140992	0.818512857	1.52E-05	0.000576	**PDPK1**	5170
ENSG00000107798	0.943829647	1.55E-05	0.000586	**LIPA**	3988
ENSG00000132541	0.687347746	1.56E-05	0.000588	**RIDA**	10247
ENSG00000188613	1.336748547	1.57E-05	0.000591	**NANOS1**	340719
ENSG00000099203	-1.280502178	1.59E-05	0.000593	**TMED1**	11018
ENSG00000157456	-0.849443292	1.59E-05	0.000593	**CCNB2**	9133
ENSG00000173727	1.284835894	1.59E-05	0.000593	**NA**	NA
ENSG00000087253	0.908759301	1.62E-05	0.000601	**LPCAT2**	54947
ENSG00000076513	0.636737134	1.64E-05	0.000604	**ANKRD13A**	88455
ENSG00000134955	-1.100291916	1.63E-05	0.000604	**SLC37A2**	219855
ENSG00000173575	-0.916738111	1.64E-05	0.000604	**CHD2**	1106
ENSG00000114383	0.673611402	1.65E-05	0.000607	**TUSC2**	11334
ENSG00000140948	0.580290226	1.66E-05	0.000607	**ZCCHC14**	23174
ENSG00000155252	0.845831062	1.66E-05	0.000607	**PI4K2A**	55361
ENSG00000162441	-0.54540774	1.66E-05	0.000607	**LZIC**	84328
ENSG00000166508	-0.645745852	1.66E-05	0.000607	**MCM7**	4176
ENSG00000205269	1.327630349	1.67E-05	0.000609	**TMEM170E**	1E+08
ENSG00000099250	-1.106787256	1.68E-05	0.00061	**NRP1**	8829
ENSG00000092820	0.408155179	1.71E-05	0.000613	**EZR**	7430
ENSG00000122218	0.276391881	1.70E-05	0.000613	**COPA**	1314
ENSG00000128595	0.330596217	1.70E-05	0.000613	**CALU**	813
ENSG00000138675	-1.122166073	1.70E-05	0.000613	**FGF5**	2250
ENSG00000197355	0.891498714	1.71E-05	0.000613	**UAP1L1**	91373
ENSG00000075223	-0.386843316	1.73E-05	0.00062	**SEMA3C**	10512
ENSG00000124145	1.220455764	1.74E-05	0.00062	**SDC4**	6385
ENSG00000154814	0.592193305	1.74E-05	0.00062	**OXNAD1**	92106

ENSG00000169813	-0.452443545	1.74E-05	0.00062	**HNRNPF**	3185
ENSG00000206417	-1.818482318	1.75E-05	0.000621	**NA**	NA
ENSG00000157240	-0.879373863	1.75E-05	0.000622	**FZD1**	8321
ENSG00000105287	1.023145401	1.81E-05	0.000625	**PRKD2**	25865
ENSG00000105698	-0.575181858	1.78E-05	0.000625	**USF2**	7392
ENSG00000106780	0.892012872	1.81E-05	0.000625	**MEGF9**	1955
ENSG00000116005	0.790214106	1.80E-05	0.000625	**PCYOX1**	51449
ENSG00000124098	0.871513636	1.82E-05	0.000625	**FAM210B**	116151
ENSG00000129038	-0.520835302	1.80E-05	0.000625	**LOXL1**	4016
ENSG00000139618	-1.021277144	1.81E-05	0.000625	**BRCA2**	675
ENSG00000154639	1.572351734	1.80E-05	0.000625	**CXADR**	1525
ENSG00000160785	0.898816539	1.80E-05	0.000625	**SLC25A44**	9673
ENSG00000164054	-0.432143659	1.77E-05	0.000625	**SHISA5**	51246
ENSG00000198960	-0.822416588	1.81E-05	0.000625	**ARMCX6**	54470
ENSG00000213694	0.807226699	1.79E-05	0.000625	**S1PR3**	1903
ENSG00000213694	0.807226699	1.79E-05	0.000625	**C9orf47**	286223
ENSG00000242732	1.314844599	1.82E-05	0.000625	**RTL5**	340526
ENSG00000279207	1.27735399	1.79E-05	0.000625	**NA**	NA
ENSG00000110060	0.930734149	1.84E-05	0.000632	**PUS3**	83480
ENSG00000077420	-1.280659627	1.86E-05	0.000635	**APBB1IP**	54518
ENSG00000135919	0.73490728	1.86E-05	0.000635	**SERPINE2**	5270
ENSG00000160633	-0.739678077	1.86E-05	0.000635	**SAFB**	6294
ENSG00000101216	-1.064552429	1.88E-05	0.000638	**GMEB2**	26205
ENSG00000170185	0.550716404	1.88E-05	0.000638	**USP38**	84640
ENSG00000242125	-0.702802479	1.89E-05	0.000639	**SNHG3**	8420
ENSG00000116199	-0.621948594	1.92E-05	0.000645	**FAM20B**	9917
ENSG00000151876	-1.335646164	1.91E-05	0.000645	**FBXO4**	26272
ENSG00000167693	0.856855042	1.92E-05	0.000645	**NXN**	64359
ENSG00000119048	0.487149635	1.94E-05	0.000652	**UBE2B**	7320
ENSG00000137198	1.443872898	1.95E-05	0.000652	**GMPR**	2766
ENSG00000006451	0.417073703	1.99E-05	0.000656	**RALA**	5898
ENSG00000081154	-0.361116703	1.97E-05	0.000656	**PCNP**	57092
ENSG00000089327	-0.395297013	1.99E-05	0.000656	**FXYD5**	53827
ENSG00000105323	-0.752071209	1.97E-05	0.000656	**HNRNPUL1**	11100
ENSG00000121864	-0.631007854	1.97E-05	0.000656	**ZNF639**	51193
ENSG00000162517	-0.8652869	1.98E-05	0.000656	**PEF1**	553115
ENSG00000171456	-0.668160913	1.99E-05	0.000656	**ASXL1**	171023
ENSG00000183856	-0.623088141	1.97E-05	0.000656	**IQGAP3**	128239
ENSG00000245532	1.37430537	1.99E-05	0.000656	**NEAT1**	283131
ENSG00000081189	1.234310651	2.01E-05	0.00066	**MEF2C**	4208
ENSG00000099282	1.128599893	2.03E-05	0.000662	**TSPAN15**	23555
ENSG00000119771	0.761101837	2.03E-05	0.000662	**KLHL29**	114818
ENSG00000124766	0.552386688	2.02E-05	0.000662	**SOX4**	6659
ENSG00000084110	2.323682897	2.04E-05	0.000663	**HAL**	3034
ENSG00000154813	0.54471407	2.05E-05	0.000665	**DPH3**	285381
ENSG00000008869	0.519527514	2.06E-05	0.000666	**HEATR5B**	54497
ENSG00000112701	-1.293163147	2.07E-05	0.000666	**SENP6**	26054
ENSG00000125835	-0.483800388	2.07E-05	0.000666	**SNRPB**	6628
ENSG00000139182	0.878018181	2.08E-05	0.000666	**CLSTN3**	9746
ENSG00000149480	-0.595204102	2.06E-05	0.000666	**MTA2**	9219

ENSG00000205413	0.69701577	2.07E-05	0.000666	**SAMD9**	54809
ENSG00000136878	1.108486274	2.08E-05	0.000668	**USP20**	10868
ENSG00000071655	-0.871134966	2.10E-05	0.000669	**MBD3**	53615
ENSG00000157227	-1.106604599	2.09E-05	0.000669	**MMP14**	4323
ENSG00000075151	-0.432345849	2.12E-05	0.000672	**EIF4G3**	8672
ENSG00000099814	0.905931262	2.11E-05	0.000672	**CEP170B**	283638
ENSG00000132434	-1.277216679	2.11E-05	0.000672	**LANCL2**	55915
ENSG00000126878	1.425577448	2.14E-05	0.000676	**AIF1L**	83543
ENSG00000144635	-0.746788334	2.16E-05	0.000682	**DYNC1LI1**	51143
ENSG00000183840	-1.093827605	2.17E-05	0.000683	**GPR39**	2863
ENSG00000011304	-0.484338349	2.20E-05	0.000683	**PTBP1**	5725
ENSG00000164220	1.228002547	2.20E-05	0.000683	**F2RL2**	2151
ENSG00000169570	-1.187377355	2.19E-05	0.000683	**DTWD2**	285605
ENSG00000172465	-0.656728464	2.19E-05	0.000683	**TCEAL1**	9338
ENSG00000206053	-0.504082	2.18E-05	0.000683	**JPT2**	90861
ENSG00000214706	-0.632445867	2.20E-05	0.000683	**IFRD2**	7866
ENSG00000251562	0.817989417	2.18E-05	0.000683	**MALAT1**	378938
ENSG00000101782	0.463369284	2.21E-05	0.000687	**RIOK3**	8780
ENSG00000108370	1.47526511	2.23E-05	0.000691	**RGS9**	8787
ENSG00000130779	-0.889792789	2.26E-05	0.000696	**CLIP1**	6249
ENSG00000137693	-1.015908894	2.26E-05	0.000696	**YAP1**	10413
ENSG00000141540	2.159599008	2.26E-05	0.000696	**TTYH2**	94015
ENSG00000161091	0.787621584	2.27E-05	0.000698	**MFSD12**	126321
ENSG00000099364	-0.714003293	2.28E-05	0.000699	**FBXL19**	54620
ENSG00000100364	1.110880482	2.30E-05	0.000699	**KIAA0930**	23313
ENSG00000101194	-0.544144461	2.31E-05	0.000699	**SLC17A9**	63910
ENSG00000110711	-0.570750188	2.30E-05	0.000699	**AIP**	9049
ENSG00000125755	-0.470474053	2.31E-05	0.000699	**SYMPK**	8189
ENSG00000126705	0.852512661	2.29E-05	0.000699	**AHDC1**	27245
ENSG00000177030	-0.857982521	2.31E-05	0.000699	**DEAF1**	10522
ENSG00000253368	1.264382958	2.30E-05	0.000699	**TRNP1**	388610
ENSG00000088812	0.582685829	2.32E-05	0.0007	**ATRN**	8455
ENSG00000011422	-1.011938295	2.33E-05	0.0007	**PLAUR**	5329
ENSG00000076641	1.239546386	2.34E-05	0.0007	**PAG1**	55824
ENSG00000106636	0.465667687	2.34E-05	0.0007	**YKT6**	10652
ENSG00000112984	-0.838397185	2.35E-05	0.0007	**KIF20A**	10112
ENSG00000152242	0.545551919	2.34E-05	0.0007	**C18orf25**	147339
ENSG00000170425	-0.613651115	2.33E-05	0.0007	**ADORA2B**	136
ENSG00000198917	-0.821494009	2.33E-05	0.0007	**SPOUT1**	51490
ENSG00000166046	1.517811356	2.36E-05	0.000702	**TCP11L2**	255394
ENSG00000125386	-0.788162201	2.37E-05	0.000703	**FAM193A**	8603
ENSG00000101224	-0.528595494	2.38E-05	0.000705	**CDC25B**	994
ENSG00000169429	0.992329518	2.39E-05	0.000708	**CXCL8**	3576
ENSG00000111602	-0.812464467	2.40E-05	0.000709	**TIMELESS**	8914
ENSG00000113810	-0.944250636	2.41E-05	0.000709	**SMC4**	10051
ENSG00000178053	1.084318372	2.41E-05	0.000709	**MLF1**	4291
ENSG00000182175	2.979923223	2.43E-05	0.000713	**RGMA**	56963
ENSG00000130590	-1.011949245	2.44E-05	0.000714	**SAMD10**	140700
ENSG00000168488	-0.732052628	2.44E-05	0.000714	**ATXN2L**	11273
ENSG00000189221	1.0248115	2.44E-05	0.000714	**MAOA**	4128

ENSG00000122545	-0.36570656	2.47E-05	0.000718	**SEPTIN7**	989
ENSG00000178726	-1.170159238	2.47E-05	0.000718	**THBD**	7056
ENSG00000127804	-0.532103869	2.48E-05	0.000719	**METTL16**	79066
ENSG00000165424	0.905611791	2.48E-05	0.000719	**ZCCHC24**	219654
ENSG00000167487	0.87394517	2.48E-05	0.000719	**KLHL26**	55295
ENSG00000173221	1.423942866	2.50E-05	0.000723	**GLRX**	2745
ENSG00000061918	1.654585188	2.51E-05	0.000724	**GUCY1B1**	2983
ENSG00000182253	0.457113581	2.52E-05	0.000726	**SYNM**	23336
ENSG00000155008	-0.574395997	2.54E-05	0.000731	**APOOL**	139322
ENSG00000090447	-1.470055382	2.56E-05	0.000734	**TFAP4**	7023
ENSG00000136868	0.770106024	2.56E-05	0.000734	**SLC31A1**	1317
ENSG00000196177	1.175669552	2.58E-05	0.000737	**ACADSB**	36
ENSG00000143418	-0.829036431	2.60E-05	0.000742	**CERS2**	29956
ENSG00000009694	1.803375544	2.61E-05	0.000743	**TENM1**	10178
ENSG00000100523	0.604189908	2.62E-05	0.000746	**DDHD1**	80821
ENSG00000184898	-1.544287815	2.63E-05	0.000747	**RBM43**	375287
ENSG00000125744	1.497404815	2.65E-05	0.000751	**RTN2**	6253
ENSG00000185187	-1.138477901	2.66E-05	0.000752	**SIGIRR**	59307
ENSG00000138678	-0.903531983	2.68E-05	0.000755	**GPAT3**	84803
ENSG00000177383	-0.33736978	2.68E-05	0.000755	**MAGEF1**	64110
ENSG00000038532	1.079946764	2.71E-05	0.00076	**CLEC16A**	23274
ENSG00000121957	0.478526819	2.71E-05	0.00076	**GPSM2**	29899
ENSG00000128050	-0.350796092	2.71E-05	0.00076	**PAICS**	10606
ENSG00000171291	0.727251374	2.72E-05	0.000761	**ZNF439**	90594
ENSG00000197429	-0.888535237	2.73E-05	0.000762	**IPP**	3652
ENSG00000073111	-0.747698646	2.75E-05	0.000766	**MCM2**	4171
ENSG00000116062	-0.559786997	2.76E-05	0.000766	**MSH6**	2956
ENSG00000157216	0.672421843	2.75E-05	0.000766	**SSBP3**	23648
ENSG00000166471	0.532422264	2.76E-05	0.000766	**TMEM41B**	440026
ENSG00000023330	1.080357582	2.78E-05	0.00077	**ALAS1**	211
ENSG00000108312	-1.262796407	2.80E-05	0.000772	**UBTF**	7343
ENSG00000109066	0.828897404	2.80E-05	0.000772	**TMEM104**	54868
ENSG00000186185	-0.975706433	2.81E-05	0.000773	**KIF18B**	146909
ENSG00000121207	1.972627987	2.83E-05	0.000776	**LRAT**	9227
ENSG00000165526	-0.566254464	2.83E-05	0.000776	**RPUSD4**	84881
ENSG00000166128	0.676817745	2.83E-05	0.000776	**RAB8B**	51762
ENSG00000100592	1.214042849	2.85E-05	0.00078	**DAAM1**	23002
ENSG00000105443	-0.661544759	2.86E-05	0.00078	**CYTH2**	9266
ENSG00000136068	0.770781723	2.87E-05	0.00078	**FLNB**	2317
ENSG00000144369	0.869236134	2.86E-05	0.00078	**FAM171B**	165215
ENSG00000135916	0.547843675	2.88E-05	0.000782	**ITM2C**	81618
ENSG00000164308	-1.131678396	2.89E-05	0.000782	**ERAP2**	64167
ENSG00000206538	1.072647897	2.89E-05	0.000782	**VGLL3**	389136
ENSG00000218283	-0.647737236	2.89E-05	0.000782	**NA**	NA
ENSG00000174007	1.444432205	2.93E-05	0.000789	**CEP19**	84984
ENSG00000213024	-0.862362843	2.92E-05	0.000789	**NUP62**	23636
ENSG00000213066	-0.703896361	2.93E-05	0.000789	**FGFR1OP**	11116
ENSG00000089685	-0.674917028	2.94E-05	0.000789	**BIRC5**	332
ENSG00000049239	-0.421151983	2.96E-05	0.000794	**H6PD**	9563
ENSG00000143772	-1.673774513	2.97E-05	0.000794	**ITPKB**	3707

ENSG00000129116	1.283237505	2.98E-05	0.000795	**PALLD**	23022
ENSG00000138772	-0.610340688	2.98E-05	0.000795	**ANXA3**	306
ENSG00000214022	-0.561430239	2.99E-05	0.000797	**REPIN1**	29803
ENSG00000175040	0.577908864	3.01E-05	0.000799	**CHST2**	9435
ENSG00000184292	-0.999887688	3.00E-05	0.000799	**TACSTD2**	4070
ENSG00000100532	0.757850459	3.03E-05	0.000804	**CGRRF1**	10668
ENSG00000266412	0.335285447	3.05E-05	0.000808	**NCOA4**	8031
ENSG00000082898	-1.026530882	3.09E-05	0.000808	**XPO1**	7514
ENSG00000099940	0.70762009	3.10E-05	0.000808	**SNAP29**	9342
ENSG00000108306	0.731203828	3.07E-05	0.000808	**FBXL20**	84961
ENSG00000123064	-0.678375706	3.08E-05	0.000808	**DDX54**	79039
ENSG00000124496	-0.480228341	3.07E-05	0.000808	**TRERF1**	55809
ENSG00000172954	-0.541118826	3.09E-05	0.000808	**LCLAT1**	253558
ENSG00000196396	0.775769828	3.07E-05	0.000808	**PTPN1**	5770
ENSG00000281189	3.117641594	3.08E-05	0.000808	**GHET1**	1.03E+08
ENSG00000104774	0.470748356	3.10E-05	0.000809	**MAN2B1**	4125
ENSG00000229117	-0.326762544	3.12E-05	0.000812	**RPL41**	6171
ENSG00000033030	-0.730257484	3.13E-05	0.000812	**ZCCHC8**	55596
ENSG00000101000	0.686916439	3.13E-05	0.000812	**PROCR**	10544
ENSG00000158769	1.762419877	3.15E-05	0.000814	**F11R**	50848
ENSG00000180801	-1.046536245	3.15E-05	0.000814	**ARSJ**	79642
ENSG00000117519	0.355971443	3.16E-05	0.000815	**CNN3**	1266
ENSG00000198162	0.425184363	3.17E-05	0.000815	**MAN1A2**	10905
ENSG00000271122	1.088085081	3.16E-05	0.000815	**OC1019300**	1.02E+08
ENSG00000152661	-0.7654586	3.18E-05	0.000816	**GJA1**	2697
ENSG00000174705	1.789936502	3.19E-05	0.000817	**SH3PXD2B**	285590
ENSG00000136114	1.33140797	3.21E-05	0.000821	**THSD1**	55901
ENSG00000184900	-0.668276396	3.21E-05	0.000821	**SUMO3**	6612
ENSG00000147316	-0.812919689	3.22E-05	0.000821	**MCPH1**	79648
ENSG00000164754	-0.78808542	3.22E-05	0.000821	**RAD21**	5885
ENSG00000040275	-0.515687	3.23E-05	0.000823	**SPDL1**	54908
ENSG00000038210	-0.620261806	3.27E-05	0.000826	**PI4K2B**	55300
ENSG00000116044	-0.4296263	3.26E-05	0.000826	**NFE2L2**	4780
ENSG00000167978	-0.931196996	3.26E-05	0.000826	**SRRM2**	23524
ENSG00000271643	-1.588807752	3.27E-05	0.000826	**NA**	NA
ENSG00000225733	-0.605407008	3.29E-05	0.000831	**FGD5-AS1**	1.01E+08
ENSG00000112096	0.649642352	3.30E-05	0.000832	**SOD2**	6648
ENSG00000166619	0.415196155	3.31E-05	0.000833	**BLCAP**	10904
ENSG00000182575	2.889895689	3.32E-05	0.000833	**NXPH3**	11248
ENSG00000143952	-0.680286282	3.33E-05	0.000835	**VPS54**	51542
ENSG00000135148	1.088593189	3.34E-05	0.000837	**TRAFD1**	10906
ENSG00000138413	0.755147841	3.35E-05	0.000839	**IDH1**	3417
ENSG00000101412	-0.675675873	3.37E-05	0.00084	**E2F1**	1869
ENSG00000171608	0.867700761	3.38E-05	0.000842	**PIK3CD**	5293
ENSG00000130165	0.909584556	3.40E-05	0.000845	**ELOF1**	84337
ENSG00000167604	-1.481492674	3.40E-05	0.000845	**NFKBID**	84807
ENSG00000116852	2.973058278	3.41E-05	0.000846	**KIF21B**	23046
ENSG00000111321	-0.924169626	3.46E-05	0.000856	**LTBR**	4055
ENSG00000164056	-0.545335837	3.47E-05	0.000856	**SPRY1**	10252
ENSG00000198208	0.936123812	3.46E-05	0.000856	**RPS6KL1**	83694

ENSG00000198176	-0.716959628	3.48E-05	0.000858	**TFDP1**	7027
ENSG00000169129	1.315831747	3.49E-05	0.000859	**AFAP1L2**	84632
ENSG00000114107	0.791811939	3.51E-05	0.000859	**CEP70**	80321
ENSG00000154473	-0.824189191	3.51E-05	0.000859	**BUB3**	9184
ENSG00000166415	-1.32737196	3.50E-05	0.000859	**WDR72**	256764
ENSG00000149679	0.977535883	3.51E-05	0.00086	**CABLES2**	81928
ENSG00000103260	0.913481499	3.52E-05	0.00086	**METRN**	79006
ENSG00000179262	-0.548535198	3.53E-05	0.00086	**RAD23A**	5886
ENSG00000189159	0.880945144	3.53E-05	0.00086	**JPT1**	51155
ENSG00000013375	0.588999559	3.61E-05	0.000861	**PGM3**	5238
ENSG00000054118	-0.363119465	3.62E-05	0.000861	**THRAP3**	9967
ENSG00000081377	0.733519757	3.63E-05	0.000861	**CDC14B**	8555
ENSG00000104897	-0.364812176	3.58E-05	0.000861	**SF3A2**	8175
ENSG00000109320	-1.172192602	3.61E-05	0.000861	**NFKB1**	4790
ENSG00000109685	-0.847565867	3.55E-05	0.000861	**NSD2**	7468
ENSG00000113916	0.796969956	3.62E-05	0.000861	**BCL6**	604
ENSG00000125741	-0.745545261	3.56E-05	0.000861	**OPA3**	80207
ENSG00000126561	-1.275484055	3.59E-05	0.000861	**STAT5A**	6776
ENSG00000138663	0.392941546	3.62E-05	0.000861	**COPS4**	51138
ENSG00000140941	0.828422938	3.60E-05	0.000861	**MAP1LC3B**	81631
ENSG00000151176	0.568763749	3.55E-05	0.000861	**PLBD2**	196463
ENSG00000166938	-0.956041473	3.62E-05	0.000861	**DIS3L**	115752
ENSG00000178209	-0.402662504	3.62E-05	0.000861	**PLEC**	5339
ENSG00000182095	-0.922970243	3.55E-05	0.000861	**TNRC18**	84629
ENSG00000186951	0.980515036	3.59E-05	0.000861	**PPARA**	5465
ENSG00000211445	0.89706939	3.59E-05	0.000861	**GPX3**	2878
ENSG00000135679	-0.656766129	3.66E-05	0.000867	**MDM2**	4193
ENSG00000164323	-0.600893086	3.67E-05	0.000868	**CFAP97**	57587
ENSG00000096070	-1.156625101	3.71E-05	0.000876	**BRPF3**	27154
ENSG00000175061	-0.316003842	3.73E-05	0.000879	**SNHG29**	125144
ENSG00000138166	1.1554969	3.75E-05	0.000882	**DUSP5**	1847
ENSG00000148835	-0.63465283	3.75E-05	0.000882	**TAF5**	6877
ENSG00000160746	0.73943848	3.76E-05	0.000882	**ANO10**	55129
ENSG00000101236	1.088245328	3.77E-05	0.000883	**RNF24**	11237
ENSG00000120549	0.708830099	3.77E-05	0.000883	**KIAA1217**	56243
ENSG00000071537	0.554462258	3.78E-05	0.000883	**SEL1L**	6400
ENSG00000104812	0.540833716	3.79E-05	0.000883	**GYS1**	2997
ENSG00000127528	-1.346763115	3.78E-05	0.000883	**KLF2**	10365
ENSG00000165097	1.089103208	3.79E-05	0.000884	**KDM1B**	221656
ENSG00000167291	1.037034621	3.81E-05	0.000885	**TBC1D16**	125058
ENSG00000047597	-1.095134256	3.83E-05	0.000889	**XK**	7504
ENSG00000024048	0.529142814	3.86E-05	0.000893	**UBR2**	23304
ENSG00000198663	0.6342811	3.86E-05	0.000893	**C6orf89**	221477
ENSG00000163565	-0.534805559	3.88E-05	0.000896	**IFI16**	3428
ENSG00000178718	-0.39379959	3.92E-05	0.000904	**RPP25**	54913
ENSG00000198373	-0.594593832	3.92E-05	0.000904	**WWP2**	11060
ENSG00000081052	1.983843634	3.95E-05	0.000909	**COL4A4**	1286
ENSG00000044574	0.820416814	4.00E-05	0.000916	**HSPA5**	3309
ENSG00000163939	-0.53060509	3.99E-05	0.000916	**PBRM1**	55193
ENSG00000174903	-0.373702395	3.99E-05	0.000916	**RAB1B**	81876

ENSG00000156509	-1.110072363	4.00E-05	0.000917	**FBXO43**	286151
ENSG00000169410	-1.179864738	4.02E-05	0.00092	**PTPN9**	5780
ENSG00000167930	0.68307642	4.03E-05	0.00092	**FAM234A**	83986
ENSG00000158109	0.969825793	4.05E-05	0.000922	**TPRG1L**	127262
ENSG00000101040	-0.865581725	4.05E-05	0.000923	**ZMYND8**	23613
ENSG00000018189	-0.857877611	4.07E-05	0.000925	**RUFY3**	22902
ENSG00000104763	0.630470162	4.08E-05	0.000925	**ASAH1**	427
ENSG00000106330	0.907123432	4.08E-05	0.000925	**MOSPD3**	64598
ENSG00000100395	-0.993029001	4.10E-05	0.000926	**L3MBTL2**	83746
ENSG00000139318	0.754094912	4.11E-05	0.000926	**DUSP6**	1848
ENSG00000143476	-0.460834178	4.11E-05	0.000926	**DTL**	51514
ENSG00000148175	0.743145421	4.10E-05	0.000926	**STOM**	2040
ENSG00000135677	0.486417505	4.15E-05	0.000934	**GNS**	2799
ENSG00000124788	0.672685232	4.17E-05	0.000937	**ATXN1**	6310
ENSG00000143367	1.328771943	4.18E-05	0.000938	**TUFT1**	7286
ENSG00000091436	-0.578556516	4.20E-05	0.000939	**MAP3K20**	51776
ENSG00000137364	1.334055047	4.20E-05	0.000939	**TPMT**	7172
ENSG00000180263	0.736908488	4.19E-05	0.000939	**FGD6**	55785
ENSG00000108582	0.478114892	4.23E-05	0.000945	**CPD**	1362
ENSG00000003402	-1.152029267	4.25E-05	0.000946	**CFLAR**	8837
ENSG00000198218	-0.658940975	4.25E-05	0.000946	**QRICH1**	54870
ENSG00000143228	-0.785755548	4.26E-05	0.000947	**NUF2**	83540
ENSG00000103591	0.530664541	4.27E-05	0.000948	**AAGAB**	79719
ENSG00000247315	-0.915369369	4.30E-05	0.000952	**ZCCHC3**	85364
ENSG00000034152	1.022563292	4.33E-05	0.000954	**MAP2K3**	5606
ENSG00000143507	0.817274125	4.33E-05	0.000954	**DUSP10**	11221
ENSG00000148153	0.643802681	4.31E-05	0.000954	**INIP**	58493
ENSG00000187555	-0.487773082	4.33E-05	0.000954	**USP7**	7874
ENSG00000110917	0.484911432	4.34E-05	0.000954	**MLEC**	9761
ENSG00000172113	-1.023213098	4.34E-05	0.000954	**NME6**	10201
ENSG00000072756	-0.851951178	4.41E-05	0.000956	**TRNT1**	51095
ENSG00000078061	-0.904411688	4.43E-05	0.000956	**ARAF**	369
ENSG00000097007	-0.458382089	4.40E-05	0.000956	**ABL1**	25
ENSG00000101158	-0.551857652	4.42E-05	0.000956	**NELFCD**	51497
ENSG00000114346	-0.678621586	4.42E-05	0.000956	**ECT2**	1894
ENSG00000122783	-0.949594668	4.40E-05	0.000956	**CYREN**	78996
ENSG00000124641	0.878007008	4.37E-05	0.000956	**MED20**	9477
ENSG00000139324	0.514760264	4.39E-05	0.000956	**TMTC3**	160418
ENSG00000143401	-0.800295366	4.40E-05	0.000956	**ANP32E**	81611
ENSG00000151726	0.871522383	4.42E-05	0.000956	**ACSL1**	2180
ENSG00000164091	-0.468547369	4.38E-05	0.000956	**WDR82**	80335
ENSG00000184661	0.387829723	4.42E-05	0.000956	**CDCA2**	157313
ENSG00000256615	2.324829199	4.37E-05	0.000956	**NA**	NA
ENSG00000111247	-0.917484682	4.45E-05	0.00096	**RAD51AP1**	10635
ENSG00000104907	-0.396938523	4.46E-05	0.000961	**TRMT1**	55621
ENSG00000104783	-0.87359161	4.51E-05	0.000962	**KCNN4**	3783
ENSG00000110422	-1.034852378	4.52E-05	0.000962	**HIPK3**	10114
ENSG00000119917	-1.294495512	4.50E-05	0.000962	**IFIT3**	3437
ENSG00000134138	-1.269191227	4.49E-05	0.000962	**MEIS2**	4212
ENSG00000154237	-1.108020805	4.51E-05	0.000962	**LRRK1**	79705

ENSG00000163577	0.821578467	4.48E-05	0.000962	**EIF5A2**	56648
ENSG00000196693	-1.066502756	4.50E-05	0.000962	**ZNF33B**	7582
ENSG00000213339	-0.64429654	4.48E-05	0.000962	**QTRT1**	81890
ENSG00000060642	-0.81102983	4.55E-05	0.000965	**PIGV**	55650
ENSG00000135775	0.604537637	4.54E-05	0.000965	**COG2**	22796
ENSG00000035862	0.450253764	4.56E-05	0.000966	**TIMP2**	7077
ENSG00000110066	-0.760340297	4.57E-05	0.000966	**KMT5B**	51111
ENSG00000129493	0.931728876	4.60E-05	0.000966	**HEATR5A**	25938
ENSG00000135842	-0.504999624	4.59E-05	0.000966	**NIBAN1**	116496
ENSG00000140416	0.387648451	4.59E-05	0.000966	**TPM1**	7168
ENSG00000145041	-0.754121056	4.58E-05	0.000966	**DCAF1**	9730
ENSG00000185909	1.193473579	4.58E-05	0.000966	**KLHDC8B**	200942
ENSG00000188559	1.158974963	4.60E-05	0.000966	**RALGAPA2**	57186
ENSG00000212978	-1.14681289	4.58E-05	0.000966	**LOC339803**	339803
ENSG00000166734	0.676535234	4.61E-05	0.000966	**CASC4**	113201
ENSG00000133818	0.608909943	4.63E-05	0.00097	**RRAS2**	22800
ENSG00000010803	-0.670974598	4.65E-05	0.000971	**SCMH1**	22955
ENSG00000101695	-1.071601632	4.65E-05	0.000971	**RNF125**	54941
ENSG00000006607	0.621791591	4.66E-05	0.000972	**FARP2**	9855
ENSG00000066697	-0.874856493	4.67E-05	0.000972	**MSANTD3**	91283
ENSG00000177508	-1.0726713	4.67E-05	0.000972	**IRX3**	79191
ENSG00000186480	1.073885716	4.70E-05	0.000977	**INSIG1**	3638
ENSG00000125730	0.566207135	4.73E-05	0.000982	**C3**	718
ENSG00000080345	-0.748464848	4.76E-05	0.000986	**RIF1**	55183
ENSG00000108424	-1.064236023	4.78E-05	0.000987	**KPNB1**	3837
ENSG00000109920	-0.55977016	4.79E-05	0.000987	**FNBP4**	23360
ENSG00000111731	-0.760912535	4.78E-05	0.000987	**C2CD5**	9847
ENSG00000154380	0.869340864	4.79E-05	0.000987	**ENAH**	55740
ENSG00000145569	1.991655159	4.83E-05	0.000995	**OTULINL**	54491
ENSG00000114503	-0.344886957	4.85E-05	0.000997	**NCBP2**	22916
ENSG00000180011	-0.983668416	4.86E-05	0.000998	**ZADH2**	284273
ENSG00000140406	-1.123708175	4.90E-05	0.001002	**TLNRD1**	59274
ENSG00000147231	1.160499578	4.91E-05	0.001002	**RADX**	55086
ENSG00000181856	2.342576812	4.91E-05	0.001002	**SLC2A4**	6517
ENSG00000184863	-0.631940262	4.92E-05	0.001002	**RBM33**	155435
ENSG00000197885	0.557921408	4.90E-05	0.001002	**NKIRAS1**	28512
ENSG00000248092	-0.959184068	4.92E-05	0.001002	**NA**	NA
ENSG00000164941	0.49176886	4.94E-05	0.001004	**INTS8**	55656
ENSG00000130066	0.776003805	4.95E-05	0.001006	**SAT1**	6303
ENSG00000093000	-0.900193275	4.98E-05	0.001011	**NUP50**	10762
ENSG00000011426	-0.502633963	5.00E-05	0.001011	**ANLN**	54443
ENSG00000150760	-0.567202541	5.01E-05	0.001011	**DOCK1**	1793
ENSG00000168411	-0.745651194	5.00E-05	0.001011	**RFWD3**	55159
ENSG00000173621	-1.258804556	5.01E-05	0.001011	**LRFN4**	78999
ENSG00000175029	-0.770010249	5.02E-05	0.001012	**CTBP2**	1488
ENSG00000055917	-0.65659488	5.06E-05	0.001012	**PUM2**	23369
ENSG00000076248	-0.841183355	5.03E-05	0.001012	**UNG**	7374
ENSG00000102780	-1.027195201	5.04E-05	0.001012	**DGKH**	160851
ENSG00000103365	0.515741424	5.06E-05	0.001012	**GGA2**	23062
ENSG00000107816	-0.593421193	5.06E-05	0.001012	**LZTS2**	84445

ENSG00000149532	-0.723279149	5.05E-05	0.001012	**CPSF7**	79869
ENSG00000110721	0.564799704	5.07E-05	0.001013	**CHKA**	1119
ENSG00000165527	-0.426491212	5.07E-05	0.001013	**ARF6**	382
ENSG00000168936	-0.441492922	5.08E-05	0.001013	**TMEM129**	92305
ENSG00000116350	-1.068306094	5.11E-05	0.001017	**SRSF4**	6429
ENSG00000197063	-0.451060934	5.15E-05	0.001024	**MAFG**	4097
ENSG00000101966	-0.731515933	5.16E-05	0.001024	**XIAP**	331
ENSG00000143158	0.745615118	5.16E-05	0.001024	**MPC2**	25874
ENSG00000149929	-1.106537965	5.19E-05	0.001028	**HIRIP3**	8479
ENSG00000168917	-0.962202555	5.20E-05	0.001029	**SLC35G2**	80723
ENSG00000171055	-0.542987681	5.22E-05	0.001033	**FEZ2**	9637
ENSG00000089351	0.629375867	5.25E-05	0.001037	**GRAMD1A**	57655
ENSG00000124762	1.111901759	5.27E-05	0.001039	**CDKN1A**	1026
ENSG00000023318	0.589109966	5.32E-05	0.001045	**ERP44**	23071
ENSG00000105676	-0.885467554	5.32E-05	0.001045	**ARMC6**	93436
ENSG00000112033	0.935361041	5.31E-05	0.001045	**PPARD**	5467
ENSG00000178397	0.85568863	5.33E-05	0.001045	**FAM220A**	84792
ENSG00000168175	-0.575813813	5.35E-05	0.001048	**MAPK1IP1I**	93487
ENSG00000015133	-0.385310245	5.40E-05	0.00105	**CCDC88C**	440193
ENSG00000104320	-0.98352866	5.40E-05	0.00105	**NBN**	4683
ENSG00000111144	0.909246235	5.40E-05	0.00105	**LTA4H**	4048
ENSG00000132749	-1.476699234	5.37E-05	0.00105	**TESMIN**	9633
ENSG00000156970	-0.652342546	5.39E-05	0.00105	**BUB1B**	701
ENSG00000173559	1.147945924	5.38E-05	0.00105	**NABP1**	64859
ENSG00000171159	0.713609882	5.41E-05	0.001051	**C9orf16**	79095
ENSG00000164045	-0.982680996	5.42E-05	0.001052	**CDC25A**	993
ENSG00000146247	-0.792723654	5.44E-05	0.001054	**PHIP**	55023
ENSG00000169641	0.617949277	5.46E-05	0.001055	**LUZP1**	7798
ENSG00000182022	-0.548851763	5.46E-05	0.001055	**CHST15**	51363
ENSG00000197905	-0.578268693	5.45E-05	0.001055	**TEAD4**	7004
ENSG00000099797	0.368616754	5.52E-05	0.001064	**TECR**	9524
ENSG00000181458	0.992774854	5.52E-05	0.001064	**TMEM45A**	55076
ENSG00000173230	0.513325928	5.53E-05	0.001065	**GOLGB1**	2804
ENSG00000138801	0.673525571	5.57E-05	0.001068	**PAPSS1**	9061
ENSG00000185900	-1.446272282	5.57E-05	0.001068	**POMK**	84197
ENSG00000196922	0.70501511	5.57E-05	0.001068	**NA**	NA
ENSG00000168032	2.191685824	5.59E-05	0.001071	**ENTPD3**	956
ENSG00000069275	-0.917788379	5.61E-05	0.001073	**NUCKS1**	64710
ENSG00000103335	-0.696753883	5.63E-05	0.001075	**PIEZO1**	9780
ENSG00000022567	1.003271386	5.64E-05	0.001076	**SLC45A4**	57210
ENSG00000085433	1.103699057	5.66E-05	0.001076	**WDR47**	22911
ENSG00000113648	-0.261115321	5.66E-05	0.001076	**IACROH2A**	9555
ENSG00000128908	-0.665555116	5.65E-05	0.001076	**INO80**	54617
ENSG00000121060	0.456012662	5.70E-05	0.001082	**TRIM25**	7706
ENSG00000145390	0.41799117	5.70E-05	0.001082	**USP53**	54532
ENSG00000168393	-0.543655469	5.72E-05	0.001083	**DTYMK**	1841
ENSG00000077514	-0.844944968	5.74E-05	0.001087	**POLD3**	10714
ENSG00000157540	-1.099366077	5.79E-05	0.001093	**DYRK1A**	1859
ENSG00000139746	-0.595100464	5.81E-05	0.001096	**RBM26**	64062
ENSG00000183617	-0.83452934	5.82E-05	0.001096	**MRPL54**	116541

ENSG00000121680	0.733911917	5.82E-05	0.001096	**PEX16**	9409
ENSG00000106462	-0.503483333	5.86E-05	0.001101	**EZH2**	2146
ENSG00000175283	0.546400648	5.86E-05	0.001101	**DOLK**	22845
ENSG00000256663	-1.391333353	5.88E-05	0.001103	**NA**	NA
ENSG00000105974	-0.546710267	5.89E-05	0.001103	**CAV1**	857
ENSG00000153936	0.507281166	5.92E-05	0.001108	**HS2ST1**	9653
ENSG00000168569	-0.747915196	5.93E-05	0.001109	**TMEM223**	79064
ENSG00000049759	1.126068166	5.97E-05	0.001112	**NEDD4L**	23327
ENSG00000100106	-0.81911872	5.96E-05	0.001112	**TRIOBP**	11078
ENSG00000103005	0.849646489	5.97E-05	0.001112	**USB1**	79650
ENSG00000151725	-0.472405243	5.96E-05	0.001112	**CENPU**	79682
ENSG00000167721	-0.571559752	6.00E-05	0.001115	**TSR1**	55720
ENSG00000165476	-1.00048091	6.02E-05	0.001117	**REEP3**	221035
ENSG00000063244	-0.762528507	6.03E-05	0.001118	**U2AF2**	11338
ENSG00000047932	0.489890907	6.05E-05	0.001121	**GOPC**	57120
ENSG00000135414	1.112486335	6.06E-05	0.001121	**GDF11**	10220
ENSG00000111358	-0.413972764	6.07E-05	0.001122	**GTF2H3**	2967
ENSG00000085185	1.092594323	6.10E-05	0.001125	**BCORL1**	63035
ENSG00000117475	0.533599872	6.11E-05	0.001125	**BLZF1**	8548
ENSG00000118407	2.520301084	6.11E-05	0.001125	**FILIP1**	27145
ENSG00000145191	-0.386478365	6.12E-05	0.001125	**EIF2B5**	8893
ENSG00000075643	-0.901992173	6.18E-05	0.001134	**MOCOS**	55034
ENSG00000099785	1.30593522	6.19E-05	0.001134	**MARCHF2**	51257
ENSG00000100281	-0.845957866	6.18E-05	0.001134	**HMGXB4**	10042
ENSG00000169564	-0.753866047	6.19E-05	0.001134	**PCBP1**	5093
ENSG00000065600	0.610521055	6.21E-05	0.001134	**PACC1**	55248
ENSG00000154640	-0.752815494	6.20E-05	0.001134	**BTG3**	10950
ENSG00000152078	0.891275637	6.22E-05	0.001135	**TLCD4**	148534
ENSG00000117862	0.428606074	6.27E-05	0.001141	**TXNDC12**	51060
ENSG00000159267	-0.526250114	6.26E-05	0.001141	**HLCS**	3141
ENSG00000095321	0.929560138	6.28E-05	0.001142	**CRAT**	1384
ENSG00000181481	-0.935405191	6.29E-05	0.001142	**RNF135**	84282
ENSG00000148730	0.564044531	6.30E-05	0.001144	**EIF4EBP2**	1979
ENSG00000183684	-0.480444026	6.32E-05	0.001146	**ALYREF**	10189
ENSG00000197971	-1.262069469	6.34E-05	0.001148	**MBP**	4155
ENSG00000005339	-0.937005357	6.37E-05	0.001149	**CREBBP**	1387
ENSG00000072682	0.984039133	6.39E-05	0.001149	**P4HA2**	8974
ENSG00000112576	0.810884615	6.42E-05	0.001149	**CCND3**	896
ENSG00000114062	-0.598978699	6.41E-05	0.001149	**UBE3A**	7337
ENSG00000126091	-0.583097	6.38E-05	0.001149	**ST3GAL3**	6487
ENSG00000135373	0.952157205	6.42E-05	0.001149	**EHF**	26298
ENSG00000160445	0.789894755	6.41E-05	0.001149	**ZER1**	10444
ENSG00000171817	2.705023785	6.41E-05	0.001149	**ZNF540**	163255
ENSG00000177106	-0.817990459	6.38E-05	0.001149	**EPS8L2**	64787
ENSG00000189241	0.535595375	6.41E-05	0.001149	**TSPYL1**	7259
ENSG00000198113	-0.697915224	6.42E-05	0.001149	**TOR4A**	54863
ENSG00000078142	0.613481315	6.45E-05	0.00115	**PIK3C3**	5289
ENSG00000082497	-1.211917016	6.44E-05	0.00115	**SERTAD4**	56256
ENSG00000099308	1.03264007	6.46E-05	0.00115	**MAST3**	23031
ENSG00000165650	0.369826399	6.46E-05	0.00115	**PDZD8**	118987

ENSG00000130787	1.051844552	6.47E-05	0.00115	**HIP1R**	9026
ENSG00000129484	-1.071356593	6.49E-05	0.001152	**PARP2**	10038
ENSG00000169967	-0.596821548	6.49E-05	0.001152	**MAP3K2**	10746
ENSG00000065328	-0.453264156	6.55E-05	0.001159	**MCM10**	55388
ENSG00000135114	1.492427419	6.54E-05	0.001159	**OASL**	8638
ENSG00000158158	0.709933225	6.55E-05	0.001159	**CNNM4**	26504
ENSG00000115875	-0.727220263	6.61E-05	0.001167	**SRSF7**	6432
ENSG00000006118	0.467951053	6.64E-05	0.001168	**TMEM132A**	54972
ENSG00000140199	0.502080409	6.64E-05	0.001168	**SLC12A6**	9990
ENSG00000165671	-1.196828714	6.64E-05	0.001168	**NSD1**	64324
ENSG00000165689	-0.407406109	6.64E-05	0.001168	**ENTR1**	10807
ENSG00000196470	-1.130969638	6.65E-05	0.001168	**SIAH1**	6477
ENSG00000065802	-0.485503001	6.73E-05	0.001172	**ASB1**	51665
ENSG00000070413	0.514463764	6.72E-05	0.001172	**DGCR2**	9993
ENSG00000101493	0.863260848	6.72E-05	0.001172	**ZNF516**	9658
ENSG00000102096	0.884692932	6.71E-05	0.001172	**PIM2**	11040
ENSG00000102804	0.468169674	6.74E-05	0.001172	**TSC22D1**	8848
ENSG00000116918	0.499389879	6.71E-05	0.001172	**TSNAX**	7257
ENSG00000122299	-0.791293432	6.73E-05	0.001172	**ZC3H7A**	29066
ENSG00000124151	0.645478151	6.74E-05	0.001172	**NCOA3**	8202
ENSG00000167747	-0.67266055	6.73E-05	0.001172	**C19orf48**	84798
ENSG00000182208	0.596844318	6.68E-05	0.001172	**MOB2**	81532
ENSG00000177084	-0.472591373	6.76E-05	0.001174	**POLE**	5426
ENSG00000106608	-0.990356468	6.83E-05	0.001179	**URGCP**	55665
ENSG00000149187	-0.394174756	6.83E-05	0.001179	**CELF1**	10658
ENSG00000149357	-0.581376878	6.83E-05	0.001179	**LAMTOR1**	55004
ENSG00000168802	-0.603470512	6.82E-05	0.001179	**CHTF8**	54921
ENSG00000168802	-0.603470512	6.82E-05	0.001179	**DERPC**	1.13E+08
ENSG00000179051	-1.057746442	6.81E-05	0.001179	**RCC2**	55920
ENSG00000120885	1.09568354	6.87E-05	0.001185	**CLU**	1191
ENSG00000006327	-0.714310637	6.93E-05	0.001189	**TNFRSF12A**	51330
ENSG00000087299	0.600364842	6.91E-05	0.001189	**L2HGDH**	79944
ENSG00000156136	-0.718774123	6.93E-05	0.001189	**DCK**	1633
ENSG00000163683	0.818629565	6.93E-05	0.001189	**SMIM14**	201895
ENSG00000179431	-1.206688725	6.93E-05	0.001189	**FJX1**	24147
ENSG00000148660	0.603651349	6.94E-05	0.001189	**CAMK2G**	818
ENSG00000143924	0.478498677	6.97E-05	0.001191	**EML4**	27436
ENSG00000186340	0.545883695	6.96E-05	0.001191	**THBS2**	7058
ENSG00000024526	-1.06051349	7.04E-05	0.001194	**DEPDC1**	55635
ENSG00000076984	-0.95116328	7.07E-05	0.001194	**MAP2K7**	5609
ENSG00000104691	-1.141426709	7.01E-05	0.001194	**UBXN8**	7993
ENSG00000114554	-0.625952194	7.02E-05	0.001194	**PLXNA1**	5361
ENSG00000116675	1.249974286	7.04E-05	0.001194	**DNAJC6**	9829
ENSG00000130311	-0.396384095	7.09E-05	0.001194	**DDA1**	79016
ENSG00000132424	-0.856041618	7.05E-05	0.001194	**PNISR**	25957
ENSG00000139437	-0.951575762	7.08E-05	0.001194	**TCHP**	84260
ENSG00000161179	-0.595527035	7.07E-05	0.001194	**YDJC**	150223
ENSG00000163516	0.735252486	7.07E-05	0.001194	**ANKZF1**	55139
ENSG00000164620	1.510585122	7.01E-05	0.001194	**RELL2**	285613
ENSG00000165490	0.628947225	7.10E-05	0.001194	**DDIAS**	220042

ENSG00000174516	1.35502863	7.00E-05	0.001194	**PELI3**	246330
ENSG00000205629	0.687068681	7.09E-05	0.001194	**LCMT1**	51451
ENSG00000262879	-0.796644775	7.08E-05	0.001194	**)C1019270**	1.02E+08
ENSG00000105135	-0.602473968	7.11E-05	0.001195	**ILVBL**	10994
ENSG00000087586	-0.997013117	7.13E-05	0.001198	**AURKA**	6790
ENSG00000086475	-0.788594301	7.19E-05	0.001204	**SEPHS1**	22929
ENSG00000162889	0.448270802	7.19E-05	0.001204	**MAPKAPK2**	9261
ENSG00000005175	0.574094979	7.24E-05	0.001207	**RPAP3**	79657
ENSG00000008083	0.614119377	7.21E-05	0.001207	**JARID2**	3720
ENSG00000153291	1.19398657	7.24E-05	0.001207	**SLC25A27**	9481
ENSG00000167685	-0.567694151	7.23E-05	0.001207	**ZNF444**	55311
ENSG00000173272	-0.638468545	7.23E-05	0.001207	**MZT2A**	653784
ENSG00000085998	-0.427733498	7.26E-05	0.001209	**POMGNT1**	55624
ENSG00000112983	-0.814011283	7.30E-05	0.001214	**BRD8**	10902
ENSG00000112715	-0.669482989	7.31E-05	0.001215	**VEGFA**	7422
ENSG00000182903	-0.661851484	7.32E-05	0.001216	**ZNF721**	170960
ENSG00000129292	0.378021546	7.35E-05	0.001217	**PHF20L1**	51105
ENSG00000136448	-0.679257165	7.35E-05	0.001217	**NMT1**	4836
ENSG00000203993	-0.774353438	7.35E-05	0.001217	**\RRDC1-AS**	85026
ENSG00000113621	-0.667252654	7.41E-05	0.001219	**TXNDC15**	79770
ENSG00000116679	-0.597723286	7.42E-05	0.001219	**IVNS1ABP**	10625
ENSG00000129197	0.547765217	7.40E-05	0.001219	**RPAIN**	84268
ENSG00000137203	-0.80752583	7.38E-05	0.001219	**TFAP2A**	7020
ENSG00000140931	-0.953563407	7.39E-05	0.001219	**CMTM3**	123920
ENSG00000145632	0.666835272	7.40E-05	0.001219	**PLK2**	10769
ENSG00000173705	-0.583386556	7.42E-05	0.001219	**SUSD5**	26032
ENSG00000085644	1.058533638	7.45E-05	0.001221	**ZNF213**	7760
ENSG00000196914	0.334825851	7.44E-05	0.001221	**ARHGEF12**	23365
ENSG00000108854	0.421323305	7.47E-05	0.001224	**SMURF2**	64750
ENSG00000198792	0.666580682	7.48E-05	0.001224	**TMEM184E**	25829
ENSG00000139718	-0.956173952	7.51E-05	0.001228	**SETD1B**	23067
ENSG00000114480	-0.511720803	7.52E-05	0.001228	**GBE1**	2632
ENSG00000180667	-0.443515594	7.54E-05	0.00123	**YOD1**	55432
ENSG00000031081	-1.101831984	7.57E-05	0.001234	**ARHGAP31**	57514
ENSG00000103034	1.44565098	7.66E-05	0.001242	**NDRG4**	65009
ENSG00000108826	-0.685627723	7.64E-05	0.001242	**MRPL27**	51264
ENSG00000122335	1.130935048	7.64E-05	0.001242	**SERAC1**	84947
ENSG00000139734	-0.566210838	7.66E-05	0.001242	**DIAPH3**	81624
ENSG00000184216	-0.457334868	7.66E-05	0.001242	**IRAK1**	3654
ENSG00000113649	-0.946450593	7.69E-05	0.001244	**TCERG1**	10915
ENSG00000116017	1.00388745	7.69E-05	0.001244	**ARID3A**	1820
ENSG00000116903	0.491942347	7.73E-05	0.001249	**EXOC8**	149371
ENSG00000020426	0.657493841	7.84E-05	0.001254	**MNAT1**	4331
ENSG00000049449	-0.890203258	7.78E-05	0.001254	**RCN1**	5954
ENSG00000090530	-0.337497854	7.83E-05	0.001254	**P3H2**	55214
ENSG00000101844	0.95576249	7.81E-05	0.001254	**ATG4A**	115201
ENSG00000104738	-0.662855887	7.83E-05	0.001254	**MCM4**	4173
ENSG00000126947	-0.768533608	7.78E-05	0.001254	**ARMCX1**	51309
ENSG00000128591	1.083073319	7.78E-05	0.001254	**FLNC**	2318
ENSG00000140526	0.669840446	7.81E-05	0.001254	**ABHD2**	11057

ENSG00000171016	-1.309706135	7.82E-05	0.001254	**PYGO1**	26108
ENSG00000174136	-0.592771514	7.82E-05	0.001254	**RGMB**	285704
ENSG00000136504	-0.689355598	7.85E-05	0.001255	**KAT7**	11143
ENSG00000138346	-1.047453796	7.88E-05	0.001258	**DNA2**	1763
ENSG00000050405	0.522573847	7.90E-05	0.00126	**LIMA1**	51474
ENSG00000013810	-0.393925801	7.95E-05	0.001261	**TACC3**	10460
ENSG00000110367	-0.937164476	7.95E-05	0.001261	**DDX6**	1656
ENSG00000132646	-0.436436658	7.94E-05	0.001261	**PCNA**	5111
ENSG00000157657	-1.51741296	7.92E-05	0.001261	**ZNF618**	114991
ENSG00000178202	-1.274014062	7.95E-05	0.001261	**POGLUT3**	143888
ENSG00000178821	2.134932844	7.92E-05	0.001261	**TMEM52**	339456
ENSG00000115648	-0.826642143	7.97E-05	0.001263	**MLPH**	79083
ENSG00000175505	-2.25413939	7.98E-05	0.001263	**CLCF1**	23529
ENSG00000080608	-0.732037125	8.00E-05	0.001264	**PUM3**	9933
ENSG00000153187	-0.662356479	8.01E-05	0.001265	**HNRNPU**	3192
ENSG00000117481	-0.913722058	8.04E-05	0.001268	**NSUN4**	387338
ENSG00000213949	0.435469034	8.06E-05	0.00127	**ITGA1**	3672
ENSG00000079156	0.968460608	8.09E-05	0.001271	**OSBPL6**	114880
ENSG00000139278	0.511450273	8.10E-05	0.001271	**GLIPR1**	11010
ENSG00000163346	0.948611447	8.07E-05	0.001271	**PBXIP1**	57326
ENSG00000178996	0.904217855	8.08E-05	0.001271	**SNX18**	112574
ENSG00000120889	0.811659085	8.12E-05	0.001273	**TNFRSF10B**	8795
ENSG00000145391	-0.987931612	8.12E-05	0.001273	**SETD7**	80854
ENSG00000100600	0.877078706	8.14E-05	0.001273	**LGMN**	5641
ENSG00000197142	-0.67673403	8.14E-05	0.001273	**ACSL5**	51703
ENSG00000164251	-0.92878789	8.15E-05	0.001274	**F2RL1**	2150
ENSG00000176871	-0.731282079	8.17E-05	0.001276	**WSB2**	55884
ENSG00000075426	-0.85977553	8.21E-05	0.001278	**FOSL2**	2355
ENSG00000149823	-0.406311098	8.20E-05	0.001278	**VPS51**	738
ENSG00000130584	1.282472678	8.22E-05	0.001279	**ZBTB46**	140685
ENSG00000159202	-0.586808135	8.23E-05	0.00128	**UBE2Z**	65264
ENSG00000148926	0.731995316	8.25E-05	0.001281	**ADM**	133
ENSG00000125656	-0.531165037	8.28E-05	0.001285	**CLPP**	8192
ENSG00000155561	-0.696533437	8.31E-05	0.001287	**NUP205**	23165
ENSG00000169047	-0.564865038	8.31E-05	0.001287	**IRS1**	3667
ENSG00000101126	-1.058576348	8.35E-05	0.001289	**ADNP**	23394
ENSG00000104756	0.424907397	8.38E-05	0.001289	**KCTD9**	54793
ENSG00000105771	-1.0413267	8.39E-05	0.001289	**SMG9**	56006
ENSG00000116133	0.51398152	8.39E-05	0.001289	**DHCR24**	1718
ENSG00000118503	0.756265351	8.38E-05	0.001289	**TNFAIP3**	7128
ENSG00000126216	-1.07855831	8.35E-05	0.001289	**TUBGCP3**	10426
ENSG00000135272	-1.568531228	8.38E-05	0.001289	**MDFIC**	29969
ENSG00000197343	-0.616779083	8.35E-05	0.001289	**ZNF655**	79027
ENSG00000213593	-0.413764212	8.39E-05	0.001289	**TMX2**	51075
ENSG00000166451	-0.534644437	8.41E-05	0.001289	**CENPN**	55839
ENSG00000178607	0.778780122	8.41E-05	0.001289	**ERN1**	2081
ENSG00000135966	-0.595586211	8.42E-05	0.001289	**TGFBRAP1**	9392
ENSG00000096401	-0.490206912	8.45E-05	0.001289	**CDC5L**	988
ENSG00000168496	-0.594328707	8.45E-05	0.001289	**FEN1**	2237
ENSG00000169855	0.5976577	8.44E-05	0.001289	**ROBO1**	6091

ENSG00000188895	-1.209838749	8.45E-05	0.001289	**MSL1**	339287
ENSG00000005889	-1.660463184	8.48E-05	0.001291	**ZFX**	7543
ENSG00000092203	0.563485691	8.48E-05	0.001291	**TOX4**	9878
ENSG00000178252	-0.717199695	8.50E-05	0.001292	**WDR6**	11180
ENSG00000023608	1.004966048	8.52E-05	0.001295	**SNAPC1**	6617
ENSG00000160299	-1.004594209	8.54E-05	0.001297	**PCNT**	5116
ENSG00000112312	-0.404735364	8.56E-05	0.001298	**GMNN**	51053
ENSG00000163125	-1.34394627	8.57E-05	0.001298	**RPRD2**	23248
ENSG00000153130	0.518029947	8.58E-05	0.001299	**SCOC**	60592
ENSG00000138172	-0.996610521	8.60E-05	0.001301	**CALHM2**	51063
ENSG00000091140	0.280884465	8.63E-05	0.001301	**DLD**	1738
ENSG00000101361	-0.508125323	8.62E-05	0.001301	**NOP56**	10528
ENSG00000116266	-0.535351886	8.62E-05	0.001301	**STXBP3**	6814
ENSG00000123136	-0.51509163	8.65E-05	0.001302	**DDX39A**	10212
ENSG00000165458	-0.655830193	8.67E-05	0.001304	**INPPL1**	3636
ENSG00000174032	0.767514941	8.67E-05	0.001304	**SLC25A30**	253512
ENSG00000187147	-0.503218252	8.68E-05	0.001304	**RNF220**	55182
ENSG00000070540	0.644453553	8.71E-05	0.001305	**WIPI1**	55062
ENSG00000147162	-0.667064636	8.71E-05	0.001305	**OGT**	8473
ENSG00000150347	0.913853694	8.71E-05	0.001305	**ARID5B**	84159
ENSG00000108828	0.508717555	8.75E-05	0.001306	**VAT1**	10493
ENSG00000116128	0.593668137	8.73E-05	0.001306	**BCL9**	607
ENSG00000138061	-0.966255913	8.76E-05	0.001306	**CYP1B1**	1545
ENSG00000165355	0.99028559	8.77E-05	0.001306	**FBXO33**	254170
ENSG00000169136	-1.362417239	8.74E-05	0.001306	**ATF5**	22809
ENSG00000173208	5.366954311	8.77E-05	0.001306	**ABCD2**	225
ENSG00000078177	-1.037594864	8.78E-05	0.001306	**N4BP2**	55728
ENSG00000113594	1.029543447	8.84E-05	0.001315	**LIFR**	3977
ENSG00000164332	0.509257311	8.89E-05	0.00132	**UBLCP1**	134510
ENSG00000119878	0.830545521	8.91E-05	0.00132	**CRIPT**	9419
ENSG00000134852	-0.693265822	8.91E-05	0.00132	**CLOCK**	9575
ENSG00000148719	-1.00676764	8.91E-05	0.00132	**DNAJB12**	54788
ENSG00000177311	-0.779499058	8.93E-05	0.001322	**ZBTB38**	253461
ENSG00000144840	-1.22507127	8.95E-05	0.001323	**RABL3**	285282
ENSG00000070814	-0.39815676	8.98E-05	0.001325	**TCOF1**	6949
ENSG00000092531	0.646922141	8.97E-05	0.001325	**SNAP23**	8773
ENSG00000128294	0.842067477	8.99E-05	0.001325	**TPST2**	8459
ENSG00000148082	-0.569306327	9.00E-05	0.001325	**SHC3**	53358
ENSG00000122952	-0.643749664	9.04E-05	0.001328	**ZWINT**	11130
ENSG00000136827	-0.505871782	9.05E-05	0.001328	**TOR1A**	1861
ENSG00000185236	-0.497261963	9.04E-05	0.001328	**RAB11B**	9230
ENSG00000263465	-0.854679239	9.02E-05	0.001328	**SRSF8**	10929
ENSG00000111145	-0.87601259	9.10E-05	0.00133	**ELK3**	2004
ENSG00000119673	-0.791254933	9.08E-05	0.00133	**ACOT2**	10965
ENSG00000123144	-0.417010841	9.10E-05	0.00133	**TRIR**	79002
ENSG00000166974	0.819218619	9.09E-05	0.00133	**MAPRE2**	10982
ENSG00000166821	1.065242711	9.13E-05	0.001334	**PEX11A**	8800
ENSG00000135451	-0.678388377	9.17E-05	0.001334	**TROAP**	10024
ENSG00000161021	-0.349128241	9.16E-05	0.001334	**MAML1**	9794
ENSG00000164743	1.357112362	9.15E-05	0.001334	**C8orf48**	157773

ENSG00000189079	-1.09609284	9.15E-05	0.001334	**ARID2**	196528
ENSG00000042286	-0.781727728	9.20E-05	0.001338	**AIFM2**	84883
ENSG00000117676	0.737866823	9.23E-05	0.001339	**RPS6KA1**	6195
ENSG00000130856	-0.990809129	9.22E-05	0.001339	**ZNF236**	7776
ENSG00000105983	0.610945979	9.30E-05	0.001345	**LMBR1**	64327
ENSG00000145990	0.748035598	9.29E-05	0.001345	**GFOD1**	54438
ENSG00000167880	-0.925357158	9.27E-05	0.001345	**EVPL**	2125
ENSG00000169714	-0.676034667	9.29E-05	0.001345	**CNBP**	7555
ENSG00000130816	-0.632757144	9.34E-05	0.001346	**DNMT1**	1786
ENSG00000138614	-0.683867854	9.33E-05	0.001346	**INTS14**	81556
ENSG00000157193	-0.891540667	9.33E-05	0.001346	**LRP8**	7804
ENSG00000130725	-0.43437838	9.36E-05	0.001348	**UBE2M**	9040
ENSG00000198689	0.881914395	9.37E-05	0.001349	**SLC9A6**	10479
ENSG00000130826	-0.601932733	9.45E-05	0.001358	**DKC1**	1736
ENSG00000134769	0.63984661	9.46E-05	0.001358	**DTNA**	1837
ENSG00000184887	-0.620995303	9.46E-05	0.001358	**BTBD6**	90135
ENSG00000108821	1.403198095	9.49E-05	0.001359	**COL1A1**	1277
ENSG00000116285	0.730007538	9.49E-05	0.001359	**ERRFI1**	54206
ENSG00000117528	0.793501909	9.48E-05	0.001359	**ABCD3**	5825
ENSG00000138750	-0.332799085	9.51E-05	0.00136	**NUP54**	53371
ENSG00000105373	-0.492385724	9.53E-05	0.001362	**NOP53**	29997
ENSG00000148297	-0.547380155	9.54E-05	0.001362	**MED22**	6837
ENSG00000162337	-0.959184322	9.54E-05	0.001362	**LRP5**	4041
ENSG00000127837	0.437200451	9.58E-05	0.001365	**AAMP**	14
ENSG00000078269	-0.869540238	9.60E-05	0.001365	**SYNJ2**	8871
ENSG00000087301	0.803291957	9.59E-05	0.001365	**TXNDC16**	57544
ENSG00000109606	-0.589196038	9.60E-05	0.001365	**DHX15**	1665
ENSG00000126457	-0.323683147	9.62E-05	0.001366	**PRMT1**	3276
ENSG00000139697	0.351581092	9.64E-05	0.001368	**SBNO1**	55206
ENSG00000115363	-0.939407848	9.68E-05	0.001372	**EVA1A**	84141
ENSG00000136813	-0.92660282	9.73E-05	0.001379	**ECPAS**	23392
ENSG00000154133	-1.338294276	9.76E-05	0.001381	**ROBO4**	54538
ENSG00000144354	-1.15558111	9.77E-05	0.001382	**CDCA7**	83879
ENSG00000157796	0.922940213	9.80E-05	0.001384	**WDR19**	57728
ENSG00000169116	1.954269908	9.81E-05	0.001384	**PARM1**	25849
ENSG00000072071	0.934257091	9.91E-05	0.001387	**ADGRL1**	22859
ENSG00000100385	2.945381	9.94E-05	0.001387	**IL2RB**	3560
ENSG00000111540	0.345934167	9.85E-05	0.001387	**RAB5B**	5869
ENSG00000125901	-0.562311832	9.90E-05	0.001387	**MRPS26**	64949
ENSG00000128159	0.556012171	9.88E-05	0.001387	**TUBGCP6**	85378
ENSG00000131165	0.341384977	9.88E-05	0.001387	**CHMP1A**	5119
ENSG00000143157	-0.945200865	9.86E-05	0.001387	**POGK**	57645
ENSG00000145088	2.103487131	9.85E-05	0.001387	**EAF2**	55840
ENSG00000149218	0.806801212	9.90E-05	0.001387	**ENDOD1**	23052
ENSG00000152642	0.59711343	9.89E-05	0.001387	**GPD1L**	23171
ENSG00000163904	0.429644241	9.93E-05	0.001387	**SENP2**	59343
ENSG00000169902	0.73400873	9.92E-05	0.001387	**TPST1**	8460
ENSG00000182957	0.736541934	9.93E-05	0.001387	**SPATA13**	221178
ENSG00000186660	-0.991032796	9.96E-05	0.001388	**ZFP91**	80829
ENSG00000106537	1.612684087	9.99E-05	0.001389	**TSPAN13**	27075

ENSG00000116874	-1.193765555	9.99E-05	0.001389	**WARS2**	10352
ENSG00000141452	0.855919112	9.97E-05	0.001389	**RMC1**	29919
ENSG00000164284	-0.619538733	9.98E-05	0.001389	**GRPEL2**	134266
ENSG00000114268	0.865426022	0.0001	0.001389	**PFKFB4**	5210
ENSG00000181938	-1.425032291	0.0001	0.001389	**GINS3**	64785
ENSG00000239264	-0.707459372	0.0001	0.001389	**TXNDC5**	81567
ENSG00000129055	-0.475767507	0.0001	0.001389	**ANAPC13**	25847
ENSG00000137193	0.41908507	0.000101	0.001391	**PIM1**	5292
ENSG00000162869	0.640476957	0.000101	0.001391	**PPP1R21**	129285
ENSG00000066651	-0.721640395	0.000101	0.001396	**TRMT11**	60487
ENSG00000116985	1.627417143	0.000101	0.001396	**BMP8B**	656
ENSG00000176490	4.033548517	0.000101	0.001396	**DIRAS1**	148252
ENSG00000198467	-0.943760093	0.000101	0.001396	**TPM2**	7169
ENSG00000102109	1.214942154	0.000101	0.001397	**PCSK1N**	27344
ENSG00000095787	-0.709431371	0.000102	0.001399	**WAC**	51322
ENSG00000176422	-0.940490623	0.000102	0.001399	**SPRYD4**	283377
ENSG00000237187	-0.871007951	0.000102	0.001399	**NR2F1-AS1**	441094
ENSG00000266094	0.76756697	0.000102	0.001399	**RASSF5**	83593
ENSG00000141002	-0.748178631	0.000103	0.001407	**TCF25**	22980
ENSG00000266074	-0.834651694	0.000103	0.001409	**BAHCC1**	57597
ENSG00000167619	3.127329374	0.000103	0.001411	**TMEM145**	284339
ENSG00000272933	-1.606494016	0.000103	0.001411	**NA**	NA
ENSG00000110660	-1.0752146	0.000103	0.001413	**SLC35F2**	54733
ENSG00000164294	-0.933356137	0.000104	0.001415	**GPX8**	493869
ENSG00000136010	3.60423255	0.000104	0.001416	**ALDH1L2**	160428
ENSG00000198171	-0.61605072	0.000104	0.001416	**DDRGK1**	65992
ENSG00000168066	-0.276297084	0.000104	0.001418	**SF1**	7536
ENSG00000163453	-0.425677834	0.000104	0.001419	**IGFBP7**	3490
ENSG00000227375	2.924196918	0.000104	0.001419	**DLG1-AS1**	1.01E+08
ENSG00000100297	-0.783882334	0.000105	0.001424	**MCM5**	4174
ENSG00000138738	-1.510683651	0.000105	0.001424	**PRDM5**	11107
ENSG00000008294	0.525355791	0.000105	0.001429	**SPAG9**	9043
ENSG00000156860	-0.39311161	0.000106	0.00143	**FBRS**	64319
ENSG00000008513	0.513234133	0.000106	0.001436	**ST3GAL1**	6482
ENSG00000178921	-0.580718952	0.000106	0.001438	**PFAS**	5198
ENSG00000105127	-0.987753702	0.000106	0.001438	**AKAP8**	10270
ENSG00000135387	-0.520801754	0.000107	0.00144	**CAPRIN1**	4076
ENSG00000184371	-0.519155527	0.000107	0.00144	**CSF1**	1435
ENSG00000110092	-1.101419073	0.000108	0.001446	**CCND1**	595
ENSG00000111674	0.865965973	0.000107	0.001446	**ENO2**	2026
ENSG00000168724	0.380148782	0.000107	0.001446	**DNAJC21**	134218
ENSG00000099326	-1.218174257	0.000108	0.001446	**MZF1**	7593
ENSG00000100034	-0.557421778	0.000108	0.001446	**PPM1F**	9647
ENSG00000119729	0.635409784	0.000108	0.001446	**RHOQ**	23433
ENSG00000124226	0.646113725	0.000108	0.001446	**RNF114**	55905
ENSG00000127946	0.799308131	0.000108	0.001446	**HIP1**	3092
ENSG00000151422	-0.498743591	0.000108	0.001446	**FER**	2241
ENSG00000155506	-0.925865151	0.000108	0.001446	**LARP1**	23367
ENSG00000280435	0.5417226	0.000108	0.001446	**NA**	NA
ENSG00000184009	0.684024117	0.000109	0.001452	**ACTG1**	71

ENSG00000112697	0.402680131	0.000109	0.001453	**TMEM30A**	55754
ENSG00000155827	0.455164631	0.000109	0.001454	**RNF20**	56254
ENSG00000114270	-0.396024019	0.000109	0.001456	**COL7A1**	1294
ENSG00000041982	0.78967183	0.00011	0.001457	**TNC**	3371
ENSG00000167191	1.007390416	0.00011	0.001457	**GPRC5B**	51704
ENSG00000172613	0.771194073	0.00011	0.001457	**RAD9A**	5883
ENSG00000171475	0.426716426	0.00011	0.001457	**WIPF2**	147179
ENSG00000183779	0.60516875	0.00011	0.001458	**ZNF703**	80139
ENSG00000169554	-1.036444861	0.00011	0.001462	**ZEB2**	9839
ENSG00000136193	-0.781515112	0.000111	0.001463	**SCRN1**	9805
ENSG00000136450	-0.688785043	0.000111	0.001463	**SRSF1**	6426
ENSG00000157578	2.868771175	0.000111	0.001463	**LCA5L**	150082
ENSG00000159131	-0.570853696	0.000111	0.001463	**GART**	2618
ENSG00000161642	1.572233236	0.000111	0.001463	**ZNF385A**	25946
ENSG00000136205	-0.807433892	0.000111	0.001468	**TNS3**	64759
ENSG00000187193	1.47690482	0.000111	0.001468	**MT1X**	4501
ENSG00000058668	1.318044665	0.000112	0.001469	**ATP2B4**	493
ENSG00000179218	0.468082589	0.000112	0.001469	**CALR**	811
ENSG00000063438	-0.926248228	0.000112	0.001472	**NA**	NA
ENSG00000120539	-0.653958217	0.000112	0.001472	**MASTL**	84930
ENSG00000164609	0.604838206	0.000112	0.001472	**SLU7**	10569
ENSG00000148516	-0.727608368	0.000113	0.00148	**ZEB1**	6935
ENSG00000079819	0.426278819	0.000113	0.001481	**EPB41L2**	2037
ENSG00000084774	-0.570702123	0.000113	0.001481	**CAD**	790
ENSG00000234616	-0.949667542	0.000113	0.001481	**JRK**	8629
ENSG00000274523	-0.744401357	0.000113	0.001481	**RCC1L**	81554
ENSG00000166250	-1.078498289	0.000114	0.001482	**CLMP**	79827
ENSG00000069122	-0.588238983	0.000114	0.001486	**ADGRF5**	221395
ENSG00000112118	-0.591865769	0.000114	0.001486	**MCM3**	4172
ENSG00000147394	0.408643221	0.000114	0.001488	**ZNF185**	7739
ENSG00000167513	-0.918608425	0.000114	0.001488	**CDT1**	81620
ENSG00000176890	-0.871768643	0.000114	0.001488	**TYMS**	7298
ENSG00000164976	1.449690035	0.000115	0.001492	**MYORG**	57462
ENSG00000154310	0.950835929	0.000115	0.001494	**TNIK**	23043
ENSG00000147676	2.48532253	0.000116	0.0015	**MAL2**	114569
ENSG00000068697	0.423825676	0.000116	0.001502	**LAPTM4A**	9741
ENSG00000119414	0.33915915	0.000116	0.001502	**PPP6C**	5537
ENSG00000182180	-0.513480687	0.000116	0.001502	**MRPS16**	51021
ENSG00000204977	-0.700383361	0.000116	0.001502	**TRIM13**	10206
ENSG00000141458	0.643510304	0.000116	0.001502	**NPC1**	4864
ENSG00000149485	-0.58872319	0.000116	0.001502	**FADS1**	3992
ENSG00000136100	-1.115225131	0.000117	0.001506	**VPS36**	51028
ENSG00000100941	-0.493546582	0.000117	0.001506	**PNN**	5411
ENSG00000108175	-0.359499535	0.000117	0.001507	**ZMIZ1**	57178
ENSG00000127947	-0.761205099	0.000117	0.00151	**PTPN12**	5782
ENSG00000062598	0.873714254	0.000118	0.001511	**ELMO2**	63916
ENSG00000072062	0.71068512	0.000118	0.001511	**PRKACA**	5566
ENSG00000104064	-0.765654037	0.000118	0.001515	**GABPB1**	2553
ENSG00000169258	0.959435178	0.000118	0.001519	**GPRIN1**	114787
ENSG00000100596	0.341017739	0.000119	0.00152	**SPTLC2**	9517

ENSG00000150782	0.629194045	0.000119	0.00152	**IL18**	3606
ENSG00000173137	0.693997204	0.000119	0.00152	**ADCK5**	203054
ENSG00000151923	-0.528623728	0.000119	0.001524	**TIAL1**	7073
ENSG00000167552	0.922618946	0.000119	0.001524	**TUBA1A**	7846
ENSG00000105568	-0.381536193	0.00012	0.001527	**PPP2R1A**	5518
ENSG00000175727	0.459384096	0.00012	0.001532	**MLXIP**	22877
ENSG00000083223	0.473437748	0.000121	0.001533	**TUT7**	79670
ENSG00000111371	-0.701719398	0.00012	0.001533	**SLC38A1**	81539
ENSG00000126804	-1.231866508	0.00012	0.001533	**ZBTB1**	22890
ENSG00000221968	0.653013864	0.000121	0.001533	**FADS3**	3995
ENSG00000080839	-0.768297718	0.000121	0.001535	**RBL1**	5933
ENSG00000099901	-0.469898728	0.000121	0.001535	**RANBP1**	5902
ENSG00000163214	0.603129043	0.000121	0.001538	**DHX57**	90957
ENSG00000198890	-0.734449529	0.000122	0.001548	**PRMT6**	55170
ENSG00000119318	-0.421784979	0.000123	0.001557	**RAD23B**	5887
ENSG00000143756	-0.329414301	0.000123	0.001557	**FBXO28**	23219
ENSG00000122547	1.653581791	0.000123	0.001557	**EEPD1**	80820
ENSG00000141569	-0.793647182	0.000124	0.00156	**TRIM65**	201292
ENSG00000137309	-0.307580033	0.000124	0.001566	**HMGA1**	3159
ENSG00000155265	2.894540679	0.000124	0.001566	**GOLGA7B**	401647
ENSG00000107957	-0.800283741	0.000124	0.001567	**SH3PXD2A**	9644
ENSG00000122550	0.912461229	0.000124	0.001567	**KLHL7**	55975
ENSG00000068903	0.625627782	0.000125	0.001571	**SIRT2**	22933
ENSG00000116685	0.434370121	0.000126	0.001578	**KIAA2013**	90231
ENSG00000139880	0.657614913	0.000126	0.001578	**CDH24**	64403
ENSG00000185745	1.15071808	0.000126	0.001578	**IFIT1**	3434
ENSG00000096746	-0.550349562	0.000126	0.001578	**HNRNPH3**	3189
ENSG00000145781	-0.573550874	0.000126	0.00158	**COMMD10**	51397
ENSG00000105854	-1.323404838	0.000126	0.001583	**PON2**	5445
ENSG00000090975	-0.909790834	0.000127	0.001588	**PITPNM2**	57605
ENSG00000083799	0.484756936	0.000127	0.001588	**CYLD**	1540
ENSG00000138668	-0.557492396	0.000127	0.001588	**HNRNPD**	3184
ENSG00000174791	-0.728561108	0.000127	0.001588	**RIN1**	9610
ENSG00000013563	0.839001078	0.000128	0.001591	**DNASE1L1**	1774
ENSG00000100441	-0.402766702	0.000128	0.001591	**KHNYN**	23351
ENSG00000136051	0.522147313	0.000128	0.001594	**WASHC4**	23325
ENSG00000108061	0.737151965	0.000128	0.001595	**SHOC2**	8036
ENSG00000013441	-0.824613038	0.000129	0.001597	**CLK1**	1195
ENSG00000064703	-0.491952563	0.000128	0.001597	**DDX20**	11218
ENSG00000089094	-0.679390239	0.000129	0.001598	**KDM2B**	84678
ENSG00000004897	-0.582401946	0.000129	0.0016	**CDC27**	996
ENSG00000087087	-0.429384947	0.000129	0.0016	**SRRT**	51593
ENSG00000122641	2.106077698	0.000129	0.0016	**INHBA**	3624
ENSG00000163808	-0.537713531	0.00013	0.0016	**KIF15**	56992
ENSG00000196535	0.569309765	0.000129	0.0016	**MYO18A**	399687
ENSG00000198839	0.992133119	0.000129	0.0016	**ZNF277**	11179
ENSG00000136052	1.035635054	0.00013	0.001609	**SLC41A2**	84102
ENSG00000103647	-0.66380598	0.000131	0.001615	**CORO2B**	10391
ENSG00000142733	-0.925951046	0.000131	0.001615	**MAP3K6**	9064
ENSG00000103502	0.577570383	0.000131	0.001617	**CDIPT**	10423

ENSG00000142784	0.709947163	0.000131	0.001617	**WDTC1**	23038
ENSG00000145901	-0.599916372	0.000132	0.001617	**TNIP1**	10318
ENSG00000175931	0.723486962	0.000131	0.001617	**UBE2O**	63893
ENSG00000146648	-0.650049888	0.000132	0.001618	**EGFR**	1956
ENSG00000189223	-0.573075099	0.000132	0.001619	**PAX8-AS1**	654433
ENSG00000105726	-0.364256635	0.000132	0.00162	**ATP13A1**	57130
ENSG00000216775	0.880900339	0.000132	0.00162	**LOC730101**	730101
ENSG00000101945	-1.334177954	0.000132	0.00162	**SUV39H1**	6839
ENSG00000163297	-0.655716229	0.000133	0.001622	**ANTXR2**	118429
ENSG00000008086	1.327471003	0.000133	0.001626	**CDKL5**	6792
ENSG00000115163	-0.89087216	0.000133	0.001626	**CENPA**	1058
ENSG00000197808	1.412857395	0.000133	0.001626	**ZNF461**	92283
ENSG00000100201	-0.873506033	0.000134	0.001629	**DDX17**	10521
ENSG00000170571	-0.662027262	0.000134	0.00163	**EMB**	133418
ENSG00000117408	0.936794688	0.000134	0.001633	**IPO13**	9670
ENSG00000160193	-1.046826508	0.000134	0.001634	**WDR4**	10785
ENSG00000061936	-0.492363264	0.000135	0.001641	**SFSWAP**	6433
ENSG00000137261	1.887856782	0.000135	0.001645	**KIAA0319**	9856
ENSG00000130638	-0.354322941	0.000136	0.001646	**ATXN10**	25814
ENSG00000126351	0.687603132	0.000136	0.001648	**THRA**	7067
ENSG00000155254	-1.070369733	0.000136	0.001648	**MARVELD1**	83742
ENSG00000163291	0.566060174	0.000136	0.001648	**PAQR3**	152559
ENSG00000165512	-0.578040061	0.000136	0.00165	**ZNF22**	7570
ENSG00000169490	1.030524126	0.000137	0.001653	**TM2D2**	83877
ENSG00000006634	-0.636473354	0.000137	0.001654	**DBF4**	10926
ENSG00000134824	-0.388653566	0.000137	0.001654	**FADS2**	9415
ENSG00000144029	-0.463784562	0.000137	0.001654	**MRPS5**	64969
ENSG00000058056	-1.240725353	0.000138	0.001658	**USP13**	8975
ENSG00000128342	-1.281276488	0.000138	0.001658	**LIF**	3976
ENSG00000169252	0.629905313	0.000138	0.001658	**ADRB2**	154
ENSG00000010310	2.378890904	0.000138	0.001662	**GIPR**	2696
ENSG00000101310	0.4095066	0.000138	0.001664	**SEC23B**	10483
ENSG00000254858	0.882869562	0.000139	0.001672	**MPV17L2**	84769
ENSG00000087502	-0.915065992	0.00014	0.001674	**ERGIC2**	51290
ENSG00000167522	-0.479774177	0.00014	0.001674	**ANKRD11**	29123
ENSG00000110046	0.898124818	0.00014	0.001674	**ATG2A**	23130
ENSG00000122966	-0.599295027	0.00014	0.00168	**CIT**	11113
ENSG00000165102	-0.81067807	0.000141	0.001683	**HGSNAT**	138050
ENSG00000196428	0.82985938	0.000141	0.001683	**TSC22D2**	9819
ENSG00000150687	-0.456773537	0.000141	0.001684	**PRSS23**	11098
ENSG00000044090	-0.581481961	0.000142	0.00169	**CUL7**	9820
ENSG00000057019	0.314462128	0.000142	0.001696	**DCBLD2**	131566
ENSG00000102317	-0.537301002	0.000142	0.001696	**RBM3**	5935
ENSG00000114805	0.971811959	0.000143	0.001696	**PLCH1**	23007
ENSG00000124615	0.524897774	0.000142	0.001696	**MOCS1**	4337
ENSG00000164307	-0.416171294	0.000142	0.001696	**ERAP1**	51752
ENSG00000198252	0.539218863	0.000143	0.001696	**STYX**	6815
ENSG00000197265	0.495018822	0.000143	0.001698	**GTF2E2**	2961
ENSG00000146223	-0.728678925	0.000143	0.001699	**RPL7L1**	285855
ENSG00000148773	-0.789405744	0.000143	0.001699	**MKI67**	4288

ENSG00000159231	-1.749399357	0.000143	0.001699	**CBR3**	874
ENSG00000143294	-0.373910959	0.000144	0.0017	**PRCC**	5546
ENSG00000120733	-0.55280254	0.000144	0.001702	**KDM3B**	51780
ENSG00000196235	0.408528599	0.000144	0.001702	**SUPT5H**	6829
ENSG00000149639	-0.864678577	0.000144	0.001705	**SOGA1**	140710
ENSG00000136861	-0.718612711	0.000145	0.001707	**CDK5RAP2**	55755
ENSG00000188529	-0.517858559	0.000145	0.001707	**SRSF10**	10772
ENSG00000047249	0.630907385	0.000145	0.001709	**ATP6V1H**	51606
ENSG00000125845	0.965686015	0.000145	0.001709	**BMP2**	650
ENSG00000175063	-0.67082802	0.000145	0.001709	**UBE2C**	11065
ENSG00000168038	1.39258339	0.000146	0.001715	**ULK4**	54986
ENSG00000233237	0.442965692	0.000146	0.001715	**LINC00472**	79940
ENSG00000106366	0.824793881	0.000146	0.001718	**SERPINE1**	5054
ENSG00000130038	-1.266866073	0.000147	0.00172	**CRACR2A**	84766
ENSG00000139546	-0.733024562	0.000147	0.00172	**TARBP2**	6895
ENSG00000080986	-0.992273183	0.000147	0.001721	**NDC80**	10403
ENSG00000085491	-0.76502769	0.000147	0.001721	**SLC25A24**	29957
ENSG00000113742	1.047081983	0.000147	0.001721	**CPEB4**	80315
ENSG00000114541	1.136115339	0.000147	0.001721	**FRMD4B**	23150
ENSG00000115255	1.736393217	0.000147	0.001721	**REEP6**	92840
ENSG00000174485	0.590180909	0.000147	0.001721	**DENND4A**	10260
ENSG00000185760	-1.219922489	0.000147	0.001721	**KCNQ5**	56479
ENSG00000035403	0.460873398	0.000148	0.001726	**VCL**	7414
ENSG00000048707	0.549707805	0.000149	0.001726	**VPS13D**	55187
ENSG00000065559	0.411445508	0.000149	0.001726	**MAP2K4**	6416
ENSG00000103187	0.377147727	0.000149	0.001726	**COTL1**	23406
ENSG00000105339	1.106219588	0.000149	0.001726	**DENND3**	22898
ENSG00000111885	0.661705515	0.000149	0.001726	**MAN1A1**	4121
ENSG00000124006	-0.627148815	0.000149	0.001726	**OBSL1**	23363
ENSG00000137947	0.830241498	0.000148	0.001726	**GTF2B**	2959
ENSG00000152527	1.293971094	0.000148	0.001726	**PLEKHH2**	130271
ENSG00000198830	-0.559707575	0.000149	0.001726	**HMGN2**	3151
ENSG00000184743	-0.755863321	0.00015	0.001741	**ATL3**	25923
ENSG00000123636	-0.801049784	0.000151	0.001742	**BAZ2B**	29994
ENSG00000147459	-0.771165111	0.000151	0.001742	**DOCK5**	80005
ENSG00000151651	-0.554022341	0.000151	0.001742	**ADAM8**	101
ENSG00000154822	-0.814484687	0.000151	0.001742	**PLCL2**	23228
ENSG00000161847	-0.763235987	0.000151	0.001742	**RAVER1**	125950
ENSG00000165175	-0.899686948	0.000151	0.001742	**MID1IP1**	58526
ENSG00000004838	3.255217239	0.000152	0.001745	**ZMYND10**	51364
ENSG00000114739	-1.188100131	0.000152	0.001747	**ACVR2B**	93
ENSG00000132688	1.068735774	0.000152	0.001748	**NES**	10763
ENSG00000154305	0.513451188	0.000152	0.001748	**MIA3**	375056
ENSG00000166233	0.362739427	0.000152	0.001748	**ARIH1**	25820
ENSG00000076053	-0.472894407	0.000152	0.001749	**RBM7**	10179
ENSG00000120616	-1.183864025	0.000153	0.00175	**EPC1**	80314
ENSG00000009844	0.389256929	0.000153	0.00175	**VTA1**	51534
ENSG00000130830	1.328910004	0.000153	0.001751	**MPP1**	4354
ENSG00000100811	-0.46920184	0.000153	0.001751	**YY1**	7528
ENSG00000167107	0.968911681	0.000153	0.001751	**ACSF2**	80221

ENSG00000101109	0.577700929	0.000154	0.001756	**STK4**	6789
ENSG00000162521	-0.494485754	0.000154	0.001764	**RBBP4**	5928
ENSG00000130702	-0.391106195	0.000155	0.001768	**LAMA5**	3911
ENSG00000100603	-0.550340099	0.000155	0.00177	**SNW1**	22938
ENSG00000156011	0.521665211	0.000155	0.00177	**PSD3**	23362
ENSG00000085662	0.504210841	0.000156	0.001779	**AKR1B1**	231
ENSG00000197070	0.631677017	0.000156	0.00178	**ARRDC1**	92714
ENSG00000114316	-0.516362669	0.000157	0.001787	**USP4**	7375
ENSG00000114316	-0.516362669	0.000157	0.001787	**OC10798608**	1.08E+08
ENSG00000148343	0.754981095	0.000157	0.001787	**MIGA2**	84895
ENSG00000198513	1.483476514	0.000157	0.001787	**ATL1**	51062
ENSG00000110344	0.558284297	0.000158	0.001797	**UBE4A**	9354
ENSG00000124795	-0.665593962	0.000159	0.001797	**DEK**	7913
ENSG00000072501	-0.604253178	0.000159	0.0018	**SMC1A**	8243
ENSG00000129173	-1.357743637	0.000159	0.001803	**E2F8**	79733
ENSG00000221890	1.00615176	0.00016	0.001808	**NPTXR**	23467
ENSG00000101298	1.422019522	0.000161	0.001809	**SNPH**	9751
ENSG00000106477	-0.710079755	0.000161	0.001809	**CEP41**	95681
ENSG00000126464	-0.941564551	0.000161	0.001809	**PRR12**	57479
ENSG00000149177	-0.995626619	0.00016	0.001809	**PTPRJ**	5795
ENSG00000151718	0.422623169	0.000161	0.001809	**WWC2**	80014
ENSG00000171681	-0.631245316	0.000161	0.001809	**ATF7IP**	55729
ENSG00000174738	-0.517657192	0.00016	0.001809	**NR1D2**	9975
ENSG00000226950	-0.581535712	0.000161	0.001809	**DANCR**	57291
ENSG00000242265	0.39547123	0.000161	0.001809	**PEG10**	23089
ENSG00000265808	0.427865521	0.00016	0.001809	**SEC22B**	9554
ENSG00000071054	-0.7286595	0.000162	0.001814	**MAP4K4**	9448
ENSG00000152503	0.637077076	0.000162	0.001816	**TRIM36**	55521
ENSG00000090372	-0.576067333	0.000162	0.001816	**STRN4**	29888
ENSG00000179833	0.380767554	0.000162	0.001816	**SERTAD2**	9792
ENSG00000167642	-0.363933874	0.000163	0.001824	**SPINT2**	10653
ENSG00000100744	1.145990033	0.000164	0.001833	**GSKIP**	51527
ENSG00000135045	-0.775658459	0.000164	0.001833	**C9orf40**	55071
ENSG00000137812	-0.560264362	0.000164	0.001833	**KNL1**	57082
ENSG00000168404	-0.817387058	0.000164	0.001833	**MLKL**	197259
ENSG00000159873	-0.484273214	0.000165	0.001835	**CCDC117**	150275
ENSG00000163659	0.720434931	0.000165	0.001835	**TIPARP**	25976
ENSG00000181513	0.975802188	0.000165	0.001839	**ACBD4**	79777
ENSG00000013573	-0.707126125	0.000166	0.001843	**DDX11**	1663
ENSG00000198121	-0.911152359	0.000166	0.001843	**LPAR1**	1902
ENSG00000055130	-1.235660476	0.000167	0.001848	**CUL1**	8454
ENSG00000092148	-0.465284788	0.000167	0.001848	**HECTD1**	25831
ENSG00000108395	0.350460079	0.000167	0.001848	**TRIM37**	4591
ENSG00000108813	2.524216988	0.000167	0.001848	**DLX4**	1748
ENSG00000118007	-1.121829038	0.000167	0.001848	**STAG1**	10274
ENSG00000123104	0.493829802	0.000167	0.001848	**ITPR2**	3709
ENSG00000124942	-0.782167426	0.000167	0.001848	**AHNAK**	79026
ENSG00000135678	1.023800057	0.000167	0.001848	**CPM**	1368
ENSG00000138604	0.796303806	0.000167	0.001848	**GLCE**	26035
ENSG00000152784	-1.383627341	0.000167	0.001848	**PRDM8**	56978

ENSG00000104472	-0.911785394	0.000168	0.00185	**CHRAC1**	54108
ENSG00000134802	-0.802623831	0.000168	0.001851	**SLC43A3**	29015
ENSG00000100403	-0.757022197	0.000168	0.001853	**ZC3H7B**	23264
ENSG00000119655	0.534553923	0.000168	0.001853	**NPC2**	10577
ENSG00000182795	2.184754707	0.000169	0.001859	**C1orf116**	79098
ENSG00000097033	-0.43550855	0.000169	0.001864	**SH3GLB1**	51100
ENSG00000119669	-0.901137382	0.00017	0.001864	**IRF2BPL**	64207
ENSG00000105248	-1.068522261	0.00017	0.001866	**YJU2**	55702
ENSG00000117226	-0.838057172	0.00017	0.001866	**GBP3**	2635
ENSG00000163528	-0.891857908	0.00017	0.001866	**CHCHD4**	131474
ENSG00000277443	-0.358051539	0.00017	0.001866	**MARCKS**	4082
ENSG00000120699	-0.772316057	0.00017	0.001866	**EXOSC8**	11340
ENSG00000198324	0.890175522	0.000171	0.001874	**PHETA1**	144717
ENSG00000138942	0.679243764	0.000171	0.001874	**RNF185**	91445
ENSG00000140859	-0.608651781	0.000171	0.001874	**KIFC3**	3801
ENSG00000156050	0.876706369	0.000172	0.001876	**FAM161B**	145483
ENSG00000087338	0.63128901	0.000172	0.001878	**GMCL1**	64395
ENSG00000085511	-0.471743518	0.000173	0.001884	**MAP3K4**	4216
ENSG00000162642	0.627746581	0.000173	0.001884	**C1orf52**	148423
ENSG00000137776	-0.690561437	0.000173	0.001884	**SLTM**	79811
ENSG00000113013	-0.386506919	0.000173	0.001884	**HSPA9**	3313
ENSG00000115109	1.045245449	0.000173	0.001884	**EPB41L5**	57669
ENSG00000155975	0.52376596	0.000173	0.001884	**VPS37A**	137492
ENSG00000095066	1.162032006	0.000174	0.001887	**HOOK2**	29911
ENSG00000111328	-0.407943164	0.000174	0.001887	**CDK2AP1**	8099
ENSG00000102034	-1.011338209	0.000174	0.001887	**ELF4**	2000
ENSG00000164163	-0.717142322	0.000174	0.001889	**ABCE1**	6059
ENSG00000109576	-0.762182191	0.000175	0.001895	**AADAT**	51166
ENSG00000160211	0.856172175	0.000175	0.001897	**G6PD**	2539
ENSG00000162909	-0.413441165	0.000175	0.001897	**CAPN2**	824
ENSG00000163510	-0.633615942	0.000176	0.001903	**CWC22**	57703
ENSG00000189091	-0.52092941	0.000176	0.001905	**SF3B3**	23450
ENSG00000082258	-0.605967843	0.000177	0.001912	**CCNT2**	905
ENSG00000136824	-0.593338324	0.000177	0.001912	**SMC2**	10592
ENSG00000184575	-0.651768389	0.000177	0.001912	**XPOT**	11260
ENSG00000103978	-0.86302136	0.000178	0.001916	**TMEM87A**	25963
ENSG00000136932	-0.779639408	0.000178	0.001916	**TRMO**	51531
ENSG00000118508	-0.584943121	0.000179	0.00192	**RAB32**	10981
ENSG00000143878	0.892529608	0.000179	0.00192	**RHOB**	388
ENSG00000160551	-0.50578501	0.000179	0.00192	**TAOK1**	57551
ENSG00000162607	-0.8678923	0.000179	0.00192	**USP1**	7398
ENSG00000196843	1.099296933	0.000178	0.00192	**ARID5A**	10865
ENSG00000071794	0.441160588	0.00018	0.001922	**HLTF**	6596
ENSG00000073350	1.99411998	0.00018	0.001922	**LLGL2**	3993
ENSG00000124374	1.784306442	0.00018	0.001922	**PAIP2B**	400961
ENSG00000124813	-1.023133702	0.00018	0.001922	**RUNX2**	860
ENSG00000130827	0.981408049	0.000179	0.001922	**PLXNA3**	55558
ENSG00000132669	-0.861444302	0.00018	0.001922	**RIN2**	54453
ENSG00000140262	-0.667762557	0.00018	0.001922	**TCF12**	6938
ENSG00000085741	3.136502642	0.00018	0.001923	**WNT11**	7481

ENSG00000213551	-0.504269922	0.00018	0.001925	**DNAJC9**	23234
ENSG00000101187	-1.1884925	0.000181	0.001926	**SLCO4A1**	28231
ENSG00000111676	-1.101229911	0.000181	0.001927	**ATN1**	1822
ENSG00000074657	-0.963821696	0.000182	0.001938	**ZNF532**	55205
ENSG00000116016	1.220005933	0.000182	0.001939	**EPAS1**	2034
ENSG00000166016	0.918265036	0.000183	0.001942	**ABTB2**	25841
ENSG00000185697	0.517842726	0.000183	0.001945	**MYBL1**	4603
ENSG00000143850	-1.068091699	0.000183	0.001946	**PLEKHA6**	22874
ENSG00000092201	-0.547299196	0.000184	0.001949	**SUPT16H**	11198
ENSG00000112992	-0.510782014	0.000184	0.001949	**NNT**	23530
ENSG00000135316	-0.531345328	0.000184	0.001949	**SYNCRIP**	10492
ENSG00000162650	-0.999824555	0.000184	0.001949	**ATXN7L2**	127002
ENSG00000186063	-0.408789591	0.000184	0.001949	**AIDA**	64853
ENSG00000186501	-0.60244217	0.000184	0.001949	**TMEM222**	84065
ENSG00000198963	1.638141834	0.000184	0.001949	**RORB**	6096
ENSG00000172893	0.377512942	0.000185	0.00195	**DHCR7**	1717
ENSG00000268001	-1.92363903	0.000185	0.00195	**CARD8-AS1**	1.01E+08
ENSG00000115963	0.875009237	0.000185	0.00195	**RND3**	390
ENSG00000133961	0.398892388	0.000185	0.001952	**NUMB**	8650
ENSG00000136111	-0.700198271	0.000185	0.001952	**TBC1D4**	9882
ENSG00000101773	-0.621363348	0.000186	0.001954	**RBBP8**	5932
ENSG00000114423	0.895110588	0.000186	0.001954	**CBLB**	868
ENSG00000079387	-0.761461315	0.000186	0.001958	**SENP1**	29843
ENSG00000177706	-1.230829416	0.000186	0.001958	**FAM20C**	56975
ENSG00000074855	0.610416129	0.000187	0.001959	**ANO8**	57719
ENSG00000134690	-0.888310354	0.000187	0.001959	**CDCA8**	55143
ENSG00000129932	-0.51235064	0.000187	0.001961	**DOHH**	83475
ENSG00000163131	-0.952533414	0.000187	0.001961	**CTSS**	1520
ENSG00000115339	-0.978587726	0.000187	0.001962	**GALNT3**	2591
ENSG00000142227	-0.807735334	0.000188	0.001963	**EMP3**	2014
ENSG00000127533	-1.976456345	0.000188	0.001966	**F2RL3**	9002
ENSG00000156931	0.524880241	0.000188	0.001966	**VPS8**	23355
ENSG00000108840	0.877545124	0.000188	0.001968	**HDAC5**	10014
ENSG00000010810	0.666202085	0.000189	0.00197	**FYN**	2534
ENSG00000156976	-0.428274326	0.000189	0.00197	**EIF4A2**	1974
ENSG00000149260	1.30374062	0.000189	0.001974	**CAPN5**	726
ENSG00000106789	1.491861323	0.00019	0.001975	**CORO2A**	7464
ENSG00000140396	-1.26833129	0.00019	0.001978	**NCOA2**	10499
ENSG00000101350	0.506577832	0.00019	0.001978	**KIF3B**	9371
ENSG00000147274	-0.784973772	0.00019	0.001978	**RBMX**	27316
ENSG00000091127	-0.544726071	0.000191	0.001979	**PUS7**	54517
ENSG00000142910	1.042066977	0.000191	0.001979	**TINAGL1**	64129
ENSG00000061676	-0.443761558	0.000192	0.001988	**NCKAP1**	10787
ENSG00000169607	-0.490356913	0.000192	0.001991	**CKAP2L**	150468
ENSG00000176619	-0.49362083	0.000192	0.001991	**LMNB2**	84823
ENSG00000130935	-0.476431192	0.000193	0.001994	**NOL11**	25926
ENSG00000117523	-0.400759197	0.000193	0.001998	**PRRC2C**	23215
ENSG00000159140	-0.54802707	0.000193	0.001998	**SON**	6651
ENSG00000198538	0.915206031	0.000194	0.002001	**ZNF28**	7576
ENSG00000106852	2.108110388	0.000195	0.002012	**LHX6**	26468

ENSG00000182134	0.875484526	0.000195	0.002014	**TDRKH**	11022
ENSG00000003436	-1.099432954	0.000196	0.002015	**TFPI**	7035
ENSG00000132155	-0.515361812	0.000196	0.002015	**RAF1**	5894
ENSG00000143126	1.571696645	0.000196	0.002015	**CELSR2**	1952
ENSG00000152056	0.852766189	0.000196	0.002015	**AP1S3**	130340
ENSG00000163026	0.613157758	0.000196	0.002015	**WDCP**	80304
ENSG00000174238	0.506830948	0.000196	0.002015	**PITPNA**	5306
ENSG00000176014	-0.66568674	0.000196	0.002015	**TUBB6**	84617
ENSG00000168769	-0.672158417	0.000196	0.002018	**TET2**	54790
ENSG00000130164	0.417631626	0.000197	0.00202	**LDLR**	3949
ENSG00000167861	1.689596446	0.000198	0.002034	**HID1**	283987
ENSG00000101311	-0.928344129	0.000199	0.002041	**FERMT1**	55612
ENSG00000115419	0.531328657	0.0002	0.002044	**GLS**	2744
ENSG00000173706	-0.705372966	0.0002	0.002044	**HEG1**	57493
ENSG00000177283	-0.950059729	0.0002	0.002044	**FZD8**	8325
ENSG00000196642	-0.507362564	0.0002	0.002044	**RABL6**	55684
ENSG00000196950	-0.837574659	0.0002	0.002044	**SLC39A10**	57181
ENSG00000167695	-1.161019518	0.0002	0.002047	**TLCD3A**	79850
ENSG00000011105	0.646078593	0.000201	0.00205	**TSPAN9**	10867
ENSG00000087448	-1.196685457	0.000201	0.002054	**KLHL42**	57542
ENSG00000110090	0.664097273	0.000202	0.002054	**CPT1A**	1374
ENSG00000183718	0.746377368	0.000202	0.002054	**TRIM52**	84851
ENSG00000204899	-0.491475691	0.000202	0.002054	**MZT1**	440145
ENSG00000151470	0.776649116	0.000202	0.002056	**C4orf33**	132321
ENSG00000179409	-0.628636065	0.000203	0.002059	**GEMIN4**	50628
ENSG00000182199	-0.353520464	0.000203	0.00206	**SHMT2**	6472
ENSG00000166801	-0.928573116	0.000204	0.002067	**FAM111A**	63901
ENSG00000033867	0.321800893	0.000204	0.002068	**SLC4A7**	9497
ENSG00000132716	-0.690909781	0.000204	0.002069	**DCAF8**	50717
ENSG00000162542	-0.752341113	0.000204	0.00207	**TMCO4**	255104
ENSG00000137804	-0.483134558	0.000204	0.002071	**NUSAP1**	51203
ENSG00000003393	0.651150152	0.000205	0.002073	**ALS2**	57679
ENSG00000165732	-0.460883918	0.000205	0.002075	**DDX21**	9188
ENSG00000072195	0.99582691	0.000205	0.002075	**SPEG**	10290
ENSG00000113758	-0.404823546	0.000206	0.002075	**DBN1**	1627
ENSG00000137494	0.8946125	0.000206	0.002075	**ANKRD42**	338699
ENSG00000140450	1.140129609	0.000206	0.002075	**ARRDC4**	91947
ENSG00000177685	0.96755059	0.000206	0.002075	**CRACR2B**	283229
ENSG00000183963	-0.634842317	0.000206	0.002075	**SMTN**	6525
ENSG00000015475	-0.752512973	0.000206	0.002077	**BID**	637
ENSG00000128203	1.516716828	0.000206	0.002077	**ASPHD2**	57168
ENSG00000100554	0.515471528	0.000207	0.002083	**ATP6V1D**	51382
ENSG00000144677	-0.814295242	0.000208	0.002091	**CTDSPL**	10217
ENSG00000171132	-0.470193911	0.000208	0.002093	**PRKCE**	5581
ENSG00000186300	1.191626411	0.000208	0.002093	**ZNF555**	148254
ENSG00000143344	1.515470689	0.000209	0.002097	**RGL1**	23179
ENSG00000198853	-0.479980802	0.000209	0.002097	**RUSC2**	9853
ENSG00000143321	-0.677558227	0.00021	0.0021	**HDGF**	3068
ENSG00000129295	1.412306063	0.00021	0.002105	**LRRC6**	23639
ENSG00000147419	0.394550392	0.00021	0.002105	**CCDC25**	55246

ENSG00000112245	-0.342546549	0.000211	0.002113	**PTP4A1**	7803
ENSG00000177666	0.749710541	0.000211	0.002113	**PNPLA2**	57104
ENSG00000172375	0.6612581	0.000212	0.002114	**C2CD2L**	9854
ENSG00000124422	-0.754239244	0.000212	0.002117	**USP22**	23326
ENSG00000175449	1.661382275	0.000212	0.002117	**RFESD**	317671
ENSG00000168026	2.156260291	0.000213	0.002122	**TTC21A**	199223
ENSG00000131378	-0.437526795	0.000213	0.002123	**RFTN1**	23180
ENSG00000110315	0.689763588	0.000213	0.002123	**RNF141**	50862
ENSG00000136938	-0.955675174	0.000214	0.002126	**ANP32B**	10541
ENSG00000132341	-0.636187493	0.000214	0.00213	**RAN**	5901
ENSG00000036549	-0.705982959	0.000215	0.002132	**ZZZ3**	26009
ENSG00000155850	0.483120405	0.000215	0.002132	**SLC26A2**	1836
ENSG00000174744	0.576289697	0.000215	0.002132	**BRMS1**	25855
ENSG00000177463	-0.366508683	0.000215	0.002132	**NR2C2**	7182
ENSG00000072310	0.684211107	0.000216	0.002137	**SREBF1**	6720
ENSG00000104833	2.582157725	0.000216	0.002137	**TUBB4A**	10382
ENSG00000115053	-0.598866865	0.000216	0.002137	**NCL**	4691
ENSG00000137216	0.778719541	0.000216	0.002137	**TMEM63B**	55362
ENSG00000141101	-0.587824048	0.000216	0.002137	**NOB1**	28987
ENSG00000108852	1.329620194	0.000216	0.002138	**MPP2**	4355
ENSG00000186767	-0.977774024	0.000216	0.002138	**SPIN4**	139886
ENSG00000128245	-0.671437394	0.000218	0.002146	**YWHAH**	7533
ENSG00000133226	-0.470525863	0.000218	0.002146	**SRRM1**	10250
ENSG00000197329	0.620755944	0.000218	0.002146	**PELI1**	57162
ENSG00000167202	0.562773349	0.000218	0.002149	**TBC1D2B**	23102
ENSG00000079616	-0.557384891	0.000219	0.002154	**KIF22**	3835
ENSG00000158793	0.7845051	0.000219	0.002154	**NIT1**	4817
ENSG00000245910	-0.543502628	0.000219	0.002154	**SNHG6**	641638
ENSG00000131323	-0.560978575	0.000219	0.002155	**TRAF3**	7187
ENSG00000187605	-0.705419124	0.00022	0.002161	**TET3**	200424
ENSG00000035681	-0.782805075	0.000221	0.002167	**NSMAF**	8439
ENSG00000113384	-0.508181347	0.000221	0.002167	**GOLPH3**	64083
ENSG00000117713	-0.873214115	0.000221	0.002167	**ARID1A**	8289
ENSG00000137168	-0.607195898	0.000221	0.002167	**PPIL1**	51645
ENSG00000139971	1.572946545	0.000221	0.002167	**ARMH4**	145407
ENSG00000152990	-0.632947486	0.000221	0.002167	**ADGRA3**	166647
ENSG00000140104	1.393433217	0.000222	0.002168	**CLBA1**	122616
ENSG00000145916	0.472833055	0.000222	0.002168	**RMND5B**	64777
ENSG00000169679	-0.363145328	0.000222	0.002168	**BUB1**	699
ENSG00000171951	1.827832785	0.000222	0.002168	**SCG2**	7857
ENSG00000151623	0.878644971	0.000222	0.00217	**NR3C2**	4306
ENSG00000162522	1.303619436	0.000224	0.002179	**KIAA1522**	57648
ENSG00000112972	0.788325651	0.000225	0.002189	**HMGCS1**	3157
ENSG00000118985	0.610631057	0.000225	0.002189	**ELL2**	22936
ENSG00000115183	0.510012988	0.000225	0.002189	**TANC1**	85461
ENSG00000139514	-0.769204518	0.000225	0.002189	**SLC7A1**	6541
ENSG00000199753	-1.465279087	0.000225	0.00219	**SNORD104**	692227
ENSG00000129187	-0.589417466	0.000225	0.00219	**DCTD**	1635
ENSG00000115415	0.531877902	0.000226	0.002192	**STAT1**	6772
ENSG00000044446	0.582773419	0.000226	0.002192	**PHKA2**	5256

ENSG00000082458	0.97209138	0.000226	0.002192	**DLG3**	1741
ENSG00000165934	-0.541151009	0.000226	0.002194	**CPSF2**	53981
ENSG00000130313	-0.637701358	0.000227	0.002199	**PGLS**	25796
ENSG00000180530	-0.829291428	0.000227	0.0022	**NRIP1**	8204
ENSG00000065060	-0.604732898	0.000228	0.002201	**UHRF1BP1**	54887
ENSG00000167306	1.044784948	0.000228	0.002201	**MYO5B**	4645
ENSG00000182504	-0.73040447	0.000228	0.002201	**CEP97**	79598
ENSG00000177885	0.513122812	0.000229	0.002208	**GRB2**	2885
ENSG00000130723	-0.611949591	0.000229	0.002212	**PRRC2B**	84726
ENSG00000103121	0.38744018	0.00023	0.002214	**CMC2**	56942
ENSG00000091136	-0.74061071	0.00023	0.002218	**LAMB1**	3912
ENSG00000142459	0.81004858	0.00023	0.002218	**EVI5L**	115704
ENSG00000143156	0.517866063	0.000231	0.002221	**NME7**	29922
ENSG00000123268	-0.754171547	0.000231	0.002222	**ATF1**	466
ENSG00000145287	-0.536108107	0.000231	0.002223	**PLAC8**	51316
ENSG00000104946	0.573156587	0.000231	0.002224	**TBC1D17**	79735
ENSG00000117155	0.405945766	0.000232	0.002225	**SSX2IP**	117178
ENSG00000140836	-0.565968992	0.000232	0.002225	**ZFHX3**	463
ENSG00000142409	-0.880750179	0.000232	0.002225	**ZNF787**	126208
ENSG00000198910	2.11329051	0.000233	0.00223	**L1CAM**	3897
ENSG00000112242	-0.81332426	0.000233	0.002233	**E2F3**	1871
ENSG00000131408	-0.803729979	0.000234	0.002241	**NR1H2**	7376
ENSG00000141524	0.638570895	0.000234	0.002241	**TMC6**	11322
ENSG00000169439	0.727431364	0.000234	0.002241	**SDC2**	6383
ENSG00000133740	-0.706602338	0.000235	0.002247	**E2F5**	1875
ENSG00000272888	-0.751869806	0.000235	0.002248	**NA**	NA
ENSG00000121058	-0.827520133	0.000236	0.002253	**COIL**	8161
ENSG00000179295	-0.259743294	0.000236	0.002253	**PTPN11**	5781
ENSG00000132357	-1.24918779	0.000237	0.002254	**CARD6**	84674
ENSG00000170293	2.259877628	0.000237	0.002254	**CMTM8**	152189
ENSG00000166145	2.46847678	0.000237	0.002256	**SPINT1**	6692
ENSG00000196588	0.567579114	0.000238	0.002261	**MRTFA**	57591
ENSG00000105767	1.542718264	0.000239	0.002267	**CADM4**	199731
ENSG00000136527	-0.383207033	0.000239	0.002267	**TRA2B**	6434
ENSG00000137824	0.59938888	0.000238	0.002267	**RMDN3**	55177
ENSG00000154065	0.637505717	0.000239	0.002267	**ANKRD29**	147463
ENSG00000167965	-0.49405675	0.000239	0.002267	**MLST8**	64223
ENSG00000171867	-0.377862133	0.000239	0.002267	**PRNP**	5621
ENSG00000156802	-0.554879121	0.00024	0.002271	**ATAD2**	29028
ENSG00000198478	0.9240931	0.000241	0.002278	**SH3BGRL2**	83699
ENSG00000123485	-0.799636188	0.000241	0.002283	**HJURP**	55355
ENSG00000171552	-0.654312104	0.000241	0.002284	**BCL2L1**	598
ENSG00000138032	0.347183828	0.000242	0.00229	**PPM1B**	5495
ENSG00000152128	-0.797290118	0.000243	0.002292	**TMEM163**	81615
ENSG00000166401	-0.787634252	0.000243	0.002294	**SERPINB8**	5271
ENSG00000197381	0.771843979	0.000243	0.002294	**ADARB1**	104
ENSG00000100650	-0.634663698	0.000244	0.002297	**SRSF5**	6430
ENSG00000106443	-0.715918779	0.000244	0.002297	**PHF14**	9678
ENSG00000058799	0.722675969	0.000244	0.002298	**YIPF1**	54432
ENSG00000143797	0.555514243	0.000244	0.002298	**MBOAT2**	129642

ENSG00000158526	-0.459819229	0.000245	0.002303	**TSR2**	90121
ENSG00000196943	0.686358832	0.000245	0.002303	**NOP9**	161424
ENSG00000164221	1.038547783	0.000247	0.002316	**CCDC112**	153733
ENSG00000197603	-0.688523632	0.000246	0.002316	**CPLANE1**	65250
ENSG00000152669	-1.06799556	0.000248	0.002327	**CCNO**	10309
ENSG00000129474	-0.50609542	0.000248	0.002327	**AJUBA**	84962
ENSG00000169871	-0.732692593	0.000249	0.00233	**TRIM56**	81844
ENSG00000183853	-0.571318268	0.000249	0.002334	**KIRREL1**	55243
ENSG00000165119	-0.353074624	0.00025	0.00234	**HNRNPK**	3190
ENSG00000060656	1.217823082	0.00025	0.002341	**PTPRU**	10076
ENSG00000109332	-0.427493121	0.00025	0.002342	**UBE2D3**	7323
ENSG00000111077	-0.677373605	0.000251	0.002346	**TNS2**	23371
ENSG00000125977	-0.680684695	0.000251	0.002346	**EIF2S2**	8894
ENSG00000135931	0.699143038	0.000251	0.002348	**ARMC9**	80210
ENSG00000137807	-0.809545167	0.000252	0.002351	**KIF23**	9493
ENSG00000094880	-0.36059903	0.000253	0.002356	**CDC23**	8697
ENSG00000120800	-0.953967954	0.000253	0.002356	**UTP20**	27340
ENSG00000139197	0.604063907	0.000253	0.002356	**PEX5**	5830
ENSG00000168813	-0.514277648	0.000253	0.002358	**ZNF507**	22847
ENSG00000153071	0.487064279	0.000253	0.002358	**DAB2**	1601
ENSG00000153071	0.487064279	0.000253	0.002358	**OC1122679**	1.12E+08
ENSG00000130147	0.496230081	0.000254	0.002366	**SH3BP4**	23677
ENSG00000182307	-0.616373443	0.000255	0.002367	**C8orf33**	65265
ENSG00000131018	0.515548585	0.000256	0.002376	**SYNE1**	23345
ENSG00000120265	0.353517386	0.000257	0.002384	**PCMT1**	5110
ENSG00000079459	0.405044137	0.000257	0.002385	**FDFT1**	2222
ENSG00000133773	-0.884622107	0.000257	0.002385	**CCDC59**	29080
ENSG00000148672	-0.382165337	0.000257	0.002385	**GLUD1**	2746
ENSG00000130202	0.369606069	0.000258	0.002386	**NECTIN2**	5819
ENSG00000162063	-0.458823588	0.000258	0.002386	**CCNF**	899
ENSG00000100836	-0.624104596	0.000259	0.002391	**PABPN1**	8106
ENSG00000178685	-0.570131062	0.000259	0.002391	**PARP10**	84875
ENSG00000170545	-0.749281562	0.000259	0.002391	**SMAGP**	57228
ENSG00000223891	1.089996799	0.000259	0.002391	**OSER1-DT**	1.01E+08
ENSG00000136021	0.46889337	0.000259	0.002392	**SCYL2**	55681
ENSG00000076321	0.559101216	0.00026	0.002394	**KLHL20**	27252
ENSG00000103356	-0.65329837	0.00026	0.002394	**EARS2**	124454
ENSG00000181523	-0.489477635	0.00026	0.002394	**SGSH**	6448
ENSG00000090674	0.677722406	0.00026	0.002395	**MCOLN1**	57192
ENSG00000133250	-1.049826042	0.00026	0.002395	**ZNF414**	84330
ENSG00000142546	0.729200304	0.000261	0.002404	**NOSIP**	51070
ENSG00000119471	0.47386449	0.000262	0.00241	**HSDL2**	84263
ENSG00000113448	-0.77780736	0.000262	0.002411	**PDE4D**	5144
ENSG00000100714	-0.280912737	0.000263	0.002412	**MTHFD1**	4522
ENSG00000110880	0.335660786	0.000263	0.002412	**CORO1C**	23603
ENSG00000130821	1.413885799	0.000263	0.002412	**SLC6A8**	6535
ENSG00000173457	-0.566346314	0.000263	0.002412	**PPP1R14B**	26472
ENSG00000181027	-1.205220157	0.000263	0.002412	**FKRP**	79147
ENSG00000123473	-0.458502068	0.000265	0.002427	**STIL**	6491
ENSG00000088247	-0.396884476	0.000265	0.002427	**KHSRP**	8570

ENSG00000185630	1.279140059	0.000265	0.002427	**PBX1**	5087
ENSG00000198959	0.467023163	0.000266	0.002428	**TGM2**	7052
ENSG00000055332	-0.549482898	0.000267	0.002438	**EIF2AK2**	5610
ENSG00000073921	0.324720639	0.000267	0.002438	**PICALM**	8301
ENSG00000103740	2.261596908	0.000267	0.002438	**ACSBG1**	23205
ENSG00000104976	0.8169238	0.000267	0.002438	**SNAPC2**	6618
ENSG00000132792	0.539874953	0.000268	0.002438	**CTNNBL1**	56259
ENSG00000214595	1.064140179	0.000268	0.002438	**EML6**	400954
ENSG00000005893	0.433819987	0.000268	0.00244	**LAMP2**	3920
ENSG00000080189	-0.554099221	0.000268	0.00244	**SLC35C2**	51006
ENSG00000197451	-0.517535771	0.000269	0.002443	**HNRNPAB**	3182
ENSG00000100105	-1.334139793	0.00027	0.00245	**PATZ1**	23598
ENSG00000144579	-0.716350016	0.00027	0.00245	**CTDSP1**	58190
ENSG00000151093	0.64344118	0.00027	0.002453	**OXSM**	54995
ENSG00000126785	-1.558751671	0.000271	0.002457	**RHOJ**	57381
ENSG00000153317	0.358549154	0.000272	0.002465	**ASAP1**	50807
ENSG00000166510	0.706480762	0.000272	0.002465	**CCDC68**	80323
ENSG00000152518	-0.539116686	0.000272	0.002467	**ZFP36L2**	678
ENSG00000079435	1.335970452	0.000274	0.002477	**LIPE**	3991
ENSG00000223749	1.064726092	0.000274	0.002481	**NA**	NA
ENSG00000109083	0.690784963	0.000275	0.002487	**IFT20**	90410
ENSG00000154146	0.666412214	0.000275	0.002489	**NRGN**	4900
ENSG00000166411	0.618210883	0.000276	0.002491	**IDH3A**	3419
ENSG00000197555	-0.681978911	0.000277	0.002498	**SIPA1L1**	26037
ENSG00000165244	-1.422818865	0.000278	0.00251	**ZNF367**	195828
ENSG00000198755	-0.295901591	0.000278	0.00251	**RPL10A**	4736
ENSG00000161813	0.439252964	0.000279	0.002514	**LARP4**	113251
ENSG00000112081	-0.488283932	0.00028	0.002519	**SRSF3**	6428
ENSG00000134202	0.911878396	0.00028	0.002519	**GSTM3**	2947
ENSG00000156966	-1.26781993	0.00028	0.002521	**B3GNT7**	93010
ENSG00000177728	-0.522366758	0.000281	0.002527	**TMEM94**	9772
ENSG00000109062	0.696710289	0.000281	0.002529	**SLC9A3R1**	9368
ENSG00000173821	-0.566452577	0.000282	0.00253	**RNF213**	57674
ENSG00000137103	2.223037478	0.000282	0.002534	**TMEM8B**	51754
ENSG00000150907	1.094394686	0.000283	0.002534	**FOXO1**	2308
ENSG00000184584	-0.859630437	0.000283	0.002534	**STING1**	340061
ENSG00000185090	-0.79842144	0.000282	0.002534	**MANEAL**	149175
ENSG00000057935	-0.85940008	0.000283	0.002534	**MTA3**	57504
ENSG00000136122	-0.547018666	0.000283	0.002538	**BORA**	79866
ENSG00000075413	0.283077558	0.000285	0.002546	**MARK3**	4140
ENSG00000197771	-0.443218053	0.000286	0.002555	**MCMBP**	79892
ENSG00000179094	0.815827334	0.000286	0.002558	**PER1**	5187
ENSG00000110013	0.74819928	0.000287	0.002565	**SIAE**	54414
ENSG00000155876	0.554213132	0.000288	0.002569	**RRAGA**	10670
ENSG00000120254	-0.666316394	0.000289	0.002574	**MTHFD1L**	25902
ENSG00000143198	0.645699687	0.000289	0.002574	**MGST3**	4259
ENSG00000166311	0.636368366	0.00029	0.00258	**SMPD1**	6609
ENSG00000132879	1.962505912	0.00029	0.002581	**FBXO44**	93611
ENSG00000105185	-0.428703665	0.000291	0.002588	**PDCD5**	9141
ENSG00000160999	1.123308501	0.000291	0.002591	**SH2B2**	10603

ENSG00000119403	-1.471144343	0.000291	0.002591	**PHF19**	26147
ENSG00000185420	0.576000894	0.000292	0.002598	**SMYD3**	64754
ENSG00000084652	-0.320868605	0.000293	0.0026	**TXLNA**	200081
ENSG00000182718	0.348290867	0.000293	0.0026	**ANXA2**	302
ENSG00000100949	-0.859606471	0.000294	0.002603	**RABGGTA**	5875
ENSG00000122515	0.489526336	0.000294	0.002603	**ZMIZ2**	83637
ENSG00000123146	-0.564276265	0.000294	0.002603	**ADGRE5**	976
ENSG00000129562	0.424734027	0.000294	0.002603	**DAD1**	1603
ENSG00000142945	-0.526963886	0.000294	0.002603	**KIF2C**	11004
ENSG00000240891	1.381917602	0.000294	0.002603	**PLCXD2**	257068
ENSG00000163513	-0.455355965	0.000295	0.002605	**TGFBR2**	7048
ENSG00000164930	-0.367130361	0.000295	0.002606	**FZD6**	8323
ENSG00000118246	-0.786016012	0.000295	0.002607	**FASTKD2**	22868
ENSG00000221963	-1.065450433	0.000296	0.002611	**APOL6**	80830
ENSG00000136770	0.577107389	0.000296	0.002615	**DNAJC1**	64215
ENSG00000276043	-1.165385231	0.000298	0.002626	**UHRF1**	29128
ENSG00000137815	0.391488244	0.000299	0.002632	**RTF1**	23168
ENSG00000134243	0.610412502	0.0003	0.002639	**SORT1**	6272
ENSG00000128805	-0.669186935	0.000301	0.002654	**ARHGAP22**	58504
ENSG00000127337	-0.781343594	0.000302	0.002655	**YEATS4**	8089
ENSG00000176244	1.135868676	0.000302	0.002655	**ACBD7**	414149
ENSG00000079999	-1.582476402	0.000303	0.002657	**KEAP1**	9817
ENSG00000099331	0.382026032	0.000303	0.002657	**MYO9B**	4650
ENSG00000150433	0.952847442	0.000303	0.002657	**TMEM218**	219854
ENSG00000163602	0.473583839	0.000303	0.002658	**RYBP**	23429
ENSG00000137221	-0.342567991	0.000304	0.002667	**TJAP1**	93643
ENSG00000162377	-0.745872376	0.000304	0.002667	**COA7**	65260
ENSG00000160949	-0.487876202	0.000305	0.002674	**TONSL**	4796
ENSG00000129933	-0.522935132	0.000306	0.002675	**MAU2**	23383
ENSG00000156675	1.461509188	0.000306	0.002675	**RAB11FIP1**	80223
ENSG00000091947	-0.599888448	0.000306	0.002679	**TMEM101**	84336
ENSG00000126870	0.704510957	0.000306	0.002679	**WDR60**	55112
ENSG00000121039	0.610720806	0.000307	0.002679	**RDH10**	157506
ENSG00000104998	-1.255909264	0.000308	0.002692	**IL27RA**	9466
ENSG00000161800	-0.615490599	0.000309	0.0027	**RACGAP1**	29127
ENSG00000085276	-0.840068167	0.00031	0.0027	**MECOM**	2122
ENSG00000184349	-1.005562003	0.00031	0.0027	**EFNA5**	1946
ENSG00000087266	-0.406141634	0.00031	0.002705	**SH3BP2**	6452
ENSG00000136141	-0.844785813	0.000312	0.002713	**LRCH1**	23143
ENSG00000145386	-0.906820534	0.000312	0.002713	**CCNA2**	890
ENSG00000158234	-1.259394079	0.000312	0.002713	**FAIM**	55179
ENSG00000076928	-0.442188809	0.000313	0.002721	**ARHGEF1**	9138
ENSG00000076928	-0.442188809	0.000313	0.002721	**OC1005055**	1.01E+08
ENSG00000146830	-0.446476336	0.000313	0.002721	**GIGYF1**	64599
ENSG00000147400	0.516238138	0.000314	0.002723	**CETN2**	1069
ENSG00000060749	-0.77237291	0.000314	0.002724	**QSER1**	79832
ENSG00000133794	1.143983455	0.000314	0.002724	**ARNTL**	406
ENSG00000028277	-0.39708486	0.000317	0.002745	**POU2F2**	5452
ENSG00000189362	-1.081379218	0.000317	0.002745	**NEMP2**	1E+08
ENSG00000106948	0.720075329	0.000318	0.002753	**AKNA**	80709

ENSG00000161217	0.514203086	0.000318	0.002756	**PCYT1A**	5130
ENSG00000171444	0.910767051	0.000319	0.002757	**MCC**	4163
ENSG00000120279	-2.111659045	0.00032	0.002765	**MYCT1**	80177
ENSG00000099804	0.321153417	0.00032	0.002766	**CDC34**	997
ENSG00000157168	-1.094079539	0.00032	0.002766	**NRG1**	3084
ENSG00000140511	1.56386505	0.000321	0.002768	**HAPLN3**	145864
ENSG00000077235	0.511180777	0.000322	0.002775	**GTF3C1**	2975
ENSG00000127334	0.525379006	0.000322	0.002775	**DYRK2**	8445
ENSG00000121064	0.85043624	0.000322	0.002776	**SCPEP1**	59342
ENSG00000179119	0.515127148	0.000325	0.002798	**SPTY2D1**	144108
ENSG00000132823	0.549452195	0.000325	0.002799	**OSER1**	51526
ENSG00000167106	0.747412054	0.000326	0.002799	**FAM102A**	399665
ENSG00000170791	0.789291583	0.000325	0.002799	**CHCHD7**	79145
ENSG00000116525	1.175762676	0.000327	0.002806	**TRIM62**	55223
ENSG00000065183	0.365277189	0.000327	0.00281	**WDR3**	10885
ENSG00000072786	-0.35206524	0.000327	0.00281	**STK10**	6793
ENSG00000138029	0.477297159	0.000327	0.00281	**HADHB**	3032
ENSG00000138696	-1.522016004	0.000328	0.002811	**BMPR1B**	658
ENSG00000160710	-0.356263953	0.000328	0.002811	**ADAR**	103
ENSG00000161682	1.340274029	0.000328	0.002811	**FAM171A2**	284069
ENSG00000105053	0.516501036	0.000329	0.002815	**VRK3**	51231
ENSG00000157693	-1.103723185	0.00033	0.002819	**TMEM268**	203197
ENSG00000166986	-0.244939208	0.00033	0.002819	**MARS1**	4141
ENSG00000198730	0.736565576	0.00033	0.002819	**CTR9**	9646
ENSG00000090889	-0.733295383	0.000331	0.002822	**KIF4A**	24137
ENSG00000132912	-0.560702276	0.000331	0.002822	**DCTN4**	51164
ENSG00000279692	-1.891663494	0.000331	0.002822	**NA**	NA
ENSG00000223959	-0.819912695	0.000331	0.002825	**AFG3L1P**	172
ENSG00000185085	0.512575173	0.000332	0.002827	**INTS5**	80789
ENSG00000062822	-0.419135718	0.000332	0.002831	**POLD1**	5424
ENSG00000125944	-0.663756573	0.000333	0.002832	**HNRNPR**	10236
ENSG00000049769	-0.963216616	0.000333	0.002834	**PPP1R3F**	89801
ENSG00000172380	-0.282255437	0.000334	0.002838	**GNG12**	55970
ENSG00000164171	-0.346099904	0.000334	0.002841	**ITGA2**	3673
ENSG00000148218	1.084128796	0.000334	0.002841	**ALAD**	210
ENSG00000160131	-0.346792718	0.000335	0.002841	**VMA21**	203547
ENSG00000078808	-0.323266144	0.000335	0.002845	**SDF4**	51150
ENSG00000272269	-1.924752037	0.000335	0.002845	**)C1053749**	1.05E+08
ENSG00000120129	1.258226353	0.000336	0.002846	**DUSP1**	1843
ENSG00000147854	-0.467218475	0.000336	0.002849	**UHRF2**	115426
ENSG00000055070	-0.541736642	0.000337	0.002855	**SZRD1**	26099
ENSG00000113595	0.537498575	0.000337	0.002855	**TRIM23**	373
ENSG00000257167	-1.354034536	0.000338	0.002858	**TMPO-AS1**	1E+08
ENSG00000079950	0.806049514	0.00034	0.002871	**STX7**	8417
ENSG00000106351	1.088690619	0.00034	0.002871	**AGFG2**	3268
ENSG00000121152	-0.602150863	0.00034	0.002871	**NCAPH**	23397
ENSG00000171067	0.590933476	0.00034	0.002871	**C11orf24**	53838
ENSG00000105227	0.831716504	0.00034	0.002873	**PRX**	57716
ENSG00000150893	0.878803431	0.000341	0.002873	**FREM2**	341640
ENSG00000136478	0.753998084	0.000341	0.002876	**TEX2**	55852

ENSG00000143324	0.626097514	0.000343	0.002887	**XPR1**	9213
ENSG00000198911	-0.309443761	0.000343	0.002887	**SREBF2**	6721
ENSG00000100983	-0.323620123	0.000344	0.002889	**GSS**	2937
ENSG00000124486	0.362931843	0.000344	0.002889	**USP9X**	8239
ENSG00000131015	0.991337116	0.000344	0.002889	**ULBP2**	80328
ENSG00000166845	-0.681170504	0.000344	0.002889	**C18orf54**	162681
ENSG00000015153	-0.653222816	0.000345	0.002891	**YAF2**	10138
ENSG00000088808	0.7014488	0.000345	0.002891	**PPP1R13B**	23368
ENSG00000124508	0.517238274	0.000345	0.002891	**BTN2A2**	10385
ENSG00000158008	5.095711357	0.000345	0.002891	**EXTL1**	2134
ENSG00000187608	1.151201689	0.000345	0.002891	**ISG15**	9636
ENSG00000205213	0.356052992	0.000345	0.002891	**LGR4**	55366
ENSG00000172927	-0.509583877	0.000346	0.002893	**MYEOV**	26579
ENSG00000168395	-0.688467087	0.000346	0.002894	**ING5**	84289
ENSG00000115008	1.608280122	0.000346	0.002896	**IL1A**	3552
ENSG00000120837	-0.354068974	0.000347	0.002899	**NFYB**	4801
ENSG00000123908	-0.645913997	0.000347	0.002899	**AGO2**	27161
ENSG00000181638	0.800645681	0.000347	0.002899	**ZFP41**	286128
ENSG00000003756	-0.627902092	0.000348	0.002899	**RBM5**	10181
ENSG00000117139	0.543422863	0.000347	0.002899	**KDM5B**	10765
ENSG00000133119	-0.522004962	0.000348	0.002905	**RFC3**	5983
ENSG00000065361	1.926806748	0.000349	0.002907	**ERBB3**	2065
ENSG00000177599	1.859629672	0.00035	0.002913	**ZNF491**	126069
ENSG00000109323	0.710096899	0.000351	0.002918	**MANBA**	4126
ENSG00000116793	-0.738604809	0.000351	0.002918	**PHTF1**	10745
ENSG00000135424	3.06242341	0.000351	0.002918	**ITGA7**	3679
ENSG00000197860	1.423075855	0.000351	0.002918	**SGTB**	54557
ENSG00000213390	-0.518874766	0.000351	0.002918	**ARHGAP19**	84986
ENSG00000100417	0.774540941	0.000352	0.00292	**PMM1**	5372
ENSG00000065154	0.485843676	0.000352	0.002921	**OAT**	4942
ENSG00000080819	0.358685953	0.000353	0.002921	**CPOX**	1371
ENSG00000126261	-0.4982607	0.000353	0.002921	**UBA2**	10054
ENSG00000134278	0.513005389	0.000352	0.002921	**SPIRE1**	56907
ENSG00000149182	-0.501631007	0.000353	0.002921	**ARFGAP2**	84364
ENSG00000135372	-0.426724201	0.000353	0.002924	**NAT10**	55226
ENSG00000158773	-0.444531191	0.000354	0.002926	**USF1**	7391
ENSG00000112624	-0.915111046	0.000354	0.002928	**BICRAL**	23506
ENSG00000138246	-0.486109804	0.000355	0.002934	**DNAJC13**	23317
ENSG00000075618	0.787237378	0.000355	0.002935	**FSCN1**	6624
ENSG00000105520	0.901977066	0.000356	0.002936	**PLPPR2**	64748
ENSG00000122482	-0.579564273	0.000356	0.002936	**ZNF644**	84146
ENSG00000166483	-1.050985058	0.000356	0.002936	**WEE1**	7465
ENSG00000187244	0.870558896	0.000357	0.002942	**BCAM**	4059
ENSG00000123739	0.873426107	0.000358	0.002945	**PLA2G12A**	81579
ENSG00000171241	-0.69427692	0.000358	0.002945	**SHCBP1**	79801
ENSG00000204130	-0.617145467	0.000358	0.002949	**RUFY2**	55680
ENSG00000214357	1.04039849	0.00036	0.002957	**NEURL1B**	54492
ENSG00000125871	-0.415430527	0.00036	0.002958	**MGME1**	92667
ENSG00000122863	-1.154722886	0.00036	0.002959	**CHST3**	9469
ENSG00000128791	0.445671606	0.000362	0.002976	**TWSG1**	57045

ENSG00000174327	1.564217774	0.000363	0.002984	**SLC16A13**	201232
ENSG00000138074	-0.557271722	0.000364	0.002985	**SLC5A6**	8884
ENSG00000151612	-1.283637049	0.000364	0.002986	**ZNF827**	152485
ENSG00000135709	0.926155939	0.000365	0.00299	**KIAA0513**	9764
ENSG00000163104	-0.644779559	0.000366	0.002995	**SMARCAD1**	56916
ENSG00000100664	-0.570153095	0.000367	0.002997	**EIF5**	1983
ENSG00000135476	-0.704402203	0.000367	0.002997	**ESPL1**	9700
ENSG00000138119	-0.504665622	0.000366	0.002997	**MYOF**	26509
ENSG00000164402	-0.462295738	0.000367	0.002997	**SEPTIN8**	23176
ENSG00000179454	0.783952827	0.000367	0.002997	**KLHL28**	54813
ENSG00000196655	0.639542453	0.000366	0.002997	**TRAPPC4**	51399
ENSG00000121210	0.433896542	0.000367	0.002997	**TMEM131L**	23240
ENSG00000121879	-0.767925039	0.000367	0.002997	**PIK3CA**	5290
ENSG00000166165	0.828203206	0.000367	0.002997	**CKB**	1152
ENSG00000163171	0.2870467	0.000368	0.003002	**CDC42EP3**	10602
ENSG00000177034	-0.547998149	0.000369	0.003002	**MTX3**	345778
ENSG00000126709	1.346361139	0.000369	0.003003	**IFI6**	2537
ENSG00000162694	0.581066098	0.000371	0.003019	**EXTL2**	2135
ENSG00000180616	1.427493008	0.000372	0.003024	**SSTR2**	6752
ENSG00000119661	0.83333474	0.000372	0.003024	**DNAL1**	83544
ENSG00000088367	1.293785017	0.000373	0.00303	**EPB41L1**	2036
ENSG00000139793	0.711893144	0.000373	0.00303	**MBNL2**	10150
ENSG00000158163	-1.198818687	0.000373	0.00303	**DZIP1L**	199221
ENSG00000105607	0.748447964	0.000374	0.00303	**GCDH**	2639
ENSG00000139436	0.418055863	0.000374	0.003031	**GIT2**	9815
ENSG00000182272	0.67522001	0.000375	0.003036	**B4GALNT4**	338707
ENSG00000188242	-0.783409445	0.000376	0.003049	**PP7080**	25845
ENSG00000106991	-0.42293454	0.000377	0.003053	**ENG**	2022
ENSG00000124459	0.52501482	0.000377	0.003053	**ZNF45**	7596
ENSG00000014123	-0.77754782	0.000378	0.003053	**UFL1**	23376
ENSG00000107669	-0.436269558	0.000378	0.003053	**ATE1**	11101
ENSG00000136108	-0.528075612	0.000379	0.00306	**CKAP2**	26586
ENSG00000015171	-1.212461126	0.000381	0.003074	**ZMYND11**	10771
ENSG00000028116	-1.041634821	0.000382	0.003074	**VRK2**	7444
ENSG00000100425	-0.903333869	0.000382	0.003074	**BRD1**	23774
ENSG00000111737	-0.303939794	0.000381	0.003074	**RAB35**	11021
ENSG00000119844	0.679761546	0.000381	0.003074	**AFTPH**	54812
ENSG00000137486	-0.511285249	0.000381	0.003074	**ARRB1**	408
ENSG00000196810	-0.858419636	0.000381	0.003074	**NA**	NA
ENSG00000164466	-0.563928381	0.000382	0.003075	**SFXN1**	94081
ENSG00000182809	2.208328642	0.000383	0.003084	**CRIP2**	1397
ENSG00000104611	-1.234114494	0.000386	0.003102	**SH2D4A**	63898
ENSG00000146233	1.826764918	0.000386	0.003104	**CYP39A1**	51302
ENSG00000138134	-1.35803614	0.000387	0.003107	**STAMBPL1**	57559
ENSG00000135537	-1.437643259	0.000387	0.003109	**AFG1L**	246269
ENSG00000158186	0.8125131	0.000389	0.003119	**MRAS**	22808
ENSG00000133318	0.425467475	0.00039	0.003125	**RTN3**	10313
ENSG00000139636	1.024468451	0.00039	0.003126	**LMBR1L**	55716
ENSG00000162729	1.530895421	0.000392	0.003143	**IGSF8**	93185
ENSG00000114686	-0.537133551	0.000393	0.003146	**MRPL3**	11222

ENSG00000175203	-0.317950466	0.000393	0.003146	**DCTN2**	10540
ENSG00000111252	-0.50761025	0.000395	0.003156	**SH2B3**	10019
ENSG00000153914	-0.88577977	0.000395	0.003161	**SREK1**	140890
ENSG00000185122	-0.328887002	0.000396	0.003163	**HSF1**	3297
ENSG00000079246	-0.355471692	0.000396	0.003163	**XRCC5**	7520
ENSG00000163995	2.049529153	0.000396	0.003163	**ABLIM2**	84448
ENSG00000100796	-0.787677503	0.000397	0.003167	**PPP4R3A**	55671
ENSG00000130304	1.287588529	0.000397	0.003167	**SLC27A1**	376497
ENSG00000140682	-1.385072357	0.000397	0.003167	**TGFB1I1**	7041
ENSG00000180543	0.871867144	0.000397	0.003167	**TSPYL5**	85453
ENSG00000132846	-0.555718157	0.000398	0.003172	**ZBED3**	84327
ENSG00000163811	-0.537604094	0.000399	0.003174	**WDR43**	23160
ENSG00000196440	-1.366792059	0.000399	0.003175	**ARMCX4**	1E+08
ENSG00000089006	-0.588319807	0.000402	0.003191	**SNX5**	27131
ENSG00000134057	-0.807494563	0.000402	0.003191	**CCNB1**	891
ENSG00000137504	-0.478938393	0.000402	0.003191	**CREBZF**	58487
ENSG00000159692	-0.595375961	0.000402	0.003191	**CTBP1**	1487
ENSG00000168566	0.505674191	0.000402	0.003191	**SNRNP48**	154007
ENSG00000185585	0.888575669	0.000401	0.003191	**OLFML2A**	169611
ENSG00000079313	-0.66201311	0.000402	0.003191	**REXO1**	57455
ENSG00000086758	-0.49032554	0.000403	0.003191	**HUWE1**	10075
ENSG00000101343	0.402281528	0.000403	0.003193	**CRNKL1**	51340
ENSG00000168904	0.943607564	0.000404	0.003199	**LRRC28**	123355
ENSG00000232098	0.460531314	0.000404	0.003199	**NA**	NA
ENSG00000043355	-1.43040112	0.000405	0.003199	**ZIC2**	7546
ENSG00000119042	-0.568708182	0.000405	0.003199	**SATB2**	23314
ENSG00000122678	0.464486718	0.000407	0.003208	**POLM**	27434
ENSG00000158373	0.938430444	0.000406	0.003208	**H2BC5**	3017
ENSG00000172775	-0.38756104	0.000406	0.003208	**PSME3IP1**	80011
ENSG00000197724	-1.310461615	0.000407	0.003208	**PHF2**	5253
ENSG00000109103	1.057486864	0.000407	0.00321	**UNC119**	9094
ENSG00000156515	0.62554643	0.000408	0.00321	**HK1**	3098
ENSG00000181744	-0.793161835	0.000408	0.00321	**DIPK2A**	205428
ENSG00000099139	1.501789609	0.000408	0.003211	**PCSK5**	5125
ENSG00000127616	-0.481165071	0.000408	0.003212	**SMARCA4**	6597
ENSG00000131351	-0.511835823	0.000408	0.003212	**HAUS8**	93323
ENSG00000115539	-0.731409109	0.000409	0.003213	**PDCL3**	79031
ENSG00000172348	1.823380635	0.00041	0.003218	**RCAN2**	10231
ENSG00000141424	0.543695008	0.000412	0.003235	**SLC39A6**	25800
ENSG00000198554	-0.446491565	0.000412	0.003235	**WDHD1**	11169
ENSG00000213626	1.590480231	0.000412	0.003235	**LBH**	81606
ENSG00000132254	0.434045267	0.000413	0.00324	**ARFIP2**	23647
ENSG00000175662	0.826380663	0.000413	0.003241	**TOM1L2**	146691
ENSG00000144231	-0.351387554	0.000414	0.003241	**POLR2D**	5433
ENSG00000132436	0.470368001	0.000414	0.003245	**FIGNL1**	63979
ENSG00000184432	0.351535145	0.000415	0.003247	**COPB2**	9276
ENSG00000051180	-0.767102611	0.000415	0.003248	**RAD51**	5888
ENSG00000084733	0.378161041	0.000416	0.003249	**RAB10**	10890
ENSG00000128016	1.021125455	0.000416	0.003251	**ZFP36**	7538
ENSG00000171408	-1.362054794	0.000416	0.003251	**PDE7B**	27115

ENSG00000164199	0.833449479	0.000417	0.003256	**ADGRV1**	84059
ENSG00000071246	1.17951633	0.000418	0.003263	**VASH1**	22846
ENSG00000167325	-0.527527341	0.000418	0.003263	**RRM1**	6240
ENSG00000179630	-1.025093538	0.000419	0.003269	**LACC1**	144811
ENSG00000125731	1.548876243	0.000422	0.003288	**SH2D3A**	10045
ENSG00000170100	0.678921928	0.000422	0.003288	**ZNF778**	197320
ENSG00000197619	0.871648312	0.000422	0.003288	**ZNF615**	284370
ENSG00000224051	0.725168925	0.000423	0.003294	**CPTP**	80772
ENSG00000062716	0.452443127	0.000424	0.003297	**VMP1**	81671
ENSG00000101407	-0.305117079	0.000425	0.003297	**TTI1**	9675
ENSG00000139737	0.74229911	0.000424	0.003297	**SLAIN1**	122060
ENSG00000172201	1.437785924	0.000424	0.003297	**ID4**	3400
ENSG00000181396	-0.55050055	0.000425	0.003301	**OGFOD3**	79701
ENSG00000131480	1.161468587	0.000426	0.003302	**AOC2**	314
ENSG00000173546	0.998731416	0.000426	0.003303	**CSPG4**	1464
ENSG00000166173	0.491244606	0.000426	0.003303	**LARP6**	55323
ENSG00000144655	0.821756473	0.000426	0.003303	**CSRNP1**	64651
ENSG00000100461	-0.795110682	0.000427	0.003305	**RBM23**	55147
ENSG00000182173	0.631736312	0.000428	0.003314	**TSEN54**	283989
ENSG00000174373	0.447258496	0.000429	0.003315	**RALGAPA1**	253959
ENSG00000261801	-0.688998774	0.000429	0.003316	**LOXL1-AS1**	1E+08
ENSG00000138646	0.540880285	0.000429	0.003318	**HERC5**	51191
ENSG00000167553	-0.439850654	0.000431	0.003325	**TUBA1C**	84790
ENSG00000157911	-0.729227347	0.000431	0.00333	**PEX10**	5192
ENSG00000179403	2.235106695	0.000432	0.00333	**VWA1**	64856
ENSG00000117410	0.578643509	0.000432	0.003332	**ATP6V0B**	533
ENSG00000116539	-0.276544971	0.000433	0.003334	**ASH1L**	55870
ENSG00000137449	-1.159497745	0.000432	0.003334	**CPEB2**	132864
ENSG00000095383	-0.502944281	0.000434	0.003339	**TBC1D2**	55357
ENSG00000164576	0.494704265	0.000434	0.003339	**SAP30L**	79685
ENSG00000198015	-0.282569067	0.000434	0.003339	**MRPL42**	28977
ENSG00000060339	-0.653733486	0.000435	0.003342	**CCAR1**	55749
ENSG00000119333	-0.368900697	0.000435	0.003342	**WDR34**	89891
ENSG00000168259	-0.598885731	0.000435	0.003342	**DNAJC7**	7266
ENSG00000121690	1.413844114	0.000437	0.003353	**DEPDC7**	91614
ENSG00000115306	-0.419789056	0.000438	0.003359	**SPTBN1**	6711
ENSG00000185621	0.530440295	0.000438	0.003359	**LMLN**	89782
ENSG00000188735	1.134955206	0.000438	0.003362	**TMEM120B**	144404
ENSG00000128699	-0.520127476	0.000439	0.003366	**ORMDL1**	94101
ENSG00000125657	1.747271591	0.00044	0.003371	**TNFSF9**	8744
ENSG00000181026	-0.501220197	0.00044	0.003371	**AEN**	64782
ENSG00000170779	-0.459518666	0.000441	0.003374	**CDCA4**	55038
ENSG00000048828	-0.360584303	0.000441	0.003376	**FAM120A**	23196
ENSG00000182379	1.554472403	0.000442	0.003377	**NXPH4**	11247
ENSG00000092853	-0.750153554	0.000442	0.003379	**CLSPN**	63967
ENSG00000226752	0.572450972	0.000443	0.003381	**CUTALP**	253039
ENSG00000100883	0.610907468	0.000444	0.003386	**SRP54**	6729
ENSG00000148840	-0.924051718	0.000444	0.003386	**PPRC1**	23082
ENSG00000170340	0.78475469	0.000444	0.003386	**B3GNT2**	10678
ENSG00000197296	0.539775871	0.000443	0.003386	**FITM2**	128486

ENSG00000196083	0.944896758	0.000445	0.00339	**IL1RAP**	3556
ENSG00000147130	-0.732375032	0.000445	0.003391	**ZMYM3**	9203
ENSG00000100410	-0.609018865	0.000446	0.003395	**PHF5A**	84844
ENSG00000106089	-0.493279625	0.000446	0.003395	**STX1A**	6804
ENSG00000088325	-0.45515018	0.000447	0.003403	**TPX2**	22974
ENSG00000170473	-0.84259332	0.000448	0.003405	**PYM1**	84305
ENSG00000091640	-1.013445016	0.000449	0.003409	**SPAG7**	9552
ENSG00000132305	-0.489465947	0.00045	0.00342	**IMMT**	10989
ENSG00000159579	0.582675155	0.00045	0.00342	**RSPRY1**	89970
ENSG00000217801	1.618701622	0.000451	0.003424	**OC1002881**	1E+08
ENSG00000088836	0.853705161	0.000455	0.003445	**SLC4A11**	83959
ENSG00000109180	-0.50716031	0.000455	0.003445	**OCIAD1**	54940
ENSG00000140545	0.674946157	0.000455	0.003445	**MFGE8**	4240
ENSG00000074410	0.872564587	0.000455	0.003447	**CA12**	771
ENSG00000180448	0.640692547	0.000455	0.003447	**ARHGAP45**	23526
ENSG00000142002	-0.391263401	0.000456	0.003453	**DPP9**	91039
ENSG00000105605	0.653821699	0.000462	0.003494	**CACNG7**	59284
ENSG00000115295	-0.729207151	0.000462	0.003494	**CLIP4**	79745
ENSG00000168779	-0.976370888	0.000463	0.003498	**SHOX2**	6474
ENSG00000150787	-0.746278975	0.000464	0.003503	**PTS**	5805
ENSG00000178409	-1.575879661	0.000464	0.003505	**BEND3**	57673
ENSG00000083937	0.525485944	0.000465	0.003511	**CHMP2B**	25978
ENSG00000196968	-0.856099634	0.000466	0.003512	**FUT11**	170384
ENSG00000150967	0.86918145	0.000466	0.003515	**ABCB9**	23457
ENSG00000117859	0.364073327	0.000467	0.003517	**OSBPL9**	114883
ENSG00000136231	-0.726762078	0.000467	0.003519	**IGF2BP3**	10643
ENSG00000180537	0.929207308	0.000467	0.003519	**RNF182**	221687
ENSG00000123191	1.449479259	0.00047	0.003535	**ATP7B**	540
ENSG00000167964	1.638481212	0.000471	0.00354	**RAB26**	25837
ENSG00000175182	0.618247654	0.000471	0.003543	**FAM131A**	131408
ENSG00000185813	0.588812114	0.000472	0.003545	**PCYT2**	5833
ENSG00000131188	-0.718082295	0.000472	0.003546	**PRR7**	80758
ENSG00000170322	0.475704908	0.000473	0.003548	**NFRKB**	4798
ENSG00000064687	1.335921043	0.000473	0.00355	**ABCA7**	10347
ENSG00000119335	-0.418194892	0.000474	0.003552	**SET**	6418
ENSG00000204946	-0.856668729	0.000475	0.003558	**ZNF783**	1E+08
ENSG00000137501	1.352906941	0.000475	0.003562	**SYTL2**	54843
ENSG00000165672	-0.297258643	0.000476	0.003566	**PRDX3**	10935
ENSG00000140548	-1.290396929	0.000476	0.003566	**ZNF710**	374655
ENSG00000137135	-0.878212326	0.000477	0.003568	**ARHGEF39**	84904
ENSG00000180370	-0.341133569	0.000477	0.003568	**PAK2**	5062
ENSG00000178075	0.972412459	0.000479	0.003582	**GRAMD1C**	54762
ENSG00000082641	-0.463431296	0.00048	0.003584	**NFE2L1**	4779
ENSG00000146085	0.419619358	0.00048	0.003586	**MMUT**	4594
ENSG00000108518	-0.315952035	0.000482	0.003596	**PFN1**	5216
ENSG00000165304	-0.579459272	0.000482	0.003596	**MELK**	9833
ENSG00000177548	-1.15918309	0.000483	0.0036	**RABEP2**	79874
ENSG00000151657	0.640355309	0.000483	0.003602	**KIN**	22944
ENSG00000171566	0.286452375	0.000485	0.00361	**PLRG1**	5356
ENSG00000164828	0.424952684	0.000486	0.003616	**SUN1**	23353

ENSG00000089682	-0.588839313	0.000486	0.003619	**RBM41**	55285
ENSG00000182628	-0.566425794	0.000488	0.003628	**SKA2**	348235
ENSG00000175220	-0.341603451	0.000488	0.003629	**ARHGAP1**	392
ENSG00000100401	-0.468432898	0.000488	0.003629	**RANGAP1**	5905
ENSG00000132153	-0.388984645	0.000489	0.003631	**DHX30**	22907
ENSG00000114812	-0.62569675	0.000489	0.003631	**VIPR1**	7433
ENSG00000172869	0.399434528	0.000489	0.003631	**DMXL1**	1657
ENSG00000143494	1.468374227	0.000491	0.00364	**VASH2**	79805
ENSG00000141401	0.618872281	0.000492	0.003648	**IMPA2**	3613
ENSG00000173083	0.765407616	0.000492	0.003648	**HPSE**	10855
ENSG00000272711	-1.137823246	0.000493	0.003653	**NA**	NA
ENSG00000081760	0.4856752	0.000494	0.003653	**AACS**	65985
ENSG00000169359	0.516535479	0.000494	0.003653	**SLC33A1**	9197
ENSG00000176024	0.871979656	0.000494	0.003653	**ZNF613**	79898
ENSG00000132467	0.36374131	0.000495	0.003654	**UTP3**	57050
ENSG00000164022	-0.583491801	0.000494	0.003654	**AIMP1**	9255
ENSG00000164142	1.283464514	0.000495	0.003654	**FAM160A1**	729830
ENSG00000197702	-0.563268925	0.000495	0.003654	**PARVA**	55742
ENSG00000068912	0.309985716	0.000497	0.003663	**ERLEC1**	27248
ENSG00000097046	-0.600088984	0.000497	0.003663	**CDC7**	8317
ENSG00000102531	-0.568125317	0.000497	0.003663	**FNDC3A**	22862
ENSG00000171100	1.105342248	0.000497	0.003663	**MTM1**	4534
ENSG00000178999	-0.796976882	0.000496	0.003663	**AURKB**	9212
ENSG00000154511	0.492314638	0.000498	0.003666	**DIPK1A**	388650
ENSG00000263001	-1.030275701	0.000498	0.003667	**GTF2I**	2969
ENSG00000164164	-0.831602221	0.0005	0.003675	**OTUD4**	54726
ENSG00000123159	0.702916148	0.0005	0.003678	**GIPC1**	10755
ENSG00000143502	1.856309967	0.0005	0.003678	**SUSD4**	55061
ENSG00000138641	0.595410866	0.000501	0.003681	**HERC3**	8916
ENSG00000162378	0.415934492	0.000501	0.003681	**ZYG11B**	79699
ENSG00000132376	0.971579987	0.000502	0.003685	**INPP5K**	51763
ENSG00000123395	0.865198022	0.000503	0.00369	**ATG101**	60673
ENSG00000136828	1.511772112	0.000503	0.00369	**RALGPS1**	9649
ENSG00000109089	0.54785879	0.000504	0.003694	**CDR2L**	30850
ENSG00000162231	0.652763393	0.000506	0.003711	**NXF1**	10482
ENSG00000162711	-0.545854935	0.000508	0.00372	**NLRP3**	114548
ENSG00000106086	0.800445022	0.000509	0.003722	**PLEKHA8**	84725
ENSG00000198924	-0.632100567	0.000509	0.003722	**DCLRE1A**	9937
ENSG00000257219	1.048247576	0.000509	0.003722	**LNCOG**	1.05E+08
ENSG00000166435	1.072014834	0.000509	0.003723	**XRRA1**	143570
ENSG00000115446	-0.697055565	0.00051	0.003723	**UNC50**	25972
ENSG00000126778	-0.436198036	0.00051	0.003723	**SIX1**	6495
ENSG00000113048	-0.45070165	0.000511	0.003732	**MRPS27**	23107
ENSG00000058804	-0.587024668	0.000513	0.003742	**NDC1**	55706
ENSG00000162775	-0.677623486	0.000513	0.003742	**RBM15**	64783
ENSG00000164542	1.124886615	0.000513	0.003742	**KIAA0895**	23366
ENSG00000136997	0.364123509	0.000515	0.003753	**MYC**	4609
ENSG00000089009	-0.414053901	0.000516	0.00376	**RPL6**	6128
ENSG00000115282	-0.564409897	0.000517	0.003761	**TTC31**	64427
ENSG00000115525	1.687434379	0.000517	0.003761	**ST3GAL5**	8869

ENSG00000134697	0.253088945	0.000517	0.003761	**GNL2**	29889
ENSG00000160783	-0.884820724	0.000517	0.003761	**PMF1**	11243
ENSG00000162645	0.798445608	0.000517	0.003761	**GBP2**	2634
ENSG00000169905	0.484241778	0.000519	0.003773	**TOR1AIP2**	163590
ENSG00000183570	1.351702848	0.000522	0.003788	**PCBP3**	54039
ENSG00000113273	-0.712089869	0.000524	0.003798	**ARSB**	411
ENSG00000151388	-0.535060541	0.000523	0.003798	**ADAMTS12**	81792
ENSG00000117266	4.065366498	0.000524	0.003799	**CDK18**	5129
ENSG00000052802	0.517904803	0.000525	0.0038	**MSMO1**	6307
ENSG00000128944	-0.640939488	0.000524	0.0038	**KNSTRN**	90417
ENSG00000146477	-0.73189354	0.000525	0.0038	**SLC22A3**	6581
ENSG00000166266	-0.697219047	0.000525	0.0038	**CUL5**	8065
ENSG00000213281	-0.388599864	0.000525	0.0038	**NRAS**	4893
ENSG00000136273	-0.594194064	0.000526	0.0038	**HUS1**	3364
ENSG00000100325	0.487798973	0.000527	0.003806	**ASCC2**	84164
ENSG00000187800	-1.044274393	0.000527	0.003808	**PEAR1**	375033
ENSG00000203666	-0.995692237	0.000527	0.003808	**EFCAB2**	84288
ENSG00000174013	0.308978246	0.000528	0.003813	**FBXO45**	200933
ENSG00000137802	0.670199324	0.000529	0.003817	**MAPKBP1**	23005
ENSG00000140691	-1.382417695	0.00053	0.003817	**ARMC5**	79798
ENSG00000146373	-0.804801985	0.00053	0.003817	**RNF217**	154214
ENSG00000021355	0.380845673	0.00053	0.003818	**SERPINB1**	1992
ENSG00000143631	0.757633353	0.00053	0.003818	**FLG**	2312
ENSG00000170915	0.719833415	0.00053	0.003818	**PAQR8**	85315
ENSG00000179361	0.871336695	0.000531	0.003818	**ARID3B**	10620
ENSG00000106771	0.273896832	0.000532	0.003823	**TMEM245**	23731
ENSG00000122741	0.434125365	0.000532	0.003823	**DCAF10**	79269
ENSG00000164543	0.30057901	0.000532	0.003823	**STK17A**	9263
ENSG00000169057	-0.583129969	0.000532	0.003823	**MECP2**	4204
ENSG00000170325	-1.491833365	0.000533	0.003823	**PRDM10**	56980
ENSG00000018699	-0.777160991	0.000533	0.003826	**TTC27**	55622
ENSG00000186871	-0.325480411	0.000533	0.003826	**ERCC6L**	54821
ENSG00000116954	0.587833285	0.000534	0.003826	**RRAGC**	64121
ENSG00000176834	-0.564103109	0.000534	0.003827	**VSIG10**	54621
ENSG00000143970	-0.427768174	0.000536	0.003839	**ASXL2**	55252
ENSG00000160216	-0.492611768	0.000536	0.003839	**AGPAT3**	56894
ENSG00000243364	1.301372946	0.000536	0.003839	**EFNA4**	1945
ENSG00000106245	0.550297862	0.000537	0.003841	**BUD31**	8896
ENSG00000099889	1.740178465	0.000538	0.003844	**ARVCF**	421
ENSG00000189057	-0.564947145	0.000538	0.003844	**FAM111B**	374393
ENSG00000144802	0.800280644	0.000538	0.003846	**NFKBIZ**	64332
ENSG00000163584	-0.3759963	0.00054	0.003857	**RPL22L1**	200916
ENSG00000156650	-1.219053802	0.000541	0.003858	**KAT6B**	23522
ENSG00000107175	0.573483193	0.000544	0.003876	**CREB3**	10488
ENSG00000158246	1.423039976	0.000543	0.003876	**TENT5B**	115572
ENSG00000104679	0.51263849	0.000544	0.003876	**R3HCC1**	203069
ENSG00000160352	-0.856477159	0.000544	0.003876	**ZNF714**	148206
ENSG00000170802	-0.50519283	0.000546	0.00389	**FOXN2**	3344
ENSG00000143847	1.871343703	0.000547	0.003893	**PPFIA4**	8497
ENSG00000101017	0.842550771	0.000548	0.003899	**CD40**	958

ENSG00000135338	1.947753862	0.000548	0.0039	**LCA5**	167691
ENSG00000188021	-0.519860217	0.000549	0.0039	**UBQLN2**	29978
ENSG00000136830	-0.370221216	0.00055	0.003907	**NIBAN2**	64855
ENSG00000116096	-0.633205129	0.000551	0.003909	**SPR**	6697
ENSG00000197780	0.38741806	0.000551	0.003909	**TAF13**	6884
ENSG00000163739	0.530674327	0.000551	0.003909	**CXCL1**	2919
ENSG00000170852	-0.931340585	0.000551	0.003909	**KBTBD2**	25948
ENSG00000137941	0.859879919	0.000552	0.003915	**TTLL7**	79739
ENSG00000047315	0.419810567	0.000553	0.003918	**POLR2B**	5431
ENSG00000125249	0.474973671	0.000553	0.003918	**RAP2A**	5911
ENSG00000005882	0.721906123	0.000554	0.00392	**PDK2**	5164
ENSG00000049249	2.168574309	0.000554	0.00392	**TNFRSF9**	3604
ENSG00000185480	-0.450632652	0.000554	0.003921	**PARPBP**	55010
ENSG00000106344	-0.369243109	0.000555	0.003924	**RBM28**	55131
ENSG00000141258	-0.46010951	0.000555	0.003924	**SGSM2**	9905
ENSG00000184203	0.397950611	0.000556	0.003925	**PPP1R2**	5504
ENSG00000067798	-0.688395449	0.000556	0.003927	**NAV3**	89795
ENSG00000274356	-4.989735893	0.000556	0.003927	**NA**	NA
ENSG00000130255	-0.265218251	0.000557	0.003928	**RPL36**	25873
ENSG00000172031	-1.368539361	0.000557	0.003928	**EPHX4**	253152
ENSG00000165417	-0.428287051	0.000558	0.00393	**GTF2A1**	2957
ENSG00000081870	0.464104306	0.000558	0.003931	**HSPB11**	51668
ENSG00000051128	-0.695004978	0.000559	0.003931	**HOMER3**	9454
ENSG00000163714	-0.479385697	0.000559	0.003931	**U2SURP**	23350
ENSG00000170955	-0.948354455	0.000558	0.003931	**CAVIN3**	112464
ENSG00000128052	1.304386421	0.000559	0.003933	**KDR**	3791
ENSG00000184005	1.383621865	0.00056	0.003936	**T6GALNAC**	256435
ENSG00000171488	-0.398684583	0.000561	0.003943	**LRRC8C**	84230
ENSG00000064309	0.932810045	0.000561	0.003943	**CDON**	50937
ENSG00000183655	0.894702897	0.000562	0.003946	**KLHL25**	64410
ENSG00000140451	-0.714598524	0.000562	0.003947	**PIF1**	80119
ENSG00000100889	-0.461222576	0.000564	0.003953	**PCK2**	5106
ENSG00000204673	-0.803355053	0.000564	0.003957	**AKT1S1**	84335
ENSG00000136379	-0.700005454	0.000566	0.003961	**ABHD17C**	58489
ENSG00000138814	0.356339351	0.000565	0.003961	**PPP3CA**	5530
ENSG00000146282	0.436353492	0.000566	0.003961	**RARS2**	57038
ENSG00000104824	-0.50858005	0.000567	0.003963	**HNRNPL**	3191
ENSG00000136167	0.761984913	0.000567	0.003963	**LCP1**	3936
ENSG00000150991	0.481920188	0.000566	0.003963	**UBC**	7316
ENSG00000158106	1.612784487	0.000567	0.003963	**RHPN1**	114822
ENSG00000198055	0.44948042	0.000567	0.003963	**GRK6**	2870
ENSG00000169021	-0.395614978	0.000569	0.003974	**UQCRFS1**	7386
ENSG00000151883	-0.720130228	0.00057	0.003976	**PARP8**	79668
ENSG00000117569	-0.886295812	0.00057	0.00398	**PTBP2**	58155
ENSG00000163347	0.976860592	0.000571	0.003981	**CLDN1**	9076
ENSG00000273062	3.330354159	0.000571	0.003981	**NA**	NA
ENSG00000087884	1.068491865	0.000572	0.003983	**AAMDC**	28971
ENSG00000159147	-0.537407493	0.000572	0.003983	**DONSON**	29980
ENSG00000127990	-0.380448266	0.000572	0.003986	**SGCE**	8910
ENSG00000188641	0.435673917	0.000573	0.003986	**DPYD**	1806

ENSG00000149313	-0.337663806	0.000573	0.003986	**AASDHPPT**	60496
ENSG00000166971	0.580666465	0.000573	0.003986	**AKTIP**	64400
ENSG00000164983	0.554450429	0.000574	0.003993	**TMEM65**	157378
ENSG00000135999	-1.230154755	0.000575	0.003994	**EPC2**	26122
ENSG00000102158	0.478692229	0.000575	0.003995	**MAGT1**	84061
ENSG00000164400	-0.770434198	0.000577	0.004002	**CSF2**	1437
ENSG00000171914	0.880101469	0.000577	0.004002	**TLN2**	83660
ENSG00000107554	-0.537087355	0.000577	0.004002	**DNMBP**	23268
ENSG00000236809	-2.199938178	0.000577	0.004003	**NA**	NA
ENSG00000001631	-0.473991583	0.000579	0.00401	**KRIT1**	889
ENSG00000088986	0.376110388	0.000579	0.004011	**DYNLL1**	8655
ENSG00000133131	-0.442150041	0.000579	0.004011	**MORC4**	79710
ENSG00000153922	-0.365151196	0.00058	0.004012	**CHD1**	1105
ENSG00000163629	0.792301964	0.000581	0.004019	**PTPN13**	5783
ENSG00000029363	-0.772985803	0.000582	0.004026	**BCLAF1**	9774
ENSG00000173442	0.440130887	0.000583	0.004029	**EHBP1L1**	254102
ENSG00000144711	0.652110423	0.000584	0.004035	**IQSEC1**	9922
ENSG00000120875	-0.520141945	0.000585	0.004038	**DUSP4**	1846
ENSG00000134884	-0.519133465	0.000585	0.004038	**ARGLU1**	55082
ENSG00000197324	-0.256663219	0.000585	0.004039	**LRP10**	26020
ENSG00000078804	0.56525653	0.000586	0.004041	**TP53INP2**	58476
ENSG00000187678	-0.49930449	0.000588	0.004056	**SPRY4**	81848
ENSG00000076604	0.552606262	0.00059	0.004066	**TRAF4**	9618
ENSG00000132199	-0.643655863	0.00059	0.004066	**ENOSF1**	55556
ENSG00000021574	0.505573226	0.000591	0.004066	**SPAST**	6683
ENSG00000107581	-0.42401801	0.000594	0.004089	**EIF3A**	8661
ENSG00000023228	-0.300947836	0.000595	0.004091	**NDUFS1**	4719
ENSG00000141753	-0.358437519	0.000595	0.004094	**IGFBP4**	3487
ENSG00000198455	-0.763062833	0.000596	0.004098	**ZXDB**	158586
ENSG00000267575	-0.911572553	0.000597	0.004102	**OC1019271**	1.02E+08
ENSG00000154845	-0.620089078	0.000598	0.004105	**PPP4R1**	9989
ENSG00000171988	-0.425422808	0.000598	0.004105	**JMJD1C**	221037
ENSG00000184481	0.835522477	0.000599	0.004108	**FOXO4**	4303
ENSG00000145819	0.721313432	0.000599	0.004109	**ARHGAP26**	23092
ENSG00000188549	-1.107103131	0.000602	0.004124	**CCDC9B**	388115
ENSG00000177169	0.67509776	0.000602	0.004125	**ULK1**	8408
ENSG00000107789	0.583871226	0.000603	0.004127	**MINPP1**	9562
ENSG00000128849	2.129350121	0.000603	0.004127	**CGNL1**	84952
ENSG00000067141	1.870944036	0.000605	0.004141	**NEO1**	4756
ENSG00000241343	-0.815092866	0.000606	0.004142	**RPL36A**	6173
ENSG00000085365	0.353405661	0.000606	0.004146	**SCAMP1**	9522
ENSG00000008394	-0.501267697	0.000608	0.004154	**MGST1**	4257
ENSG00000105281	-0.538689913	0.000608	0.004155	**SLC1A5**	6510
ENSG00000119862	1.163098734	0.00061	0.004166	**LGALSL**	29094
ENSG00000183186	2.488460028	0.00061	0.004166	**C2CD4C**	126567
ENSG00000134107	-1.067084833	0.000611	0.004167	**BHLHE40**	8553
ENSG00000154240	0.897529671	0.000611	0.004167	**CEP112**	201134
ENSG00000174574	0.398082158	0.000612	0.004172	**AKIRIN1**	79647
ENSG00000035141	-0.529466008	0.000613	0.004174	**FAM136A**	84908
ENSG00000091844	1.012915755	0.000613	0.004174	**RGS17**	26575

ENSG00000106605	0.66797911	0.000613	0.004174	**BLVRA**	644
ENSG00000205476	0.664403892	0.000613	0.004174	**CCDC85C**	317762
ENSG00000151748	0.460302456	0.000614	0.004176	**SAV1**	60485
ENSG00000241764	-1.50453783	0.000614	0.004176	**)C1019279**	1.02E+08
ENSG00000051108	0.560270152	0.000615	0.004177	**HERPUD1**	9709
ENSG00000139405	0.503960929	0.000615	0.004179	**RITA1**	84934
ENSG00000238227	-0.769887332	0.000617	0.004187	**TMEM250**	90120
ENSG00000138050	-0.587950243	0.000617	0.00419	**THUMPD2**	80745
ENSG00000166033	0.505240757	0.000619	0.004199	**HTRA1**	5654
ENSG00000136002	0.829878678	0.000622	0.004215	**ARHGEF4**	50649
ENSG00000198771	-1.70394982	0.000622	0.004217	**RCSD1**	92241
ENSG00000114867	-0.336763442	0.000625	0.004235	**EIF4G1**	1981
ENSG00000164244	0.361954936	0.000626	0.004237	**PRRC1**	133619
ENSG00000122729	0.284931678	0.000627	0.004244	**ACO1**	48
ENSG00000131023	-0.695681791	0.000627	0.004246	**LATS1**	9113
ENSG00000184602	1.487633534	0.000628	0.004246	**SNN**	8303
ENSG00000110321	-0.42566353	0.00063	0.004261	**EIF4G2**	1982
ENSG00000134490	0.958393865	0.000632	0.00427	**TMEM241**	85019
ENSG00000187479	2.767770937	0.000633	0.004273	**C11orf96**	387763
ENSG00000223784	-0.913326058	0.000633	0.004273	**NA**	NA
ENSG00000168228	-0.674578337	0.000633	0.004275	**ZCCHC4**	29063
ENSG00000151461	-0.448337446	0.000634	0.004276	**UPF2**	26019
ENSG00000213888	-1.187747135	0.000637	0.004296	**NA**	NA
ENSG00000136840	0.69218	0.000638	0.0043	**T6GALNAC**	27090
ENSG00000143545	-0.353267208	0.000639	0.004303	**RAB13**	5872
ENSG00000114126	0.446767336	0.00064	0.004305	**TFDP2**	7029
ENSG00000150540	-0.621194167	0.00064	0.004305	**HNMT**	3176
ENSG00000165959	2.415180951	0.000639	0.004305	**CLMN**	79789
ENSG00000169891	1.073808777	0.000639	0.004305	**REPS2**	9185
ENSG00000090097	0.487919211	0.000641	0.00431	**PCBP4**	57060
ENSG00000122591	0.639206799	0.000641	0.00431	**FAM126A**	84668
ENSG00000185298	-0.638425972	0.000641	0.00431	**CCDC137**	339230
ENSG00000136146	-0.473230891	0.000643	0.004315	**MED4**	29079
ENSG00000137601	0.562414981	0.000643	0.004315	**NEK1**	4750
ENSG00000151532	0.386797325	0.000646	0.004332	**VTI1A**	143187
ENSG00000153140	-0.50336414	0.000646	0.004332	**CETN3**	1070
ENSG00000165895	-0.50410288	0.000646	0.004332	**ARHGAP42**	143872
ENSG00000185189	0.72334241	0.000646	0.004332	**NRBP2**	340371
ENSG00000113578	-0.535269304	0.000648	0.004339	**FGF1**	2246
ENSG00000182934	-0.34039852	0.000648	0.004343	**SRPRA**	6734
ENSG00000088881	1.492759092	0.00065	0.004349	**EBF4**	57593
ENSG00000101199	0.541902672	0.000651	0.004353	**ARFGAP1**	55738
ENSG00000221869	-1.447909506	0.000651	0.004357	**CEBPD**	1052
ENSG00000070759	1.0626837	0.000652	0.004361	**TESK2**	10420
ENSG00000128272	-0.483222886	0.000653	0.004361	**ATF4**	468
ENSG00000198589	0.490960494	0.000653	0.004363	**LRBA**	987
ENSG00000186638	-0.665712761	0.000654	0.004364	**KIF24**	347240
ENSG00000197694	0.354834814	0.000654	0.004364	**SPTAN1**	6709
ENSG00000067208	0.631321974	0.000656	0.004378	**EVI5**	7813
ENSG00000171310	0.424094071	0.000657	0.00438	**CHST11**	50515

ENSG00000162783	0.44260934	0.000658	0.004384	**IER5**	51278
ENSG00000160679	-0.530949681	0.000658	0.004385	**CHTOP**	26097
ENSG00000125648	0.456241293	0.000658	0.004385	**SLC25A23**	79085
ENSG00000060762	0.54384968	0.000661	0.004401	**MPC1**	51660
ENSG00000164967	-0.508471223	0.000661	0.004401	**RPP25L**	138716
ENSG00000184232	0.305707129	0.000662	0.004403	**OAF**	220323
ENSG00000141510	-0.929343596	0.000663	0.004408	**TP53**	7157
ENSG00000170522	-0.594849056	0.000665	0.004417	**ELOVL6**	79071
ENSG00000211584	0.79611679	0.000665	0.004417	**SLC48A1**	55652
ENSG00000142541	-0.310822698	0.000668	0.004432	**RPL13A**	23521
ENSG00000187741	-0.370812237	0.000667	0.004432	**FANCA**	2175
ENSG00000104853	0.283393245	0.000668	0.004433	**CLPTM1**	1209
ENSG00000138175	0.81494003	0.000669	0.004439	**ARL3**	403
ENSG00000118369	0.820904993	0.00067	0.00444	**USP35**	57558
ENSG00000132781	-0.708602976	0.000671	0.004443	**MUTYH**	4595
ENSG00000155115	-0.886430783	0.000671	0.004443	**GTF3C6**	112495
ENSG00000108671	-0.448853969	0.000672	0.004443	**PSMD11**	5717
ENSG00000164151	0.357893719	0.000672	0.004443	**ICE1**	23379
ENSG00000164219	-0.738319755	0.000672	0.004443	**PGGT1B**	5229
ENSG00000164733	0.339787783	0.000671	0.004443	**CTSB**	1508
ENSG00000171634	-0.738500909	0.000673	0.004448	**BPTF**	2186
ENSG00000165030	0.774201107	0.000673	0.004449	**NFIL3**	4783
ENSG00000083097	0.945196525	0.000674	0.004453	**DOP1A**	23033
ENSG00000130021	-0.586188541	0.000675	0.004453	**PUDP**	8226
ENSG00000177485	-0.412762942	0.000675	0.004453	**ZBTB33**	10009
ENSG00000152601	-0.484792645	0.000675	0.004454	**MBNL1**	4154
ENSG00000116337	0.575111373	0.000676	0.004458	**AMPD2**	271
ENSG00000136451	-0.468440136	0.000676	0.004459	**VEZF1**	7716
ENSG00000019549	0.663088984	0.000677	0.00446	**SNAI2**	6591
ENSG00000125375	-0.521193542	0.000677	0.00446	**DMAC2L**	27109
ENSG00000115486	0.633807684	0.000678	0.004464	**GGCX**	2677
ENSG00000105851	-0.861160359	0.000679	0.00447	**PIK3CG**	5294
ENSG00000122786	0.385696813	0.00068	0.00447	**CALD1**	800
ENSG00000143368	-0.341082399	0.00068	0.00447	**SF3B4**	10262
ENSG00000149782	0.627491092	0.00068	0.00447	**PLCB3**	5331
ENSG00000119938	1.711250986	0.000681	0.004473	**PPP1R3C**	5507
ENSG00000138434	-0.477464934	0.000681	0.004473	**ITPRID2**	6744
ENSG00000187210	0.473902643	0.000681	0.004473	**GCNT1**	2650
ENSG00000103264	0.49315375	0.000682	0.004475	**FBXO31**	79791
ENSG00000196526	0.354711711	0.000684	0.004483	**AFAP1**	60312
ENSG00000123219	-0.441517	0.000685	0.004494	**CENPK**	64105
ENSG00000141179	0.516443342	0.000688	0.004505	**PCTP**	58488
ENSG00000197620	0.789179733	0.000688	0.004505	**CXorf40A**	91966
ENSG00000104361	-1.171199271	0.00069	0.004513	**NIPAL2**	79815
ENSG00000165233	0.459748604	0.00069	0.004513	**CARD19**	84270
ENSG00000187764	0.391861669	0.00069	0.004513	**SEMA4D**	10507
ENSG00000188917	-0.627554501	0.00069	0.004513	**TRMT2B**	79979
ENSG00000066739	0.57192595	0.000691	0.004516	**ATG2B**	55102
ENSG00000162623	-1.07791037	0.000691	0.004516	**TYW3**	127253
ENSG00000023734	0.232739426	0.000693	0.004529	**STRAP**	11171

ENSG00000112425	-1.252626918	0.000694	0.004532	**EPM2A**	7957
ENSG00000141068	0.667410646	0.000695	0.004535	**KSR1**	8844
ENSG00000100479	-0.648728388	0.000697	0.004546	**POLE2**	5427
ENSG00000042753	-0.21470903	0.000698	0.004548	**AP2S1**	1175
ENSG00000075975	0.399469211	0.000698	0.004548	**MKRN2**	23609
ENSG00000158480	0.665146801	0.000698	0.004549	**SPATA2**	9825
ENSG00000137094	0.803053495	0.000699	0.004551	**DNAJB5**	25822
ENSG00000223356	4.806155086	0.0007	0.004556	**NA**	NA
ENSG00000130635	0.840744563	0.0007	0.004556	**COL5A1**	1289
ENSG00000185551	-0.393429224	0.0007	0.004556	**NR2F2**	7026
ENSG00000028839	0.954952542	0.000702	0.004559	**TBPL1**	9519
ENSG00000029993	-0.618507099	0.000701	0.004559	**HMGB3**	3149
ENSG00000175334	-0.246892532	0.000702	0.004559	**BANF1**	8815
ENSG00000196505	0.53309088	0.000702	0.004559	**GDAP2**	54834
ENSG00000205517	0.89606168	0.000702	0.004559	**RGL3**	57139
ENSG00000149554	-0.596946335	0.000704	0.004571	**CHEK1**	1111
ENSG00000131067	0.798218405	0.000705	0.004576	**GGT7**	2686
ENSG00000087269	-0.371456752	0.000706	0.00458	**NOP14**	8602
ENSG00000103657	0.522621378	0.000708	0.004587	**HERC1**	8925
ENSG00000160007	0.32516226	0.000709	0.004593	**ARHGAP35**	2909
ENSG00000175221	-0.703307348	0.00071	0.004596	**MED16**	10025
ENSG00000142731	-0.88778469	0.000711	0.004605	**PLK4**	10733
ENSG00000204856	-0.723033972	0.000713	0.004613	**FAM216A**	29902
ENSG00000117682	0.578898307	0.000714	0.00462	**DHDDS**	79947
ENSG00000149136	-0.340795896	0.000715	0.00462	**SSRP1**	6749
ENSG00000049860	0.312944885	0.000715	0.004623	**HEXB**	3074
ENSG00000160953	0.528317657	0.000716	0.004624	**PWWP3A**	84939
ENSG00000107829	-0.963148889	0.000716	0.004626	**FBXW4**	6468
ENSG00000180066	-1.820575759	0.000717	0.004629	**NA**	NA
ENSG00000213079	-0.84399477	0.000718	0.004634	**SCAF8**	22828
ENSG00000130309	-0.289795988	0.000719	0.004636	**COLGALT1**	79709
ENSG00000232024	-0.68505076	0.000719	0.004636	**NA**	NA
ENSG00000135269	0.376340845	0.00072	0.004638	**TES**	26136
ENSG00000124198	-0.314597404	0.00072	0.00464	**ARFGEF2**	10564
ENSG00000250312	-0.773574719	0.000721	0.00464	**ZNF718**	255403
ENSG00000068489	-0.521569856	0.000721	0.004641	**PRR11**	55771
ENSG00000178177	-0.491264496	0.000723	0.00465	**LCORL**	254251
ENSG00000091651	-0.636493584	0.000723	0.004652	**ORC6**	23594
ENSG00000099204	0.442219799	0.000724	0.004657	**ABLIM1**	3983
ENSG00000154059	-0.575497054	0.000725	0.004657	**IMPACT**	55364
ENSG00000188368	-2.05836678	0.000725	0.004657	**PRR19**	284338
ENSG00000123737	-0.295579668	0.000726	0.00466	**EXOSC9**	5393
ENSG00000148154	0.33753089	0.000726	0.00466	**UGCG**	7357
ENSG00000160049	0.340394649	0.000727	0.004662	**DFFA**	1676
ENSG00000197217	0.394608459	0.000727	0.004665	**ENTPD4**	9583
ENSG00000138795	-1.075996492	0.000728	0.004667	**LEF1**	51176
ENSG00000174243	-0.506052583	0.00073	0.004676	**DDX23**	9416
ENSG00000084731	0.766546538	0.000731	0.004684	**KIF3C**	3797
ENSG00000138593	0.550618843	0.000731	0.004684	**SECISBP2L**	9728
ENSG00000073536	-0.443455888	0.000733	0.004689	**NLE1**	54475

ENSG00000154174	0.226963003	0.000733	0.004689	**TOMM70**	9868
ENSG00000083642	-0.5701001	0.000733	0.004691	**PDS5B**	23047
ENSG00000177565	-0.679701892	0.000734	0.004693	**TBL1XR1**	79718
ENSG00000137502	0.590261497	0.000735	0.004694	**RAB30**	27314
ENSG00000005100	-0.304001134	0.000735	0.004696	**DHX33**	56919
ENSG00000149582	1.780267786	0.000737	0.004706	**TMEM25**	84866
ENSG00000164741	0.603407961	0.000739	0.004713	**DLC1**	10395
ENSG00000168264	-0.478310718	0.000738	0.004713	**IRF2BP2**	359948
ENSG00000143442	-0.537564302	0.000739	0.004713	**POGZ**	23126
ENSG00000169247	-0.953052428	0.000741	0.004721	**SH3TC2**	79628
ENSG00000170745	1.235665621	0.000741	0.004721	**KCNS3**	3790
ENSG00000080824	0.279660129	0.000741	0.004723	**HSP90AA1**	3320
ENSG00000088205	-0.367945157	0.000743	0.00473	**DDX18**	8886
ENSG00000006432	0.464782069	0.000745	0.004733	**MAP3K9**	4293
ENSG00000106305	-0.507648575	0.000744	0.004733	**AIMP2**	7965
ENSG00000116991	1.961923603	0.000745	0.004733	**SIPA1L2**	57568
ENSG00000119888	-0.690946646	0.000744	0.004733	**EPCAM**	4072
ENSG00000132780	-0.351717657	0.000744	0.004733	**NASP**	4678
ENSG00000122376	-0.438787995	0.000746	0.004739	**SHLD2**	54537
ENSG00000011523	0.807191493	0.000747	0.004741	**CEP68**	23177
ENSG00000124789	-0.537222014	0.000747	0.004741	**NUP153**	9972
ENSG00000131437	0.939036864	0.000748	0.004743	**KIF3A**	11127
ENSG00000166012	-0.60171633	0.00075	0.004756	**TAF1D**	79101
ENSG00000165138	0.611444624	0.000751	0.004761	**ANKS6**	203286
ENSG00000184922	-1.145580585	0.000752	0.004763	**FMNL1**	752
ENSG00000226479	-0.617896271	0.000755	0.004781	**TMEM185B**	79134
ENSG00000153561	0.375222572	0.000755	0.004782	**RMND5A**	64795
ENSG00000160613	0.565709452	0.000755	0.004782	**PCSK7**	9159
ENSG00000130363	0.825439714	0.000756	0.004785	**RSPH3**	83861
ENSG00000135622	0.691277113	0.000758	0.00479	**SEMA4F**	10505
ENSG00000155090	-0.521609874	0.000757	0.00479	**KLF10**	7071
ENSG00000139832	0.686756465	0.000758	0.004791	**RAB20**	55647
ENSG00000164430	-1.266543352	0.000759	0.004792	**CGAS**	115004
ENSG00000148411	-0.373447067	0.00076	0.004797	**NACC2**	138151
ENSG00000050327	-1.574935286	0.000762	0.004805	**ARHGEF5**	7984
ENSG00000101986	0.679333454	0.000761	0.004805	**ABCD1**	215
ENSG00000112851	-0.556638637	0.000762	0.004809	**ERBIN**	55914
ENSG00000100813	-0.339069994	0.000763	0.00481	**ACIN1**	22985
ENSG00000115380	0.898742446	0.000763	0.00481	**EFEMP1**	2202
ENSG00000134684	-0.397697515	0.000764	0.00481	**YARS1**	8565
ENSG00000168710	0.35813351	0.000764	0.004814	**AHCYL1**	10768
ENSG00000152133	0.748602279	0.000765	0.004817	**GPATCH11**	253635
ENSG00000167699	-0.328896814	0.000766	0.00482	**GLOD4**	51031
ENSG00000170759	-0.563811383	0.000766	0.00482	**KIF5B**	3799
ENSG00000111596	-0.625778287	0.000768	0.004828	**CNOT2**	4848
ENSG00000147140	-0.306497046	0.000768	0.004828	**NONO**	4841
ENSG00000136895	1.362601632	0.000771	0.004842	**GARNL3**	84253
ENSG00000164209	-0.288637603	0.000771	0.004844	**SLC25A46**	91137
ENSG00000112893	-0.433269048	0.000772	0.004846	**MAN2A1**	4124
ENSG00000113732	0.419378807	0.000772	0.004846	**ATP6V0E1**	8992

ENSG00000159399	0.244024298	0.000773	0.004848	**HK2**	3099
ENSG00000115884	-0.428949344	0.000774	0.004854	**SDC1**	6382
ENSG00000144824	0.385354029	0.000775	0.004856	**PHLDB2**	90102
ENSG00000000419	0.49828475	0.000775	0.004856	**DPM1**	8813
ENSG00000051009	0.594765262	0.000775	0.004856	**FAM160A2**	84067
ENSG00000013306	0.383335997	0.000779	0.004872	**SLC25A39**	51629
ENSG00000131626	0.337648051	0.000779	0.004872	**PPFIA1**	8500
ENSG00000198646	-0.620952573	0.000779	0.004872	**NCOA6**	23054
ENSG00000134452	0.515996296	0.000781	0.004882	**FBH1**	84893
ENSG00000066455	0.388410204	0.000783	0.004894	**GOLGA5**	9950
ENSG00000115084	0.450390009	0.00079	0.004931	**SLC35F5**	80255
ENSG00000171992	0.57010945	0.000789	0.004931	**SYNPO**	11346
ENSG00000267121	-1.582426569	0.00079	0.004931	**LOC339192**	339192
ENSG00000100490	-1.014946957	0.000791	0.004939	**CDKL1**	8814
ENSG00000173548	-0.469310736	0.000795	0.004958	**SNX33**	257364
ENSG00000067057	-0.196664696	0.000795	0.00496	**PFKP**	5214
ENSG00000163898	0.748980053	0.000798	0.004975	**LIPH**	200879
ENSG00000067836	1.281904852	0.0008	0.004982	**ROGDI**	79641
ENSG00000116668	0.927353105	0.000801	0.004987	**SWT1**	54823
ENSG00000198805	0.316245078	0.000801	0.004987	**PNP**	4860
ENSG00000105186	0.456708279	0.000802	0.004989	**ANKRD27**	84079
ENSG00000159055	-0.505709577	0.000802	0.004989	**MIS18A**	54069
ENSG00000082996	0.647190905	0.000804	0.004997	**RNF13**	11342
ENSG00000168734	0.651495298	0.000804	0.004997	**PKIG**	11142
ENSG00000077254	0.422517321	0.000806	0.005002	**USP33**	23032
ENSG00000141564	-0.529300796	0.000806	0.005002	**RPTOR**	57521
ENSG00000162407	-0.818383683	0.000805	0.005002	**PLPP3**	8613
ENSG00000166562	0.528802009	0.000806	0.005002	**SEC11C**	90701
ENSG00000028137	0.736162842	0.000807	0.005004	**TNFRSF1B**	7133
ENSG00000039560	-0.498418366	0.00081	0.005022	**RAI14**	26064
ENSG00000100393	-0.651220846	0.00081	0.005022	**EP300**	2033
ENSG00000126391	0.799162899	0.00081	0.005022	**FRMD8**	83786
ENSG00000114742	-0.340171213	0.000812	0.005032	**WDR48**	57599
ENSG00000183935	0.937328881	0.000813	0.005033	**HTR7P1**	93164
ENSG00000161888	-0.608148283	0.000816	0.005045	**SPC24**	147841
ENSG00000162129	-0.661112815	0.000816	0.005045	**CLPB**	81570
ENSG00000204116	-0.552390481	0.000816	0.005045	**CHIC1**	53344
ENSG00000176994	-0.327246523	0.000817	0.005053	**SMCR8**	140775
ENSG00000094631	-0.337647304	0.000818	0.005054	**HDAC6**	10013
ENSG00000110171	0.908994342	0.000818	0.005054	**TRIM3**	10612
ENSG00000048392	0.551355143	0.000819	0.005054	**RRM2B**	50484
ENSG00000130449	0.529567574	0.000819	0.005054	**ZSWIM6**	57688
ENSG00000153815	-0.328116651	0.000819	0.005054	**CMIP**	80790
ENSG00000070501	1.270270108	0.00082	0.005055	**POLB**	5423
ENSG00000113407	-0.444206289	0.00082	0.005058	**TARS1**	6897
ENSG00000162813	0.292871205	0.000823	0.00507	**BPNT1**	10380
ENSG00000005249	0.996590673	0.000823	0.00507	**PRKAR2B**	5577
ENSG00000110851	-0.417717521	0.000823	0.00507	**PRDM4**	11108
ENSG00000111011	-0.418962939	0.000826	0.005081	**RSRC2**	65117
ENSG00000167595	-1.114308219	0.000826	0.005081	**PROSER3**	148137

ENSG00000247077	-0.301450481	0.000825	0.005081	**PGAM5**	192111
ENSG00000154328	0.556616765	0.000827	0.005088	**NEIL2**	252969
ENSG00000092847	0.40331488	0.000828	0.00509	**AGO1**	26523
ENSG00000128191	-0.929391145	0.000828	0.00509	**DGCR8**	54487
ENSG00000131747	-0.462610201	0.000829	0.00509	**TOP2A**	7153
ENSG00000243244	1.571777493	0.000829	0.005093	**STON1**	11037
ENSG00000105825	0.76494153	0.000831	0.005101	**TFPI2**	7980
ENSG00000248712	1.987068548	0.000832	0.005105	**CCDC153**	283152
ENSG00000106070	0.326120133	0.000834	0.00511	**GRB10**	2887
ENSG00000185787	-0.469998335	0.000833	0.00511	**MORF4L1**	10933
ENSG00000146678	-0.257743311	0.000835	0.005112	**IGFBP1**	3484
ENSG00000163378	-0.762685965	0.000835	0.005112	**EOGT**	285203
ENSG00000213199	1.581143213	0.000835	0.005114	**ASIC3**	9311
ENSG00000081791	-0.821646139	0.000836	0.005118	**DELE1**	9812
ENSG00000109756	0.425606751	0.000836	0.005118	**RAPGEF2**	9693
ENSG00000176853	0.596082895	0.000837	0.00512	**FAM91A1**	157769
ENSG00000269893	-0.491355205	0.000837	0.00512	**SNHG8**	1E+08
ENSG00000074181	1.999553079	0.000839	0.005126	**NOTCH3**	4854
ENSG00000117643	3.996677418	0.000839	0.005126	**MAN1C1**	57134
ENSG00000260231	1.417143841	0.000839	0.005126	**KDM7A-DT**	1E+08
ENSG00000105447	-0.550528878	0.00084	0.005128	**GRWD1**	83743
ENSG00000180190	0.744178833	0.00084	0.005128	**TDRP**	157695
ENSG00000178401	-0.875053561	0.000841	0.005131	**DNAJC22**	79962
ENSG00000037637	0.441575192	0.000842	0.005137	**FBXO42**	54455
ENSG00000121005	-0.71886173	0.000843	0.005138	**CRISPLD1**	83690
ENSG00000168078	-0.634721807	0.000843	0.005138	**PBK**	55872
ENSG00000116977	-0.581397079	0.000844	0.005139	**LGALS8**	3964
ENSG00000143590	2.329927564	0.000844	0.005139	**EFNA3**	1944
ENSG00000057757	0.39181724	0.00085	0.005167	**PITHD1**	57095
ENSG00000111300	-0.556509175	0.00085	0.005167	**NAA25**	80018
ENSG00000112167	-0.702766349	0.000849	0.005167	**SAYSD1**	55776
ENSG00000119630	0.682978301	0.00085	0.005167	**PGF**	5228
ENSG00000100379	0.815494156	0.000851	0.005171	**KCTD17**	79734
ENSG00000164944	-0.272942126	0.000851	0.005171	**VIRMA**	25962
ENSG00000173812	-0.422544763	0.000852	0.005172	**EIF1**	10209
ENSG00000130707	0.993964549	0.000852	0.005173	**ASS1**	445
ENSG00000125817	-0.336492436	0.000855	0.005186	**CENPB**	1059
ENSG00000155959	-0.569778107	0.000855	0.005187	**VBP1**	7411
ENSG00000169504	0.205331626	0.00086	0.005215	**CLIC4**	25932
ENSG00000133401	-0.775973999	0.000864	0.005237	**PDZD2**	23037
ENSG00000100266	0.378463072	0.000865	0.005242	**PACSIN2**	11252
ENSG00000143369	0.548387252	0.000867	0.005246	**ECM1**	1893
ENSG00000160075	-0.451842678	0.000867	0.005246	**SSU72**	29101
ENSG00000162244	-0.302258055	0.000867	0.005246	**RPL29**	6159
ENSG00000143153	0.369754382	0.000868	0.005249	**ATP1B1**	481
ENSG00000180758	0.73208677	0.000868	0.005249	**GPR157**	80045
ENSG00000132507	-0.358937772	0.000868	0.005249	**EIF5A**	1984
ENSG00000001036	-0.333173638	0.000869	0.005251	**FUCA2**	2519
ENSG00000081059	1.290701155	0.000872	0.005261	**TCF7**	6932
ENSG00000119636	1.062693796	0.000871	0.005261	**BBOF1**	80127

ENSG00000130766	0.681993837	0.000872	0.005261	**SESN2**	83667
ENSG00000171450	1.827652337	0.000873	0.005264	**CDK5R2**	8941
ENSG00000172057	0.764697644	0.000875	0.005277	**ORMDL3**	94103
ENSG00000166347	0.569270297	0.000876	0.005282	**CYB5A**	1528
ENSG00000075391	0.366662612	0.00088	0.005299	**RASAL2**	9462
ENSG00000148572	0.484876831	0.00088	0.005299	**NRBF2**	29982
ENSG00000184634	-0.597379036	0.00088	0.005299	**MED12**	9968
ENSG00000246763	-1.418511724	0.00088	0.0053	**RGMB-AS1**	503569
ENSG00000257354	-1.117613209	0.000882	0.005304	**NA**	NA
ENSG00000185860	-1.177279787	0.000882	0.005305	**CCDC190**	339512
ENSG00000253161	-0.904487305	0.000882	0.005305	**NA**	NA
ENSG00000111012	1.863075599	0.000883	0.005307	**CYP27B1**	1594
ENSG00000114744	0.437227286	0.000883	0.005307	**COMMD2**	51122
ENSG00000125931	2.417406805	0.000884	0.005308	**CITED1**	4435
ENSG00000157978	0.628898735	0.000885	0.005309	**LDLRAP1**	26119
ENSG00000176473	0.756529093	0.000884	0.005309	**WDR25**	79446
ENSG00000171962	1.317178096	0.000885	0.00531	**DRC3**	83450
ENSG00000172732	-0.516991052	0.000886	0.00531	**MUS81**	80198
ENSG00000008441	-0.436484862	0.000886	0.005311	**NFIX**	4784
ENSG00000139218	-0.603299866	0.000886	0.005311	**SCAF11**	9169
ENSG00000110881	0.963637395	0.000888	0.005319	**ASIC1**	41
ENSG00000095397	1.74892395	0.00089	0.005322	**WHRN**	25861
ENSG00000126522	0.639436593	0.00089	0.005322	**ASL**	435
ENSG00000126860	-0.979597691	0.00089	0.005322	**EVI2A**	2123
ENSG00000160345	1.527904656	0.000889	0.005322	**C9orf116**	138162
ENSG00000169895	0.643744786	0.000891	0.005326	**SYAP1**	94056
ENSG00000132471	0.293380172	0.000893	0.005339	**WBP2**	23558
ENSG00000164574	0.381926483	0.000896	0.005352	**GALNT10**	55568
ENSG00000060971	1.001166694	0.000897	0.005355	**ACAA1**	30
ENSG00000159176	0.495830922	0.000897	0.005357	**CSRP1**	1465
ENSG00000138834	0.570334855	0.000898	0.005358	**MAPK8IP3**	23162
ENSG00000188342	0.301507677	0.0009	0.005366	**GTF2F2**	2963
ENSG00000076770	-0.718888929	0.000904	0.00539	**MBNL3**	55796
ENSG00000109189	-0.467193207	0.000904	0.00539	**USP46**	64854
ENSG00000112742	-0.64187354	0.000905	0.005394	**TTK**	7272
ENSG00000148180	1.422483656	0.000907	0.005401	**GSN**	2934
ENSG00000176624	-0.699910671	0.000907	0.005401	**MEX3C**	51320
ENSG00000068323	-0.42201877	0.000908	0.005407	**TFE3**	7030
ENSG00000133026	0.310706004	0.00091	0.005414	**MYH10**	4628
ENSG00000179941	0.596415703	0.00091	0.005414	**BBS10**	79738
ENSG00000117036	0.502315566	0.000914	0.005432	**ETV3**	2117
ENSG00000167548	-0.52015767	0.000914	0.005432	**KMT2D**	8085
ENSG00000215808	-0.957717981	0.000915	0.005432	**LINC01139**	339535
ENSG00000265763	0.677407046	0.000914	0.005432	**ZNF488**	118738
ENSG00000135945	-0.548735518	0.000916	0.005435	**REV1**	51455
ENSG00000188419	-0.602372564	0.000916	0.005436	**CHM**	1121
ENSG00000184281	-0.427843078	0.000918	0.005443	**TSSC4**	10078
ENSG00000164081	-0.45953776	0.000918	0.005446	**TEX264**	51368
ENSG00000197757	-1.801428402	0.000919	0.005446	**HOXC6**	3223
ENSG00000084444	0.932044773	0.000924	0.005473	**FAM234B**	57613

ENSG00000070371	0.741621307	0.000927	0.005488	**CLTCL1**	8218
ENSG00000179820	0.283262495	0.000927	0.005488	**MYADM**	91663
ENSG00000205581	-0.291899567	0.000927	0.005488	**HMGN1**	3150
ENSG00000185238	-0.695626935	0.00093	0.005503	**PRMT3**	10196
ENSG00000183421	0.987403427	0.000931	0.005507	**RIPK4**	54101
ENSG00000182197	0.31220743	0.000932	0.005512	**EXT1**	2131
ENSG00000108774	0.455030876	0.000935	0.00552	**RAB5C**	5878
ENSG00000173209	-1.061795952	0.000935	0.00552	**AHSA2P**	130872
ENSG00000181450	-0.872086232	0.000935	0.00552	**ZNF678**	339500
ENSG00000196187	-0.374868601	0.000935	0.00552	**TMEM63A**	9725
ENSG00000151500	0.441919457	0.000936	0.005524	**THYN1**	29087
ENSG00000134874	0.733212149	0.000938	0.005534	**DZIP1**	22873
ENSG00000149289	-0.549536237	0.00094	0.005542	**ZC3H12C**	85463
ENSG00000123091	0.294341896	0.000941	0.005544	**RNF11**	26994
ENSG00000105221	-0.434276976	0.000942	0.005553	**AKT2**	208
ENSG00000101096	1.227888715	0.000944	0.005556	**NFATC2**	4773
ENSG00000112299	-1.016683496	0.000944	0.005556	**VNN1**	8876
ENSG00000103035	-0.629075346	0.000945	0.005563	**PSMD7**	5713
ENSG00000169710	0.39289226	0.000948	0.005577	**FASN**	2194
ENSG00000175866	0.811205976	0.000949	0.005583	**BAIAP2**	10458
ENSG00000119397	-0.622871804	0.000952	0.005599	**CNTRL**	11064
ENSG00000163874	0.333223883	0.000953	0.0056	**ZC3H12A**	80149
ENSG00000071994	-0.427381016	0.000954	0.0056	**PDCD2**	5134
ENSG00000100558	-0.382790121	0.000954	0.0056	**PLEK2**	26499
ENSG00000008118	2.386835039	0.000959	0.005628	**CAMK1G**	57172
ENSG00000127948	0.705475408	0.00096	0.005632	**POR**	5447
ENSG00000170876	-0.306146068	0.000962	0.005643	**TMEM43**	79188
ENSG00000011332	0.931373941	0.000963	0.005645	**DPF1**	8193
ENSG00000174720	-0.769340331	0.000964	0.005648	**LARP7**	51574
ENSG00000196476	0.884794335	0.000964	0.005648	**C20orf96**	140680
ENSG00000145780	0.333502718	0.000964	0.00565	**FEM1C**	56929
ENSG00000153391	-0.582074384	0.000966	0.005654	**INO80C**	125476
ENSG00000162613	-0.712536512	0.000966	0.005654	**FUBP1**	8880
ENSG00000104980	-0.460156347	0.000967	0.005654	**TIMM44**	10469
ENSG00000182359	1.05963712	0.000966	0.005654	**KBTBD3**	143879
ENSG00000115970	-0.471215712	0.000968	0.005656	**THADA**	63892
ENSG00000164291	0.681732305	0.000968	0.005656	**ARSK**	153642
ENSG00000163781	-0.465628938	0.000968	0.005657	**TOPBP1**	11073
ENSG00000169992	0.385195094	0.000969	0.005661	**NLGN2**	57555
ENSG00000196865	-0.621349283	0.00097	0.005661	**NHLRC2**	374354
ENSG00000073849	-0.563917362	0.00097	0.005663	**ST6GAL1**	6480
ENSG00000157557	0.368369108	0.000974	0.005681	**ETS2**	2114
ENSG00000196081	-1.249503736	0.000975	0.005684	**ZNF724**	440519
ENSG00000197798	0.600094067	0.000976	0.00569	**FAM118B**	79607
ENSG00000141551	-0.534790216	0.000978	0.005697	**CSNK1D**	1453
ENSG00000102786	-0.738600629	0.00098	0.005709	**INTS6**	26512
ENSG00000198668	0.252780852	0.00098	0.005709	**CALM1**	801
ENSG00000145743	-0.622011054	0.000983	0.005725	**FBXL17**	64839
ENSG00000107651	0.406934836	0.000984	0.005725	**SEC23IP**	11196
ENSG00000116984	-0.551321235	0.000984	0.005725	**MTR**	4548

ENSG00000198680	-1.314514387	0.000986	0.005735	**TUSC1**	286319
ENSG00000243069	2.773975357	0.000987	0.005738	**RHGEF26-A**	1.01E+08
ENSG00000141219	0.383738535	0.000989	0.00575	**C17orf80**	55028
ENSG00000104133	-0.455692414	0.000991	0.005754	**SPG11**	80208
ENSG00000086289	-0.550443485	0.000994	0.005773	**EPDR1**	54749
ENSG00000182534	0.374001213	0.000995	0.005777	**MXRA7**	439921
ENSG00000183778	-0.676428133	0.000996	0.005781	**B3GALT5**	10317
ENSG00000116141	-0.869649238	0.000998	0.005791	**MARK1**	4139
ENSG00000176407	-0.413286879	0.001	0.005796	**KCMF1**	56888
ENSG00000178694	-0.759739022	0.001	0.005796	**NSUN3**	63899
ENSG00000116138	0.485643513	0.001003	0.005809	**DNAJC16**	23341
ENSG00000144857	-0.774676034	0.001004	0.005814	**BOC**	91653
ENSG00000185664	2.136140864	0.001004	0.005815	**PMEL**	6490
ENSG00000255112	0.50451569	0.001006	0.005821	**CHMP1B**	57132
ENSG00000177426	-0.641413106	0.001006	0.005823	**TGIF1**	7050
ENSG00000135617	0.594173793	0.001008	0.005826	**PRADC1**	84279
ENSG00000196961	0.424703023	0.001007	0.005826	**AP2A1**	160
ENSG00000164187	0.528906149	0.001008	0.005827	**LMBRD2**	92255
ENSG00000176406	-0.734421273	0.001009	0.005828	**RIMS2**	9699
ENSG00000163349	0.258652701	0.001011	0.00584	**HIPK1**	204851
ENSG00000170035	-0.60204557	0.001013	0.005844	**UBE2E3**	10477
ENSG00000197959	1.426255324	0.001013	0.005844	**DNM3**	26052
ENSG00000088766	-0.482311002	0.001016	0.005858	**CRLS1**	54675
ENSG00000113593	-0.482893605	0.001016	0.005858	**PPWD1**	23398
ENSG00000133065	0.441256986	0.001017	0.00586	**SLC41A1**	254428
ENSG00000076003	-0.636528246	0.001017	0.00586	**MCM6**	4175
ENSG00000185591	-0.545287514	0.00102	0.005878	**SP1**	6667
ENSG00000107643	0.425967958	0.001022	0.005887	**MAPK8**	5599
ENSG00000185477	1.72980454	0.001023	0.005887	**GPRIN3**	285513
ENSG00000167257	-0.555825606	0.001025	0.005897	**RNF214**	257160
ENSG00000124207	-0.417998606	0.001027	0.005906	**CSE1L**	1434
ENSG00000172296	0.476625014	0.001027	0.005906	**SPTLC3**	55304
ENSG00000133104	-0.528208654	0.001028	0.005907	**SPART**	23111
ENSG00000063601	-0.639571025	0.00103	0.005916	**MTMR1**	8776
ENSG00000172878	-1.314413719	0.001031	0.005923	**METAP1D**	254042
ENSG00000092929	-0.445003825	0.001034	0.005938	**UNC13D**	201294
ENSG00000234498	0.983733793	0.001038	0.005958	**RPL13AP20**	387841
ENSG00000167670	-0.387781118	0.001046	0.005999	**CHAF1A**	10036
ENSG00000124406	1.109659608	0.001047	0.006001	**ATP8A1**	10396
ENSG00000129347	-0.393028003	0.001046	0.006001	**KRI1**	65095
ENSG00000264112	-0.837345226	0.001048	0.006009	**NA**	NA
ENSG00000250899	-0.538244223	0.00105	0.006013	**NA**	NA
ENSG00000143183	0.409575971	0.001052	0.006025	**TMCO1**	54499
ENSG00000183077	0.512599622	0.001053	0.00603	**AFMID**	125061
ENSG00000099995	-0.322375852	0.001054	0.00603	**SF3A1**	10291
ENSG00000123384	0.798040114	0.001055	0.006036	**LRP1**	4035
ENSG00000197321	-0.259673349	0.001061	0.006066	**SVIL**	6840
ENSG00000121766	0.709157439	0.001063	0.006073	**ZCCHC17**	51538
ENSG00000172409	0.606025211	0.001063	0.006073	**CLP1**	10978
ENSG00000170921	0.430262985	0.001064	0.006077	**TANC2**	26115

ENSG00000110931	-0.331797536	0.001067	0.006091	**CAMKK2**	10645
ENSG00000101452	-0.614345455	0.001068	0.006098	**DHX35**	60625
ENSG00000261468	-1.332054767	0.00107	0.006109	**NA**	NA
ENSG00000140829	-0.356290403	0.001071	0.006111	**DHX38**	9785
ENSG00000143891	0.691795996	0.001071	0.006111	**GALM**	130589
ENSG00000112640	0.425244366	0.001072	0.006113	**PPP2R5D**	5528
ENSG00000139726	-0.293602976	0.001073	0.006117	**DENR**	8562
ENSG00000055483	-0.364973863	0.001081	0.006152	**USP36**	57602
ENSG00000172262	-0.392689976	0.00108	0.006152	**ZNF131**	7690
ENSG00000183283	-0.446750306	0.001081	0.006152	**DAZAP2**	9802
ENSG00000165886	0.629592582	0.001082	0.006156	**UBTD1**	80019
ENSG00000167702	0.836924452	0.001083	0.006161	**KIFC2**	90990
ENSG00000141522	-0.382966305	0.001087	0.006179	**ARHGDIA**	396
ENSG00000116221	-0.281176334	0.001087	0.006181	**MRPL37**	51253
ENSG00000168610	0.345954045	0.001088	0.006181	**STAT3**	6774
ENSG00000146858	-1.21859819	0.00109	0.006188	**ZC3HAV1L**	92092
ENSG00000178585	0.56177456	0.00109	0.006188	**CTNNBIP1**	56998
ENSG00000127220	0.812272165	0.001093	0.006202	**ABHD8**	79575
ENSG00000165219	0.462979502	0.001094	0.00621	**GAPVD1**	26130
ENSG00000216937	-1.395613586	0.001097	0.006222	**CCDC7**	79741
ENSG00000075702	-0.683789254	0.0011	0.006238	**WDR62**	284403
ENSG00000160124	-0.763825056	0.001101	0.006242	**CCDC58**	131076
ENSG00000107341	-0.503877678	0.001104	0.006255	**UBE2R2**	54926
ENSG00000153162	1.423660391	0.001104	0.006256	**BMP6**	654
ENSG00000161714	0.325372846	0.001109	0.006279	**PLCD3**	113026
ENSG00000205730	-1.624966346	0.001113	0.006302	**ITPRIPL2**	162073
ENSG00000169085	1.160277866	0.001115	0.006305	**VXN**	254778
ENSG00000177000	1.085092432	0.001114	0.006305	**MTHFR**	4524
ENSG00000107819	0.289815573	0.001116	0.006309	**SFXN3**	81855
ENSG00000159720	0.496629775	0.001117	0.006312	**ATP6V0D1**	9114
ENSG00000166441	-0.266020151	0.001118	0.006317	**RPL27A**	6157
ENSG00000102974	-0.709833752	0.001119	0.006321	**CTCF**	10664
ENSG00000176927	-1.040789214	0.001119	0.006321	**EFCAB5**	374786
ENSG00000120868	0.333105222	0.00112	0.006324	**APAF1**	317
ENSG00000056097	-0.450894936	0.001123	0.006331	**ZFR**	51663
ENSG00000135486	-0.36928851	0.001122	0.006331	**HNRNPA1**	3178
ENSG00000154153	1.834249764	0.001123	0.006331	**RETREG1**	54463
ENSG00000009307	-0.504550518	0.001127	0.006353	**CSDE1**	7812
ENSG00000099998	-0.84714412	0.001128	0.006355	**GGT5**	2687
ENSG00000136104	-0.630135108	0.00113	0.006364	**RNASEH2B**	79621
ENSG00000137154	-0.191153012	0.00113	0.006364	**RPS6**	6194
ENSG00000079332	0.40985504	0.001135	0.006385	**SAR1A**	56681
ENSG00000085760	-0.534476992	0.001135	0.006385	**MTIF2**	4528
ENSG00000166888	-0.633876797	0.001135	0.006385	**STAT6**	6778
ENSG00000146731	-0.27372044	0.001138	0.006399	**CCT6A**	908
ENSG00000126790	-0.62721249	0.001141	0.006405	**L3HYPDH**	112849
ENSG00000136161	-1.637137862	0.001141	0.006405	**RCBTB2**	1102
ENSG00000178951	-0.476318096	0.001141	0.006405	**ZBTB7A**	51341
ENSG00000186907	1.207852696	0.001141	0.006405	**RTN4RL2**	349667
ENSG00000197111	-0.410396317	0.00114	0.006405	**PCBP2**	5094

ENSG00000070882	0.331858119	0.001142	0.006405	**OSBPL3**	26031
ENSG00000105298	0.398535356	0.001142	0.006405	**CACTIN**	58509
ENSG00000078369	-0.30883518	0.001144	0.006412	**GNB1**	2782
ENSG00000070785	0.439854314	0.001145	0.006418	**EIF2B3**	8891
ENSG00000150471	1.833715934	0.001148	0.006433	**ADGRL3**	23284
ENSG00000124126	1.41215885	0.001151	0.006444	**PREX1**	57580
ENSG00000143569	-0.480535516	0.001151	0.006444	**UBAP2L**	9898
ENSG00000171793	-0.787818727	0.001151	0.006444	**CTPS1**	1503
ENSG00000176454	-0.372716085	0.001152	0.006445	**LPCAT4**	254531
ENSG00000121749	-0.382943481	0.001158	0.00647	**TBC1D15**	64786
ENSG00000178695	-0.237224353	0.001158	0.00647	**KCTD12**	115207
ENSG00000183762	1.827401706	0.001157	0.00647	**KREMEN1**	83999
ENSG00000187522	-0.331693258	0.001159	0.006477	**HSPA14**	51182
ENSG00000204628	-0.179390485	0.001161	0.006487	**RACK1**	10399
ENSG00000121274	0.586507218	0.001162	0.006487	**TENT4B**	64282
ENSG00000084092	0.57467148	0.001163	0.006491	**NOA1**	84273
ENSG00000128891	0.71167169	0.001167	0.006513	**CCDC32**	90416
ENSG00000137845	0.297911346	0.001168	0.006515	**ADAM10**	102
ENSG00000186395	-0.383049407	0.001169	0.006515	**KRT10**	3858
ENSG00000104856	-0.649938728	0.001171	0.006523	**RELB**	5971
ENSG00000170043	0.340915256	0.001171	0.006523	**TRAPPC1**	58485
ENSG00000143493	-0.487363526	0.001172	0.006526	**INTS7**	25896
ENSG00000186577	0.48431961	0.001172	0.006527	**SMIM29**	221491
ENSG00000169299	0.292233041	0.001173	0.006528	**PGM2**	55276
ENSG00000138629	0.500738611	0.001175	0.006534	**UBL7**	84993
ENSG00000204767	-0.582061403	0.001175	0.006534	**INSYN2B**	1E+08
ENSG00000070087	-0.306409944	0.001176	0.006537	**PFN2**	5217
ENSG00000048162	-0.498884153	0.001179	0.006548	**NOP16**	51491
ENSG00000086589	-0.607746894	0.001179	0.006548	**RBM22**	55696
ENSG00000111412	0.358715829	0.001178	0.006548	**C12orf49**	79794
ENSG00000076706	0.628451126	0.001181	0.006553	**MCAM**	4162
ENSG00000146281	-0.689703936	0.001181	0.006553	**PM20D2**	135293
ENSG00000185347	-0.508241282	0.001182	0.00656	**TEDC1**	283643
ENSG00000179862	2.157067959	0.001184	0.006564	**CITED4**	163732
ENSG00000136935	-0.459849947	0.001184	0.006565	**GOLGA1**	2800
ENSG00000061794	-0.562137839	0.001187	0.006569	**MRPS35**	60488
ENSG00000071073	1.304143142	0.001187	0.006569	**MGAT4A**	11320
ENSG00000132635	-0.477208642	0.001186	0.006569	**PCED1A**	64773
ENSG00000135469	0.733293859	0.001186	0.006569	**COQ10A**	93058
ENSG00000144445	-0.694513775	0.001186	0.006569	**KANSL1L**	151050
ENSG00000164032	-0.385759256	0.001188	0.006569	**H2AZ1**	3015
ENSG00000188786	0.45357711	0.001188	0.006569	**MTF1**	4520
ENSG00000101400	0.691317682	0.001189	0.006575	**SNTA1**	6640
ENSG00000141543	0.349560349	0.001192	0.006588	**EIF4A3**	9775
ENSG00000151117	1.877127716	0.001193	0.006589	**TMEM86A**	144110
ENSG00000147416	0.502956806	0.001193	0.00659	**ATP6V1B2**	526
ENSG00000141655	0.646670601	0.001194	0.006594	**TNFRSF11A**	8792
ENSG00000100439	0.603728944	0.001202	0.006637	**ABHD4**	63874
ENSG00000094916	-0.633526581	0.001207	0.006656	**CBX5**	23468
ENSG00000105486	-0.457324973	0.001207	0.006656	**LIG1**	3978

ENSG00000176915	-0.559817148	0.00121	0.006672	**ANKLE2**	23141
ENSG00000111834	4.022574468	0.001215	0.006696	**RSPH4A**	345895
ENSG00000151893	0.389607184	0.001215	0.006696	**CACUL1**	143384
ENSG00000075213	-0.50921585	0.001218	0.006708	**SEMA3A**	10371
ENSG00000164342	-0.885852482	0.001218	0.006708	**TLR3**	7098
ENSG00000114861	-0.365527937	0.001219	0.006708	**FOXP1**	27086
ENSG00000256525	0.461279335	0.001221	0.006717	**POLG2**	11232
ENSG00000087460	-0.245098629	0.001223	0.006719	**GNAS**	2778
ENSG00000147852	0.536653565	0.001222	0.006719	**VLDLR**	7436
ENSG00000170540	0.213922995	0.001222	0.006719	**ARL6IP1**	23204
ENSG00000214367	0.369374212	0.001223	0.006719	**HAUS3**	79441
ENSG00000104613	-0.386119482	0.001226	0.006734	**INTS10**	55174
ENSG00000135250	0.310122912	0.001227	0.006734	**SRPK2**	6733
ENSG00000174606	-0.642704534	0.001227	0.006734	**ANGEL2**	90806
ENSG00000004142	-0.30481811	0.001228	0.006735	**POLDIP2**	26073
ENSG00000042088	-0.569341885	0.001228	0.006735	**TDP1**	55775
ENSG00000104964	-0.419715731	0.001229	0.00674	**TLE5**	166
ENSG00000130158	0.547154535	0.001233	0.006759	**DOCK6**	57572
ENSG00000107672	-0.451392482	0.001235	0.006765	**NSMCE4A**	54780
ENSG00000109572	0.389772146	0.001235	0.006765	**CLCN3**	1182
ENSG00000147535	-0.781571214	0.001235	0.006765	**PLPP5**	84513
ENSG00000161996	-0.431276415	0.001238	0.006778	**WDR90**	197335
ENSG00000127663	0.678323311	0.001239	0.006782	**KDM4B**	23030
ENSG00000146834	-1.569297943	0.001242	0.006793	**MEPCE**	56257
ENSG00000079462	0.420382051	0.001244	0.006801	**PAFAH1B3**	5050
ENSG00000102390	-1.544671557	0.001245	0.006808	**PBDC1**	51260
ENSG00000108387	2.602185356	0.001246	0.006809	**SEPTIN4**	5414
ENSG00000095380	0.46390262	0.001249	0.006821	**NANS**	54187
ENSG00000146909	-0.421454525	0.001253	0.006842	**NOM1**	64434
ENSG00000187446	0.759074102	0.001253	0.006842	**CHP1**	11261
ENSG00000156042	1.735529336	0.001254	0.006842	**CFAP70**	118491
ENSG00000100647	0.53390967	0.001256	0.006848	**SUSD6**	9766
ENSG00000108578	-0.272545217	0.001257	0.006848	**BLMH**	642
ENSG00000124523	-0.591114556	0.001256	0.006848	**SIRT5**	23408
ENSG00000228589	-1.639699155	0.001257	0.006848	**NA**	NA
ENSG00000100280	0.278890034	0.001259	0.006858	**AP1B1**	162
ENSG00000104164	-0.429423684	0.00126	0.006862	**BLOC1S6**	26258
ENSG00000064666	0.437216861	0.001261	0.006865	**CNN2**	1265
ENSG00000118193	-0.606581818	0.001265	0.006882	**KIF14**	9928
ENSG00000156256	-0.620936298	0.001265	0.006882	**USP16**	10600
ENSG00000269713	-0.433201723	0.001266	0.006883	**NBPF9**	400818
ENSG00000125618	-0.8910045	0.001271	0.006908	**PAX8**	7849
ENSG00000111057	-0.392830921	0.001275	0.006932	**KRT18**	3875
ENSG00000138398	-0.670444256	0.001278	0.006945	**PPIG**	9360
ENSG00000135776	-0.635883779	0.001279	0.006946	**ABCB10**	23456
ENSG00000144485	1.46141799	0.00128	0.006952	**HES6**	55502
ENSG00000136378	2.747524917	0.001283	0.006965	**ADAMTS7**	11173
ENSG00000103091	0.551826165	0.001284	0.006965	**WDR59**	79726
ENSG00000147403	-0.211194129	0.001295	0.007022	**RPL10**	6134
ENSG00000102100	0.499433733	0.001296	0.007024	**SLC35A2**	7355

ENSG00000133935	0.483937312	0.001297	0.007024	**ERG28**	11161
ENSG00000167182	-0.702010117	0.001297	0.007024	**SP2**	6668
ENSG00000167460	-0.291815889	0.001297	0.007024	**TPM4**	7171
ENSG00000171877	0.751149417	0.001297	0.007024	**FRMD5**	84978
ENSG00000164885	0.706901288	0.0013	0.007035	**CDK5**	1020
ENSG00000164603	0.826252432	0.001301	0.007038	**BMT2**	154743
ENSG00000170027	0.277449003	0.001303	0.007048	**YWHAG**	7532
ENSG00000145016	0.421520612	0.001308	0.007071	**RUBCN**	9711
ENSG00000260761	-1.185210202	0.001308	0.007071	**NA**	NA
ENSG00000249743	-0.971041729	0.00131	0.007079	**NA**	NA
ENSG00000164062	0.405750255	0.001311	0.007081	**APEH**	327
ENSG00000120899	1.461486307	0.001315	0.0071	**PTK2B**	2185
ENSG00000141956	-0.605928679	0.001316	0.007104	**PRDM15**	63977
ENSG00000158711	-0.893987396	0.001317	0.007108	**ELK4**	2005
ENSG00000110330	-0.32305791	0.001326	0.007148	**BIRC2**	329
ENSG00000168214	-0.298690793	0.001325	0.007148	**RBPJ**	3516
ENSG00000184445	-0.365290134	0.001326	0.007148	**KNTC1**	9735
ENSG00000173120	-0.375844567	0.001327	0.007148	**KDM2A**	22992
ENSG00000078018	1.996642653	0.001329	0.007155	**MAP2**	4133
ENSG00000169045	-0.510163488	0.001329	0.007155	**HNRNPH1**	3187
ENSG00000251322	0.702301482	0.001329	0.007155	**SHANK3**	85358
ENSG00000103707	0.661373064	0.001334	0.007178	**MTFMT**	123263
ENSG00000068724	0.682129968	0.001336	0.007184	**TTC7A**	57217
ENSG00000100353	-0.361136932	0.001336	0.007184	**EIF3D**	8664
ENSG00000043143	-1.498267238	0.001336	0.007185	**JADE2**	23338
ENSG00000121940	-0.431656376	0.001337	0.007185	**CLCC1**	23155
ENSG00000151240	0.557729829	0.001337	0.007185	**DIP2C**	22982
ENSG00000129968	0.465233967	0.001339	0.007193	**ABHD17A**	81926
ENSG00000178913	-0.39063823	0.001343	0.00721	**TAF7**	6879
ENSG00000086619	0.530573593	0.001344	0.007212	**ERO1B**	56605
ENSG00000174456	0.685881731	0.001345	0.007214	**C12orf76**	400073
ENSG00000165029	0.688608995	0.001345	0.007214	**ABCA1**	19
ENSG00000254535	-0.750344737	0.001346	0.007218	**PABPC4L**	132430
ENSG00000127124	0.347897054	0.001348	0.007223	**HIVEP3**	59269
ENSG00000231770	1.166775862	0.001349	0.007231	**NA**	NA
ENSG00000162368	-0.434846101	0.001351	0.007237	**CMPK1**	51727
ENSG00000065150	-0.329117689	0.001355	0.007254	**IPO5**	3843
ENSG00000073464	-0.489187312	0.001355	0.007254	**CLCN4**	1183
ENSG00000174004	-1.375739081	0.001357	0.00726	**NRROS**	375387
ENSG00000057294	0.598028027	0.00136	0.007274	**PKP2**	5318
ENSG00000121067	-0.685029799	0.001361	0.007276	**SPOP**	8405
ENSG00000101246	-0.344382472	0.001364	0.007289	**ARFRP1**	10139
ENSG00000008838	-0.538141287	0.001369	0.007313	**MED24**	9862
ENSG00000143162	0.574204444	0.001369	0.007313	**CREG1**	8804
ENSG00000260361	-3.83620541	0.001369	0.007313	**NA**	NA
ENSG00000139842	-0.339585098	0.001371	0.007318	**CUL4A**	8451
ENSG00000196141	-0.540146233	0.001371	0.007318	**SPATS2L**	26010
ENSG00000105640	-0.227460065	0.001373	0.007324	**RPL18A**	6142
ENSG00000101654	0.343891709	0.001377	0.007345	**RNMT**	8731
ENSG00000133056	0.796348488	0.00138	0.007358	**PIK3C2B**	5287

ENSG00000124217	0.516818387	0.001381	0.007361	**MOCS3**	27304
ENSG00000001617	0.848466892	0.001383	0.007368	**SEMA3F**	6405
ENSG00000078081	2.542585145	0.001388	0.007391	**LAMP3**	27074
ENSG00000151746	-0.567462835	0.00139	0.0074	**BICD1**	636
ENSG00000164597	0.544832042	0.001391	0.007401	**COG5**	10466
ENSG00000188596	1.849904039	0.001391	0.007403	**CFAP54**	144535
ENSG00000108557	-0.576915596	0.001392	0.007403	**RAI1**	10743
ENSG00000173193	-0.491075437	0.001392	0.007403	**PARP14**	54625
ENSG00000132603	-0.490410543	0.001393	0.007406	**NIP7**	51388
ENSG00000006747	1.416763843	0.001396	0.00742	**SCIN**	85477
ENSG00000138069	0.267560519	0.001403	0.007454	**RAB1A**	5861
ENSG00000125827	-0.567708544	0.001405	0.007459	**TMX4**	56255
ENSG00000050748	0.300010371	0.001405	0.007459	**MAPK9**	5601
ENSG00000137509	0.320499794	0.001409	0.007475	**PRCP**	5547
ENSG00000101265	0.819969033	0.001409	0.007476	**RASSF2**	9770
ENSG00000105479	2.437366972	0.001413	0.007492	**CCDC114**	93233
ENSG00000172602	1.813441983	0.001413	0.007492	**RND1**	27289
ENSG00000108262	0.655627885	0.001417	0.007504	**GIT1**	28964
ENSG00000149308	0.315142339	0.001417	0.007504	**NPAT**	4863
ENSG00000186812	0.480476017	0.001417	0.007504	**ZNF397**	84307
ENSG00000214194	-0.616186554	0.00142	0.007515	**SMIM30**	401397
ENSG00000047230	-0.783356869	0.001421	0.007519	**CTPS2**	56474
ENSG00000196712	-0.49217302	0.001421	0.007519	**NF1**	4763
ENSG00000158792	0.945033943	0.001422	0.007521	**SPATA2L**	124044
ENSG00000172828	2.181978468	0.001423	0.007521	**CES3**	23491
ENSG00000266904	1.600777605	0.001423	0.007521	**NA**	NA
ENSG00000203722	1.861614716	0.001424	0.007522	**RAET1G**	353091
ENSG00000122378	0.435218974	0.001425	0.007529	**PRXL2A**	84293
ENSG00000115504	-0.51154923	0.001428	0.007535	**EHBP1**	23301
ENSG00000142694	2.239902609	0.001427	0.007535	**EVA1B**	55194
ENSG00000181350	0.995187296	0.001428	0.007535	**LRRC75A**	388341
ENSG00000004864	-0.496447033	0.001429	0.007536	**SLC25A13**	10165
ENSG00000138821	-0.468860745	0.001431	0.007544	**SLC39A8**	64116
ENSG00000064313	-0.350028994	0.001433	0.007552	**TAF2**	6873
ENSG00000047346	0.948328538	0.001438	0.007565	**FAM214A**	56204
ENSG00000100528	-0.264935367	0.001437	0.007565	**CNIH1**	10175
ENSG00000125520	-0.392352766	0.001436	0.007565	**SLC2A4RG**	56731
ENSG00000130396	0.312237119	0.001437	0.007565	**AFDN**	4301
ENSG00000161547	-0.389890142	0.001437	0.007565	**SRSF2**	6427
ENSG00000160867	1.392863399	0.001446	0.007605	**FGFR4**	2264
ENSG00000196652	0.52937185	0.001446	0.007605	**ZKSCAN5**	23660
ENSG00000177225	-0.347890559	0.001449	0.007619	**GATD1**	347862
ENSG00000152104	-0.354279466	0.001451	0.007627	**PTPN14**	5784
ENSG00000159082	0.666082502	0.001453	0.00763	**SYNJ1**	8867
ENSG00000179335	0.355234895	0.001452	0.00763	**CLK3**	1198
ENSG00000141127	0.504510841	0.001453	0.00763	**PRPSAP2**	5636
ENSG00000234741	-0.377037899	0.001456	0.00764	**GAS5**	60674
ENSG00000129235	0.419683933	0.00146	0.00766	**TXNDC17**	84817
ENSG00000169715	0.613355456	0.00146	0.00766	**MT1E**	4493
ENSG00000135480	-0.450090316	0.001465	0.007679	**KRT7**	3855

ENSG00000144283	-0.385292276	0.00147	0.007707	**PKP4**	8502
ENSG00000086666	0.40835852	0.001473	0.00772	**ZFAND6**	54469
ENSG00000111642	0.180750129	0.001474	0.00772	**CHD4**	1108
ENSG00000137547	-0.656104832	0.001474	0.00772	**MRPL15**	29088
ENSG00000105877	1.031128654	0.001478	0.007737	**DNAH11**	8701
ENSG00000157510	-0.501439122	0.001479	0.007741	**AFAP1L1**	134265
ENSG00000105792	1.929442352	0.00148	0.007742	**CFAP69**	79846
ENSG00000134013	0.351886465	0.00148	0.007742	**LOXL2**	4017
ENSG00000162997	-2.383483062	0.001481	0.007743	**PRORSD1P**	344405
ENSG00000135318	-0.34093614	0.001484	0.007749	**NT5E**	4907
ENSG00000139289	-0.299062624	0.001483	0.007749	**PHLDA1**	22822
ENSG00000139291	-0.479344875	0.001484	0.007749	**TMEM19**	55266
ENSG00000152952	-0.24890955	0.001484	0.007749	**PLOD2**	5352
ENSG00000164938	1.129917239	0.001485	0.007749	**TP53INP1**	94241
ENSG00000022976	-0.486177387	0.001487	0.007755	**ZNF839**	55778
ENSG00000156162	-0.522766437	0.001487	0.007755	**DPY19L4**	286148
ENSG00000115756	0.269893302	0.00149	0.007769	**HPCAL1**	3241
ENSG00000163364	-0.681640657	0.001493	0.00778	**NA**	NA
ENSG00000272145	-1.6665046	0.001493	0.00778	**NA**	NA
ENSG00000196668	2.424290116	0.001495	0.007788	**LINC00173**	1E+08
ENSG00000013523	0.60159715	0.001498	0.007793	**ANGEL1**	23357
ENSG00000082438	-0.840131876	0.001498	0.007793	**COBLL1**	22837
ENSG00000107738	1.739163397	0.001498	0.007793	**VSIR**	64115
ENSG00000151012	-0.720740565	0.001498	0.007793	**SLC7A11**	23657
ENSG00000143387	1.200295667	0.001501	0.007804	**CTSK**	1513
ENSG00000196458	-0.562127597	0.001501	0.007804	**ZNF605**	1E+08
ENSG00000168005	-0.615745815	0.001502	0.007804	**SPINDOC**	144097
ENSG00000113161	0.311467974	0.001504	0.007814	**HMGCR**	3156
ENSG00000147133	-0.362104078	0.001506	0.007824	**TAF1**	6872
ENSG00000162434	0.4416074	0.001507	0.007825	**JAK1**	3716
ENSG00000189180	-0.881811206	0.00151	0.007839	**ZNF33A**	7581
ENSG00000151498	0.374516153	0.001512	0.007844	**ACAD8**	27034
ENSG00000156299	0.542655852	0.001512	0.007845	**TIAM1**	7074
ENSG00000152465	0.567377253	0.001517	0.007868	**NMT2**	9397
ENSG00000161681	-1.04012248	0.001519	0.007875	**SHANK1**	50944
ENSG00000101138	-0.304271644	0.001521	0.007879	**CSTF1**	1477
ENSG00000161904	-0.352413612	0.001521	0.007879	**LEMD2**	221496
ENSG00000101347	0.478223086	0.001522	0.007882	**SAMHD1**	25939
ENSG00000165060	-0.63177442	0.001522	0.007882	**FXN**	2395
ENSG00000157625	0.592018626	0.001524	0.007885	**TAB3**	257397
ENSG00000181634	0.656563266	0.00153	0.007916	**TNFSF15**	9966
ENSG00000181104	-0.320559395	0.001531	0.007917	**F2R**	2149
ENSG00000005238	0.426979077	0.001532	0.007919	**FAM214B**	80256
ENSG00000120063	-0.285873552	0.001532	0.007919	**GNA13**	10672
ENSG00000148384	-0.548660213	0.001532	0.007919	**INPP5E**	56623
ENSG00000113391	-0.476344686	0.001534	0.007922	**FAM172A**	83989
ENSG00000164509	-0.4531911	0.001535	0.007924	**IL31RA**	133396
ENSG00000184056	0.553316721	0.001535	0.007924	**VPS33B**	26276
ENSG00000198892	1.057379336	0.001539	0.007941	**SHISA4**	149345
ENSG00000264364	0.529129698	0.001541	0.007949	**DYNLL2**	140735

ENSG00000004487	0.284472207	0.001543	0.007956	**KDM1A**	23028
ENSG00000242372	-0.22831507	0.001546	0.00797	**EIF6**	3692
ENSG00000121310	-0.720780391	0.001547	0.007971	**ECHDC2**	55268
ENSG00000160298	-0.485016449	0.001547	0.007972	**C21orf58**	54058
ENSG00000132405	0.3606794	0.001548	0.007975	**TBC1D14**	57533
ENSG00000116809	-0.591936625	0.001552	0.007984	**ZBTB17**	7709
ENSG00000136718	-0.469357914	0.001551	0.007984	**IMP4**	92856
ENSG00000167657	0.643403005	0.001551	0.007984	**DAPK3**	1613
ENSG00000122877	1.769635566	0.001553	0.007986	**EGR2**	1959
ENSG00000164403	-1.111390792	0.001558	0.00801	**SHROOM1**	134549
ENSG00000079134	-0.39986407	0.001559	0.008014	**THOC1**	9984
ENSG00000006468	-0.426518601	0.001563	0.00803	**ETV1**	2115
ENSG00000126653	-0.581506648	0.001564	0.008035	**NSRP1**	84081
ENSG00000163191	-0.272444355	0.001567	0.008048	**S100A11**	6282
ENSG00000255717	-0.278426084	0.001568	0.008051	**SNHG1**	23642
ENSG00000116791	0.411669959	0.001569	0.008052	**CRYZ**	1429
ENSG00000162065	0.483577583	0.001569	0.008052	**TBC1D24**	57465
ENSG00000131069	0.960868469	0.001571	0.008055	**ACSS2**	55902
ENSG00000142627	-0.221565012	0.001571	0.008055	**EPHA2**	1969
ENSG00000197461	-0.789224974	0.001571	0.008055	**PDGFA**	5154
ENSG00000065308	0.27683689	0.001576	0.008074	**TRAM2**	9697
ENSG00000138092	-0.849347971	0.001576	0.008074	**CENPO**	79172
ENSG00000204469	-3.222042589	0.001577	0.008075	**PRRC2A**	7916
ENSG00000167110	0.285118822	0.001578	0.008077	**GOLGA2**	2801
ENSG00000231205	-0.953609556	0.001579	0.00808	**NA**	NA
ENSG00000143222	-0.551166694	0.001587	0.008117	**UFC1**	51506
ENSG00000077782	0.488855643	0.001588	0.008122	**FGFR1**	2260
ENSG00000040531	0.62495101	0.00159	0.008126	**CTNS**	1497
ENSG00000163002	-0.83532431	0.00159	0.008128	**NUP35**	129401
ENSG00000103066	0.685941357	0.001593	0.008137	**PLA2G15**	23659
ENSG00000198929	0.930160921	0.001595	0.008145	**NOS1AP**	9722
ENSG00000254004	0.445962106	0.001595	0.008145	**ZNF260**	339324
ENSG00000100599	-0.690515705	0.001606	0.008197	**RIN3**	79890
ENSG00000083544	-0.811207328	0.001607	0.008201	**TDRD3**	81550
ENSG00000067082	0.273187731	0.001608	0.008205	**KLF6**	1316
ENSG00000168092	-0.275449859	0.00161	0.008209	**PAFAH1B2**	5049
ENSG00000142327	-0.583246043	0.001612	0.008215	**RNPEPL1**	57140
ENSG00000167258	-0.474561557	0.001614	0.008225	**CDK12**	51755
ENSG00000125798	-0.641909961	0.001618	0.008243	**FOXA2**	3170
ENSG00000165140	-0.445195022	0.001621	0.008254	**FBP1**	2203
ENSG00000134108	0.260227876	0.001622	0.008254	**ARL8B**	55207
ENSG00000163322	0.550574323	0.001622	0.008254	**ABRAXAS1**	84142
ENSG00000165733	-0.268122419	0.001622	0.008254	**BMS1**	9790
ENSG00000165338	-0.539387181	0.001626	0.008269	**HECTD2**	143279
ENSG00000172466	-0.524769522	0.001627	0.008273	**ZNF24**	7572
ENSG00000197746	0.364269351	0.001627	0.008273	**PSAP**	5660
ENSG00000068831	4.757297115	0.001628	0.008273	**RASGRP2**	10235
ENSG00000186448	0.453224173	0.001629	0.008273	**ZNF197**	10168
ENSG00000186448	0.453224173	0.001629	0.008273	**F660-ZNF1**	1.1E+08
ENSG00000171345	1.592338975	0.001631	0.008284	**KRT19**	3880

ENSG00000162654	-1.102444161	0.001632	0.008284	**GBP4**	115361
ENSG00000164144	0.421040781	0.001635	0.008296	**ARFIP1**	27236
ENSG00000149428	0.317050087	0.001635	0.008296	**HYOU1**	10525
ENSG00000156795	0.710336254	0.001638	0.008307	**WDYHV1**	55093
ENSG00000167977	0.498291929	0.001639	0.00831	**KCTD5**	54442
ENSG00000154429	0.532543474	0.00164	0.008315	**CCSAP**	126731
ENSG00000153956	-0.621080259	0.001649	0.008354	**CACNA2D1**	781
ENSG00000104290	1.197430879	0.001651	0.008362	**FZD3**	7976
ENSG00000170265	-0.623093333	0.001653	0.008373	**ZNF282**	8427
ENSG00000134644	-0.281425675	0.001655	0.00838	**PUM1**	9698
ENSG00000114353	0.429790782	0.001657	0.008384	**GNAI2**	2771
ENSG00000159214	1.328342141	0.001659	0.008394	**CCDC24**	149473
ENSG00000187699	-1.640755543	0.001663	0.008408	**C2orf88**	84281
ENSG00000259673	-0.952267433	0.001665	0.008419	**IQCH-AS1**	1.01E+08
ENSG00000223496	-0.584775832	0.001668	0.008432	**EXOSC6**	118460
ENSG00000184208	-0.354874351	0.001671	0.008443	**C22orf46**	79640
ENSG00000162076	0.786990739	0.001672	0.008444	**FLYWCH2**	114984
ENSG00000172339	-0.850769664	0.001672	0.008444	**ALG14**	199857
ENSG00000136826	0.663175988	0.001674	0.008449	**KLF4**	9314
ENSG00000145050	0.306915035	0.001677	0.008464	**MANF**	7873
ENSG00000156873	0.608957724	0.001679	0.008467	**PHKG2**	5261
ENSG00000254470	-0.498428457	0.001679	0.008467	**AP5B1**	91056
ENSG00000204789	1.890516511	0.00168	0.008468	**NA**	NA
ENSG00000102900	0.388042282	0.00168	0.008468	**NUP93**	9688
ENSG00000159593	-0.467523646	0.001682	0.008473	**NAE1**	8883
ENSG00000214655	0.595722064	0.001682	0.008473	**ZSWIM8**	23053
ENSG00000035499	-0.379089573	0.001686	0.008488	**DEPDC1B**	55789
ENSG00000130024	-0.425419152	0.001687	0.008493	**PHF10**	55274
ENSG00000106459	-0.523876307	0.001688	0.008494	**NRF1**	4899
ENSG00000176531	0.847194184	0.001689	0.008494	**PHLDB3**	653583
ENSG00000029725	0.281328498	0.001692	0.008499	**RABEP1**	9135
ENSG00000116729	-0.296752078	0.001692	0.008499	**WLS**	79971
ENSG00000124588	0.346094345	0.001691	0.008499	**NQO2**	4835
ENSG00000141994	-1.279784389	0.00169	0.008499	**DUS3L**	56931
ENSG00000197557	0.745684561	0.001695	0.008511	**TTC30A**	92104
ENSG00000181004	1.080348466	0.001695	0.008513	**BBS12**	166379
ENSG00000110395	0.405036652	0.001697	0.008515	**CBL**	867
ENSG00000165209	0.48753543	0.001701	0.008536	**STRBP**	55342
ENSG00000136643	0.503815939	0.001702	0.008538	**RPS6KC1**	26750
ENSG00000175984	1.128172005	0.001703	0.008539	**DENND2C**	163259
ENSG00000154222	-0.590591288	0.001704	0.008543	**CC2D1B**	200014
ENSG00000151062	-0.424613397	0.001705	0.008544	**CACNA2D4**	93589
ENSG00000136490	-1.194510411	0.001707	0.00855	**LIMD2**	80774
ENSG00000041357	-0.273389574	0.001708	0.008555	**PSMA4**	5685
ENSG00000095752	-0.489411438	0.001717	0.008595	**IL11**	3589
ENSG00000188322	3.072582639	0.001721	0.008615	**SBK1**	388228
ENSG00000205758	0.526620515	0.001722	0.008616	**CRYZL1**	9946
ENSG00000141252	0.53164753	0.001723	0.008619	**VPS53**	55275
ENSG00000134758	-0.493177302	0.001724	0.00862	**RNF138**	51444
ENSG00000118276	-0.511830157	0.001725	0.008625	**B4GALT6**	9331

ENSG00000077147	-0.379364755	0.001732	0.008655	**TM9SF3**	56889
ENSG00000196371	-0.859148159	0.001735	0.008667	**FUT4**	2526
ENSG00000119684	-0.536372204	0.001738	0.008672	**MLH3**	27030
ENSG00000134186	-0.375339868	0.001737	0.008672	**PRPF38B**	55119
ENSG00000143801	0.520438055	0.001738	0.008672	**PSEN2**	5664
ENSG00000170312	-0.331533214	0.001737	0.008672	**CDK1**	983
ENSG00000135048	0.350127734	0.001743	0.008693	**CEMIP2**	23670
ENSG00000161921	2.094846485	0.001743	0.008693	**CXCL16**	58191
ENSG00000152700	0.470300638	0.00175	0.008725	**SAR1B**	51128
ENSG00000103522	5.392527997	0.001751	0.008725	**IL21R**	50615
ENSG00000130517	0.534947777	0.001752	0.008725	**PGPEP1**	54858
ENSG00000088305	0.452608735	0.001758	0.008745	**DNMT3B**	1789
ENSG00000104904	0.246968899	0.001757	0.008745	**OAZ1**	4946
ENSG00000106100	-1.226084899	0.001759	0.008745	**NOD1**	10392
ENSG00000106976	0.640650777	0.001758	0.008745	**DNM1**	1759
ENSG00000125691	-0.189668798	0.001759	0.008745	**RPL23**	9349
ENSG00000127526	0.298456565	0.001759	0.008745	**SLC35E1**	79939
ENSG00000139988	2.815536207	0.001759	0.008745	**RDH12**	145226
ENSG00000120053	0.492180538	0.001761	0.008748	**GOT1**	2805
ENSG00000224660	1.372736971	0.001761	0.008748	**SH3BP5-AS**	1.01E+08
ENSG00000121579	-0.276305212	0.001763	0.008748	**NAA50**	80218
ENSG00000158467	0.747850361	0.001762	0.008748	**AHCYL2**	23382
ENSG00000197386	0.326528379	0.001762	0.008748	**HTT**	3064
ENSG00000137801	1.403401899	0.001766	0.008764	**THBS1**	7057
ENSG00000100241	0.392573002	0.00177	0.00878	**SBF1**	6305
ENSG00000272886	-0.560378752	0.001771	0.008782	**DCP1A**	55802
ENSG00000136492	-0.649570814	0.001774	0.008792	**BRIP1**	83990
ENSG00000124120	0.405473435	0.00178	0.008818	**TTPAL**	79183
ENSG00000198198	0.487381368	0.001781	0.008822	**SZT2**	23334
ENSG00000075624	0.27277493	0.001782	0.008825	**ACTB**	60
ENSG00000204104	0.586028372	0.001785	0.008835	**TRAF3IP1**	26146
ENSG00000197961	-0.547449744	0.001794	0.008877	**ZNF121**	7675
ENSG00000188010	0.773172811	0.001802	0.008914	**MORN2**	729967
ENSG00000166532	-0.542998059	0.001803	0.008917	**RIMKLB**	57494
ENSG00000163909	2.568671173	0.001804	0.00892	**HEYL**	26508
ENSG00000110318	0.732010134	0.001806	0.008924	**CEP126**	57562
ENSG00000167272	0.396859417	0.001807	0.008924	**POP5**	51367
ENSG00000282418	2.659923672	0.001805	0.008924	**NA**	NA
ENSG00000104885	-0.54814244	0.00181	0.008933	**DOT1L**	84444
ENSG00000118680	0.22481322	0.001809	0.008933	**MYL12B**	103910
ENSG00000162601	0.469081881	0.00181	0.008933	**MYSM1**	114803
ENSG00000011405	0.385412712	0.001813	0.008941	**PIK3C2A**	5286
ENSG00000167378	0.612287851	0.001812	0.008941	**IRGQ**	126298
ENSG00000012048	-0.486163368	0.001814	0.008945	**BRCA1**	672
ENSG00000146872	-0.636159693	0.001817	0.008954	**TLK2**	11011
ENSG00000196358	1.245604042	0.00182	0.008968	**NTNG2**	84628
ENSG00000109861	-0.236668691	0.001827	0.009	**CTSC**	1075
ENSG00000182944	-0.399438268	0.00183	0.009013	**EWSR1**	2130
ENSG00000000460	-0.445137533	0.001832	0.009018	**C1orf112**	55732
ENSG00000213420	2.704373361	0.001833	0.009021	**GPC2**	221914

ENSG00000185262	-0.467137712	0.001835	0.009029	**UBALD2**	283991
ENSG00000125952	0.541226126	0.001836	0.009029	**MAX**	4149
ENSG00000196792	-0.564654218	0.001836	0.009029	**STRN3**	29966
ENSG00000128284	-1.014149927	0.001838	0.009035	**APOL3**	80833
ENSG00000157600	-0.35001163	0.00184	0.009041	**TMEM164**	84187
ENSG00000167088	-0.451176843	0.001841	0.009042	**SNRPD1**	6632
ENSG00000092470	-0.692579083	0.001847	0.009068	**WDR76**	79968
ENSG00000124541	-0.367987214	0.001848	0.009068	**RRP36**	88745
ENSG00000168502	-0.384841275	0.001847	0.009068	**MTCL1**	23255
ENSG00000180875	3.280883878	0.001848	0.009068	**GREM2**	64388
ENSG00000149927	0.741160911	0.00185	0.009071	**DOC2A**	8448
ENSG00000163832	-0.776714979	0.00185	0.009071	**ELP6**	54859
ENSG00000173473	-0.342175861	0.001851	0.009071	**SMARCC1**	6599
ENSG00000105088	0.802915641	0.001852	0.009073	**OLFM2**	93145
ENSG00000108588	0.289090708	0.001852	0.009073	**CCDC47**	57003
ENSG00000214517	0.514964723	0.001853	0.009077	**PPME1**	51400
ENSG00000167771	2.022363782	0.001858	0.009097	**RCOR2**	283248
ENSG00000186088	-0.612003547	0.001859	0.009099	**GSAP**	54103
ENSG00000132361	-0.209646134	0.001861	0.009103	**CLUH**	23277
ENSG00000171862	0.362292777	0.001861	0.009103	**PTEN**	5728
ENSG00000138688	0.260759179	0.001865	0.00912	**KIAA1109**	84162
ENSG00000172432	-0.361163799	0.001867	0.009125	**GTPBP2**	54676
ENSG00000135643	1.394811148	0.001868	0.009128	**KCNMB4**	27345
ENSG00000139620	-0.372403408	0.00187	0.009135	**KANSL2**	54934
ENSG00000140382	-0.36640729	0.001874	0.009155	**HMG20A**	10363
ENSG00000085274	-0.525514998	0.001875	0.009156	**MYNN**	55892
ENSG00000090339	0.506191935	0.001878	0.009165	**ICAM1**	3383
ENSG00000142871	1.175271333	0.00188	0.009172	**CCN1**	3491
ENSG00000066654	-0.680979236	0.001881	0.009176	**THUMPD1**	55623
ENSG00000116754	-0.401893497	0.001883	0.009176	**SRSF11**	9295
ENSG00000134330	-0.513101876	0.001883	0.009176	**IAH1**	285148
ENSG00000198018	0.953614241	0.001882	0.009176	**ENTPD7**	57089
ENSG00000069812	1.518695233	0.001884	0.009178	**HES2**	54626
ENSG00000177453	2.454289214	0.001885	0.009179	**NIM1K**	167359
ENSG00000274265	-1.129244523	0.001885	0.009179	**NA**	NA
ENSG00000121104	1.785481414	0.001886	0.00918	**FAM117A**	81558
ENSG00000117525	1.472283823	0.001888	0.009187	**F3**	2152
ENSG00000106012	0.897795151	0.001891	0.009197	**IQCE**	23288
ENSG00000135744	2.654625208	0.001892	0.009203	**AGT**	183
ENSG00000100227	-0.60681264	0.001895	0.009205	**POLDIP3**	84271
ENSG00000115841	-0.952441722	0.001894	0.009205	**RMDN2**	151393
ENSG00000181090	-0.321946151	0.001894	0.009205	**EHMT1**	79813
ENSG00000181191	0.445901345	0.001896	0.009205	**PJA1**	64219
ENSG00000197261	-0.851079079	0.001896	0.009205	**C6orf141**	135398
ENSG00000143256	-0.31815229	0.001897	0.009208	**PFDN2**	5202
ENSG00000145354	-0.561405659	0.001899	0.009216	**CISD2**	493856
ENSG00000155858	-0.627515551	0.001906	0.009244	**LSM11**	134353
ENSG00000170004	-0.353039626	0.001906	0.009244	**CHD3**	1107
ENSG00000128641	0.271250689	0.001907	0.009247	**MYO1B**	4430
ENSG00000185950	0.662319151	0.001908	0.009247	**IRS2**	8660

ENSG00000183196	1.903626256	0.001912	0.009262	**CHST6**	4166
ENSG00000144061	1.232270647	0.001917	0.009288	**NPHP1**	4867
ENSG00000100626	0.923360252	0.001927	0.009331	**GALNT16**	57452
ENSG00000197965	0.565207181	0.001928	0.009331	**MPZL1**	9019
ENSG00000167740	-0.612119084	0.001929	0.009337	**CYB5D2**	124936
ENSG00000092621	-0.320577467	0.001931	0.009342	**PHGDH**	26227
ENSG00000117632	-0.289383485	0.001932	0.009346	**STMN1**	3925
ENSG00000069998	-0.424286463	0.001937	0.009364	**HDHD5**	27440
ENSG00000116198	0.614857249	0.001942	0.009386	**CEP104**	9731
ENSG00000066136	-0.746470461	0.001944	0.00939	**NFYC**	4802
ENSG00000125843	-1.291267238	0.001943	0.00939	**AP5S1**	55317
ENSG00000132382	-0.357574312	0.001945	0.009391	**MYBBP1A**	10514
ENSG00000172239	-0.327227263	0.001952	0.009422	**PAIP1**	10605
ENSG00000215883	0.577986663	0.001952	0.009423	**CYB5RL**	606495
ENSG00000023445	1.272169103	0.001953	0.009425	**BIRC3**	330
ENSG00000165916	-0.388200156	0.001954	0.009426	**PSMC3**	5702
ENSG00000087152	-0.337700458	0.001956	0.009431	**ATXN7L3**	56970
ENSG00000001460	0.775378412	0.001961	0.009448	**STPG1**	90529
ENSG00000138363	-0.364431254	0.00196	0.009448	**ATIC**	471
ENSG00000124479	-1.985921577	0.001961	0.009448	**NDP**	4693
ENSG00000122420	1.466869179	0.001964	0.00945	**PTGFR**	5737
ENSG00000148606	0.373485939	0.001963	0.00945	**POLR3A**	11128
ENSG00000171806	-1.070409471	0.001964	0.00945	**METTL18**	92342
ENSG00000196275	-2.302095076	0.001963	0.00945	**GTF2IRD2**	84163
ENSG00000110497	0.442502999	0.001966	0.009454	**AMBRA1**	55626
ENSG00000232774	1.06446253	0.001966	0.009454	**FLJ22447**	400221
ENSG00000090376	-0.389481061	0.001968	0.009455	**IRAK3**	11213
ENSG00000174749	0.637188586	0.001967	0.009455	**FAM241A**	132720
ENSG00000181852	0.546869814	0.001968	0.009455	**RNF41**	10193
ENSG00000129353	0.561727757	0.001969	0.009457	**SLC44A2**	57153
ENSG00000166949	-0.347922428	0.00197	0.009457	**SMAD3**	4088
ENSG00000120742	0.341495756	0.00197	0.009457	**SERP1**	27230
ENSG00000038219	0.314020658	0.001979	0.009492	**BOD1L1**	259282
ENSG00000142794	0.666837421	0.001979	0.009492	**NBPF3**	84224
ENSG00000196305	-0.283750855	0.001979	0.009492	**IARS1**	3376
ENSG00000180773	-0.555072487	0.001981	0.009496	**SLC36A4**	120103
ENSG00000146411	1.365268725	0.001982	0.009497	**SLC2A12**	154091
ENSG00000163635	-0.562873006	0.001982	0.009497	**ATXN7**	6314
ENSG00000198721	0.281178489	0.001991	0.009536	**ECI2**	10455
ENSG00000024862	0.760097503	0.001992	0.009538	**CCDC28A**	25901
ENSG00000105613	1.727350869	0.001998	0.009567	**MAST1**	22983
ENSG00000135069	-0.310237337	0.001999	0.009568	**PSAT1**	29968
ENSG00000143436	-0.550672939	0.002001	0.009575	**MRPL9**	65005
ENSG00000104765	0.232256501	0.002004	0.009583	**BNIP3L**	665
ENSG00000229373	-1.32811139	0.002011	0.009618	**LINC00452**	643365
ENSG00000117222	0.435057148	0.002018	0.009646	**RBBP5**	5929
ENSG00000115307	-0.336039808	0.002019	0.009647	**AUP1**	550
ENSG00000152804	-0.561566859	0.002019	0.009647	**HHEX**	3087
ENSG00000005156	-0.769825688	0.002021	0.009648	**LIG3**	3980
ENSG00000119986	1.085937073	0.002021	0.009648	**AVPI1**	60370

ENSG00000228709	1.750292425	0.002021	0.009648	**NA**	NA
ENSG00000010818	-0.408798809	0.002025	0.009664	**HIVEP2**	3097
ENSG00000146250	2.660832176	0.002028	0.009672	**PRSS35**	167681
ENSG00000042317	0.905785315	0.002032	0.009675	**SPATA7**	55812
ENSG00000111846	-0.356171052	0.002031	0.009675	**GCNT2**	2651
ENSG00000112029	-0.767984708	0.002032	0.009675	**FBXO5**	26271
ENSG00000141873	0.518828396	0.002033	0.009675	**SLC39A3**	29985
ENSG00000153827	-0.515089589	0.00203	0.009675	**TRIP12**	9320
ENSG00000156876	-0.682808868	0.002034	0.009675	**SASS6**	163786
ENSG00000175854	0.520154656	0.002029	0.009675	**SWI5**	375757
ENSG00000184702	1.938822788	0.002031	0.009675	**SEPTIN5**	5413
ENSG00000197563	0.662438045	0.002033	0.009675	**PIGN**	23556
ENSG00000182405	0.79020658	0.002035	0.009678	**PGBD4**	161779
ENSG00000187583	-1.424172921	0.002039	0.009692	**PLEKHN1**	84069
ENSG00000111581	-0.381731754	0.002041	0.009699	**NUP107**	57122
ENSG00000117748	-0.491057622	0.002044	0.009709	**RPA2**	6118
ENSG00000176783	-0.297412233	0.002044	0.009709	**RUFY1**	80230
ENSG00000185278	-0.760971784	0.002045	0.009709	**ZBTB37**	84614
ENSG00000141562	0.626754337	0.002049	0.009728	**NARF**	26502
ENSG00000213085	-1.198779553	0.002054	0.009747	**CFAP45**	25790
ENSG00000112319	-0.590161506	0.002056	0.009748	**EYA4**	2070
ENSG00000116741	0.829235779	0.002055	0.009748	**RGS2**	5997
ENSG00000128872	1.693873247	0.002056	0.009748	**TMOD2**	29767
ENSG00000150961	0.275371781	0.002055	0.009748	**SEC24D**	9871
ENSG00000168758	-1.534317047	0.002062	0.009771	**SEMA4C**	54910
ENSG00000131263	-0.487249621	0.002071	0.00981	**RLIM**	51132
ENSG00000173914	-0.70656266	0.002074	0.00982	**RBM4B**	83759
ENSG00000178467	-0.58816229	0.002073	0.00982	**P4HTM**	54681
ENSG00000131269	-0.486965301	0.00208	0.009837	**ABCB7**	22
ENSG00000133101	0.94109351	0.002079	0.009837	**CCNA1**	8900
ENSG00000146066	-0.490514444	0.002079	0.009837	**HIGD2A**	192286
ENSG00000183691	-0.526044648	0.002082	0.009848	**NOG**	9241
ENSG00000136536	-0.414523406	0.002085	0.009859	**MARCHF7**	64844
ENSG00000186106	0.945266001	0.002088	0.009867	**ANKRD46**	157567
ENSG00000119929	-0.451217537	0.002089	0.009872	**CUTC**	51076
ENSG00000138376	-0.763950911	0.00209	0.009872	**BARD1**	580
ENSG00000003989	0.460382044	0.002094	0.009875	**SLC7A2**	6542
ENSG00000105639	3.765199144	0.002094	0.009875	**JAK3**	3718
ENSG00000162976	0.819868963	0.002092	0.009875	**SLC66A3**	130814
ENSG00000196954	-0.467969368	0.002094	0.009875	**CASP4**	837
ENSG00000204381	0.772877581	0.002093	0.009875	**LAYN**	143903
ENSG00000126775	-0.471492354	0.002095	0.009876	**ATG14**	22863
ENSG00000274180	1.812890066	0.002098	0.009889	**NATD1**	256302
ENSG00000167646	1.959742789	0.002099	0.009892	**DNAAF3**	352909
ENSG00000213047	-0.701986116	0.002102	0.009901	**DENND1B**	163486
ENSG00000049883	0.415949868	0.002103	0.009906	**PTCD2**	79810
ENSG00000113580	-0.391915586	0.002104	0.009906	**NR3C1**	2908
ENSG00000131094	1.483107196	0.002106	0.009912	**C1QL1**	10882
ENSG00000106723	-0.522918759	0.002108	0.00992	**SPIN1**	10927
ENSG00000123643	0.498664712	0.002115	0.009945	**SLC36A1**	206358

ENSG00000141030	-0.411189629	0.002114	0.009945	**COPS3**	8533
ENSG00000074755	0.294894345	0.002116	0.009948	**ZZEF1**	23140
ENSG00000102781	-0.527712002	0.00212	0.009965	**KATNAL1**	84056
ENSG00000109381	-0.464138662	0.002122	0.00997	**ELF2**	1998
ENSG00000120802	-1.150684108	0.002126	0.009981	**TMPO**	7112
ENSG00000237276	3.052376802	0.002125	0.009981	**NA**	NA
ENSG00000107954	2.657572186	0.002132	0.010008	**NEURL1**	9148
ENSG00000213965	-0.391242669	0.002133	0.010008	**NUDT19**	390916
ENSG00000237172	-1.017189787	0.002133	0.010008	**B3GNT9**	84752
ENSG00000237424	-1.143016717	0.002137	0.010023	**FOXD2-AS1**	84793
ENSG00000177076	1.117953189	0.002138	0.010024	**ACER2**	340485
ENSG00000205133	-0.536048162	0.002139	0.010028	**TRIQK**	286144
ENSG00000152520	-0.750549442	0.002147	0.010059	**PAN3**	255967
ENSG00000139263	-0.59704861	0.002148	0.01006	**LRIG3**	121227
ENSG00000182979	-0.406251579	0.00215	0.010068	**MTA1**	9112
ENSG00000026103	-1.017417076	0.002154	0.010082	**FAS**	355
ENSG00000167600	1.802012949	0.002154	0.010082	**CYP2S1**	29785
ENSG00000145919	-0.42466636	0.002159	0.010101	**BOD1**	91272
ENSG00000165494	-0.505081298	0.002165	0.010129	**PCF11**	51585
ENSG00000120253	0.425041097	0.00217	0.010142	**NUP43**	348995
ENSG00000144218	-0.556198002	0.00217	0.010142	**AFF3**	3899
ENSG00000267249	2.028162652	0.00217	0.010142	**NA**	NA
ENSG00000066135	0.369627418	0.002172	0.010148	**KDM4A**	9682
ENSG00000138686	0.772308	0.002175	0.010161	**BBS7**	55212
ENSG00000154127	0.384043042	0.00218	0.01018	**UBASH3B**	84959
ENSG00000085999	-0.498294783	0.002194	0.010237	**RAD54L**	8438
ENSG00000168237	-1.149394413	0.002194	0.010237	**GLYCTK**	132158
ENSG00000185245	1.021856581	0.002194	0.010237	**GP1BA**	2811
ENSG00000060982	-0.477970746	0.002201	0.010266	**BCAT1**	586
ENSG00000116771	-0.801813819	0.002202	0.010269	**AGMAT**	79814
ENSG00000117594	2.21499542	0.002208	0.010278	**HSD11B1**	3290
ENSG00000140577	-0.42471636	0.002208	0.010278	**CRTC3**	64784
ENSG00000155792	1.179850312	0.002208	0.010278	**DEPTOR**	64798
ENSG00000177192	-0.450267624	0.002206	0.010278	**PUS1**	80324
ENSG00000197943	1.731040202	0.002205	0.010278	**PLCG2**	5336
ENSG00000224578	-0.473331992	0.002206	0.010278	**NA**	NA
ENSG00000186591	0.689137815	0.002211	0.010289	**UBE2H**	7328
ENSG00000126583	2.16236118	0.002212	0.010289	**PRKCG**	5582
ENSG00000137936	-1.618319919	0.002216	0.010304	**BCAR3**	8412
ENSG00000137936	-1.618319919	0.002216	0.010304	**MIG7**	723788
ENSG00000129422	1.146365089	0.002217	0.010307	**MTUS1**	57509
ENSG00000141504	0.491179015	0.002218	0.010307	**SAT2**	112483
ENSG00000172123	0.409627798	0.002223	0.01033	**SLFN12**	55106
ENSG00000100345	0.235146539	0.002225	0.01033	**MYH9**	4627
ENSG00000167526	-0.239908841	0.002224	0.01033	**RPL13**	6137
ENSG00000198576	1.666614172	0.002225	0.01033	**ARC**	23237
ENSG00000133704	-0.395351592	0.002227	0.010337	**IPO8**	10526
ENSG00000112367	0.475695629	0.002229	0.010342	**FIG4**	9896
ENSG00000196730	-0.424612352	0.00223	0.010344	**DAPK1**	1612
ENSG00000146802	-0.648205004	0.002233	0.010354	**TMEM168**	64418

ENSG00000175175	1.386606217	0.002235	0.010359	**PPM1E**	22843
ENSG00000113569	-0.464178488	0.002238	0.010367	**NUP155**	9631
ENSG00000124702	-0.371828748	0.002237	0.010367	**KLHDC3**	116138
ENSG00000167323	0.503198389	0.002242	0.010382	**STIM1**	6786
ENSG00000070770	0.267321535	0.002246	0.010391	**CSNK2A2**	1459
ENSG00000123689	-0.215159311	0.002245	0.010391	**G0S2**	50486
ENSG00000177058	0.444781847	0.002245	0.010391	**SLC38A9**	153129
ENSG00000122390	-0.391742312	0.002247	0.010396	**NAA60**	79903
ENSG00000056998	0.893066474	0.002249	0.010397	**GYG2**	8908
ENSG00000122642	-0.434084087	0.002248	0.010397	**FKBP9**	11328
ENSG00000172340	-0.414232098	0.002249	0.010397	**SUCLG2**	8801
ENSG00000168003	-0.277900679	0.002252	0.010407	**SLC3A2**	6520
ENSG00000096654	0.617993185	0.002263	0.010454	**ZNF184**	7738
ENSG00000123411	-1.034759612	0.002267	0.010469	**IKZF4**	64375
ENSG00000259803	2.38159089	0.002268	0.010469	**SLC22A31**	146429
ENSG00000069974	-0.501846555	0.002276	0.010494	**RAB27A**	5873
ENSG00000132970	0.618775668	0.002275	0.010494	**WASF3**	10810
ENSG00000141577	0.865792765	0.002275	0.010494	**CEP131**	22994
ENSG00000184916	1.692637211	0.002274	0.010494	**JAG2**	3714
ENSG00000102287	0.847215434	0.002277	0.010497	**GABRE**	2564
ENSG00000117308	-0.373922218	0.002281	0.010511	**GALE**	2582
ENSG00000085982	-0.434025421	0.002282	0.010515	**USP40**	55230
ENSG00000125676	-0.377918433	0.002291	0.010554	**THOC2**	57187
ENSG00000183248	1.678987558	0.002295	0.010566	**PRR36**	80164
ENSG00000175906	0.907542812	0.002296	0.010568	**ARL4D**	379
ENSG00000115902	-0.580457429	0.0023	0.010583	**SLC1A4**	6509
ENSG00000267475	1.492536043	0.002302	0.010593	**NA**	NA
ENSG00000275052	-0.41232184	0.002305	0.010602	**PPP4R3B**	57223
ENSG00000198586	-0.820377832	0.00231	0.010621	**TLK1**	9874
ENSG00000115526	-0.582132302	0.002315	0.010641	**CHST10**	9486
ENSG00000119138	0.675044147	0.002317	0.010646	**KLF9**	687
ENSG00000228716	-0.296003799	0.002319	0.010654	**DHFR**	1719
ENSG00000180964	-0.594918769	0.002324	0.010673	**TCEAL8**	90843
ENSG00000163430	-0.300490825	0.002325	0.010674	**FSTL1**	11167
ENSG00000173894	0.443538412	0.002332	0.010706	**CBX2**	84733
ENSG00000091157	0.485881061	0.002344	0.010756	**WDR7**	23335
ENSG00000143319	0.452280463	0.002346	0.010762	**ISG20L2**	81875
ENSG00000108349	-0.360226681	0.002348	0.010766	**CASC3**	22794
ENSG00000119711	0.618041724	0.002348	0.010766	**ALDH6A1**	4329
ENSG00000253746	-1.018763646	0.002352	0.010779	**NA**	NA
ENSG00000169696	-0.763092381	0.002354	0.010786	**ASPSCR1**	79058
ENSG00000100271	1.314376156	0.002355	0.010788	**TTLL1**	25809
ENSG00000100029	-0.317442031	0.002356	0.01079	**PES1**	23481
ENSG00000116560	-0.302214403	0.002358	0.010794	**SFPQ**	6421
ENSG00000051825	-0.54121508	0.002363	0.010816	**MPHOSPH9**	10198
ENSG00000115271	0.937333607	0.002366	0.010821	**GCA**	25801
ENSG00000146676	0.233621095	0.002365	0.010821	**PURB**	5814
ENSG00000213753	-0.518789832	0.002366	0.010821	**CENPBD1P1**	65996
ENSG00000130332	-0.405904686	0.00237	0.010836	**LSM7**	51690
ENSG00000068885	0.597489463	0.002372	0.01084	**IFT80**	57560

ENSG00000275216	0.58569614	0.002373	0.010845	**NA**	NA
ENSG00000136270	-0.336382643	0.002379	0.010868	**TBRG4**	9238
ENSG00000154719	-0.399809704	0.002381	0.010872	**MRPL39**	54148
ENSG00000063177	-0.163247513	0.002385	0.010887	**RPL18**	6141
ENSG00000101558	-0.354636942	0.002387	0.010895	**VAPA**	9218
ENSG00000107745	0.317631111	0.002388	0.010896	**MICU1**	10367
ENSG00000128274	1.218641304	0.002389	0.010897	**A4GALT**	53947
ENSG00000259330	0.610601725	0.00239	0.0109	**INAFM2**	1.01E+08
ENSG00000168495	-0.50756067	0.002393	0.010908	**POLR3D**	661
ENSG00000046604	-0.301989745	0.002403	0.010952	**DSG2**	1829
ENSG00000271020	-1.615969936	0.002405	0.010957	**NA**	NA
ENSG00000030582	0.347670589	0.002408	0.010968	**GRN**	2896
ENSG00000162981	1.369358409	0.002412	0.010984	**LRATD1**	151354
ENSG00000197415	-0.642645849	0.002414	0.010988	**VEPH1**	79674
ENSG00000172137	0.606770718	0.002416	0.010994	**CALB2**	794
ENSG00000067955	-0.617557575	0.002421	0.011003	**CBFB**	865
ENSG00000104368	1.380067547	0.002419	0.011003	**PLAT**	5327
ENSG00000139372	-0.521482541	0.002419	0.011003	**TDG**	6996
ENSG00000140443	-0.287622443	0.002421	0.011003	**IGF1R**	3480
ENSG00000171206	-1.429273322	0.002421	0.011003	**TRIM8**	81603
ENSG00000102103	-0.295414567	0.002423	0.011008	**PQBP1**	10084
ENSG00000123843	-0.686612173	0.002438	0.01107	**C4BPB**	725
ENSG00000181019	-0.380730595	0.002437	0.01107	**NQO1**	1728
ENSG00000196365	-0.256703954	0.002439	0.011072	**LONP1**	9361
ENSG00000143799	-0.251344555	0.002442	0.011082	**PARP1**	142
ENSG00000136816	0.462833614	0.00245	0.011113	**TOR1B**	27348
ENSG00000273015	-0.894982713	0.002454	0.01113	**NA**	NA
ENSG00000125629	0.554988966	0.002456	0.011134	**INSIG2**	51141
ENSG00000180198	-0.519010992	0.002458	0.011144	**RCC1**	1104
ENSG00000185133	1.70445915	0.002462	0.011152	**INPP5J**	27124
ENSG00000232956	-0.518986398	0.002462	0.011152	**SNHG15**	285958
ENSG00000028310	-0.583083647	0.002471	0.011191	**BRD9**	65980
ENSG00000143363	0.483548838	0.002477	0.011215	**PRUNE1**	58497
ENSG00000167280	0.726371861	0.002479	0.011222	**ENGASE**	64772
ENSG00000135905	-0.730548793	0.00248	0.011222	**DOCK10**	55619
ENSG00000132128	-0.769423091	0.002484	0.011236	**LRRC41**	10489
ENSG00000154832	0.397579122	0.002487	0.01125	**CXXC1**	30827
ENSG00000116001	-0.445544003	0.00249	0.011258	**TIA1**	7072
ENSG00000196850	0.382156605	0.002494	0.011272	**PPTC7**	160760
ENSG00000103342	-0.236644708	0.00251	0.01134	**GSPT1**	2935
ENSG00000231991	0.407948364	0.002514	0.011359	**NA**	NA
ENSG00000073803	0.492017256	0.002515	0.011359	**MAP3K13**	9175
ENSG00000176208	-0.558677872	0.002518	0.011368	**ATAD5**	79915
ENSG00000141027	-0.316120041	0.002519	0.011372	**NCOR1**	9611
ENSG00000067066	-0.321751749	0.002522	0.011382	**SP100**	6672
ENSG00000119906	-0.519825436	0.002533	0.011427	**SLF2**	55719
ENSG00000147654	-0.460486319	0.002536	0.011437	**EBAG9**	9166
ENSG00000088543	0.693472229	0.002541	0.011455	**C3orf18**	51161
ENSG00000198498	-0.755145075	0.002541	0.011455	**TMA16**	55319
ENSG00000104517	-0.347941866	0.002544	0.011461	**UBR5**	51366

ENSG00000226803	-2.79797879	0.002544	0.011461	**NA**	NA
ENSG00000127511	0.301085492	0.002549	0.011479	**SIN3B**	23309
ENSG00000103995	-0.657123031	0.002557	0.011502	**CEP152**	22995
ENSG00000151414	-0.373287129	0.002557	0.011502	**NEK7**	140609
ENSG00000198625	-0.693937281	0.002557	0.011502	**MDM4**	4194
ENSG00000248008	0.929844931	0.002556	0.011502	**NRAV**	1.01E+08
ENSG00000125149	0.635480386	0.002566	0.01154	**C16orf70**	80262
ENSG00000167535	1.126902119	0.002569	0.01155	**CACNB3**	784
ENSG00000169288	-0.437854849	0.002572	0.011558	**MRPL1**	65008
ENSG00000132581	0.547819185	0.002578	0.011582	**SDF2**	6388
ENSG00000130956	0.752683932	0.002579	0.011585	**HABP4**	22927
ENSG00000198399	-0.495228522	0.002581	0.011592	**ITSN2**	50618
ENSG00000130305	-0.479048711	0.002587	0.011613	**NSUN5**	55695
ENSG00000143776	-0.479208998	0.002597	0.011654	**CDC42BPA**	8476
ENSG00000163507	-0.489351706	0.0026	0.011664	**CIP2A**	57650
ENSG00000132002	1.259120011	0.002603	0.011675	**DNAJB1**	3337
ENSG00000080546	0.669765215	0.002605	0.011683	**SESN1**	27244
ENSG00000154642	0.670611034	0.002607	0.011685	**C21orf91**	54149
ENSG00000160712	1.349497505	0.002612	0.011708	**IL6R**	3570
ENSG00000175471	0.471004802	0.002618	0.011728	**MCTP1**	79772
ENSG00000107951	-0.514955697	0.002626	0.011765	**MTPAP**	55149
ENSG00000147650	0.276011599	0.002637	0.011807	**LRP12**	29967
ENSG00000206418	0.321644329	0.002638	0.01181	**RAB12**	201475
ENSG00000125450	-0.2592618	0.002639	0.011811	**NUP85**	79902
ENSG00000115041	-0.655059262	0.002644	0.011828	**KCNIP3**	30818
ENSG00000164109	-0.425715668	0.002649	0.011849	**MAD2L1**	4085
ENSG00000163032	1.532044293	0.002651	0.011856	**VSNL1**	7447
ENSG00000174842	-0.538541592	0.002656	0.011874	**GLMN**	11146
ENSG00000071626	-0.483371489	0.002659	0.011886	**DAZAP1**	26528
ENSG00000198860	-0.295116523	0.002661	0.01189	**TSEN15**	116461
ENSG00000143127	-0.794021763	0.002663	0.011893	**ITGA10**	8515
ENSG00000171492	-0.432148139	0.002666	0.011901	**LRRC8D**	55144
ENSG00000172006	0.934492515	0.002665	0.011901	**ZNF554**	115196
ENSG00000143320	1.656110044	0.002667	0.011903	**CRABP2**	1382
ENSG00000214046	-0.381221046	0.002668	0.011903	**SMIM7**	79086
ENSG00000233016	-0.391661098	0.002669	0.011906	**SNHG7**	84973
ENSG00000160471	2.711793882	0.002674	0.011926	**COX6B2**	125965
ENSG00000112773	0.667430716	0.002678	0.011939	**TENT5A**	55603
ENSG00000070778	-0.437980424	0.002679	0.01194	**PTPN21**	11099
ENSG00000164066	0.512252355	0.00268	0.011941	**INTU**	27152
ENSG00000096093	0.791033128	0.002684	0.011952	**EFHC1**	114327
ENSG00000105821	-0.533000049	0.002685	0.011952	**DNAJC2**	27000
ENSG00000265241	-0.293120726	0.002685	0.011952	**RBM8A**	9939
ENSG00000099800	-0.435118687	0.002691	0.011968	**TIMM13**	26517
ENSG00000100982	-0.575541596	0.00269	0.011968	**PCIF1**	63935
ENSG00000168389	0.807461748	0.00269	0.011968	**MFSD2A**	84879
ENSG00000138600	-0.498919318	0.002693	0.011974	**SPPL2A**	84888
ENSG00000158169	-0.485296986	0.002696	0.011986	**FANCC**	2176
ENSG00000152795	-0.304123056	0.002702	0.01201	**HNRNPDL**	9987
ENSG00000117751	-0.511424177	0.002704	0.012015	**PPP1R8**	5511

ENSG00000010404	0.239305654	0.002706	0.012019	**IDS**	3423
ENSG00000121851	0.510069358	0.002707	0.012021	**POLR3GL**	84265
ENSG00000119927	0.429082025	0.00271	0.01203	**GPAM**	57678
ENSG00000171843	0.435443742	0.00271	0.01203	**MLLT3**	4300
ENSG00000257923	-0.37261897	0.002719	0.012066	**CUX1**	1523
ENSG00000197442	-0.563192851	0.002723	0.012075	**MAP3K5**	4217
ENSG00000249790	4.507101589	0.002723	0.012075	**NA**	NA
ENSG00000259807	-1.724623904	0.002733	0.012115	**NA**	NA
ENSG00000122566	-0.243955346	0.002738	0.012134	**HNRNPA2B**	3181
ENSG00000170365	-1.241793764	0.002741	0.012146	**SMAD1**	4086
ENSG00000114770	-0.37270522	0.002743	0.012151	**ABCC5**	10057
ENSG00000134339	1.142491299	0.002751	0.012182	**SAA2**	6289
ENSG00000175832	-0.431746843	0.002752	0.012182	**ETV4**	2118
ENSG00000029364	0.350126203	0.002753	0.012183	**SLC39A9**	55334
ENSG00000011451	-0.314277735	0.002757	0.012194	**WIZ**	58525
ENSG00000115896	1.519586749	0.002757	0.012194	**PLCL1**	5334
ENSG00000135763	-0.380634374	0.002757	0.012194	**URB2**	9816
ENSG00000088298	0.436059813	0.002759	0.012197	**EDEM2**	55741
ENSG00000088298	0.436059813	0.002759	0.012197	**P24-AS1-ED**	1.11E+08
ENSG00000140521	-0.202948792	0.002761	0.0122	**POLG**	5428
ENSG00000179918	0.279394087	0.002761	0.0122	**SEPHS2**	22928
ENSG00000143375	1.859210624	0.002763	0.012205	**CGN**	57530
ENSG00000161654	-0.53455037	0.002765	0.012208	**LSM12**	124801
ENSG00000186111	0.698161633	0.002765	0.012208	**PIP5K1C**	23396
ENSG00000094804	-0.287400481	0.002767	0.012211	**CDC6**	990
ENSG00000119922	-1.25756278	0.002771	0.012225	**IFIT2**	3433
ENSG00000103494	0.708427774	0.002774	0.012238	**RPGRIP1L**	23322
ENSG00000140564	0.285113569	0.002775	0.012238	**FURIN**	5045
ENSG00000150779	-0.423906763	0.002776	0.012238	**TIMM8B**	26521
ENSG00000169446	0.372668859	0.00278	0.012252	**MMGT1**	93380
ENSG00000139517	0.673027491	0.002787	0.012282	**LNX2**	222484
ENSG00000011260	-0.413682065	0.002793	0.0123	**UTP18**	51096
ENSG00000074800	-0.238064664	0.002794	0.0123	**ENO1**	2023
ENSG00000148400	0.313876885	0.002794	0.0123	**NOTCH1**	4851
ENSG00000197892	0.416294726	0.002794	0.0123	**KIF13B**	23303
ENSG00000161647	-0.7718334	0.0028	0.01232	**MPP3**	4356
ENSG00000064012	-0.402937677	0.002802	0.012327	**CASP8**	841
ENSG00000232445	-0.925932459	0.002803	0.012329	**NA**	NA
ENSG00000140525	-0.4222787	0.002808	0.012344	**FANCI**	55215
ENSG00000063322	0.323751352	0.002811	0.012353	**MED29**	55588
ENSG00000164168	0.425038934	0.002811	0.012353	**TMEM184C**	55751
ENSG00000168890	0.797925977	0.002814	0.012361	**TMEM150A**	129303
ENSG00000062650	-0.354427686	0.002817	0.012374	**WAPL**	23063
ENSG00000108669	0.594490922	0.002819	0.012379	**CYTH1**	9267
ENSG00000027001	0.638129018	0.002822	0.012388	**MIPEP**	4285
ENSG00000148019	-0.29146958	0.002829	0.012413	**CEP78**	84131
ENSG00000101181	-0.328933145	0.002831	0.012413	**MTG2**	26164
ENSG00000136158	-0.509083899	0.00283	0.012413	**SPRY2**	10253
ENSG00000161036	0.64374819	0.002831	0.012413	**LRWD1**	222229
ENSG00000164949	0.822902952	0.002834	0.012422	**GEM**	2669

ENSG00000022267	0.537441461	0.002839	0.012432	**FHL1**	2273
ENSG00000113196	2.940415864	0.002839	0.012432	**HAND1**	9421
ENSG00000175354	-0.388920688	0.002837	0.012432	**PTPN2**	5771
ENSG00000078403	-0.425183002	0.002846	0.012461	**MLLT10**	8028
ENSG00000141480	0.760736243	0.002847	0.012461	**ARRB2**	409
ENSG00000155621	0.603834691	0.00285	0.012472	**C9orf85**	138241
ENSG00000196220	2.511981573	0.002852	0.012477	**SRGAP3**	9901
ENSG00000084234	0.314424117	0.002853	0.012478	**APLP2**	334
ENSG00000012660	0.226771322	0.002857	0.012488	**ELOVL5**	60481
ENSG00000107731	2.241580082	0.002858	0.012488	**UNC5B**	219699
ENSG00000140350	-0.393539822	0.002857	0.012488	**ANP32A**	8125
ENSG00000164172	0.342637926	0.002858	0.012488	**MOCS2**	4338
ENSG00000068137	0.54891092	0.00286	0.012489	**PLEKHH3**	79990
ENSG00000149115	0.478077274	0.00286	0.012489	**TNKS1BP1**	85456
ENSG00000144959	0.232761754	0.002861	0.012491	**NCEH1**	57552
ENSG00000001461	0.495814171	0.002862	0.012492	**NIPAL3**	57185
ENSG00000125968	0.51934083	0.002867	0.012508	**ID1**	3397
ENSG00000125821	-0.351634425	0.002877	0.012545	**DTD1**	92675
ENSG00000271605	-0.694244315	0.002876	0.012545	**MILR1**	284021
ENSG00000104412	0.481817584	0.002878	0.012545	**EMC2**	9694
ENSG00000115207	-0.519548101	0.002879	0.012546	**GTF3C2**	2976
ENSG00000141854	1.276308712	0.002879	0.012546	**MISP3**	113230
ENSG00000053524	1.736856668	0.002881	0.012547	**MCF2L2**	23101
ENSG00000135090	0.230547314	0.002883	0.012553	**TAOK3**	51347
ENSG00000132031	0.796386855	0.002884	0.012554	**MATN3**	4148
ENSG00000151116	0.373923965	0.002885	0.012554	**UEVLD**	55293
ENSG00000162433	-0.66230787	0.002885	0.012554	**AK4**	205
ENSG00000155508	0.298042313	0.002888	0.012564	**CNOT8**	9337
ENSG00000092020	-0.350175082	0.00289	0.012569	**PPP2R3C**	55012
ENSG00000171984	-1.688555926	0.002893	0.012577	**SHLD1**	149840
ENSG00000128607	0.506386151	0.002897	0.012586	**KLHDC10**	23008
ENSG00000130513	1.516043193	0.002896	0.012586	**GDF15**	9518
ENSG00000109971	-0.304893499	0.002898	0.01259	**HSPA8**	3312
ENSG00000108406	0.34268381	0.002904	0.012603	**DHX40**	79665
ENSG00000138621	-0.855514275	0.002902	0.012603	**PPCDC**	60490
ENSG00000198756	-0.646877271	0.002903	0.012603	**COLGALT2**	23127
ENSG00000131944	-1.138991476	0.002908	0.012616	**FAAP24**	91442
ENSG00000178537	0.692738325	0.002914	0.012641	**SLC25A20**	788
ENSG00000169567	-0.31009944	0.002916	0.012645	**HINT1**	3094
ENSG00000275395	0.960679409	0.002917	0.012646	**FCGBP**	8857
ENSG00000135974	0.446477637	0.002925	0.012678	**C2orf49**	79074
ENSG00000119599	0.40017506	0.002927	0.012685	**DCAF4**	26094
ENSG00000160201	-1.825160135	0.00293	0.012688	**U2AF1**	7307
ENSG00000173465	0.502258285	0.002929	0.012688	**ZNRD2**	10534
ENSG00000089280	-0.322858791	0.002932	0.012694	**FUS**	2521
ENSG00000185252	0.632089153	0.002934	0.012701	**ZNF74**	7625
ENSG00000182093	0.414078099	0.002936	0.012707	**GET1**	7485
ENSG00000127527	0.46067336	0.002938	0.012709	**EPS15L1**	58513
ENSG00000175581	-0.474764254	0.002939	0.012711	**MRPL48**	51642
ENSG00000142082	0.597829295	0.002941	0.012715	**SIRT3**	23410

ENSG00000091527	-0.267937675	0.002942	0.012716	**CDV3**	55573
ENSG00000145491	2.007663362	0.002943	0.012718	**ROPN1L**	83853
ENSG00000182220	0.375133327	0.00295	0.012744	**ATP6AP2**	10159
ENSG00000196139	0.971910114	0.002952	0.012752	**AKR1C3**	8644
ENSG00000104142	0.502780366	0.002957	0.01277	**VPS18**	57617
ENSG00000151151	0.697954047	0.002958	0.01277	**IPMK**	253430
ENSG00000138160	-0.451653937	0.002961	0.012771	**KIF11**	3832
ENSG00000156521	-0.614260464	0.00296	0.012771	**TYSND1**	219743
ENSG00000204382	-4.015341076	0.00296	0.012771	**XAGE1B**	653067
ENSG00000101997	-0.643697249	0.002962	0.012772	**CCDC22**	28952
ENSG00000168785	0.337997546	0.002966	0.012788	**TSPAN5**	10098
ENSG00000128989	-0.311607749	0.00297	0.012798	**ARPP19**	10776
ENSG00000123572	-0.655692011	0.002972	0.012804	**NRK**	203447
ENSG00000163755	0.409041378	0.002977	0.012821	**HPS3**	84343
ENSG00000172197	-0.803370482	0.002977	0.012821	**MBOAT1**	154141
ENSG00000136877	-0.358169096	0.002982	0.012839	**FPGS**	2356
ENSG00000117625	-0.378564631	0.002993	0.012874	**RCOR3**	55758
ENSG00000138756	0.468776326	0.002992	0.012874	**BMP2K**	55589
ENSG00000261786	0.392085621	0.002992	0.012874	**NA**	NA
ENSG00000129083	0.224611175	0.002998	0.012893	**COPB1**	1315
ENSG00000145687	1.378744804	0.003002	0.012907	**SSBP2**	23635
ENSG00000164379	-0.613300341	0.003004	0.012909	**FOXQ1**	94234
ENSG00000166889	-0.443715535	0.003004	0.012909	**PATL1**	219988
ENSG00000156671	0.351489903	0.003009	0.012921	**SAMD8**	142891
ENSG00000215182	-1.514881173	0.003008	0.012921	**MUC5AC**	4586
ENSG00000130429	-1.195872038	0.003011	0.012927	**ARPC1B**	10095
ENSG00000144741	-0.666231492	0.003016	0.012947	**SLC25A26**	115286
ENSG00000142864	-0.333093903	0.003027	0.01299	**SERBP1**	26135
ENSG00000075651	0.518207649	0.00303	0.012999	**PLD1**	5337
ENSG00000125814	0.448577871	0.003031	0.013001	**NAPB**	63908
ENSG00000133134	0.981164159	0.003035	0.013013	**BEX2**	84707
ENSG00000155097	0.43773045	0.003036	0.013013	**ATP6V1C1**	528
ENSG00000124164	-0.291702589	0.003038	0.013017	**VAPB**	9217
ENSG00000161204	0.335465059	0.003037	0.013017	**ABCF3**	55324
ENSG00000105176	-0.418947057	0.00304	0.013019	**URI1**	8725
ENSG00000205356	0.72149463	0.00304	0.013019	**TECPR1**	25851
ENSG00000162104	0.39113186	0.003053	0.01307	**ADCY9**	115
ENSG00000119977	0.479814117	0.003057	0.013081	**TCTN3**	26123
ENSG00000172071	0.565726652	0.003058	0.013081	**EIF2AK3**	9451
ENSG00000179922	-0.957242234	0.003057	0.013081	**ZNF784**	147808
ENSG00000137310	-0.608325434	0.00306	0.013083	**TCF19**	6941
ENSG00000167566	0.659699075	0.003059	0.013083	**NCKAP5L**	57701
ENSG00000010244	-0.445370261	0.003064	0.013094	**ZNF207**	7756
ENSG00000101391	0.318593402	0.003067	0.013104	**CDK5RAP1**	51654
ENSG00000177096	-3.727903843	0.003067	0.013104	**PHETA2**	150368
ENSG00000110031	-0.691322597	0.00307	0.013111	**LPXN**	9404
ENSG00000137691	1.14304779	0.003072	0.013117	**CFAP300**	85016
ENSG00000261253	-1.116594711	0.003076	0.013129	**C1002870**	1E+08
ENSG00000116957	0.316430685	0.003077	0.01313	**NA**	NA
ENSG00000008282	-0.328112546	0.003078	0.013133	**SYPL1**	6856

ENSG00000124570	-0.280371672	0.003079	0.013133	**SERPINB6**	5269
ENSG00000119707	-0.420963975	0.003081	0.013136	**RBM25**	58517
ENSG00000146859	-0.62591954	0.003082	0.013139	**TMEM140**	55281
ENSG00000166326	-0.344877585	0.003084	0.013141	**TRIM44**	54765
ENSG00000175606	0.454065598	0.003085	0.013141	**TMEM70**	54968
ENSG00000130940	1.747678039	0.003089	0.013151	**CASZ1**	54897
ENSG00000136819	0.407797869	0.00309	0.013151	**C9orf78**	51759
ENSG00000143207	-0.362644546	0.00309	0.013151	**COP1**	64326
ENSG00000184924	-0.670070769	0.00309	0.013151	**PTRHD1**	391356
ENSG00000139209	1.396944331	0.003092	0.013157	**SLC38A4**	55089
ENSG00000204934	-1.029342791	0.003096	0.013169	**TP6V0E2-A**	401431
ENSG00000148677	1.169504558	0.003098	0.013176	**ANKRD1**	27063
ENSG00000230733	0.606572911	0.003101	0.013182	**NA**	NA
ENSG00000174840	0.340695531	0.003103	0.013189	**PDE12**	201626
ENSG00000133019	-1.897386849	0.003106	0.013195	**CHRM3**	1131
ENSG00000135241	0.450643678	0.003106	0.013195	**PNPLA8**	50640
ENSG00000165300	-1.026016608	0.003108	0.0132	**SLITRK5**	26050
ENSG00000068078	1.608116229	0.003109	0.013201	**FGFR3**	2261
ENSG00000067182	-0.470534783	0.00311	0.013201	**TNFRSF1A**	7132
ENSG00000147164	-0.332986209	0.003111	0.013202	**SNX12**	29934
ENSG00000005007	-0.29033586	0.003114	0.013209	**UPF1**	5976
ENSG00000181904	-0.280664498	0.003117	0.013218	**C5orf24**	134553
ENSG00000165410	-0.341012292	0.003122	0.013239	**CFL2**	1073
ENSG00000131778	-0.361497752	0.003127	0.013257	**CHD1L**	9557
ENSG00000110696	-0.341282429	0.003128	0.013258	**C11orf58**	10944
ENSG00000091656	-0.729601458	0.003132	0.01327	**ZFHX4**	79776
ENSG00000108946	-0.281417936	0.003146	0.013326	**PRKAR1A**	5573
ENSG00000067221	0.758307638	0.003156	0.013366	**STOML1**	9399
ENSG00000121797	-0.521203859	0.00316	0.013378	**CCRL2**	9034
ENSG00000137814	-0.439698892	0.003162	0.013382	**HAUS2**	55142
ENSG00000122565	-0.431861037	0.003163	0.013385	**CBX3**	11335
ENSG00000164442	1.284908718	0.003165	0.013387	**CITED2**	10370
ENSG00000230590	-0.605427372	0.003167	0.013392	**FTX**	1E+08
ENSG00000130997	3.794123966	0.003175	0.013423	**POLN**	353497
ENSG00000170515	-0.375880939	0.003177	0.013428	**PA2G4**	5036
ENSG00000170144	-0.367579416	0.003181	0.01344	**HNRNPA3**	220988
ENSG00000197747	-0.275475636	0.003187	0.013463	**S100A10**	6281
ENSG00000089335	-0.522090464	0.00319	0.01347	**ZNF302**	55900
ENSG00000095319	-0.292333388	0.00319	0.01347	**NUP188**	23511
ENSG00000182796	0.837873298	0.003192	0.013472	**TMEM198B**	440104
ENSG00000250510	1.710566933	0.003193	0.013472	**GPR162**	27239
ENSG00000167862	-0.56684339	0.003195	0.01348	**MRPL58**	3396
ENSG00000139117	-0.894788138	0.003197	0.013481	**CPNE8**	144402
ENSG00000166741	-1.01822026	0.003197	0.013481	**NNMT**	4837
ENSG00000185219	0.3996545	0.003202	0.013496	**ZNF445**	353274
ENSG00000186787	-1.082449385	0.003206	0.01351	**SPIN2B**	474343
ENSG00000213366	1.899051551	0.003207	0.01351	**GSTM2**	2946
ENSG00000182150	-0.655358732	0.003208	0.013512	**ERCC6L2**	375748
ENSG00000173295	1.344548938	0.003215	0.013537	**FAM86B3P**	286042
ENSG00000141542	0.962766871	0.003218	0.013549	**RAB40B**	10966

ENSG00000130204	-0.262839065	0.003224	0.013566	**TOMM40**	10452
ENSG00000260912	1.369063617	0.003223	0.013566	**NA**	NA
ENSG00000146826	0.593337299	0.003227	0.013574	**MAP11**	55262
ENSG00000119523	0.434904722	0.003228	0.013574	**ALG2**	85365
ENSG00000125834	-0.422754176	0.003232	0.01359	**STK35**	140901
ENSG00000161692	-0.461781413	0.003237	0.013607	**DBF4B**	80174
ENSG00000106992	0.94159003	0.003241	0.013618	**AK1**	203
ENSG00000138639	-0.757656038	0.003242	0.013618	**ARHGAP24**	83478
ENSG00000004799	0.962250964	0.003243	0.013618	**PDK4**	5166
ENSG00000163840	-0.922074763	0.003243	0.013618	**DTX3L**	151636
ENSG00000260852	-1.373106761	0.003247	0.013628	**FBXL19-AS1**	283932
ENSG00000154654	0.68444485	0.003259	0.013678	**NCAM2**	4685
ENSG00000121417	0.486332653	0.003266	0.013701	**ZNF211**	10520
ENSG00000184209	-0.586890601	0.003269	0.013709	**SNRNP35**	11066
ENSG00000115091	-0.334423399	0.003275	0.013731	**ACTR3**	10096
ENSG00000105717	1.044484758	0.003278	0.013739	**PBX4**	80714
ENSG00000204991	0.630147281	0.003278	0.013739	**SPIRE2**	84501
ENSG00000226380	0.680223848	0.00328	0.013742	**NA**	NA
ENSG00000099991	0.348183452	0.003281	0.013742	**CABIN1**	23523
ENSG00000114978	-0.305593259	0.003283	0.013748	**MOB1A**	55233
ENSG00000179523	-0.629761913	0.003284	0.01375	**EIF3J-DT**	645212
ENSG00000092421	1.017120737	0.00329	0.013758	**SEMA6A**	57556
ENSG00000100056	0.491434264	0.003289	0.013758	**ESS2**	8220
ENSG00000148834	-0.31165409	0.003289	0.013758	**GSTO1**	9446
ENSG00000164638	2.02085555	0.003288	0.013758	**SLC29A4**	222962
ENSG00000147548	-0.347238331	0.003291	0.013761	**NSD3**	54904
ENSG00000131697	0.619302328	0.003303	0.013806	**NPHP4**	261734
ENSG00000162302	-0.328609216	0.003304	0.013806	**RPS6KA4**	8986
ENSG00000145604	-0.335056488	0.003307	0.013811	**SKP2**	6502
ENSG00000146963	-0.842514065	0.003307	0.013811	**LUC7L2**	51631
ENSG00000100711	0.433224796	0.003309	0.013815	**ZFYVE21**	79038
ENSG00000124067	0.372272699	0.003328	0.013887	**SLC12A4**	6560
ENSG00000152942	1.153871843	0.003327	0.013887	**RAD17**	5884
ENSG00000213619	-0.297435461	0.003328	0.013887	**NDUFS3**	4722
ENSG00000171466	-0.575314282	0.003335	0.01391	**ZNF562**	54811
ENSG00000185043	0.387801962	0.003336	0.013911	**CIB1**	10519
ENSG00000173846	0.676439351	0.003344	0.013942	**PLK3**	1263
ENSG00000077312	-0.348784662	0.003351	0.013966	**SNRPA**	6626
ENSG00000118200	-0.293283775	0.003352	0.013966	**CAMSAP2**	23271
ENSG00000167601	-0.270249731	0.003356	0.013981	**AXL**	558
ENSG00000107036	0.663747332	0.003357	0.013981	**RIC1**	57589
ENSG00000158195	-0.406023418	0.003359	0.013985	**WASF2**	10163
ENSG00000145147	0.438077935	0.00336	0.013987	**SLIT2**	9353
ENSG00000121653	1.679610297	0.003362	0.01399	**MAPK8IP1**	9479
ENSG00000115233	-0.228251871	0.003367	0.014003	**PSMD14**	10213
ENSG00000152102	-0.36457072	0.003366	0.014003	**FAM168B**	130074
ENSG00000197006	-0.323734173	0.003367	0.014003	**METTL9**	51108
ENSG00000037042	0.543771504	0.00337	0.014008	**TUBG2**	27175
ENSG00000125812	0.666486658	0.003373	0.01402	**GZF1**	64412
ENSG00000102904	1.855232871	0.003375	0.014021	**TSNAXIP1**	55815

ENSG00000184949	1.397770059	0.003375	0.014021	**FAM227A**	646851
ENSG00000181751	-0.435364796	0.003379	0.014033	**C5orf30**	90355
ENSG00000150051	-1.701781892	0.003385	0.014052	**MKX**	283078
ENSG00000168234	-0.736366929	0.003389	0.014065	**TTC39C**	125488
ENSG00000135093	0.560146767	0.00339	0.014066	**USP30**	84749
ENSG00000123358	1.684781318	0.003399	0.0141	**NR4A1**	3164
ENSG00000157613	0.612085237	0.0034	0.014103	**CREB3L1**	90993
ENSG00000107897	-0.347778769	0.003402	0.014106	**ACBD5**	91452
ENSG00000118960	0.901950143	0.003403	0.014106	**HS1BP3**	64342
ENSG00000188976	0.250542312	0.003411	0.014136	**NOC2L**	26155
ENSG00000187840	-0.320369898	0.003413	0.014142	**EIF4EBP1**	1978
ENSG00000135108	-0.313762287	0.003415	0.014144	**FBXO21**	23014
ENSG00000120334	-0.775641086	0.003419	0.014159	**CENPL**	91687
ENSG00000141084	0.677148977	0.003421	0.014161	**RANBP10**	57610
ENSG00000148120	-0.696067967	0.003425	0.014174	**AOPEP**	84909
ENSG00000259802	1.434990886	0.003427	0.014178	**NA**	NA
ENSG00000108861	-0.261719988	0.00343	0.014189	**DUSP3**	1845
ENSG00000136153	-0.438140138	0.003436	0.01421	**LMO7**	4008
ENSG00000032219	0.472909282	0.003441	0.014226	**ARID4A**	5926
ENSG00000100478	-0.649857843	0.003447	0.014235	**AP4S1**	11154
ENSG00000137656	-0.695990622	0.003447	0.014235	**BUD13**	84811
ENSG00000148341	0.482518635	0.003447	0.014235	**SH3GLB2**	56904
ENSG00000156958	0.662115658	0.003447	0.014235	**GALK2**	2585
ENSG00000162298	0.547781206	0.003448	0.014235	**SYVN1**	84447
ENSG00000276672	-0.464138279	0.003448	0.014235	**NA**	NA
ENSG00000075568	-0.419329947	0.003451	0.014243	**TMEM131**	23505
ENSG00000196511	-0.68925745	0.003455	0.014255	**TPK1**	27010
ENSG00000101974	-0.401498367	0.003458	0.014263	**ATP11C**	286410
ENSG00000123836	0.616849882	0.00346	0.014268	**PFKFB2**	5208
ENSG00000183726	0.289079416	0.003465	0.014286	**TMEM50A**	23585
ENSG00000007168	-0.228703481	0.003467	0.014289	**PAFAH1B1**	5048
ENSG00000218510	-0.525805166	0.003468	0.014289	**LINC00339**	29092
ENSG00000130348	-0.588390779	0.003471	0.014298	**QRSL1**	55278
ENSG00000177173	-1.367586164	0.003472	0.014301	**NA**	NA
ENSG00000156853	-0.816057965	0.003473	0.014301	**ZNF689**	115509
ENSG00000169764	-0.303985018	0.003477	0.01431	**UGP2**	7360
ENSG00000173801	1.754678952	0.003477	0.01431	**JUP**	3728
ENSG00000160877	-0.175390268	0.003481	0.014317	**NACC1**	112939
ENSG00000196205	-0.200555934	0.00348	0.014317	**NA**	NA
ENSG00000188986	-0.256319252	0.003491	0.014356	**NELFB**	25920
ENSG00000138356	-0.274216784	0.003494	0.014364	**AOX1**	316
ENSG00000273443	1.789250228	0.003497	0.014372	**NA**	NA
ENSG00000107872	0.608577827	0.003499	0.014377	**FBXL15**	79176
ENSG00000157224	-0.429048768	0.003502	0.014385	**CLDN12**	9069
ENSG00000157224	-0.429048768	0.003502	0.014385	**)C1027238**	1.03E+08
ENSG00000134815	-0.440002054	0.003503	0.014386	**DHX34**	9704
ENSG00000186716	0.375868128	0.003513	0.014422	**BCR**	613
ENSG00000143499	-0.472351375	0.003515	0.014427	**SMYD2**	56950
ENSG00000136279	0.266456292	0.003519	0.014443	**DBNL**	28988
ENSG00000172936	-0.341475339	0.003521	0.014446	**MYD88**	4615

ENSG00000100380	0.253038771	0.003525	0.014458	**ST13**	6767
ENSG00000162714	-0.314278646	0.003527	0.014461	**ZNF496**	84838
ENSG00000182899	-0.198490645	0.003534	0.014489	**RPL35A**	6165
ENSG00000164902	-0.39853832	0.003536	0.014492	**PHAX**	51808
ENSG00000166275	-0.939279964	0.00354	0.014504	**BORCS7**	119032
ENSG00000146833	0.470875361	0.003543	0.014514	**TRIM4**	89122
ENSG00000100485	-0.483249128	0.003547	0.014519	**SOS2**	6655
ENSG00000115355	0.344181051	0.003545	0.014519	**CCDC88A**	55704
ENSG00000121892	-0.359185126	0.003547	0.014519	**PDS5A**	23244
ENSG00000183087	-0.331924598	0.00355	0.014529	**GAS6**	2621
ENSG00000031691	-0.377546079	0.003552	0.014533	**CENPQ**	55166
ENSG00000133895	0.402235481	0.003555	0.014541	**MEN1**	4221
ENSG00000179913	0.986209896	0.003559	0.014551	**B3GNT3**	10331
ENSG00000184205	0.411105349	0.00356	0.014552	**TSPYL2**	64061
ENSG00000065883	-0.459204958	0.003563	0.01456	**CDK13**	8621
ENSG00000170471	0.337594632	0.003564	0.01456	**RALGAPB**	57148
ENSG00000009954	-0.475998666	0.003571	0.014584	**BAZ1B**	9031
ENSG00000129534	-0.51695155	0.003571	0.014584	**MIS18BP1**	55320
ENSG00000105409	1.581883936	0.003577	0.014602	**ATP1A3**	478
ENSG00000126773	-0.380142554	0.003579	0.014602	**PCNX4**	64430
ENSG00000165156	0.451747235	0.003578	0.014602	**ZHX1**	11244
ENSG00000122507	0.849102225	0.003581	0.014607	**BBS9**	27241
ENSG00000146476	0.401318683	0.003582	0.014609	**ARMT1**	79624
ENSG00000143740	0.459557417	0.003586	0.01462	**SNAP47**	116841
ENSG00000167772	0.800847778	0.003587	0.014623	**ANGPTL4**	51129
ENSG00000110651	0.253025735	0.003598	0.014662	**CD81**	975
ENSG00000099381	-0.419187906	0.003602	0.014676	**SETD1A**	9739
ENSG00000180357	-0.292716294	0.003606	0.014688	**ZNF609**	23060
ENSG00000265399	2.533639466	0.003614	0.014717	**NA**	NA
ENSG00000004866	-0.775717904	0.003616	0.01472	**ST7**	7982
ENSG00000004866	-0.775717904	0.003616	0.01472	**ST7-OT3**	93655
ENSG00000172534	-1.38197794	0.003617	0.014722	**HCFC1**	3054
ENSG00000150316	-0.33171724	0.003619	0.014725	**CWC15**	51503
ENSG00000134910	-0.309498347	0.003625	0.014746	**STT3A**	3703
ENSG00000147862	-0.595796597	0.003626	0.014747	**NFIB**	4781
ENSG00000157303	1.241566164	0.003629	0.014754	**SUSD3**	203328
ENSG00000148481	0.439655126	0.00363	0.014754	**MINDY3**	80013
ENSG00000243710	1.627253539	0.00363	0.014754	**CFAP57**	149465
ENSG00000119280	0.334677446	0.003633	0.014762	**C1orf198**	84886
ENSG00000088930	-0.223369345	0.003636	0.014769	**XRN2**	22803
ENSG00000102271	-0.419253056	0.00364	0.014779	**KLHL4**	56062
ENSG00000163960	0.241329405	0.003639	0.014779	**UBXN7**	26043
ENSG00000215421	-0.551903044	0.003642	0.014784	**ZNF407**	55628
ENSG00000228451	1.208978281	0.003644	0.014785	**SDAD1P1**	157489
ENSG00000161526	-0.349261572	0.003654	0.014822	**SAP30BP**	29115
ENSG00000106355	-0.371146281	0.003658	0.014836	**LSM5**	23658
ENSG00000013588	1.513591062	0.00366	0.014838	**GPRC5A**	9052
ENSG00000067829	-0.476025156	0.00366	0.014838	**IDH3G**	3421
ENSG00000126822	-0.384765697	0.003662	0.01484	**PLEKHG3**	26030
ENSG00000041353	-0.517226525	0.003666	0.014846	**RAB27B**	5874

ENSG00000115350	-0.364934028	0.003665	0.014846	**POLE4**	56655
ENSG00000150456	-0.988993845	0.003666	0.014846	**EEF1AKMT1**	221143
ENSG00000081386	0.471447753	0.003668	0.01485	**ZNF510**	22869
ENSG00000187792	0.859866563	0.003674	0.014868	**ZNF70**	7621
ENSG00000073584	-0.418524925	0.00368	0.014888	**SMARCE1**	6605
ENSG00000111726	-0.442160293	0.00368	0.014888	**CMAS**	55907
ENSG00000138378	2.78466379	0.003681	0.014888	**STAT4**	6775
ENSG00000164134	-0.220254671	0.003684	0.014894	**NAA15**	80155
ENSG00000231607	-0.97746596	0.003691	0.014922	**DLEU2**	8847
ENSG00000140400	-0.612893885	0.003695	0.014931	**MAN2C1**	4123
ENSG00000177646	-0.330382691	0.003706	0.014974	**ACAD9**	28976
ENSG00000213062	2.451495321	0.003727	0.015053	**NA**	NA
ENSG00000243701	0.78406549	0.003731	0.015065	**DUBR**	344595
ENSG00000059691	-0.462656225	0.003736	0.015083	**GATB**	5188
ENSG00000084676	0.372021034	0.003739	0.015091	**NCOA1**	8648
ENSG00000103495	-0.538080782	0.003741	0.015091	**MAZ**	4150
ENSG00000136802	-0.229045449	0.00374	0.015091	**LRRC8A**	56262
ENSG00000162426	2.186816225	0.003745	0.015102	**SLC45A1**	50651
ENSG00000071575	-0.608666307	0.003748	0.015114	**TRIB2**	28951
ENSG00000198833	-0.619540814	0.003752	0.015124	**UBE2J1**	51465
ENSG00000083093	-0.454084975	0.003755	0.015133	**PALB2**	79728
ENSG00000116120	-0.28698093	0.003757	0.015136	**FARSB**	10056
ENSG00000173013	1.18277244	0.003759	0.015142	**CCDC96**	257236
ENSG00000104549	0.368766836	0.003762	0.015151	**SQLE**	6713
ENSG00000143751	0.465285793	0.003766	0.015156	**SDE2**	163859
ENSG00000169220	0.94524606	0.003765	0.015156	**RGS14**	10636
ENSG00000147526	0.34596547	0.00377	0.015172	**TACC1**	6867
ENSG00000174428	-1.298345601	0.00378	0.015203	**GTF2IRD2B**	389524
ENSG00000205352	0.454394463	0.003779	0.015203	**PRR13**	54458
ENSG00000182512	0.491669964	0.003781	0.015203	**GLRX5**	51218
ENSG00000100292	1.21731118	0.003792	0.015236	**HMOX1**	3162
ENSG00000140463	0.560825663	0.003792	0.015236	**BBS4**	585
ENSG00000198515	1.266856968	0.003793	0.015236	**CNGA1**	1259
ENSG00000213341	0.311805679	0.003791	0.015236	**CHUK**	1147
ENSG00000117362	-0.359614891	0.003803	0.015272	**APH1A**	51107
ENSG00000136699	-0.432311871	0.003804	0.015272	**SMPD4**	55627
ENSG00000089154	-0.157569671	0.003811	0.01529	**GCN1**	10985
ENSG00000164120	1.739838719	0.00381	0.01529	**HPGD**	3248
ENSG00000197622	-0.346097325	0.003811	0.01529	**CDC42SE1**	56882
ENSG00000125875	0.390867036	0.003814	0.015296	**TBC1D20**	128637
ENSG00000189143	1.461430588	0.003819	0.015315	**CLDN4**	1364
ENSG00000149091	0.334982893	0.003821	0.015316	**DGKZ**	8525
ENSG00000182158	0.501833776	0.003826	0.015335	**CREB3L2**	64764
ENSG00000213139	1.883225823	0.00383	0.015346	**CRYGS**	1427
ENSG00000168283	-0.388248162	0.003832	0.015352	**BMI1**	648
ENSG00000107882	0.602613324	0.003847	0.015406	**SUFU**	51684
ENSG00000197081	0.291736008	0.003848	0.015406	**IGF2R**	3482
ENSG00000237686	1.133279514	0.003849	0.015406	**OC1019297**	1.02E+08
ENSG00000137070	1.301424705	0.003862	0.015455	**IL11RA**	3590
ENSG00000120690	-0.609632901	0.003866	0.015468	**ELF1**	1997

ENSG00000141699	0.345167052	0.003868	0.01547	**RETREG3**	162427
ENSG00000126759	-1.038549762	0.00387	0.015475	**CFP**	5199
ENSG00000128534	-0.381655615	0.003872	0.015475	**LSM8**	51691
ENSG00000132570	-0.823996954	0.003872	0.015475	**PCBD2**	84105
ENSG00000182580	2.635194338	0.003874	0.01548	**EPHB3**	2049
ENSG00000184939	0.403149729	0.003876	0.015484	**ZFP90**	146198
ENSG00000130962	0.500653456	0.003879	0.015488	**PRRG1**	5638
ENSG00000173320	2.005273821	0.003879	0.015488	**STOX2**	56977
ENSG00000181284	0.981402204	0.00388	0.015489	**TMEM102**	284114
ENSG00000198873	0.781491038	0.003882	0.015492	**GRK5**	2869
ENSG00000277117	-1.121102583	0.003883	0.015492	**OC1027239**	1.03E+08
ENSG00000121691	0.384988101	0.003885	0.015495	**CAT**	847
ENSG00000112655	0.322058114	0.003887	0.0155	**PTK7**	5754
ENSG00000009413	-0.403747231	0.00389	0.01551	**REV3L**	5980
ENSG00000115839	-0.39834384	0.0039	0.015544	**RAB3GAP1**	22930
ENSG00000112782	2.495004877	0.003905	0.015557	**CLIC5**	53405
ENSG00000160678	-2.167444497	0.003905	0.015557	**S100A1**	6271
ENSG00000115020	0.355990286	0.003908	0.015566	**PIKFYVE**	200576
ENSG00000147383	0.407317165	0.00391	0.015569	**NSDHL**	50814
ENSG00000108342	-1.281824925	0.003913	0.015575	**CSF3**	1440
ENSG00000077942	-1.400785163	0.003921	0.015603	**FBLN1**	2192
ENSG00000140105	-0.19305624	0.003924	0.015614	**WARS1**	7453
ENSG00000163812	0.420397864	0.003927	0.015616	**ZDHHC3**	51304
ENSG00000178385	0.511048752	0.003926	0.015616	**PLEKHM3**	389072
ENSG00000134317	1.85541337	0.00393	0.015625	**GRHL1**	29841
ENSG00000262655	3.422628326	0.003932	0.015629	**SPON1**	10418
ENSG00000185070	-0.549456957	0.003934	0.015633	**FLRT2**	23768
ENSG00000106571	-0.451111187	0.00394	0.015648	**GLI3**	2737
ENSG00000122958	0.336350561	0.003941	0.015648	**VPS26A**	9559
ENSG00000181827	-0.765663476	0.00394	0.015648	**RFX7**	64864
ENSG00000110768	-0.309879673	0.003943	0.015653	**GTF2H1**	2965
ENSG00000107959	0.250301585	0.003948	0.015668	**PITRM1**	10531
ENSG00000181163	-0.255678302	0.003953	0.015684	**NPM1**	4869
ENSG00000111684	0.786040104	0.003956	0.015685	**LPCAT3**	10162
ENSG00000184897	1.165000057	0.003954	0.015685	**H1-10**	8971
ENSG00000188486	-0.200832147	0.003955	0.015685	**H2AX**	3014
ENSG00000100697	-0.410729008	0.003958	0.015691	**DICER1**	23405
ENSG00000119421	0.392093731	0.003968	0.015723	**NDUFA8**	4702
ENSG00000076201	-0.389010735	0.00397	0.01573	**PTPN23**	25930
ENSG00000105514	1.66235654	0.003974	0.015741	**RAB3D**	9545
ENSG00000164749	-1.824111543	0.003978	0.015753	**HNF4G**	3174
ENSG00000052126	-0.466500527	0.00398	0.015756	**PLEKHA5**	54477
ENSG00000132205	-0.756498972	0.003986	0.015776	**EMILIN2**	84034
ENSG00000160325	0.669301886	0.004003	0.015836	**CACFD1**	11094
ENSG00000261061	-0.739562324	0.004002	0.015836	**NA**	NA
ENSG00000113312	0.316866492	0.004006	0.015843	**TTC1**	7265
ENSG00000121753	2.345935854	0.004009	0.015851	**ADGRB2**	576
ENSG00000154734	-0.439588733	0.004012	0.01586	**ADAMTS1**	9510
ENSG00000255121	0.734722581	0.004015	0.015869	**NA**	NA
ENSG00000109390	-0.387610834	0.00402	0.015886	**NDUFC1**	4717

ENSG00000211456	0.28532065	0.004022	0.015887	**SACM1L**	22908
ENSG00000103544	-0.580344532	0.004028	0.0159	**VPS35L**	57020
ENSG00000113721	1.895664056	0.004026	0.0159	**PDGFRB**	5159
ENSG00000127980	0.729266304	0.004029	0.0159	**PEX1**	5189
ENSG00000269343	-0.634727649	0.004027	0.0159	**ZNF587B**	1E+08
ENSG00000170854	-0.531980745	0.004031	0.015905	**RIOX2**	84864
ENSG00000144647	0.497816915	0.004033	0.015907	**POMGNT2**	84892
ENSG00000115944	0.295225864	0.004034	0.015909	**COX7A2L**	9167
ENSG00000198561	-0.181066681	0.004036	0.015911	**CTNND1**	1500
ENSG00000103769	0.270750174	0.004055	0.015984	**RAB11A**	8766
ENSG00000118523	1.459779313	0.004057	0.015986	**CCN2**	1490
ENSG00000082512	-0.7701595	0.004058	0.015987	**TRAF5**	7188
ENSG00000132485	-0.363423402	0.004073	0.016034	**ZRANB2**	9406
ENSG00000146263	-0.577577088	0.004072	0.016034	**MMS22L**	253714
ENSG00000260822	4.081221977	0.004073	0.016034	**NA**	NA
ENSG00000134222	-0.412281223	0.004075	0.016037	**PSRC1**	84722
ENSG00000104312	0.409094445	0.004078	0.01604	**RIPK2**	8767
ENSG00000105223	0.356936054	0.004077	0.01604	**PLD3**	23646
ENSG00000145817	0.350407882	0.00408	0.016044	**YIPF5**	81555
ENSG00000214113	-0.518676768	0.004085	0.016063	**LYRM4**	57128
ENSG00000105928	-0.446429987	0.004087	0.016065	**GSDME**	1687
ENSG00000185352	1.28575268	0.004088	0.016065	**HS6ST3**	266722
ENSG00000168564	0.390866894	0.004091	0.016071	**CDKN2AIP**	55602
ENSG00000105971	-0.395275766	0.004094	0.016082	**CAV2**	858
ENSG00000115257	1.778933285	0.004096	0.016086	**PCSK4**	54760
ENSG00000065057	0.714379439	0.004098	0.016088	**NTHL1**	4913
ENSG00000154479	1.68498554	0.004108	0.016122	**CCDC173**	129881
ENSG00000129219	0.520029827	0.004109	0.016124	**PLD2**	5338
ENSG00000115677	-0.151218825	0.004114	0.016137	**HDLBP**	3069
ENSG00000165434	0.811179039	0.004116	0.016141	**PGM2L1**	283209
ENSG00000188493	-0.687320605	0.004117	0.016141	**C19orf54**	284325
ENSG00000260604	2.158923276	0.004117	0.016141	**NA**	NA
ENSG00000278970	-0.781041312	0.004123	0.01616	**HEIH**	1.01E+08
ENSG00000162512	0.34634093	0.004127	0.01617	**SDC3**	9672
ENSG00000137714	0.34883944	0.004128	0.016171	**FDX1**	2230
ENSG00000189403	-0.343616595	0.004141	0.016216	**HMGB1**	3146
ENSG00000079263	-0.632572782	0.004144	0.016226	**SP140**	11262
ENSG00000110063	-0.705588675	0.004147	0.016232	**DCPS**	28960
ENSG00000076826	2.544177066	0.004149	0.016239	**CAMSAP3**	57662
ENSG00000113368	-1.594305252	0.004155	0.016255	**LMNB1**	4001
ENSG00000102178	-0.398252855	0.004157	0.016261	**UBL4A**	8266
ENSG00000166707	-1.005304273	0.004158	0.016261	**ZCCHC18**	644353
ENSG00000089818	0.393234734	0.00416	0.016264	**NECAP1**	25977
ENSG00000110047	0.283835191	0.004177	0.016326	**EHD1**	10938
ENSG00000237399	1.207829888	0.004178	0.016326	**PITRM1-AS**	1.01E+08
ENSG00000138777	-0.346921251	0.004181	0.016329	**PPA2**	27068
ENSG00000151729	0.255299305	0.004181	0.016329	**SLC25A4**	291
ENSG00000108604	0.469320907	0.004182	0.016332	**SMARCD2**	6603
ENSG00000105939	-0.528997333	0.004189	0.016351	**ZC3HAV1**	56829
ENSG00000111790	0.402214911	0.004193	0.016366	**FGFR1OP2**	26127

ENSG00000143603	1.105108252	0.004202	0.016397	**KCNN3**	3782
ENSG00000104953	4.36649325	0.004206	0.016408	**TLE6**	79816
ENSG00000088280	0.612201149	0.004209	0.016417	**ASAP3**	55616
ENSG00000126768	-0.385016452	0.004231	0.016493	**TIMM17B**	10245
ENSG00000150054	1.646512558	0.004232	0.016493	**MPP7**	143098
ENSG00000198825	0.707631073	0.004232	0.016493	**INPP5F**	22876
ENSG00000133069	1.153048813	0.004236	0.016504	**TMCC2**	9911
ENSG00000136463	-0.607159185	0.004244	0.016531	**TACO1**	51204
ENSG00000112941	-0.641771235	0.004245	0.016531	**TENT4A**	11044
ENSG00000245848	1.994138148	0.004251	0.016552	**CEBPA**	1050
ENSG00000271857	-1.772686852	0.004257	0.016569	**NA**	NA
ENSG00000156858	-0.398210953	0.00426	0.016577	**PRR14**	78994
ENSG00000117411	-0.384139461	0.004262	0.016582	**B4GALT2**	8704
ENSG00000089163	2.455493804	0.004265	0.016587	**SIRT4**	23409
ENSG00000118515	0.439096056	0.004275	0.016623	**SGK1**	6446
ENSG00000277459	-1.863478142	0.004276	0.016623	**NA**	NA
ENSG00000107443	-0.576208081	0.004281	0.016638	**CCNJ**	54619
ENSG00000196159	0.27120661	0.004282	0.016638	**FAT4**	79633
ENSG00000038358	0.301861907	0.004286	0.016645	**EDC4**	23644
ENSG00000167105	1.016491708	0.004285	0.016645	**TMEM92**	162461
ENSG00000267534	0.865503287	0.004287	0.016645	**S1PR2**	9294
ENSG00000105429	0.464266589	0.004296	0.016678	**MEGF8**	1954
ENSG00000108773	-0.455270157	0.004298	0.016678	**KAT2A**	2648
ENSG00000149948	-1.706350218	0.004298	0.016678	**HMGA2**	8091
ENSG00000254389	1.791437446	0.004304	0.016694	**RHPN1-AS1**	78998
ENSG00000174306	0.222361492	0.004307	0.016703	**ZHX3**	23051
ENSG00000077721	0.211594236	0.00431	0.01671	**UBE2A**	7319
ENSG00000086200	-0.321654594	0.004311	0.01671	**IPO11**	51194
ENSG00000086200	-0.321654594	0.004311	0.01671	**PO11-LRRC7**	1.01E+08
ENSG00000104866	0.372591002	0.004316	0.016728	**PPP1R37**	284352
ENSG00000177731	-0.29498587	0.004321	0.016743	**FLII**	2314
ENSG00000197712	0.397440852	0.004324	0.016748	**FAM114A1**	92689
ENSG00000100416	-0.347186672	0.004326	0.016753	**TRMU**	55687
ENSG00000141380	-0.285457666	0.004332	0.016772	**SS18**	6760
ENSG00000100575	-0.414735053	0.004337	0.016786	**TIMM9**	26520
ENSG00000119686	1.58535439	0.004338	0.016786	**FLVCR2**	55640
ENSG00000133216	0.57031298	0.004339	0.016786	**EPHB2**	2048
ENSG00000234155	1.155862868	0.004348	0.016816	**LINC02535**	1.02E+08
ENSG00000103510	-0.610222439	0.004352	0.016823	**KAT8**	84148
ENSG00000174780	-0.255503077	0.004351	0.016823	**SRP72**	6731
ENSG00000174839	-0.410178155	0.004353	0.016823	**DENND6A**	201627
ENSG00000198722	0.35905501	0.004354	0.016826	**UNC13B**	10497
ENSG00000150753	-0.346160111	0.004359	0.016839	**CCT5**	22948
ENSG00000081923	0.601535909	0.004369	0.016874	**ATP8B1**	5205
ENSG00000122042	0.411727762	0.004371	0.016878	**UBL3**	5412
ENSG00000105516	1.317065239	0.004379	0.0169	**DBP**	1628
ENSG00000167785	0.659502463	0.004379	0.0169	**ZNF558**	148156
ENSG00000218336	0.28042167	0.004387	0.016927	**TENM3**	55714
ENSG00000138182	-0.451447055	0.004398	0.016964	**KIF20B**	9585
ENSG00000033170	-0.331539751	0.0044	0.01697	**FUT8**	2530

ENSG00000109079	0.381516447	0.004402	0.016974	**TNFAIP1**	7126
ENSG00000169951	-0.87300594	0.004405	0.016979	**ZNF764**	92595
ENSG00000177570	0.423587827	0.00441	0.016997	**SAMD12**	401474
ENSG00000197948	0.822384698	0.004413	0.017002	**FCHSD1**	89848
ENSG00000163159	-0.480259579	0.004418	0.017013	**VPS72**	6944
ENSG00000186350	-0.364606781	0.004418	0.017013	**RXRA**	6256
ENSG00000111271	0.433535864	0.00442	0.017018	**ACAD10**	80724
ENSG00000070366	0.419283644	0.004423	0.017021	**SMG6**	23293
ENSG00000196781	0.298465225	0.004422	0.017021	**TLE1**	7088
ENSG00000055044	-0.319736695	0.004435	0.017062	**NOP58**	51602
ENSG00000196498	-0.206166154	0.004446	0.017099	**NCOR2**	9612
ENSG00000178761	-0.522124101	0.004449	0.017109	**FAM219B**	57184
ENSG00000103811	0.83294224	0.004462	0.017156	**CTSH**	1512
ENSG00000231365	-0.78483476	0.004475	0.017201	**WARS2-AS1**	1.02E+08
ENSG00000122861	-1.572612234	0.004479	0.017212	**PLAU**	5328
ENSG00000254986	-0.343433128	0.004484	0.017225	**DPP3**	10072
ENSG00000123240	0.283418519	0.004485	0.017228	**OPTN**	10133
ENSG00000110697	0.296985471	0.00449	0.017242	**PITPNM1**	9600
ENSG00000132294	0.359009661	0.004495	0.017256	**EFR3A**	23167
ENSG00000140854	0.554604442	0.004498	0.017262	**KATNB1**	10300
ENSG00000106546	0.537422018	0.004499	0.017264	**AHR**	196
ENSG00000153898	0.534563546	0.004505	0.017281	**MCOLN2**	255231
ENSG00000072134	0.33678393	0.004507	0.017283	**EPN2**	22905
ENSG00000138101	0.668085338	0.004507	0.017283	**DTNB**	1838
ENSG00000010072	0.380952178	0.004515	0.017306	**SPRTN**	83932
ENSG00000139211	-1.525016617	0.004531	0.017365	**AMIGO2**	347902
ENSG00000136848	0.384810102	0.004533	0.01737	**DAB2IP**	153090
ENSG00000115129	-0.776938941	0.004555	0.01745	**TP53I3**	9540
ENSG00000137962	0.258278476	0.004559	0.017457	**ARHGAP29**	9411
ENSG00000169976	0.27498506	0.004559	0.017457	**SF3B5**	83443
ENSG00000105755	0.65257049	0.004564	0.017471	**ETHE1**	23474
ENSG00000084754	0.263803983	0.004567	0.01748	**HADHA**	3030
ENSG00000114942	-0.246186052	0.004574	0.017498	**EEF1B2**	1933
ENSG00000145725	-0.347422781	0.004574	0.017498	**PPIP5K2**	23262
ENSG00000134851	-0.283395804	0.004576	0.017501	**TMEM165**	55858
ENSG00000099624	0.256576951	0.004579	0.017506	**ATP5F1D**	513
ENSG00000142867	0.561278517	0.004585	0.017525	**BCL10**	8915
ENSG00000188690	0.269418499	0.004587	0.01753	**UROS**	7390
ENSG00000104047	-0.508806788	0.004593	0.017549	**DTWD1**	56986
ENSG00000115268	-0.225650509	0.004602	0.017578	**RPS15**	6209
ENSG00000035687	-0.490563446	0.004607	0.017583	**ADSS2**	159
ENSG00000068878	0.24644451	0.004605	0.017583	**PSME4**	23198
ENSG00000130775	1.403855403	0.004606	0.017583	**THEMIS2**	9473
ENSG00000125247	-0.512912963	0.00461	0.017592	**TMTC4**	84899
ENSG00000175643	-0.437840607	0.004628	0.017657	**RMI2**	116028
ENSG00000115159	-0.55080655	0.004645	0.017718	**GPD2**	2820
ENSG00000163818	0.575428041	0.004648	0.017723	**LZTFL1**	54585
ENSG00000163541	0.297391213	0.004673	0.017814	**SUCLG1**	8802
ENSG00000132386	0.980499596	0.004675	0.017819	**SERPINF1**	5176
ENSG00000132300	-0.192302248	0.00468	0.017833	**PTCD3**	55037

ENSG00000099812	-2.027833883	0.004686	0.017852	**MISP**	126353
ENSG00000143933	0.169741233	0.004687	0.017852	**CALM2**	805
ENSG00000176697	0.644259802	0.004689	0.017856	**BDNF**	627
ENSG00000272686	0.766784465	0.004695	0.017874	**NA**	NA
ENSG00000105483	-0.57165661	0.004704	0.017898	**CARD8**	22900
ENSG00000177830	0.250697644	0.004703	0.017898	**CHID1**	66005
ENSG00000107854	0.217360919	0.00471	0.017915	**TNKS2**	80351
ENSG00000198783	-0.724347614	0.00471	0.017915	**ZNF830**	91603
ENSG00000019485	-1.108809433	0.004713	0.01792	**PRDM11**	56981
ENSG00000111052	2.113665826	0.004717	0.017928	**LIN7A**	8825
ENSG00000169213	1.91459847	0.004717	0.017928	**RAB3B**	5865
ENSG00000264522	0.270065322	0.004724	0.017949	**OTUD7B**	56957
ENSG00000157741	0.457952756	0.004725	0.017951	**UBN2**	254048
ENSG00000163884	0.682714895	0.004735	0.017981	**KLF15**	28999
ENSG00000233276	-0.207263086	0.004741	0.018002	**GPX1**	2876
ENSG00000052841	-0.296074644	0.004751	0.018031	**TTC17**	55761
ENSG00000077157	0.676385192	0.004752	0.018031	**PPP1R12B**	4660
ENSG00000129028	0.801100107	0.00475	0.018031	**THAP10**	56906
ENSG00000064999	0.448451012	0.004755	0.018038	**ANKS1A**	23294
ENSG00000145860	-0.527881592	0.00476	0.018053	**RNF145**	153830
ENSG00000110921	0.392566416	0.004763	0.018058	**MVK**	4598
ENSG00000115423	2.289276202	0.004766	0.018065	**DNAH6**	1768
ENSG00000113719	0.453584018	0.004778	0.018106	**ERGIC1**	57222
ENSG00000112394	0.971038111	0.004784	0.018123	**SLC16A10**	117247
ENSG00000166793	1.616477932	0.004784	0.018123	**YPEL4**	219539
ENSG00000070214	0.258472097	0.00479	0.018135	**SLC44A1**	23446
ENSG00000134463	0.632142478	0.00479	0.018135	**ECHDC3**	79746
ENSG00000110104	-0.450786985	0.004795	0.018144	**CCDC86**	79080
ENSG00000168116	-0.457655815	0.004794	0.018144	**KIAA1586**	57691
ENSG00000084764	2.025623979	0.0048	0.018159	**MAPRE3**	22924
ENSG00000048649	-0.44419905	0.004802	0.018165	**RSF1**	51773
ENSG00000113460	-0.574548871	0.004815	0.018204	**BRIX1**	55299
ENSG00000170949	0.480187629	0.004815	0.018204	**ZNF160**	90338
ENSG00000267405	-1.309584858	0.00482	0.01822	**NA**	NA
ENSG00000144747	0.414950859	0.004825	0.018232	**TMF1**	7110
ENSG00000163399	0.169573803	0.00483	0.018248	**ATP1A1**	476
ENSG00000136982	-0.65707474	0.004833	0.018255	**DSCC1**	79075
ENSG00000164327	-0.376216403	0.004835	0.018258	**RICTOR**	253260
ENSG00000167815	0.299044067	0.00484	0.018274	**PRDX2**	7001
ENSG00000138028	3.565837538	0.004846	0.018284	**CGREF1**	10669
ENSG00000165898	0.397276855	0.004846	0.018284	**ISCA2**	122961
ENSG00000171314	-0.167546629	0.004844	0.018284	**PGAM1**	5223
ENSG00000197121	0.544317811	0.004856	0.018314	**PGAP1**	80055
ENSG00000160209	0.32641381	0.004857	0.018317	**PDXK**	8566
ENSG00000160209	0.32641381	0.004857	0.018317	**)C1053728**	1.05E+08
ENSG00000163590	0.866774996	0.004862	0.018329	**PPM1L**	151742
ENSG00000196510	-0.558625057	0.004863	0.018329	**ANAPC7**	51434
ENSG00000131469	-0.148799318	0.00487	0.018351	**RPL27**	6155
ENSG00000106080	0.717825004	0.004877	0.018374	**FKBP14**	55033
ENSG00000114450	-0.480936569	0.004888	0.01841	**GNB4**	59345

ENSG00000204070	0.370809	0.004891	0.018418	**SYS1**	90196
ENSG00000077348	-0.327263002	0.004894	0.018424	**EXOSC5**	56915
ENSG00000158615	0.25358781	0.004929	0.018549	**PPP1R15B**	84919
ENSG00000165630	0.544458827	0.004929	0.018549	**PRPF18**	8559
ENSG00000158856	-0.975018129	0.004933	0.018558	**DMTN**	2039
ENSG00000107929	-0.270841862	0.004945	0.018595	**LARP4B**	23185
ENSG00000139438	1.0304971	0.004948	0.018595	**FAM222A**	84915
ENSG00000156973	0.586731871	0.004946	0.018595	**PDE6D**	5147
ENSG00000174792	2.517852704	0.004948	0.018595	**ODAPH**	152816
ENSG00000245556	-0.962596214	0.004949	0.018595	**CAMP1-AS**	728769
ENSG00000196453	-0.555206622	0.004954	0.018612	**ZNF777**	27153
ENSG00000213639	-0.330448327	0.004962	0.018636	**PPP1CB**	5500
ENSG00000025770	-0.373506901	0.004974	0.018668	**NCAPH2**	29781
ENSG00000123453	-0.77328136	0.004974	0.018668	**SARDH**	1757
ENSG00000141582	0.427757593	0.004972	0.018668	**CBX4**	8535
ENSG00000175711	-0.713515194	0.004976	0.01867	**B3GNTL1**	146712
ENSG00000166037	-0.383595931	0.004999	0.018754	**CEP57**	9702
ENSG00000058600	-0.605080023	0.005002	0.01876	**POLR3E**	55718
ENSG00000108506	-0.422269633	0.005011	0.018791	**INTS2**	57508
ENSG00000127920	-0.451448355	0.005017	0.018809	**GNG11**	2791
ENSG00000185567	1.566903219	0.005022	0.018823	**AHNAK2**	113146
ENSG00000183671	-0.834043857	0.005026	0.018833	**GPR1**	2825
ENSG00000079335	0.543887563	0.005039	0.018858	**CDC14A**	8556
ENSG00000115762	0.32412875	0.005036	0.018858	**PLEKHB2**	55041
ENSG00000138642	-0.809923832	0.005037	0.018858	**HERC6**	55008
ENSG00000173207	-0.411611653	0.005038	0.018858	**CKS1B**	1163
ENSG00000198931	-0.282524985	0.005037	0.018858	**APRT**	353
ENSG00000101150	-0.186323761	0.005044	0.018875	**TPD52L2**	7165
ENSG00000164010	-0.863524961	0.005046	0.018878	**ERMAP**	114625
ENSG00000182263	-0.707560274	0.005053	0.018894	**FIGN**	55137
ENSG00000197632	1.939825201	0.005054	0.018894	**SERPINB2**	5055
ENSG00000228106	0.831880234	0.005054	0.018894	**NA**	NA
ENSG00000160752	-0.267969659	0.005058	0.018902	**FDPS**	2224
ENSG00000064932	-1.298303729	0.00507	0.018946	**SBNO2**	22904
ENSG00000101901	-0.626575675	0.005078	0.018953	**ALG13**	79868
ENSG00000162885	0.425231074	0.005078	0.018953	**B3GALNT2**	148789
ENSG00000185896	0.162390844	0.005076	0.018953	**LAMP1**	3916
ENSG00000259153	0.647582339	0.005074	0.018953	**)C1005064**	1.01E+08
ENSG00000260686	1.608966191	0.005075	0.018953	**NA**	NA
ENSG00000100036	0.763947202	0.005087	0.018966	**SLC35E4**	339665
ENSG00000104408	-0.315752177	0.005085	0.018966	**EIF3E**	3646
ENSG00000107679	0.44065461	0.005088	0.018966	**PLEKHA1**	59338
ENSG00000112996	-0.357073696	0.005084	0.018966	**MRPS30**	10884
ENSG00000124596	-0.52483937	0.005087	0.018966	**OARD1**	221443
ENSG00000142173	0.292536846	0.005092	0.018976	**COL6A2**	1292
ENSG00000159346	0.294901236	0.005093	0.018976	**ADIPOR1**	51094
ENSG00000188760	1.107490759	0.005094	0.018976	**TMEM198**	130612
ENSG00000111711	-0.365332755	0.005101	0.018998	**GOLT1B**	51026
ENSG00000092199	-0.194391659	0.00511	0.019024	**HNRNPC**	3183
ENSG00000157827	0.306601224	0.00511	0.019024	**FMNL2**	114793

ENSG00000172943	-0.256089203	0.005115	0.019038	**PHF8**	23133
ENSG00000163281	-0.304866578	0.005118	0.019044	**GNPDA2**	132789
ENSG00000186231	2.622764075	0.005123	0.019057	**KLHL32**	114792
ENSG00000160948	-0.303863843	0.005125	0.01906	**VPS28**	51160
ENSG00000118507	-1.224156783	0.005129	0.019066	**AKAP7**	9465
ENSG00000188243	0.436557265	0.005128	0.019066	**COMMD6**	170622
ENSG00000171522	0.744601681	0.005131	0.019068	**PTGER4**	5734
ENSG00000183340	0.390172074	0.005132	0.019068	**JRKL**	8690
ENSG00000100804	-0.313404326	0.005145	0.019109	**PSMB5**	5693
ENSG00000179532	0.647801713	0.005144	0.019109	**DNHD1**	144132
ENSG00000060558	-0.432900009	0.005154	0.019137	**GNA15**	2769
ENSG00000206530	0.644905282	0.005158	0.019148	**CFAP44**	55779
ENSG00000056972	-0.772101055	0.005169	0.01918	**TRAF3IP2**	10758
ENSG00000105137	-1.397653988	0.005168	0.01918	**SYDE1**	85360
ENSG00000170903	0.356533551	0.005174	0.019192	**MSANTD4**	84437
ENSG00000171161	-0.440884279	0.005175	0.019192	**ZNF672**	79894
ENSG00000166439	-0.472092379	0.005181	0.01921	**RNF169**	254225
ENSG00000223768	0.740109605	0.005188	0.019231	**LINC00205**	642852
ENSG00000169371	-0.640507408	0.005196	0.019256	**SNUPN**	10073
ENSG00000228903	3.033275019	0.005199	0.019264	**NA**	NA
ENSG00000221823	-0.286589879	0.005204	0.019276	**PPP3R1**	5534
ENSG00000132394	0.461571041	0.005206	0.019281	**EEFSEC**	60678
ENSG00000126787	-0.329489615	0.005218	0.019321	**DLGAP5**	9787
ENSG00000121989	0.543320617	0.00522	0.019324	**ACVR2A**	92
ENSG00000159322	-0.262088361	0.005222	0.019325	**ADPGK**	83440
ENSG00000010292	-0.288291183	0.005233	0.019361	**NCAPD2**	9918
ENSG00000167645	0.412082666	0.005234	0.019361	**YIF1B**	90522
ENSG00000164236	0.253136798	0.005238	0.019371	**ANKRD33B**	651746
ENSG00000137460	2.057413581	0.005245	0.019395	**FHDC1**	85462
ENSG00000120526	-0.206884483	0.005249	0.0194	**NUDCD1**	84955
ENSG00000121211	-0.597817762	0.005248	0.0194	**MND1**	84057
ENSG00000005812	-0.503694108	0.005262	0.019442	**FBXL3**	26224
ENSG00000272870	0.935498381	0.005273	0.019477	**SAP30-DT**	1.05E+08
ENSG00000164104	-1.193332562	0.005276	0.019487	**HMGB2**	3148
ENSG00000048052	-0.411921335	0.00528	0.019491	**HDAC9**	9734
ENSG00000198855	0.55883084	0.005279	0.019491	**FICD**	11153
ENSG00000111859	1.919591162	0.005283	0.019498	**NEDD9**	4739
ENSG00000130340	-0.543058355	0.005302	0.019555	**SNX9**	51429
ENSG00000133816	0.299834897	0.005301	0.019555	**MICAL2**	9645
ENSG00000133816	0.299834897	0.005301	0.019555	**MICALCL**	84953
ENSG00000198108	-0.758438933	0.0053	0.019555	**CHSY3**	337876
ENSG00000107862	0.443065223	0.005313	0.019591	**GBF1**	8729
ENSG00000177469	0.299866776	0.005316	0.019598	**CAVIN1**	284119
ENSG00000115866	-0.46833628	0.005325	0.019626	**DARS1**	1615
ENSG00000204060	1.593424151	0.005328	0.019633	**FOXO6**	1E+08
ENSG00000100926	-0.814921629	0.005335	0.019655	**TM9SF1**	10548
ENSG00000006062	0.375539232	0.005338	0.019655	**MAP3K14**	9020
ENSG00000140365	-0.396482884	0.005337	0.019655	**COMMD4**	54939
ENSG00000214160	-0.344642224	0.005348	0.019688	**ALG3**	10195
ENSG00000169189	0.41077701	0.005351	0.019694	**NSMCE1**	197370

ENSG00000147364	0.442447089	0.005353	0.019697	**FBXO25**	26260
ENSG00000163875	-0.290937067	0.005356	0.019705	**MEAF6**	64769
ENSG00000151247	-0.295567181	0.005366	0.019735	**EIF4E**	1977
ENSG00000129255	-0.635869561	0.005375	0.019765	**MPDU1**	9526
ENSG00000108515	-0.812339124	0.005382	0.019787	**ENO3**	2027
ENSG00000205208	-0.283409485	0.005386	0.019797	**C4orf46**	201725
ENSG00000129355	0.383622484	0.005388	0.019799	**CDKN2D**	1032
ENSG00000120137	0.189311981	0.005394	0.019817	**PANK3**	79646
ENSG00000281398	-0.523465559	0.005397	0.019823	**NA**	NA
ENSG00000106733	0.864057374	0.005401	0.019834	**NMRK1**	54981
ENSG00000096092	0.608839752	0.005403	0.019836	**TMEM14A**	28978
ENSG00000095739	0.829930538	0.005409	0.019839	**BAMBI**	25805
ENSG00000152117	-0.602903008	0.005406	0.019839	**LOC150776**	150776
ENSG00000162980	-0.416508479	0.005409	0.019839	**ARL5A**	26225
ENSG00000168306	0.767816863	0.005409	0.019839	**ACOX2**	8309
ENSG00000214176	0.478584423	0.005411	0.01984	**NA**	NA
ENSG00000272333	-0.488668221	0.00542	0.019872	**KMT2B**	9757
ENSG00000178700	0.673801851	0.00543	0.019903	**DHFR2**	200895
ENSG00000160233	1.364903697	0.005432	0.019906	**LRRC3**	81543
ENSG00000131652	-0.547875512	0.005434	0.019909	**THOC6**	79228
ENSG00000120896	-0.482092963	0.005436	0.01991	**SORBS3**	10174
ENSG00000141736	-0.407838258	0.005449	0.019954	**ERBB2**	2064
ENSG00000092140	-0.56956576	0.005462	0.019997	**G2E3**	55632
ENSG00000228782	-1.130181647	0.005466	0.020005	**NA**	NA
ENSG00000100360	0.552029776	0.005471	0.020019	**IFT27**	11020
ENSG00000185634	1.922027402	0.005476	0.020035	**SHC4**	399694
ENSG00000137267	0.783836968	0.00548	0.020045	**TUBB2A**	7280
ENSG00000118900	0.238580564	0.005487	0.020064	**UBN1**	29855
ENSG00000182004	-0.410171558	0.005489	0.020069	**SNRPE**	6635
ENSG00000101670	1.989417351	0.005498	0.020096	**LIPG**	9388
ENSG00000101574	-0.685108051	0.005505	0.020118	**METTL4**	64863
ENSG00000126016	2.621370224	0.005512	0.020138	**AMOT**	154796
ENSG00000168385	-0.151298011	0.005531	0.020203	**SEPTIN2**	4735
ENSG00000158716	1.060211764	0.005534	0.020207	**DUSP23**	54935
ENSG00000165105	-0.527150674	0.005535	0.020207	**RASEF**	158158
ENSG00000233621	1.170323344	0.005549	0.020248	**LINC01137**	728431
ENSG00000270696	-0.726843866	0.005548	0.020248	**NA**	NA
ENSG00000113734	-0.806884397	0.005555	0.020266	**BNIP1**	662
ENSG00000064419	-0.327150295	0.005571	0.020319	**TNPO3**	23534
ENSG00000153015	-0.324107043	0.005584	0.020363	**CWC27**	10283
ENSG00000128965	0.72593283	0.005585	0.020364	**CHAC1**	79094
ENSG00000179134	0.347844028	0.005588	0.02037	**SAMD4B**	55095
ENSG00000141519	1.885255563	0.005592	0.020375	**CCDC40**	55036
ENSG00000182118	0.550266045	0.005592	0.020375	**FAM89A**	375061
ENSG00000120149	0.825779103	0.005595	0.020381	**MSX2**	4488
ENSG00000136960	4.087052601	0.005605	0.020411	**ENPP2**	5168
ENSG00000167632	0.750479374	0.005612	0.020435	**TRAPPC9**	83696
ENSG00000258429	-0.856380054	0.005625	0.020475	**PDF**	64146
ENSG00000020256	-0.622295769	0.005631	0.02049	**ZFP64**	55734
ENSG00000103653	-0.224811375	0.005631	0.02049	**CSK**	1445

ENSG00000136240	0.218637511	0.005638	0.020503	**KDELR2**	11014
ENSG00000140553	0.346521248	0.005637	0.020503	**UNC45A**	55898
ENSG00000067596	0.211097445	0.005641	0.02051	**DHX8**	1659
ENSG00000111802	-0.33153338	0.005645	0.020519	**TDP2**	51567
ENSG00000164535	0.43013515	0.005648	0.020527	**DAGLB**	221955
ENSG00000129128	-0.238675817	0.005656	0.020551	**SPCS3**	60559
ENSG00000204842	-0.290728627	0.005658	0.020552	**ATXN2**	6311
ENSG00000171428	0.685647436	0.00567	0.020592	**NAT1**	9
ENSG00000172469	-0.889428955	0.005673	0.0206	**MANEA**	79694
ENSG00000127863	1.843616048	0.005678	0.020614	**TNFRSF19**	55504
ENSG00000086189	-0.304806021	0.00568	0.020614	**DIMT1**	27292
ENSG00000184787	0.248416069	0.005707	0.020708	**UBE2G2**	7327
ENSG00000018610	0.425334413	0.005715	0.020731	**CXorf56**	63932
ENSG00000177380	1.13585699	0.005726	0.020768	**PPFIA3**	8541
ENSG00000106258	-1.43387762	0.00573	0.020774	**CYP3A5**	1577
ENSG00000124782	-0.535470827	0.00573	0.020774	**RREB1**	6239
ENSG00000104147	-0.604592738	0.005732	0.020776	**OIP5**	11339
ENSG00000134245	1.403961582	0.00574	0.020799	**WNT2B**	7482
ENSG00000172345	1.849943962	0.005743	0.020804	**STARD5**	80765
ENSG00000164674	0.780959922	0.005749	0.020823	**SYTL3**	94120
ENSG00000186174	-0.288188046	0.005752	0.020828	**BCL9L**	283149
ENSG00000135540	-1.446359626	0.005758	0.020844	**NHSL1**	57224
ENSG00000166979	-0.802013522	0.00577	0.020881	**EVA1C**	59271
ENSG00000181449	1.541413186	0.00577	0.020881	**SOX2**	6657
ENSG00000125304	0.229851902	0.005772	0.020884	**TM9SF2**	9375
ENSG00000128692	-0.714122015	0.005779	0.020903	**NA**	NA
ENSG00000187790	0.422639791	0.005782	0.020909	**FANCM**	57697
ENSG00000196313	-0.301030878	0.005795	0.02095	**POM121**	9883
ENSG00000123124	0.281773858	0.005798	0.020956	**WWP1**	11059
ENSG00000152439	2.515873841	0.005799	0.020956	**ZNF773**	374928
ENSG00000217241	-0.713428795	0.0058	0.020956	**NA**	NA
ENSG00000134899	-0.916615023	0.005806	0.020972	**ERCC5**	2073
ENSG00000134899	-0.916615023	0.005806	0.020972	**3IVM-ERCC**	1.01E+08
ENSG00000090020	0.340576178	0.005809	0.020977	**SLC9A1**	6548
ENSG00000114446	0.629296095	0.00581	0.020977	**IFT57**	55081
ENSG00000173926	0.836293049	0.005816	0.020995	**MARCHF3**	115123
ENSG00000006712	-0.415675456	0.005823	0.021014	**PAF1**	54623
ENSG00000131100	0.308964642	0.005827	0.021018	**ATP6V1E1**	529
ENSG00000173545	0.600611042	0.005826	0.021018	**ZNF622**	90441
ENSG00000166166	-0.381942278	0.005828	0.021019	**TRMT61A**	115708
ENSG00000131871	0.283259819	0.00584	0.021052	**SELENOS**	55829
ENSG00000144021	0.342089997	0.00584	0.021052	**CIAO1**	9391
ENSG00000114388	0.629709123	0.005862	0.021127	**NPRL2**	10641
ENSG00000072274	-0.230972339	0.005866	0.021136	**TFRC**	7037
ENSG00000196123	0.661628647	0.005871	0.021151	**KIAA0895L**	653319
ENSG00000196372	0.290175907	0.005875	0.021158	**ASB13**	79754
ENSG00000134901	0.684842485	0.005879	0.02117	**POGLUT2**	79070
ENSG00000166598	0.114813826	0.005885	0.021176	**HSP90B1**	7184
ENSG00000168137	-0.332150352	0.005884	0.021176	**SETD5**	55209
ENSG00000174233	0.344634269	0.005885	0.021176	**ADCY6**	112

ENSG00000105355	0.287219753	0.005889	0.021186	**PLIN3**	10226
ENSG00000163947	0.746099627	0.005898	0.021214	**ARHGEF3**	50650
ENSG00000101846	-0.585239976	0.005904	0.021224	**STS**	412
ENSG00000177602	-0.66728866	0.005904	0.021224	**HASPIN**	83903
ENSG00000170542	1.048622908	0.005908	0.021235	**SERPINB9**	5272
ENSG00000131711	-0.270308932	0.005911	0.02124	**MAP1B**	4131
ENSG00000156804	0.581265268	0.005932	0.02131	**FBXO32**	114907
ENSG00000233058	1.970532842	0.005935	0.021316	**NA**	NA
ENSG00000163605	-0.253942633	0.005941	0.021333	**PPP4R2**	151987
ENSG00000233396	1.273418262	0.005942	0.021333	**NA**	NA
ENSG00000164111	0.19194967	0.00595	0.021356	**ANXA5**	308
ENSG00000125384	1.45745616	0.005955	0.021371	**PTGER2**	5732
ENSG00000103047	-0.733863453	0.005963	0.021392	**TANGO6**	79613
ENSG00000263731	-1.261003651	0.005975	0.021432	**NA**	NA
ENSG00000123444	-0.768427828	0.005978	0.021437	**KBTBD4**	55709
ENSG00000139233	0.482042675	0.005987	0.021464	**LLPH**	84298
ENSG00000135314	-0.61173602	0.005991	0.021476	**KHDC1**	80759
ENSG00000132964	0.417888281	0.005995	0.021484	**CDK8**	1024
ENSG00000063854	0.41954884	0.006003	0.02151	**HAGH**	3029
ENSG00000070831	-0.254484636	0.006016	0.02155	**CDC42**	998
ENSG00000150630	-1.401254939	0.006028	0.021582	**VEGFC**	7424
ENSG00000187164	-0.405333933	0.006027	0.021582	**SHTN1**	57698
ENSG00000233117	0.540249628	0.006037	0.02161	**NA**	NA
ENSG00000101464	-0.490519368	0.006039	0.021611	**PIGU**	128869
ENSG00000186318	0.429251118	0.00604	0.021611	**BACE1**	23621
ENSG00000188647	-0.308666089	0.006064	0.021692	**PTAR1**	375743
ENSG00000136261	0.279187766	0.006067	0.021697	**BZW2**	28969
ENSG00000178971	-0.365040856	0.006087	0.021767	**CTC1**	80169
ENSG00000163938	-0.266484404	0.00609	0.021772	**GNL3**	26354
ENSG00000038382	-0.165404701	0.006098	0.021789	**TRIO**	7204
ENSG00000071082	-0.207352343	0.006097	0.021789	**RPL31**	6160
ENSG00000246898	2.212302925	0.006101	0.021797	**NA**	NA
ENSG00000023697	-0.406657578	0.006103	0.021798	**DERA**	51071
ENSG00000001084	0.402975247	0.006105	0.021799	**GCLC**	2729
ENSG00000176978	-0.523951188	0.006109	0.021809	**DPP7**	29952
ENSG00000272391	-0.355879241	0.006114	0.021823	**POM121C**	1E+08
ENSG00000230666	2.730052283	0.006118	0.021833	**CEACAM22**	388550
ENSG00000006638	1.261680395	0.006126	0.021851	**TBXA2R**	6915
ENSG00000102038	-0.345840616	0.006126	0.021851	**SMARCA1**	6594
ENSG00000068028	-0.477869949	0.00613	0.021859	**RASSF1**	11186
ENSG00000173402	0.386901258	0.006133	0.021866	**DAG1**	1605
ENSG00000166224	-0.360062022	0.006136	0.021873	**SGPL1**	8879
ENSG00000247796	0.520480612	0.006144	0.021895	**LOC257396**	257396
ENSG00000087206	-0.353632691	0.006157	0.021938	**UIMC1**	51720
ENSG00000111652	-0.28205708	0.006179	0.022005	**COPS7A**	50813
ENSG00000178105	0.304244667	0.006179	0.022005	**DDX10**	1662
ENSG00000065978	-0.237555216	0.006181	0.022008	**YBX1**	4904
ENSG00000185379	-0.801086925	0.006186	0.022021	**RAD51D**	5892
ENSG00000150867	0.286561937	0.006188	0.022023	**PIP4K2A**	5305
ENSG00000140367	-0.521144512	0.006194	0.022038	**UBE2Q2**	92912

ENSG00000235374	1.632651777	0.006195	0.022038	**SSR4P1**	728039
ENSG00000247679	1.12265967	0.006199	0.022046	**NA**	NA
ENSG00000175105	0.514606384	0.0062	0.022047	**ZNF654**	55279
ENSG00000179163	0.861951687	0.00621	0.022075	**FUCA1**	2517
ENSG00000160094	-1.098659303	0.006217	0.022097	**ZNF362**	149076
ENSG00000197157	0.207000707	0.006225	0.022119	**SND1**	27044
ENSG00000125503	0.331512123	0.006233	0.022145	**PPP1R12C**	54776
ENSG00000205002	1.261087756	0.006248	0.022191	**AARD**	441376
ENSG00000135801	-0.404619432	0.006264	0.022244	**TAF5L**	27097
ENSG00000147202	-0.417245629	0.006268	0.022253	**DIAPH2**	1730
ENSG00000137872	0.957548451	0.006275	0.022266	**SEMA6D**	80031
ENSG00000188636	-0.37586792	0.006274	0.022266	**RTL6**	84247
ENSG00000126215	-0.447299106	0.006282	0.022288	**XRCC3**	7517
ENSG00000147799	0.431659772	0.006288	0.0223	**ARHGAP39**	80728
ENSG00000168538	0.342158934	0.006288	0.0223	**TRAPPC11**	60684
ENSG00000118690	1.215681332	0.006308	0.022362	**ARMC2**	84071
ENSG00000153094	0.614320436	0.006309	0.022362	**BCL2L11**	10018
ENSG00000152219	-0.479083622	0.006313	0.022372	**ARL14EP**	120534
ENSG00000075234	0.380472955	0.006323	0.022402	**TTC38**	55020
ENSG00000114439	-0.387115966	0.00633	0.022419	**BBX**	56987
ENSG00000144357	0.298148653	0.00633	0.022419	**UBR3**	130507
ENSG00000128915	-0.305836802	0.006337	0.022436	**ICE2**	79664
ENSG00000170917	0.583314661	0.006338	0.022436	**NUDT6**	11162
ENSG00000134453	-0.232312076	0.006345	0.022455	**RBM17**	84991
ENSG00000183597	0.653646778	0.006348	0.022461	**TANGO2**	128989
ENSG00000013374	0.223549265	0.006377	0.022556	**NUB1**	51667
ENSG00000169599	-0.460708241	0.006378	0.022556	**NFU1**	27247
ENSG00000132478	-0.884090393	0.00638	0.022561	**UNK**	85451
ENSG00000138002	0.465136525	0.006384	0.02257	**IFT172**	26160
ENSG00000105376	0.594078664	0.006389	0.022582	**ICAM5**	7087
ENSG00000101294	0.189298108	0.006404	0.022631	**HM13**	81502
ENSG00000177951	0.328253795	0.006407	0.022631	**BET1L**	51272
ENSG00000196504	-0.226095495	0.006407	0.022631	**PRPF40A**	55660
ENSG00000163918	-0.351987589	0.006412	0.022641	**RFC4**	5984
ENSG00000223638	4.027719248	0.006413	0.022641	**RFPL4A**	342931
ENSG00000119574	-0.499409446	0.006415	0.022644	**ZBTB45**	84878
ENSG00000213740	-0.529303097	0.006423	0.022666	**NA**	NA
ENSG00000135363	-0.763085616	0.006428	0.022679	**LMO2**	4005
ENSG00000246090	1.030085775	0.006442	0.022723	**OC1005070**	1.01E+08
ENSG00000119638	0.325193404	0.006452	0.022749	**NEK9**	91754
ENSG00000241749	-0.851702516	0.006452	0.022749	**RPSAP52**	204010
ENSG00000275764	1.073879161	0.006454	0.022751	**NA**	NA
ENSG00000110876	1.63644249	0.006463	0.022777	**SELPLG**	6404
ENSG00000276900	0.936260602	0.00647	0.022796	**NA**	NA
ENSG00000097021	0.5197553	0.006483	0.022839	**ACOT7**	11332
ENSG00000108654	0.122215954	0.006491	0.022861	**DDX5**	1655
ENSG00000178149	0.532888353	0.006503	0.0229	**DALRD3**	55152
ENSG00000109501	-0.143144883	0.006505	0.0229	**WFS1**	7466
ENSG00000172809	-0.208710703	0.006506	0.0229	**RPL38**	6169
ENSG00000253729	-0.225826749	0.006513	0.022918	**PRKDC**	5591

ENSG00000112343	0.299739505	0.006518	0.02293	**TRIM38**	10475
ENSG00000127589	-0.536001655	0.006532	0.022974	**NA**	NA
ENSG00000126243	0.599881362	0.006554	0.023049	**LRFN3**	79414
ENSG00000183155	-0.467203667	0.006556	0.023051	**RABIF**	5877
ENSG00000122965	-0.468676288	0.006584	0.023141	**RBM19**	9904
ENSG00000164675	2.637224984	0.006595	0.023175	**IQUB**	154865
ENSG00000106261	0.482660349	0.0066	0.02319	**ZKSCAN1**	7586
ENSG00000090776	0.688730423	0.006606	0.023204	**EFNB1**	1947
ENSG00000072954	0.608689952	0.006612	0.023215	**TMEM38A**	79041
ENSG00000131016	-0.194923126	0.006611	0.023215	**AKAP12**	9590
ENSG00000158882	-0.523964684	0.006619	0.023234	**TOMM40L**	84134
ENSG00000154930	-0.545821006	0.006625	0.023252	**ACSS1**	84532
ENSG00000173517	0.492419018	0.006629	0.023259	**PEAK1**	79834
ENSG00000173889	-0.569923675	0.006633	0.023268	**PHC3**	80012
ENSG00000119508	1.511851529	0.006664	0.023374	**NR4A3**	8013
ENSG00000003147	-0.677755612	0.006678	0.023411	**ICA1**	3382
ENSG00000172845	-0.451364862	0.006677	0.023411	**SP3**	6670
ENSG00000103351	0.522067	0.006681	0.023418	**CLUAP1**	23059
ENSG00000187778	-0.519011402	0.006687	0.023433	**MCRS1**	10445
ENSG00000119004	-0.602509554	0.006706	0.02349	**CYP20A1**	57404
ENSG00000176973	1.065182079	0.006705	0.02349	**FAM89B**	23625
ENSG00000198131	-0.367204039	0.006708	0.02349	**ZNF544**	27300
ENSG00000166987	-0.374124485	0.006719	0.023524	**MBD6**	114785
ENSG00000261609	0.560377568	0.006724	0.023536	**GAN**	8139
ENSG00000124587	0.47930732	0.00673	0.023551	**PEX6**	5190
ENSG00000137338	0.332168731	0.006733	0.023557	**PGBD1**	84547
ENSG00000117114	0.295032671	0.006736	0.023564	**ADGRL2**	23266
ENSG00000162227	-0.533042281	0.006742	0.023577	**TAF6L**	10629
ENSG00000055208	-0.335644519	0.006745	0.023585	**TAB2**	23118
ENSG00000198742	-0.473107916	0.006759	0.023629	**SMURF1**	57154
ENSG00000179091	-0.26590175	0.006769	0.023656	**CYC1**	1537
ENSG00000102302	-0.416562177	0.006779	0.023688	**FGD1**	2245
ENSG00000125170	1.778135728	0.006786	0.023695	**DOK4**	55715
ENSG00000133193	0.405567388	0.006785	0.023695	**FAM104A**	84923
ENSG00000189060	1.621080504	0.006785	0.023695	**H1-0**	3005
ENSG00000067606	0.614657404	0.006793	0.023709	**PRKCZ**	5590
ENSG00000137161	-0.347129813	0.006793	0.023709	**CNPY3**	10695
ENSG00000026652	0.436588801	0.006809	0.023759	**AGPAT4**	56895
ENSG00000117984	-0.26996542	0.00681	0.023759	**CTSD**	1509
ENSG00000082212	0.249663604	0.006835	0.023842	**ME2**	4200
ENSG00000136986	-0.27268371	0.006837	0.023842	**DERL1**	79139
ENSG00000106617	0.26724635	0.00684	0.023844	**PRKAG2**	51422
ENSG00000151135	-0.204265431	0.00684	0.023844	**TMEM263**	90488
ENSG00000134014	-0.485721567	0.006843	0.023847	**ELP3**	55140
ENSG00000102743	-0.583443331	0.006847	0.023855	**SLC25A15**	10166
ENSG00000074211	-0.400138261	0.006854	0.023856	**PPP2R2C**	5522
ENSG00000101367	-0.213381789	0.006854	0.023856	**MAPRE1**	22919
ENSG00000104973	0.315785887	0.006848	0.023856	**MED25**	81857
ENSG00000185862	-0.582655789	0.006854	0.023856	**EVI2B**	2124
ENSG00000231742	1.700759445	0.00685	0.023856	**LINC01273**	1.02E+08

ENSG00000169727	0.261191512	0.006863	0.023881	**GPS1**	2873
ENSG00000186642	0.460869314	0.006871	0.023906	**PDE2A**	5138
ENSG00000002587	0.930841078	0.006885	0.023937	**HS3ST1**	9957
ENSG00000141429	0.224155113	0.006882	0.023937	**GALNT1**	2589
ENSG00000173456	-0.257392214	0.006884	0.023937	**RNF26**	79102
ENSG00000213977	-0.625291447	0.006887	0.023939	**TAX1BP3**	30851
ENSG00000113712	-0.262877758	0.006892	0.023948	**CSNK1A1**	1452
ENSG00000124466	1.989398584	0.006893	0.023948	**LYPD3**	27076
ENSG00000125037	0.377275508	0.006895	0.023949	**EMC3**	55831
ENSG00000152127	0.294589411	0.006902	0.02397	**MGAT5**	4249
ENSG00000144224	-0.298523665	0.006923	0.024039	**UBXN4**	23190
ENSG00000173085	-0.350542066	0.006935	0.024074	**COQ2**	27235
ENSG00000204604	0.624629437	0.006945	0.024103	**ZNF468**	90333
ENSG00000204604	0.624629437	0.006945	0.024103	**ZNF320**	162967
ENSG00000198948	0.470673723	0.00695	0.024115	**MFAP3L**	9848
ENSG00000166394	-0.659467192	0.006956	0.024132	**CYB5R2**	51700
ENSG00000011275	0.335014751	0.006958	0.024134	**RNF216**	54476
ENSG00000167377	1.100090256	0.006966	0.024157	**ZNF23**	7571
ENSG00000075856	-0.337117821	0.006976	0.024175	**SART3**	9733
ENSG00000119431	0.695243167	0.006975	0.024175	**HDHD3**	81932
ENSG00000171490	-0.332221703	0.006975	0.024175	**RSL1D1**	26156
ENSG00000196776	0.245247862	0.007012	0.024295	**CD47**	961
ENSG00000126012	-0.268615904	0.007016	0.024304	**KDM5C**	8242
ENSG00000156471	-0.215955863	0.007022	0.024315	**PTDSS1**	9791
ENSG00000179933	0.244768192	0.007023	0.024315	**C14orf119**	55017
ENSG00000145242	1.0687903	0.007042	0.024375	**EPHA5**	2044
ENSG00000232060	-2.763961824	0.007048	0.024391	**NA**	NA
ENSG00000173065	0.421168375	0.007055	0.024411	**FAM222B**	55731
ENSG00000166881	-0.435043319	0.007057	0.024414	**NEMP1**	23306
ENSG00000127955	0.522910639	0.007071	0.024456	**GNAI1**	2770
ENSG00000100242	-0.346087225	0.007093	0.024523	**SUN2**	25777
ENSG00000108510	-0.502080682	0.007092	0.024523	**MED13**	9969
ENSG00000163625	-0.322290807	0.007113	0.024585	**WDFY3**	23001
ENSG00000028528	0.352081202	0.007122	0.024611	**SNX1**	6642
ENSG00000101052	0.566665809	0.007126	0.024619	**IFT52**	51098
ENSG00000132356	-0.338567356	0.007127	0.024619	**PRKAA1**	5562
ENSG00000172493	0.274344701	0.007132	0.02463	**AFF1**	4299
ENSG00000085978	0.413575348	0.007134	0.02463	**ATG16L1**	55054
ENSG00000136147	-1.010700382	0.007151	0.024686	**PHF11**	51131
ENSG00000123154	0.480515405	0.007172	0.02475	**WDR83**	84292
ENSG00000188612	-0.301510282	0.00718	0.024773	**SUMO2**	6613
ENSG00000037757	0.396182085	0.007198	0.024815	**MRI1**	84245
ENSG00000064787	3.422426225	0.007196	0.024815	**BCAS1**	8537
ENSG00000106244	-0.314416422	0.007198	0.024815	**PDAP1**	11333
ENSG00000167186	-0.829574812	0.007197	0.024815	**COQ7**	10229
ENSG00000149658	0.213326946	0.007218	0.024874	**YTHDF1**	54915
ENSG00000239213	1.102094637	0.007218	0.024874	**NA**	NA
ENSG00000162139	0.467020135	0.007222	0.024879	**NEU3**	10825
ENSG00000166228	-0.435804896	0.007226	0.02489	**PCBD1**	5092
ENSG00000181704	-0.323248299	0.00723	0.024899	**YIPF6**	286451

ENSG00000104375	0.422359255	0.007233	0.024901	**STK3**	6788
ENSG00000197536	-1.425025375	0.007238	0.024916	**NA**	NA
ENSG00000123684	-0.433949907	0.007254	0.02496	**LPGAT1**	9926
ENSG00000225031	-0.744416946	0.007253	0.02496	**NA**	NA
ENSG00000101624	0.377467493	0.007256	0.02496	**CEP76**	79959
ENSG00000146463	-0.449357982	0.007259	0.024964	**ZMYM4**	9202
ENSG00000166130	-0.307357847	0.007278	0.025024	**IKBIP**	121457
ENSG00000198794	0.421282836	0.007291	0.025063	**SCAMP5**	192683
ENSG00000101898	-0.93998909	0.007294	0.025068	**NA**	NA
ENSG00000103855	0.484162021	0.007295	0.025068	**CD276**	80381
ENSG00000112290	0.329654934	0.007303	0.025089	**WASF1**	8936
ENSG00000225975	1.409537176	0.007309	0.025106	**LINC01534**	1.02E+08
ENSG00000086065	0.321896178	0.007324	0.025152	**CHMP5**	51510
ENSG00000107372	0.258465335	0.007338	0.025194	**ZFAND5**	7763
ENSG00000186193	0.23757074	0.00734	0.025196	**SAPCD2**	89958
ENSG00000080031	1.444417475	0.007342	0.025197	**PTPRH**	5794
ENSG00000088340	1.667571493	0.007346	0.025206	**FER1L4**	80307
ENSG00000105643	-0.297422735	0.007354	0.025225	**ARRDC2**	27106
ENSG00000116649	-0.355376821	0.007356	0.025225	**SRM**	6723
ENSG00000136944	3.338713039	0.007357	0.025225	**LMX1B**	4010
ENSG00000143434	1.280706192	0.007358	0.025225	**SEMA6C**	10500
ENSG00000156381	0.361818899	0.007367	0.02525	**ANKRD9**	122416
ENSG00000139428	-0.441419305	0.007373	0.025264	**MMAB**	326625
ENSG00000173786	0.212372624	0.007378	0.025275	**CNP**	1267
ENSG00000118197	-0.679286211	0.007403	0.025358	**DDX59**	83479
ENSG00000107560	-0.597537213	0.007412	0.025383	**RAB11FIP2**	22841
ENSG00000177946	-0.9793175	0.007415	0.025388	**CENPBD1**	92806
ENSG00000102172	-0.280003814	0.007422	0.025404	**SMS**	6611
ENSG00000274070	1.771172294	0.007429	0.025423	**CASTOR2**	729438
ENSG00000196367	-0.339175193	0.007436	0.025443	**TRRAP**	8295
ENSG00000054523	0.324695949	0.007442	0.025453	**KIF1B**	23095
ENSG00000143195	0.339419862	0.007442	0.025453	**ILDR2**	387597
ENSG00000182606	0.307918131	0.007464	0.025522	**TRAK1**	22906
ENSG00000155016	0.608838367	0.007467	0.025526	**CYP2U1**	113612
ENSG00000072110	-0.222808016	0.007471	0.025535	**ACTN1**	87
ENSG00000186815	0.273695806	0.007481	0.025563	**TPCN1**	53373
ENSG00000133808	1.084831446	0.00751	0.025656	**NA**	NA
ENSG00000149600	-0.390087151	0.007513	0.025663	**COMMD7**	149951
ENSG00000149809	0.825868974	0.007518	0.025674	**TM7SF2**	7108
ENSG00000172270	0.169899402	0.007521	0.025677	**BSG**	682
ENSG00000272398	1.993473586	0.007529	0.025698	**CD24**	1E+08
ENSG00000006744	-0.300318712	0.007531	0.025701	**ELAC2**	60528
ENSG00000168765	0.829225177	0.007532	0.025701	**GSTM4**	2948
ENSG00000106683	0.38556658	0.007539	0.025717	**LIMK1**	3984
ENSG00000064726	-0.294804997	0.007542	0.025722	**BTBD1**	53339
ENSG00000140534	-0.423484251	0.007545	0.025726	**TICRR**	90381
ENSG00000102241	0.199886047	0.00755	0.025731	**HTATSF1**	27336
ENSG00000128973	-0.627542534	0.007548	0.025731	**CLN6**	54982
ENSG00000181804	1.246420669	0.007551	0.025731	**SLC9A9**	285195
ENSG00000176533	1.679029251	0.007563	0.025765	**GNG7**	2788

ENSG00000140320	0.409506004	0.007567	0.025773	**BAHD1**	22893
ENSG00000172890	0.438202249	0.007571	0.025782	**NADSYN1**	55191
ENSG00000144736	0.605690932	0.007573	0.025783	**SHQ1**	55164
ENSG00000229358	-1.563534748	0.00758	0.025802	**NA**	NA
ENSG00000112419	-0.407539665	0.007586	0.025811	**PHACTR2**	9749
ENSG00000166833	-0.340229762	0.007585	0.025811	**NAV2**	89797
ENSG00000162413	-0.248593877	0.007594	0.025833	**KLHL21**	9903
ENSG00000148057	-0.988833115	0.0076	0.025843	**IDNK**	414328
ENSG00000164080	0.28393238	0.0076	0.025843	**RAD54L2**	23132
ENSG00000227039	-0.913004283	0.00762	0.025907	**ITGB2-AS1**	1.01E+08
ENSG00000111087	2.556625722	0.007637	0.025958	**GLI1**	2735
ENSG00000087258	1.478330832	0.007644	0.025976	**GNAO1**	2775
ENSG00000066777	-0.4228039	0.007663	0.026033	**ARFGEF1**	10565
ENSG00000196967	0.594408518	0.007672	0.026061	**ZNF585A**	199704
ENSG00000168439	0.1984165	0.007676	0.026068	**STIP1**	10963
ENSG00000258738	-1.346670576	0.007681	0.026079	**)C1122681**	1.12E+08
ENSG00000215417	-1.263468261	0.007687	0.026095	**MIR17HG**	407975
ENSG00000123552	0.46123752	0.007701	0.026136	**USP45**	85015
ENSG00000116489	-0.274660558	0.007705	0.026144	**CAPZA1**	829
ENSG00000116883	1.029036571	0.007727	0.026203	**NA**	NA
ENSG00000120948	-0.333501995	0.007727	0.026203	**TARDBP**	23435
ENSG00000149212	2.061455593	0.007727	0.026203	**SESN3**	143686
ENSG00000148337	-0.204999953	0.007753	0.026286	**CIZ1**	25792
ENSG00000065911	-0.417853779	0.007756	0.026291	**MTHFD2**	10797
ENSG00000211450	-0.310013641	0.007766	0.026317	**SELENOH**	280636
ENSG00000142534	-0.162160244	0.007778	0.026352	**RPS11**	6205
ENSG00000196199	0.429136507	0.00778	0.026354	**MPHOSPH8**	54737
ENSG00000061938	0.342488922	0.007792	0.026389	**TNK2**	10188
ENSG00000167674	0.395733013	0.007799	0.026408	**HDGFL2**	84717
ENSG00000143575	-0.276474001	0.007802	0.026411	**HAX1**	10456
ENSG00000114331	0.238517114	0.007809	0.026431	**ACAP2**	23527
ENSG00000105497	0.469038853	0.007813	0.026434	**ZNF175**	7728
ENSG00000143819	0.346719061	0.007814	0.026434	**EPHX1**	2052
ENSG00000110048	0.274435989	0.007827	0.026472	**OSBP**	5007
ENSG00000102385	0.92002136	0.00783	0.026479	**DRP2**	1821
ENSG00000125398	0.36022854	0.007839	0.026502	**SOX9**	6662
ENSG00000130669	-0.298817287	0.007841	0.026504	**PAK4**	10298
ENSG00000109787	-0.472716952	0.007844	0.026506	**KLF3**	51274
ENSG00000133083	1.36253063	0.007845	0.026506	**DCLK1**	9201
ENSG00000183891	-0.725672054	0.007847	0.026506	**TTC32**	130502
ENSG00000188064	-0.512964466	0.007851	0.026515	**WNT7B**	7477
ENSG00000272579	1.001710111	0.007857	0.02653	**NA**	NA
ENSG00000132549	0.283170128	0.007874	0.026581	**VPS13B**	157680
ENSG00000187801	-0.684617403	0.007878	0.026591	**ZFP69B**	65243
ENSG00000090013	0.696458111	0.007898	0.026652	**BLVRB**	645
ENSG00000100567	-0.202683828	0.007916	0.026706	**PSMA3**	5684
ENSG00000164114	0.755344764	0.007922	0.026722	**MAP9**	79884
ENSG00000115484	-0.224516387	0.007932	0.026743	**CCT4**	10575
ENSG00000178966	-0.400435878	0.007931	0.026743	**RMI1**	80010
ENSG00000149269	0.366576	0.007936	0.026753	**PAK1**	5058

ENSG00000055211	0.327086862	0.007944	0.026772	**GINM1**	116254
ENSG00000235552	-0.362108875	0.007958	0.026813	**NA**	NA
ENSG00000160972	0.412655569	0.007964	0.026828	**PPP1R16A**	84988
ENSG00000119820	-0.279306422	0.007989	0.026908	**YIPF4**	84272
ENSG00000184162	-0.731774882	0.008013	0.026983	**NR2C2AP**	126382
ENSG00000176170	-0.44069701	0.008021	0.027005	**SPHK1**	8877
ENSG00000138764	-0.316863442	0.008029	0.027018	**CCNG2**	901
ENSG00000182872	-0.366631521	0.008029	0.027018	**RBM10**	8241
ENSG00000182013	0.432519607	0.00804	0.027051	**PNMA8A**	55228
ENSG00000069869	-0.338930045	0.008056	0.027076	**NEDD4**	4734
ENSG00000111906	-0.466151199	0.00806	0.027076	**HDDC2**	51020
ENSG00000116260	0.205202641	0.008055	0.027076	**QSOX1**	5768
ENSG00000123728	-0.365850629	0.008053	0.027076	**RAP2C**	57826
ENSG00000136144	0.32115892	0.00806	0.027076	**RCBTB1**	55213
ENSG00000158457	1.690807985	0.008061	0.027076	**TSPAN33**	340348
ENSG00000198754	2.027920588	0.008058	0.027076	**OXCT2**	64064
ENSG00000205885	-1.030064604	0.008055	0.027076	**C1RL-AS1**	283314
ENSG00000145741	-0.265869526	0.008068	0.027092	**BTF3**	689
ENSG00000171757	-1.110534754	0.008076	0.027116	**LRRC34**	151827
ENSG00000124193	-0.357639187	0.008085	0.027137	**SRSF6**	6431
ENSG00000163611	0.425272171	0.008089	0.027146	**SPICE1**	152185
ENSG00000134470	-0.477057907	0.008092	0.027152	**IL15RA**	3601
ENSG00000115966	-0.342798759	0.008105	0.027189	**ATF2**	1386
ENSG00000198700	-0.272482877	0.008123	0.027243	**IPO9**	55705
ENSG00000178719	0.337263239	0.008134	0.027273	**GRINA**	2907
ENSG00000110446	-1.168832377	0.008138	0.027282	**SLC15A3**	51296
ENSG00000157212	-0.425577537	0.008143	0.027292	**PAXIP1**	22976
ENSG00000161513	0.603192273	0.008162	0.027351	**FDXR**	2232
ENSG00000114796	0.451376633	0.008172	0.027378	**KLHL24**	54800
ENSG00000111142	-0.179658066	0.00818	0.027401	**METAP2**	10988
ENSG00000160796	0.305929275	0.008192	0.027433	**NBEAL2**	23218
ENSG00000109686	-0.362360192	0.008196	0.027441	**SH3D19**	152503
ENSG00000125633	-0.331172781	0.008204	0.027458	**CCDC93**	54520
ENSG00000142937	-0.259849587	0.008204	0.027458	**RPS8**	6202
ENSG00000110723	0.731530525	0.008208	0.027464	**EXPH5**	23086
ENSG00000166595	0.37686796	0.008212	0.027474	**CIAO2B**	51647
ENSG00000182054	0.443880867	0.008216	0.027481	**IDH2**	3418
ENSG00000173376	1.045925396	0.00822	0.027486	**NDNF**	79625
ENSG00000060237	-0.241480715	0.008241	0.027551	**WNK1**	65125
ENSG00000165819	-0.346061052	0.008245	0.027557	**METTL3**	56339
ENSG00000187257	-0.337010142	0.008246	0.027557	**RSBN1L**	222194
ENSG00000100299	1.192750517	0.008249	0.027562	**ARSA**	410
ENSG00000183955	-0.32112546	0.008257	0.027581	**KMT5A**	387893
ENSG00000027075	0.785182226	0.008261	0.027585	**PRKCH**	5583
ENSG00000164283	-1.040075707	0.00826	0.027585	**ESM1**	11082
ENSG00000165280	-0.166930435	0.008268	0.027602	**VCP**	7415
ENSG00000116704	-0.352632887	0.008286	0.027656	**SLC35D1**	23169
ENSG00000143341	1.201352023	0.00829	0.027663	**HMCN1**	83872
ENSG00000146242	-0.345485331	0.008293	0.027667	**TPBG**	7162
ENSG00000181982	-0.823291882	0.008331	0.027782	**CCDC149**	91050

ENSG00000197872	2.367410723	0.00833	0.027782	**FAM49A**	81553
ENSG00000154917	1.360260361	0.008337	0.027794	**RAB6B**	51560
ENSG00000162736	-0.332560075	0.008338	0.027794	**NCSTN**	23385
ENSG00000084112	-0.195053698	0.008345	0.027807	**SSH1**	54434
ENSG00000140688	0.371024388	0.008343	0.027807	**C16orf58**	64755
ENSG00000164190	-0.386371491	0.008351	0.027822	**NIPBL**	25836
ENSG00000140939	-0.641707435	0.008355	0.027827	**NOL3**	8996
ENSG00000168077	-0.448864156	0.008366	0.027859	**SCARA3**	51435
ENSG00000225921	-0.342892386	0.008368	0.02786	**NOL7**	51406
ENSG00000165879	0.674431045	0.008373	0.027865	**FRAT1**	10023
ENSG00000230359	1.893856236	0.008373	0.027865	**TPI1P2**	286016
ENSG00000259426	-2.32299355	0.008375	0.027865	**LOC14569[illegible]**	145694
ENSG00000174796	0.476847976	0.008377	0.027866	**THAP6**	152815
ENSG00000174446	-0.482752586	0.008389	0.027902	**SNAPC5**	10302
ENSG00000135845	-0.36957911	0.008395	0.027911	**PIGC**	5279
ENSG00000177807	3.921381233	0.008395	0.027911	**KCNJ10**	3766
ENSG00000260398	2.349767891	0.008399	0.027918	**NA**	NA
ENSG00000101182	-0.209627834	0.008409	0.027943	**PSMA7**	5688
ENSG00000154144	0.400767888	0.008415	0.027957	**TBRG1**	84897
ENSG00000139131	-0.658698939	0.00842	0.027969	**YARS2**	51067
ENSG00000111348	-0.283130952	0.008426	0.027978	**ARHGDIB**	397
ENSG00000177556	0.378238499	0.008425	0.027978	**ATOX1**	475
ENSG00000119231	0.314645017	0.008457	0.028069	**SENP5**	205564
ENSG00000198585	0.413231693	0.008457	0.028069	**NUDT16**	131870
ENSG00000089060	0.375993546	0.008469	0.028098	**SLC8B1**	80024
ENSG00000181924	-0.35645269	0.008469	0.028098	**COA4**	51287
ENSG00000109854	-0.348490769	0.008475	0.02811	**HTATIP2**	10553
ENSG00000112218	0.555075626	0.008479	0.028113	**GPR63**	81491
ENSG00000244968	1.590471824	0.008478	0.028113	**LIFR-AS1**	1.01E+08
ENSG00000263528	-0.323245167	0.008489	0.028139	**IKBKE**	9641
ENSG00000174444	-0.184556845	0.0085	0.028169	**RPL4**	6124
ENSG00000104889	-0.206591916	0.008526	0.028252	**RNASEH2A**	10535
ENSG00000149926	2.439056008	0.008539	0.028286	**TLCD3B**	83723
ENSG00000071189	-0.446707922	0.008544	0.028292	**SNX13**	23161
ENSG00000156052	-0.423973034	0.008543	0.028292	**GNAQ**	2776
ENSG00000113141	0.259361693	0.00855	0.028308	**IK**	3550
ENSG00000214026	-0.731842339	0.008562	0.02834	**MRPL23**	6150
ENSG00000214026	-0.731842339	0.008562	0.02834	**[illegible]C1079873[illegible]**	1.08E+08
ENSG00000073670	1.424952496	0.008566	0.028343	**ADAM11**	4185
ENSG00000196700	-0.318974671	0.008566	0.028343	**ZNF512B**	57473
ENSG00000197021	0.405526221	0.008573	0.02836	**CXorf40B**	541578
ENSG00000180329	-0.627915894	0.008587	0.028399	**CCDC43**	124808
ENSG00000160688	0.426006859	0.008596	0.028424	**FLAD1**	80308
ENSG00000169242	1.533513304	0.008598	0.028425	**EFNA1**	1942
ENSG00000164961	0.439728713	0.008603	0.028436	**WASHC5**	9897
ENSG00000128585	0.343703287	0.008625	0.028495	**MKLN1**	4289
ENSG00000167992	1.683545452	0.008623	0.028495	**VWCE**	220001
ENSG00000185250	2.547473991	0.008627	0.028496	**PPIL6**	285755
ENSG00000196455	0.394940118	0.008633	0.02851	**PIK3R4**	30849
ENSG00000225973	-0.755646121	0.008653	0.028571	**PIGBOS1**	1.02E+08

ENSG00000006125	-0.193251139	0.008658	0.028581	**AP2B1**	163
ENSG00000162236	0.385968262	0.008661	0.028586	**STX5**	6811
ENSG00000123575	0.22326275	0.008664	0.028588	**FAM199X**	139231
ENSG00000145331	-0.858474225	0.008675	0.028619	**TRMT10A**	93587
ENSG00000212123	-2.390487753	0.008678	0.028623	**PRR22**	163154
ENSG00000126821	0.256633713	0.008681	0.028627	**SGPP1**	81537
ENSG00000243927	-0.303846673	0.008688	0.028645	**MRPS6**	64968
ENSG00000099290	-0.323369283	0.008698	0.028673	**WASHC2A**	387680
ENSG00000139410	0.72045324	0.008709	0.028701	**SDSL**	113675
ENSG00000100003	0.525419204	0.008718	0.028727	**SEC14L2**	23541
ENSG00000173898	-0.455565381	0.008749	0.028822	**SPTBN2**	6712
ENSG00000144724	-0.306555082	0.008752	0.028822	**PTPRG**	5793
ENSG00000168036	-0.220393938	0.008753	0.028822	**CTNNB1**	1499
ENSG00000132481	-0.647779504	0.00876	0.028839	**TRIM47**	91107
ENSG00000163719	0.437921512	0.008761	0.028839	**MTMR14**	64419
ENSG00000164520	-0.767457027	0.008773	0.028871	**RAET1E**	135250
ENSG00000139629	0.288694939	0.0088	0.028955	**GALNT6**	11226
ENSG00000116711	0.837688225	0.008811	0.028985	**PLA2G4A**	5321
ENSG00000131462	-0.205310855	0.008814	0.028987	**TUBG1**	7283
ENSG00000189001	1.619740317	0.008838	0.029062	**SBSN**	374897
ENSG00000011638	0.494849796	0.008849	0.029089	**TMEM159**	57146
ENSG00000196715	0.275258196	0.00885	0.029089	**VKORC1L1**	154807
ENSG00000183495	-0.242318672	0.008853	0.029094	**EP400**	57634
ENSG00000164347	0.500312847	0.008858	0.029101	**GFM2**	84340
ENSG00000185519	2.916747658	0.008864	0.029117	**FAM131C**	348487
ENSG00000205339	-0.18857232	0.008871	0.029132	**IPO7**	10527
ENSG00000103507	0.30698876	0.008875	0.029141	**BCKDK**	10295
ENSG00000115009	2.63045311	0.008885	0.029167	**CCL20**	6364
ENSG00000166681	-0.240637198	0.008887	0.029169	**BEX3**	27018
ENSG00000173542	0.324351762	0.008895	0.029189	**MOB1B**	92597
ENSG00000196646	0.643468409	0.008903	0.029204	**ZNF136**	7695
ENSG00000196756	-0.432152541	0.008902	0.029204	**SNHG17**	388796
ENSG00000259781	-0.438284397	0.008911	0.029223	**NA**	NA
ENSG00000173432	0.684243768	0.008918	0.029239	**SAA1**	6288
ENSG00000118855	0.260530853	0.008934	0.029286	**MFSD1**	64747
ENSG00000115641	0.346623385	0.008965	0.029382	**FHL2**	2274
ENSG00000104660	0.386332303	0.008981	0.029427	**LEPROTL1**	23484
ENSG00000115414	0.231696952	0.008987	0.029441	**FN1**	2335
ENSG00000185651	-0.280404104	0.008997	0.029469	**UBE2L3**	7332
ENSG00000182400	0.287092453	0.009006	0.029493	**TRAPPC6B**	122553
ENSG00000049618	-1.052452014	0.009026	0.029552	**ARID1B**	57492
ENSG00000101474	0.271665053	0.009028	0.029552	**APMAP**	57136
ENSG00000132170	-0.32314746	0.009047	0.029594	**PPARG**	5468
ENSG00000170919	0.791421068	0.009046	0.029594	**NA**	NA
ENSG00000179562	0.445149806	0.009045	0.029594	**GCC1**	79571
ENSG00000134970	0.241199187	0.00906	0.029625	**TMED7**	51014
ENSG00000189120	1.203342047	0.009059	0.029625	**SP6**	80320
ENSG00000274925	-1.047404433	0.009066	0.02964	**NA**	NA
ENSG00000159658	0.329159545	0.009078	0.029674	**EFCAB14**	9813
ENSG00000166140	-0.357273002	0.009083	0.029684	**ZFYVE19**	84936

ENSG00000002822	-0.197323581	0.009095	0.029698	**MAD1L1**	8379
ENSG00000115652	0.310892271	0.009095	0.029698	**UXS1**	80146
ENSG00000144233	-0.316908786	0.009091	0.029698	**AMMECR1L**	83607
ENSG00000170525	0.28570266	0.009095	0.029698	**PFKFB3**	5209
ENSG00000101189	-0.314256179	0.009097	0.029699	**MRGBP**	55257
ENSG00000179750	-0.412973077	0.009106	0.029721	**APOBEC3B**	9582
ENSG00000174748	-0.184610383	0.009108	0.029724	**RPL15**	6138
ENSG00000120662	-0.788319909	0.009126	0.029768	**MTRF1**	9617
ENSG00000188211	-1.045922757	0.009126	0.029768	**NCR3LG1**	374383
ENSG00000234043	-3.415690912	0.009128	0.029768	**NA**	NA
ENSG00000119714	-1.527771229	0.009146	0.029823	**GPR68**	8111
ENSG00000071282	1.875942556	0.009157	0.029852	**LMCD1**	29995
ENSG00000099783	-0.208619401	0.009159	0.029852	**HNRNPM**	4670
ENSG00000124160	-0.378169185	0.009165	0.029853	**NCOA5**	57727
ENSG00000148219	1.050566703	0.009164	0.029853	**ASTN2**	23245
ENSG00000150281	-0.901927362	0.009165	0.029853	**CTF1**	1489
ENSG00000197405	2.676269565	0.009168	0.029857	**C5AR1**	728
ENSG00000083099	-0.388522447	0.009178	0.029883	**LYRM2**	57226
ENSG00000198838	-1.679698673	0.009184	0.029896	**RYR3**	6263
ENSG00000157734	1.270591226	0.009186	0.029899	**SNX22**	79856
ENSG00000169994	-0.574633703	0.009194	0.029916	**MYO7B**	4648
ENSG00000162745	1.583000839	0.009198	0.029923	**OLFML2B**	25903
ENSG00000092964	-0.310815792	0.009202	0.029931	**DPYSL2**	1808
ENSG00000164615	0.447560698	0.009217	0.029973	**CAMLG**	819
ENSG00000007255	2.16567048	0.009234	0.030022	**TRAPPC6A**	79090
ENSG00000126945	-0.224097627	0.009236	0.030022	**HNRNPH2**	3188
ENSG00000112769	-0.438217691	0.00925	0.030063	**LAMA4**	3910
ENSG00000182541	-0.51265181	0.009262	0.030096	**LIMK2**	3985
ENSG00000139579	-0.320383373	0.009266	0.030102	**NABP2**	79035
ENSG00000164031	-0.51376465	0.00927	0.030109	**DNAJB14**	79982
ENSG00000055163	2.235710367	0.009299	0.030196	**CYFIP2**	26999
ENSG00000130176	2.618432017	0.009304	0.030208	**CNN1**	1264
ENSG00000112679	-0.463798401	0.009315	0.030238	**DUSP22**	56940
ENSG00000187554	1.287813659	0.009324	0.030259	**TLR5**	7100
ENSG00000165752	0.49277102	0.009327	0.030262	**STK32C**	282974
ENSG00000103876	0.303468504	0.00934	0.030287	**FAH**	2184
ENSG00000117597	-0.276898339	0.009344	0.030287	**UTP25**	27042
ENSG00000152291	-0.171710098	0.009341	0.030287	**TGOLN2**	10618
ENSG00000185989	-0.210839914	0.009342	0.030287	**RASA3**	22821
ENSG00000241127	0.445641994	0.009341	0.030287	**YAE1**	57002
ENSG00000186567	0.853282017	0.009355	0.030316	**CEACAM19**	56971
ENSG00000053747	-0.288787075	0.00936	0.030329	**LAMA3**	3909
ENSG00000134198	1.332657034	0.009367	0.030345	**TSPAN2**	10100
ENSG00000143727	-0.281603232	0.009394	0.030425	**ACP1**	52
ENSG00000198736	0.658822964	0.009403	0.030448	**MSRB1**	51734
ENSG00000100994	0.184938328	0.009416	0.030483	**PYGB**	5834
ENSG00000108384	0.366705026	0.00942	0.030491	**RAD51C**	5889
ENSG00000249353	-0.309426564	0.009424	0.030499	**NA**	NA
ENSG00000131236	0.21112887	0.009437	0.030533	**CAP1**	10487
ENSG00000214223	-0.441607413	0.009439	0.030535	**NA**	NA

ENSG00000266709	-0.593892035	0.009446	0.03055	**MGC12916**	84815
ENSG00000065413	-0.499271392	0.009451	0.030558	**ANKRD44**	91526
ENSG00000145703	1.930344666	0.009452	0.030558	**IQGAP2**	10788
ENSG00000101079	0.308524882	0.009454	0.030559	**NDRG3**	57446
ENSG00000079308	0.543915516	0.009461	0.030575	**TNS1**	7145
ENSG00000173744	0.323997251	0.009467	0.030587	**AGFG1**	3267
ENSG00000144909	0.307091314	0.009476	0.03061	**OSBPL11**	114885
ENSG00000128340	0.286469738	0.009493	0.03065	**RAC2**	5880
ENSG00000131153	-0.343137256	0.009494	0.03065	**GINS2**	51659
ENSG00000264538	-0.544537752	0.009491	0.03065	**NA**	NA
ENSG00000100614	-0.203713863	0.009498	0.030657	**PPM1A**	5494
ENSG00000154122	-0.412917886	0.009504	0.03067	**ANKH**	56172
ENSG00000104140	1.448587644	0.009524	0.030727	**RHOV**	171177
ENSG00000082805	0.397579983	0.009528	0.030734	**ERC1**	23085
ENSG00000155363	-0.31586848	0.009538	0.030762	**MOV10**	4343
ENSG00000143845	0.685103917	0.00955	0.030792	**ETNK2**	55224
ENSG00000116288	-0.18822913	0.009554	0.030799	**PARK7**	11315
ENSG00000153574	-0.339062482	0.009566	0.030832	**RPIA**	22934
ENSG00000163597	-0.320556684	0.009574	0.030853	**SNHG16**	1.01E+08
ENSG00000101413	-0.384090497	0.009577	0.030855	**RPRD1B**	58490
ENSG00000169432	1.533702677	0.009585	0.030874	**SCN9A**	6335
ENSG00000163743	-0.419038797	0.009595	0.030894	**RCHY1**	25898
ENSG00000175416	0.204659484	0.009593	0.030894	**CLTB**	1212
ENSG00000088826	0.615412494	0.009605	0.030919	**SMOX**	54498
ENSG00000230316	-0.626350521	0.009619	0.03096	**FEZF1-AS1**	154860
ENSG00000148396	0.241936158	0.00963	0.030989	**SEC16A**	9919
ENSG00000173638	1.622804803	0.009641	0.031018	**SLC19A1**	6573
ENSG00000136810	-0.171220903	0.009662	0.031075	**TXN**	7295
ENSG00000214174	0.842964404	0.009663	0.031075	**AMZ2P1**	201283
ENSG00000161249	1.998830831	0.009668	0.031086	**DMKN**	93099
ENSG00000122490	0.419046895	0.009673	0.031094	**SLC66A2**	80148
ENSG00000225206	-1.525695267	0.009682	0.031118	**MIR137HG**	400765
ENSG00000135862	-0.20837516	0.00969	0.031138	**LAMC1**	3915
ENSG00000124216	1.752080799	0.009696	0.031144	**SNAI1**	6615
ENSG00000204859	0.620624068	0.009696	0.031144	**ZBTB48**	3104
ENSG00000166436	0.618064859	0.0097	0.03115	**TRIM66**	9866
ENSG00000184465	0.426090318	0.00971	0.031176	**WDR27**	253769
ENSG00000116251	-0.224569101	0.009725	0.03122	**RPL22**	6146
ENSG00000156467	-0.207464794	0.009731	0.03123	**UQCRB**	7381
ENSG00000110200	-0.674019834	0.009739	0.031244	**ANAPC15**	25906
ENSG00000185963	0.296699254	0.009739	0.031244	**BICD2**	23299
ENSG00000134461	0.582936999	0.009742	0.031249	**ANKRD16**	54522
ENSG00000119682	0.306654509	0.009749	0.031263	**AREL1**	9870
ENSG00000122008	-0.383822182	0.009759	0.03129	**POLK**	51426
ENSG00000196867	0.504903533	0.009762	0.031293	**ZFP28**	140612
ENSG00000109436	0.260891933	0.009773	0.031321	**TBC1D9**	23158
ENSG00000175928	3.247490161	0.009782	0.031346	**LRRN1**	57633
ENSG00000082014	0.863516942	0.009799	0.031392	**SMARCD3**	6604
ENSG00000158079	-0.325166878	0.009813	0.031432	**PTPDC1**	138639
ENSG00000139719	-0.420789642	0.009817	0.031439	**VPS33A**	65082

ENSG00000272913	-0.744654408	0.00982	0.031441	**NA**	NA
ENSG00000107937	-0.364256366	0.009826	0.031453	**GTPBP4**	23560
ENSG00000114735	0.526702259	0.009831	0.031462	**HEMK1**	51409
ENSG00000130150	0.562703216	0.009844	0.031497	**MOSPD2**	158747
ENSG00000114354	-0.232652361	0.009847	0.031501	**TFG**	10342
ENSG00000006831	0.225039705	0.009866	0.031557	**ADIPOR2**	79602
ENSG00000139921	-0.240522541	0.009879	0.031593	**TMX1**	81542
ENSG00000172530	-0.535985826	0.009883	0.031597	**BANP**	54971
ENSG00000246228	-0.46729844	0.009887	0.031605	**CASC8**	727677
ENSG00000105197	-0.199605288	0.00989	0.031609	**TIMM50**	92609
ENSG00000048991	0.228924704	0.009894	0.031614	**R3HDM1**	23518
ENSG00000052723	0.263396611	0.009901	0.031629	**SIKE1**	80143
ENSG00000243156	0.40441124	0.009928	0.031709	**MICAL3**	57553
ENSG00000183018	1.958610358	0.00993	0.031712	**SPNS2**	124976
ENSG00000136542	-0.490218649	0.009954	0.031782	**GALNT5**	11227
ENSG00000125434	0.723370035	0.009971	0.031813	**SLC25A35**	399512
ENSG00000132623	0.767479092	0.00997	0.031813	**ANKEF1**	63926
ENSG00000162910	0.52286535	0.009966	0.031813	**MRPL55**	128308
ENSG00000186298	0.247104274	0.009972	0.031813	**PPP1CC**	5501
ENSG00000123130	-0.365302743	0.009989	0.031862	**ACOT9**	23597
ENSG00000104892	1.88765748	0.010007	0.031913	**KLC3**	147700
ENSG00000037474	-0.176323548	0.010013	0.031923	**NSUN2**	54888
ENSG00000136048	-0.415952595	0.010022	0.031935	**DRAM1**	55332
ENSG00000144815	0.483321478	0.010021	0.031935	**NXPE3**	91775
ENSG00000183255	0.171510853	0.010021	0.031935	**PTTG1IP**	754
ENSG00000119408	-0.591498748	0.010056	0.032036	**NEK6**	10783
ENSG00000023171	3.829623725	0.010063	0.03205	**GRAMD1B**	57476
ENSG00000114125	-0.365571567	0.010067	0.03205	**RNF7**	9616
ENSG00000179240	0.969066853	0.010067	0.03205	**GVQW3**	1.01E+08
ENSG00000181588	0.332510784	0.010069	0.03205	**MEX3D**	399664
ENSG00000163872	-0.287885021	0.010092	0.032118	**YEATS2**	55689
ENSG00000215105	0.924791893	0.010101	0.032142	**NA**	NA
ENSG00000106031	2.096692103	0.010106	0.03215	**HOXA13**	3209
ENSG00000043591	1.164877435	0.010139	0.032243	**ADRB1**	153
ENSG00000165632	0.433495308	0.010139	0.032243	**TAF3**	83860
ENSG00000113812	-0.364417682	0.010146	0.032257	**ACTR8**	93973
ENSG00000234171	-0.76872172	0.010148	0.032257	**NASEH1-A[illegible]**	1.01E+08
ENSG00000142186	0.312627979	0.01016	0.032289	**SCYL1**	57410
ENSG00000145214	0.750462872	0.010173	0.032324	**DGKQ**	1609
ENSG00000143578	-0.645929196	0.010184	0.032354	**CREB3L4**	148327
ENSG00000136518	-0.21105867	0.010191	0.03237	**ACTL6A**	86
ENSG00000047365	-0.493808592	0.010194	0.032371	**ARAP2**	116984
ENSG00000107863	-0.338920097	0.010202	0.032385	**ARHGAP21**	57584
ENSG00000157778	-0.45078806	0.010202	0.032385	**PSMG3**	84262
ENSG00000175155	1.190785079	0.010207	0.032393	**YPEL2**	388403
ENSG00000259295	1.629234011	0.010209	0.032393	**NA**	NA
ENSG00000170584	-0.270986194	0.01022	0.032423	**NUDCD2**	134492
ENSG00000156508	-0.208181393	0.010225	0.032431	**EEF1A1**	1915
ENSG00000197122	-0.412505004	0.010236	0.03246	**SRC**	6714
ENSG00000100060	2.347715925	0.010261	0.03252	**MFNG**	4242

ENSG00000118518	-0.410672194	0.01026	0.03252	**RNF146**	81847
ENSG00000196597	0.653265636	0.010259	0.03252	**ZNF782**	158431
ENSG00000233006	1.921928943	0.01027	0.032531	**MIR3936HC**	553103
ENSG00000235890	2.162503829	0.010271	0.032531	**TSPEAR-AS:**	54082
ENSG00000264247	-0.613805279	0.010267	0.032531	**LINC00909**	400657
ENSG00000099385	-0.359187237	0.010275	0.032538	**BCL7C**	9274
ENSG00000107758	0.272390547	0.010293	0.032588	**PPP3CB**	5532
ENSG00000134825	-0.331825176	0.010326	0.032686	**TMEM258**	746
ENSG00000175294	-0.516970683	0.01033	0.032692	**CATSPER1**	117144
ENSG00000185722	-0.317050391	0.010377	0.032835	**ANKFY1**	51479
ENSG00000152240	-0.286936217	0.010388	0.032864	**HAUS1**	115106
ENSG00000100979	0.698333361	0.010395	0.032879	**PLTP**	5360
ENSG00000233101	-3.322596705	0.010403	0.032898	**HOXB-AS3**	404266
ENSG00000228794	0.47836013	0.010413	0.032922	**LINC01128**	643837
ENSG00000228794	0.47836013	0.010413	0.032922	**OC1079848**	1.08E+08
ENSG00000168014	0.327248964	0.010433	0.032981	**C2CD3**	26005
ENSG00000165501	-0.392153552	0.010436	0.032984	**LRR1**	122769
ENSG00000150527	0.220480085	0.010448	0.033015	**MIA2**	4253
ENSG00000100906	-0.203283259	0.010459	0.033043	**NFKBIA**	4792
ENSG00000180423	0.778489418	0.010472	0.033078	**HARBI1**	283254
ENSG00000088756	0.998342122	0.010478	0.033089	**ARHGAP28**	79822
ENSG00000184678	0.834961727	0.010484	0.033102	**H2BC21**	8349
ENSG00000062194	-0.275585633	0.010492	0.033112	**GPBP1**	65056
ENSG00000239887	0.823706981	0.01049	0.033112	**C1orf226**	400793
ENSG00000178295	-0.51599403	0.010497	0.033123	**GEN1**	348654
ENSG00000197818	-0.498004454	0.010502	0.033133	**SLC9A8**	23315
ENSG00000185359	-0.306106006	0.010522	0.033187	**HGS**	9146
ENSG00000111880	0.355247086	0.010524	0.03319	**RNGTT**	8732
ENSG00000120437	-0.281428507	0.01054	0.033232	**ACAT2**	39
ENSG00000008130	-0.273280014	0.010553	0.033266	**NADK**	65220
ENSG00000115808	0.294711213	0.010559	0.03328	**STRN**	6801
ENSG00000113583	-0.247475069	0.010578	0.033333	**C5orf15**	56951
ENSG00000169946	-0.70143924	0.010582	0.033338	**ZFPM2**	23414
ENSG00000105649	1.691718007	0.010621	0.033454	**RAB3A**	5864
ENSG00000081721	-0.386530818	0.010634	0.033483	**DUSP12**	11266
ENSG00000118257	0.519978776	0.010634	0.033483	**NRP2**	8828
ENSG00000037749	0.40828276	0.010641	0.033497	**MFAP3**	4238
ENSG00000135185	-0.437629024	0.010653	0.033526	**TMEM243**	79161
ENSG00000168256	0.402561525	0.010654	0.033526	**NKIRAS2**	28511
ENSG00000187123	0.581084127	0.010666	0.033555	**LYPD6**	130574
ENSG00000163945	0.364157602	0.010673	0.033572	**UVSSA**	57654
ENSG00000259985	-1.642003546	0.010675	0.033572	**NA**	NA
ENSG00000073605	1.80587236	0.010684	0.033592	**GSDMB**	55876
ENSG00000167130	-0.578072295	0.010686	0.033592	**DOLPP1**	57171
ENSG00000169914	0.365756316	0.010691	0.033601	**OTUD3**	23252
ENSG00000182287	0.283407664	0.010698	0.033617	**AP1S2**	8905
ENSG00000101057	-0.205455227	0.01072	0.033679	**MYBL2**	4605
ENSG00000130270	-0.587620479	0.010724	0.033686	**ATP8B3**	148229
ENSG00000171033	0.349691473	0.010742	0.033735	**PKIA**	5569
ENSG00000178307	-0.508562743	0.010756	0.033774	**TMEM11**	8834

ENSG00000108256	-0.215166585	0.010761	0.033782	**NUFIP2**	57532
ENSG00000009950	1.817363806	0.010766	0.033792	**MLXIPL**	51085
ENSG00000109107	1.171149841	0.01077	0.033793	**ALDOC**	230
ENSG00000141076	-0.356055114	0.010771	0.033793	**UTP4**	84916
ENSG00000115073	-0.243198288	0.01079	0.033847	**ACTR1B**	10120
ENSG00000167716	-0.768188072	0.010793	0.03385	**WDR81**	124997
ENSG00000187049	-0.493124983	0.010799	0.033861	**TMEM216**	51259
ENSG00000137628	-0.631385371	0.010812	0.033894	**DDX60**	55601
ENSG00000149016	0.564791082	0.010814	0.033894	**TUT1**	64852
ENSG00000130726	-0.135952672	0.010817	0.033899	**TRIM28**	10155
ENSG00000162552	3.517589622	0.010823	0.033907	**WNT4**	54361
ENSG00000272325	-0.678543453	0.010824	0.033907	**NUDT3**	11165
ENSG00000168288	-0.306174269	0.01083	0.033919	**MMADHC**	27249
ENSG00000100764	-0.311391768	0.010836	0.033932	**PSMC1**	5700
ENSG00000145949	-1.458000811	0.010895	0.034108	**MYLK4**	340156
ENSG00000197302	-0.560236374	0.010915	0.034165	**ZNF720**	124411
ENSG00000197302	-0.560236374	0.010915	0.034165	**)C1079839**	1.08E+08
ENSG00000164855	1.502350556	0.010933	0.034213	**TMEM184A**	202915
ENSG00000101019	-0.29439764	0.010949	0.034258	**UQCC1**	55245
ENSG00000198788	-2.873370294	0.010956	0.034274	**MUC2**	4583
ENSG00000140057	3.161387588	0.010976	0.034328	**AK7**	122481
ENSG00000170153	1.324002368	0.011012	0.034436	**RNF150**	57484
ENSG00000122406	-0.180110705	0.011018	0.034446	**RPL5**	6125
ENSG00000011347	2.019195918	0.011023	0.034456	**SYT7**	9066
ENSG00000115904	-0.355086718	0.011035	0.034481	**SOS1**	6654
ENSG00000234456	-1.253746985	0.011035	0.034481	**MAGI2-AS3**	1.01E+08
ENSG00000143337	-0.287200839	0.01104	0.034485	**TOR1AIP1**	26092
ENSG00000166181	0.265074024	0.011041	0.034485	**API5**	8539
ENSG00000184117	0.442626925	0.011052	0.034511	**NIPSNAP1**	8508
ENSG00000187866	0.320171622	0.011059	0.034529	**FAM122A**	116224
ENSG00000117724	-0.310000909	0.011081	0.034579	**CENPF**	1063
ENSG00000152404	-0.59278695	0.011079	0.034579	**CWF19L2**	143884
ENSG00000204860	-0.563321354	0.011082	0.034579	**NA**	NA
ENSG00000112186	-0.404569811	0.011098	0.034621	**CAP2**	10486
ENSG00000142657	0.236212785	0.011108	0.034646	**PGD**	5226
ENSG00000164061	1.495238615	0.011124	0.034689	**BSN**	8927
ENSG00000142507	-0.248707718	0.011133	0.034704	**PSMB6**	5694
ENSG00000262003	-1.238228	0.011132	0.034704	**)C1019277**	1.02E+08
ENSG00000099822	0.564153532	0.01116	0.034762	**HCN2**	610
ENSG00000107140	0.571666164	0.011162	0.034762	**TESK1**	7016
ENSG00000110717	-0.231481844	0.011156	0.034762	**NDUFS8**	4728
ENSG00000131061	-0.850238407	0.011161	0.034762	**ZNF341**	84905
ENSG00000146918	-0.201205522	0.01116	0.034762	**NCAPG2**	54892
ENSG00000167011	2.837285516	0.011189	0.034837	**NAT16**	375607
ENSG00000249115	-0.491584536	0.011204	0.034879	**HAUS5**	23354
ENSG00000183044	2.137770463	0.011208	0.034884	**ABAT**	18
ENSG00000119801	0.340889187	0.011226	0.034933	**YPEL5**	51646
ENSG00000256268	2.125499356	0.011245	0.034986	**LINC02454**	1.05E+08
ENSG00000062282	0.361849016	0.011254	0.035008	**DGAT2**	84649
ENSG00000253958	1.08314189	0.011266	0.035036	**CLDN23**	137075

ENSG00000114988	0.368429978	0.011276	0.035055	**LMAN2L**	81562
ENSG00000198682	-0.215691468	0.011276	0.035055	**PAPSS2**	9060
ENSG00000179041	-0.422268323	0.011298	0.035116	**RRS1**	23212
ENSG00000074582	-0.345980009	0.011312	0.035152	**BCS1L**	617
ENSG00000160172	1.260304946	0.011319	0.035166	**FAM86C2P**	645332
ENSG00000235410	1.776052225	0.011334	0.035207	**NA**	NA
ENSG00000076555	0.556186129	0.011344	0.035224	**ACACB**	32
ENSG00000132475	0.217037709	0.011344	0.035224	**H3-3B**	3021
ENSG00000140332	-0.266289888	0.01135	0.035238	**TLE3**	7090
ENSG00000144589	-0.589612964	0.011364	0.035274	**STK11IP**	114790
ENSG00000112667	-0.406730212	0.011378	0.035311	**DNPH1**	10591
ENSG00000082781	-0.320189335	0.011462	0.035563	**ITGB5**	3693
ENSG00000108599	-0.540167346	0.011472	0.035586	**AKAP10**	11216
ENSG00000101935	-0.330573831	0.011507	0.035688	**AMMECR1**	9949
ENSG00000110435	-0.222889067	0.01152	0.035695	**PDHX**	8050
ENSG00000162688	0.271424527	0.011514	0.035695	**AGL**	178
ENSG00000171848	0.134765594	0.01152	0.035695	**RRM2**	6241
ENSG00000226054	-1.029263556	0.011515	0.035695	**NA**	NA
ENSG00000235194	-0.94965943	0.01152	0.035695	**PPP1R3E**	90673
ENSG00000168994	-0.796873364	0.011542	0.035755	**PXDC1**	221749
ENSG00000130733	-0.344255772	0.011555	0.03579	**YIPF2**	78992
ENSG00000187642	-1.797116664	0.011557	0.03579	**PERM1**	84808
ENSG00000104450	0.270881693	0.01156	0.03579	**SPAG1**	6674
ENSG00000133612	-0.251251343	0.011606	0.03592	**AGAP3**	116988
ENSG00000180071	-0.607663965	0.011605	0.03592	**ANKRD18A**	253650
ENSG00000112561	1.465225155	0.011624	0.035961	**TFEB**	7942
ENSG00000134369	-0.26270845	0.011622	0.035961	**NAV1**	89796
ENSG00000101222	2.482267469	0.011629	0.035968	**SPEF1**	25876
ENSG00000143315	0.454705266	0.011642	0.036002	**PIGM**	93183
ENSG00000136738	0.32425828	0.011648	0.036014	**STAM**	8027
ENSG00000138311	2.288504128	0.01165	0.036014	**ZNF365**	22891
ENSG00000148225	1.582371927	0.011678	0.036092	**WDR31**	114987
ENSG00000122694	0.866226561	0.011695	0.036138	**GLIPR2**	152007
ENSG00000214783	1.733865288	0.011708	0.036171	**POLR2J4**	84820
ENSG00000224786	1.257692962	0.01172	0.036203	**NA**	NA
ENSG00000156374	-0.419480996	0.011748	0.036277	**PCGF6**	84108
ENSG00000170345	0.780286657	0.011749	0.036277	**FOS**	2353
ENSG00000239462	1.519910805	0.011753	0.036281	**NA**	NA
ENSG00000177606	-0.196529124	0.011757	0.036289	**JUN**	3725
ENSG00000103540	0.256318162	0.01176	0.036291	**CCP110**	9738
ENSG00000081087	0.348987222	0.011763	0.036292	**OSTM1**	28962
ENSG00000081041	0.361277178	0.01177	0.036299	**CXCL2**	2920
ENSG00000166797	-0.357233625	0.011772	0.036299	**CIAO2A**	84191
ENSG00000198862	0.286493532	0.011768	0.036299	**LTN1**	26046
ENSG00000197050	0.631311945	0.011781	0.036319	**ZNF420**	147923
ENSG00000054267	0.267640556	0.011811	0.036405	**ARID4B**	51742
ENSG00000063660	0.446504563	0.011834	0.036469	**GPC1**	2817
ENSG00000146457	-0.303996877	0.011843	0.036489	**WTAP**	9589
ENSG00000174808	-1.514271829	0.011852	0.036512	**BTC**	685
ENSG00000025772	-0.23533937	0.011866	0.036547	**TOMM34**	10953

ENSG00000152223	0.342142417	0.011873	0.03656	**EPG5**	57724
ENSG00000254887	-1.00254826	0.011875	0.03656	**)C1005056.**	1.01E+08
ENSG00000132591	-0.262623117	0.011905	0.036647	**ERAL1**	26284
ENSG00000110958	-0.313656319	0.011915	0.036654	**PTGES3**	10728
ENSG00000160050	0.755726934	0.011919	0.036654	**CCDC28B**	79140
ENSG00000172403	1.269007193	0.011916	0.036654	**SYNPO2**	171024
ENSG00000179046	-0.725082063	0.011912	0.036654	**TRIML2**	205860
ENSG00000188290	0.890008448	0.011918	0.036654	**HES4**	57801
ENSG00000133706	-0.28979697	0.011924	0.036663	**LARS1**	51520
ENSG00000157800	0.315672292	0.011944	0.036715	**SLC37A3**	84255
ENSG00000267374	-1.018512405	0.011957	0.03675	**MIR924HG**	647946
ENSG00000228486	-0.919331967	0.011982	0.036819	**C2orf92**	728537
ENSG00000165626	-0.38723313	0.012009	0.036895	**BEND7**	222389
ENSG00000124787	-0.447672116	0.012052	0.037018	**RPP40**	10799
ENSG00000164619	1.259313937	0.01206	0.037036	**BMPER**	168667
ENSG00000188611	-0.670994627	0.012067	0.037051	**ASAH2**	56624
ENSG00000107331	0.534229633	0.012104	0.037157	**ABCA2**	20
ENSG00000198258	0.285107165	0.012106	0.037157	**UBL5**	59286
ENSG00000006695	-0.369771468	0.012126	0.037212	**COX10**	1352
ENSG00000198720	0.330144873	0.012129	0.037213	**ANKRD13B**	124930
ENSG00000133872	0.377797764	0.01214	0.037231	**SARAF**	51669
ENSG00000144554	-0.422579335	0.012138	0.037231	**FANCD2**	2177
ENSG00000123612	1.841676373	0.012148	0.037245	**ACVR1C**	130399
ENSG00000138778	-0.453201579	0.012149	0.037245	**CENPE**	1062
ENSG00000130511	-0.200497699	0.012155	0.037257	**SSBP4**	170463
ENSG00000168461	0.27993979	0.012175	0.037312	**RAB31**	11031
ENSG00000229043	1.310698946	0.01218	0.037318	**)C1019270.**	1.02E+08
ENSG00000253304	-0.891048024	0.012183	0.037321	**TMEM200E**	399474
ENSG00000198604	-0.397496526	0.012189	0.037332	**BAZ1A**	11177
ENSG00000161395	-0.730601108	0.012195	0.037344	**PGAP3**	93210
ENSG00000116857	0.366843221	0.012203	0.037362	**TMEM9**	252839
ENSG00000100916	-0.463797938	0.012207	0.037367	**BRMS1L**	84312
ENSG00000165475	0.499265565	0.012246	0.037478	**CRYL1**	51084
ENSG00000137076	-0.152967181	0.012267	0.037529	**TLN1**	7094
ENSG00000204876	-0.785739496	0.012266	0.037529	**LOC389602**	389602
ENSG00000179021	-0.334933663	0.012277	0.037552	**C3orf38**	285237
ENSG00000144895	-0.273036599	0.012283	0.037563	**EIF2A**	83939
ENSG00000014138	-0.305476283	0.012287	0.037568	**POLA2**	23649
ENSG00000262814	-0.36321873	0.012311	0.037634	**MRPL12**	6182
ENSG00000089091	0.777231381	0.012338	0.037701	**DZANK1**	55184
ENSG00000158864	-0.474016083	0.012337	0.037701	**NDUFS2**	4720
ENSG00000138658	-0.534190437	0.012344	0.037712	**ZGRF1**	55345
ENSG00000104343	0.407119989	0.012366	0.037774	**UBE2W**	55284
ENSG00000283445	-1.661905259	0.012369	0.037775	**NA**	NA
ENSG00000147224	-0.20410042	0.012376	0.037788	**PRPS1**	5631
ENSG00000143013	-0.335645972	0.012386	0.037804	**LMO4**	8543
ENSG00000179364	0.231346239	0.012385	0.037804	**PACS2**	23241
ENSG00000161791	-0.371141962	0.012397	0.03783	**FMNL3**	91010
ENSG00000167173	0.246940546	0.012403	0.037836	**C15orf39**	56905
ENSG00000215193	-0.377902007	0.012404	0.037836	**PEX26**	55670

ENSG00000280120	-0.667571961	0.012406	0.037836	**NA**	NA
ENSG00000054983	-0.315302842	0.012412	0.037847	**GALC**	2581
ENSG00000149547	-0.216405549	0.012419	0.037856	**EI24**	9538
ENSG00000156709	0.285925486	0.012418	0.037856	**AIFM1**	9131
ENSG00000102098	-0.676989799	0.012445	0.037926	**SCML2**	10389
ENSG00000138769	1.579469738	0.012448	0.037929	**CDKL2**	8999
ENSG00000135535	-0.200087983	0.012453	0.037937	**CD164**	8763
ENSG00000229666	1.070105959	0.012492	0.03805	**NA**	NA
ENSG00000171169	-0.64450233	0.012501	0.038067	**NAIF1**	203245
ENSG00000092200	-1.074750186	0.01251	0.038074	**RPGRIP1**	57096
ENSG00000167889	-0.6235452	0.012508	0.038074	**MGAT5B**	146664
ENSG00000196262	-0.169425225	0.012507	0.038074	**PPIA**	5478
ENSG00000177700	0.247832671	0.01252	0.038098	**POLR2L**	5441
ENSG00000271888	1.582309731	0.012559	0.038208	**NA**	NA
ENSG00000091483	0.183007025	0.012561	0.038209	**FH**	2271
ENSG00000010030	1.971806792	0.012574	0.038239	**ETV7**	51513
ENSG00000166446	0.290061075	0.012597	0.038303	**CDYL2**	124359
ENSG00000138495	-0.477313088	0.012639	0.038422	**COX17**	10063
ENSG00000131446	-0.209793729	0.012652	0.03844	**MGAT1**	4245
ENSG00000162892	2.295376015	0.012651	0.03844	**IL24**	11009
ENSG00000198026	-0.364430544	0.012652	0.03844	**ZNF335**	63925
ENSG00000119203	0.206309859	0.012662	0.038448	**CPSF3**	51692
ENSG00000136045	-0.206461801	0.012662	0.038448	**PWP1**	11137
ENSG00000196547	-0.283035597	0.012659	0.038448	**MAN2A2**	4122
ENSG00000088899	0.505298598	0.012682	0.038503	**LZTS3**	9762
ENSG00000130254	-0.218387759	0.012697	0.038534	**SAFB2**	9667
ENSG00000277053	1.120071235	0.012696	0.038534	**GTF2IP1**	2970
ENSG00000144827	0.490996149	0.012717	0.038588	**ABHD10**	55347
ENSG00000073792	0.180809362	0.012721	0.03859	**IGF2BP2**	10644
ENSG00000139154	-0.434212038	0.012748	0.038666	**AEBP2**	121536
ENSG00000167081	-0.516196195	0.012757	0.038686	**PBX3**	5090
ENSG00000138744	-0.464516083	0.012767	0.038701	**NAAA**	27163
ENSG00000261150	2.600009379	0.012766	0.038701	**EPPK1**	83481
ENSG00000152767	-0.25780813	0.012795	0.03878	**FARP1**	10160
ENSG00000110328	0.559635977	0.012799	0.038783	**GALNT18**	374378
ENSG00000119689	0.172239438	0.01284	0.038899	**DLST**	1743
ENSG00000073008	-0.238314587	0.012849	0.038919	**PVR**	5817
ENSG00000032389	-0.370575219	0.012889	0.039018	**EIPR1**	7260
ENSG00000111696	0.354991966	0.012892	0.039018	**NT5DC3**	51559
ENSG00000151715	2.018970237	0.012893	0.039018	**TMEM45B**	120224
ENSG00000160284	-4.253306912	0.012887	0.039018	**SPATC1L**	84221
ENSG00000174938	0.490921631	0.012889	0.039018	**SEZ6L2**	26470
ENSG00000147119	0.497151533	0.012896	0.039019	**CHST7**	56548
ENSG00000145592	-0.205660547	0.012904	0.039035	**RPL37**	6167
ENSG00000110675	1.504588107	0.012909	0.039042	**ELMOD1**	55531
ENSG00000076242	-0.316349782	0.012952	0.039165	**MLH1**	4292
ENSG00000119547	0.572440055	0.01296	0.039182	**ONECUT2**	9480
ENSG00000126067	-0.182904597	0.012967	0.039196	**PSMB2**	5690
ENSG00000188811	-0.473104122	0.012984	0.039241	**NHLRC3**	387921
ENSG00000186862	1.802225812	0.012993	0.039261	**PDZD7**	79955

ENSG00000105341	-0.34024339	0.013001	0.039275	**DMAC2**	55101
ENSG00000138286	-0.308764765	0.013012	0.039293	**FAM149B1**	317662
ENSG00000260121	-0.942530929	0.01301	0.039293	**NA**	NA
ENSG00000122026	-0.261000712	0.013033	0.039352	**RPL21**	6144
ENSG00000139531	0.513574018	0.013048	0.039382	**SUOX**	6821
ENSG00000162191	0.296588962	0.013047	0.039382	**UBXN1**	51035
ENSG00000101003	-0.509262278	0.013068	0.039433	**GINS1**	9837
ENSG00000062524	0.796265778	0.013075	0.039448	**LTK**	4058
ENSG00000169032	0.233691487	0.013093	0.039496	**MAP2K1**	5604
ENSG00000112137	-0.510178829	0.013129	0.039594	**PHACTR1**	221692
ENSG00000136925	-0.393955078	0.013134	0.039603	**TSTD2**	158427
ENSG00000099875	0.296740908	0.013139	0.039609	**MKNK2**	2872
ENSG00000266401	2.731041483	0.013164	0.039677	**)C1053719(**	1.05E+08
ENSG00000130544	0.526200271	0.013179	0.039717	**ZNF557**	79230
ENSG00000102218	0.389881437	0.013185	0.03972	**RP2**	6102
ENSG00000116922	-0.311598575	0.013184	0.03972	**C1orf109**	54955
ENSG00000008735	2.418892207	0.013202	0.039762	**MAPK8IP2**	23542
ENSG00000229809	-0.84363669	0.013206	0.039768	**ZNF688**	146542
ENSG00000148832	0.933811648	0.013251	0.039894	**PAOX**	196743
ENSG00000071967	-0.502506043	0.013266	0.039932	**CYBRD1**	79901
ENSG00000131791	0.511521957	0.013314	0.040056	**PRKAB2**	5565
ENSG00000148356	-0.440117435	0.01331	0.040056	**LRSAM1**	90678
ENSG00000184182	0.528127097	0.013313	0.040056	**UBE2F**	140739
ENSG00000096063	-0.146135034	0.013327	0.040087	**SRPK1**	6732
ENSG00000121964	-0.430836511	0.013343	0.040127	**GTDC1**	79712
ENSG00000127054	0.277921775	0.013358	0.040157	**INTS11**	54973
ENSG00000204852	0.470148841	0.013357	0.040157	**TCTN1**	79600
ENSG00000101220	0.29500336	0.013367	0.040176	**C20orf27**	54976
ENSG00000178927	0.418399436	0.013379	0.040206	**CYBC1**	79415
ENSG00000114650	0.284518816	0.01341	0.040288	**SCAP**	22937
ENSG00000255248	-0.29806038	0.013412	0.040288	**MIR100HG**	399959
ENSG00000081320	0.333687805	0.013443	0.040376	**STK17B**	9262
ENSG00000176658	0.566117151	0.013463	0.040426	**MYO1D**	4642
ENSG00000068001	0.341772254	0.013493	0.04051	**HYAL2**	8692
ENSG00000006652	-0.307439203	0.013501	0.040527	**IFRD1**	3475
ENSG00000123545	-0.348666919	0.013516	0.040563	**NDUFAF4**	29078
ENSG00000127561	2.046994127	0.013545	0.040642	**SYNGR3**	9143
ENSG00000076864	2.486179415	0.013552	0.040647	**RAP1GAP**	5909
ENSG00000254087	0.378088553	0.01355	0.040647	**LYN**	4067
ENSG00000152620	-0.482516703	0.013561	0.040669	**NADK2**	133686
ENSG00000166313	0.314301844	0.013567	0.040669	**APBB1**	322
ENSG00000258102	0.759552133	0.013564	0.040669	**MAP1LC3B2**	643246
ENSG00000161970	-0.287370396	0.013579	0.040698	**RPL26**	6154
ENSG00000141858	-0.370898318	0.013589	0.040722	**SAMD1**	90378
ENSG00000158301	-0.696188236	0.013604	0.040757	**GPRASP2**	114928
ENSG00000158301	-0.696188236	0.013604	0.040757	**MCX5-GPRA**	1.01E+08
ENSG00000075188	-0.281914027	0.013615	0.040784	**NUP37**	79023
ENSG00000145414	-0.383103163	0.013626	0.040808	**NAF1**	92345
ENSG00000105875	0.466900799	0.013637	0.040833	**WDR91**	29062
ENSG00000160305	-0.298986935	0.013654	0.04087	**DIP2A**	23181

ENSG00000165272	1.714318812	0.013652	0.04087	**AQP3**	360
ENSG00000204954	-0.480644246	0.013661	0.040884	**C12orf73**	728568
ENSG00000131196	0.659652242	0.013692	0.040968	**NFATC1**	4772
ENSG00000184719	-0.64519644	0.01372	0.041043	**RNLS**	55328
ENSG00000128951	-0.369072815	0.013735	0.041082	**DUT**	1854
ENSG00000253738	-0.475315269	0.013739	0.041087	**)TUD6B-AS**	1.01E+08
ENSG00000010438	1.306723768	0.013755	0.041113	**PRSS3**	5646
ENSG00000196586	0.257243109	0.013756	0.041113	**MYO6**	4646
ENSG00000198001	0.3673062	0.013756	0.041113	**IRAK4**	51135
ENSG00000164305	-0.27266462	0.013772	0.041155	**CASP3**	836
ENSG00000278828	1.28947855	0.0138	0.041229	**H3C10**	8357
ENSG00000150764	0.892459424	0.013808	0.041245	**DIXDC1**	85458
ENSG00000084463	-0.22528957	0.013822	0.041265	**WBP11**	51729
ENSG00000144306	0.425725265	0.01382	0.041265	**SCRN3**	79634
ENSG00000215021	-0.243578105	0.013818	0.041265	**PHB2**	11331
ENSG00000165389	0.260258147	0.013848	0.041335	**SPTSSA**	171546
ENSG00000100577	-0.390583484	0.013859	0.041351	**GSTZ1**	2954
ENSG00000166685	-0.261917575	0.013858	0.041351	**COG1**	9382
ENSG00000173275	-0.590480152	0.013885	0.04142	**ZNF449**	203523
ENSG00000106615	-0.24582142	0.013898	0.041452	**RHEB**	6009
ENSG00000111667	-0.351592369	0.013943	0.041557	**USP5**	8078
ENSG00000149418	1.992672813	0.013942	0.041557	**ST14**	6768
ENSG00000173890	0.422111198	0.013944	0.041557	**GPR160**	26996
ENSG00000249867	-1.113386269	0.013938	0.041557	**LINC02742**	1.05E+08
ENSG00000111203	-0.888575164	0.013958	0.041586	**ITFG2**	55846
ENSG00000111727	0.514860413	0.013957	0.041586	**HCFC2**	29915
ENSG00000106804	-0.93211121	0.013967	0.041598	**C5**	727
ENSG00000147872	0.248223179	0.013968	0.041598	**PLIN2**	123
ENSG00000146729	-0.374928501	0.01398	0.041619	**NIPSNAP2**	2631
ENSG00000182362	-0.473731416	0.01398	0.041619	**YBEY**	54059
ENSG00000141959	-0.299710581	0.014013	0.04171	**PFKL**	5211
ENSG00000130475	1.378801505	0.014028	0.041747	**FCHO1**	23149
ENSG00000125458	-0.634696179	0.014031	0.041749	**NT5C**	30833
ENSG00000072135	-0.556471675	0.014061	0.041828	**PTPN18**	26469
ENSG00000157064	0.386989291	0.014064	0.04183	**NMNAT2**	23057
ENSG00000019144	0.289960607	0.014076	0.041857	**PHLDB1**	23187
ENSG00000160190	1.767264474	0.014102	0.041927	**SLC37A1**	54020
ENSG00000197363	0.682908269	0.014122	0.041979	**ZNF517**	340385
ENSG00000118363	-0.290658705	0.014125	0.041981	**SPCS2**	9789
ENSG00000163682	-0.197167074	0.014139	0.041997	**RPL9**	6133
ENSG00000227354	0.617151992	0.014137	0.041997	**RBM26-AS1**	1.01E+08
ENSG00000250072	-1.264486884	0.014139	0.041997	**SH3TC2-DT**	255187
ENSG00000187266	0.949048336	0.014148	0.042013	**EPOR**	2057
ENSG00000277734	-1.020445444	0.014149	0.042013	**NA**	NA
ENSG00000279456	1.640340384	0.014165	0.042052	**NA**	NA
ENSG00000145439	0.466629805	0.014173	0.042069	**CBR4**	84869
ENSG00000123989	0.282270508	0.014177	0.042071	**CHPF**	79586
ENSG00000105948	0.416787992	0.014186	0.042082	**TTC26**	79989
ENSG00000156463	0.520820762	0.014185	0.042082	**SH3RF2**	153769
ENSG00000120256	0.381581437	0.014209	0.042142	**LRP11**	84918

ENSG00000169180	-0.204369888	0.014246	0.042244	**XPO6**	23214
ENSG00000176593	-0.54486521	0.014256	0.042267	**C1001283**	1E+08
ENSG00000139116	-0.47635634	0.014264	0.042282	**KIF21A**	55605
ENSG00000203814	1.208849573	0.014275	0.042309	**H2BC18**	440689
ENSG00000161243	-0.474692822	0.014279	0.042311	**FBXO27**	126433
ENSG00000106628	-0.166447072	0.014291	0.04234	**POLD2**	5425
ENSG00000127586	0.358151186	0.01433	0.042447	**CHTF18**	63922
ENSG00000067992	1.951058519	0.014349	0.042496	**PDK3**	5165
ENSG00000144674	0.318687368	0.014375	0.042564	**GOLGA4**	2803
ENSG00000139946	1.721517934	0.014384	0.042584	**PELI2**	57161
ENSG00000136867	1.554857077	0.014395	0.042606	**SLC31A2**	1318
ENSG00000178033	1.708428981	0.014398	0.042606	**CALHM5**	254228
ENSG00000255769	1.252345742	0.0144	0.042606	**NA**	NA
ENSG00000240344	-0.45586975	0.014453	0.042757	**PPIL3**	53938
ENSG00000128617	0.88320094	0.014466	0.042788	**OPN1SW**	611
ENSG00000130810	-0.666674233	0.014475	0.042806	**PPAN**	56342
ENSG00000185838	0.691766361	0.014489	0.042839	**GNB1L**	54584
ENSG00000122257	-0.232694747	0.014511	0.042878	**RBBP6**	5930
ENSG00000145425	-0.212659539	0.014509	0.042878	**RPS3A**	6189
ENSG00000179399	2.655240706	0.01451	0.042878	**GPC5**	2262
ENSG00000134250	0.150037015	0.014542	0.042964	**NOTCH2**	4853
ENSG00000171295	0.530432476	0.014552	0.042985	**ZNF440**	126070
ENSG00000186468	-0.187487045	0.014572	0.043035	**RPS23**	6228
ENSG00000167508	0.311061725	0.014576	0.04304	**MVD**	4597
ENSG00000174276	-0.586113417	0.014585	0.043059	**ZNHIT2**	741
ENSG00000148737	-0.409976838	0.014588	0.043059	**TCF7L2**	6934
ENSG00000109929	0.406655992	0.014613	0.043126	**SC5D**	6309
ENSG00000176896	2.379929256	0.014621	0.043142	**TCEANC**	170082
ENSG00000100629	0.371382707	0.014678	0.043285	**CEP128**	145508
ENSG00000139178	-0.977840219	0.014676	0.043285	**C1RL**	51279
ENSG00000144645	-0.238221198	0.014678	0.043285	**OSBPL10**	114884
ENSG00000103021	2.38347531	0.0147	0.043343	**CCDC113**	29070
ENSG00000125991	-0.191945819	0.014704	0.043346	**ERGIC3**	51614
ENSG00000071051	0.467790864	0.014724	0.04338	**NCK2**	8440
ENSG00000083535	0.342014174	0.014723	0.04338	**PIBF1**	10464
ENSG00000198718	0.401462875	0.014719	0.04338	**TOGARAM1**	23116
ENSG00000126934	0.174429748	0.014735	0.043406	**MAP2K2**	5605
ENSG00000057704	0.341404166	0.014752	0.043448	**TMCC3**	57458
ENSG00000172273	0.425102468	0.014758	0.043457	**HINFP**	25988
ENSG00000188706	0.372665872	0.014773	0.043493	**ZDHHC9**	51114
ENSG00000136842	2.643636397	0.014792	0.043539	**TMOD1**	7111
ENSG00000107438	0.29038573	0.014801	0.043558	**PDLIM1**	9124
ENSG00000198408	-0.240829957	0.014803	0.043558	**OGA**	10724
ENSG00000278175	-1.206371923	0.014809	0.043567	**GLIDR**	389741
ENSG00000099910	0.60221431	0.014822	0.043589	**KLHL22**	84861
ENSG00000139613	-0.253877674	0.014822	0.043589	**SMARCC2**	6601
ENSG00000144504	-0.949194009	0.014829	0.043601	**ANKMY1**	51281
ENSG00000159228	0.219181867	0.014858	0.043679	**CBR1**	873
ENSG00000092969	-0.409695567	0.014872	0.043712	**TGFB2**	7042
ENSG00000251000	-1.565003405	0.014878	0.04372	**NA**	NA

ENSG00000185361	0.451826439	0.01491	0.043807	**TNFAIP8L1**	126282
ENSG00000134030	0.603685973	0.014915	0.043814	**CTIF**	9811
ENSG00000021300	1.597162551	0.014919	0.043817	**PLEKHB1**	58473
ENSG00000185305	0.562628865	0.014929	0.043839	**ARL15**	54622
ENSG00000072803	0.236702972	0.014939	0.043844	**FBXW11**	23291
ENSG00000126217	2.306976108	0.014937	0.043844	**MCF2L**	23263
ENSG00000271614	0.936473332	0.014934	0.043844	**ATP2B1-AS**	338758
ENSG00000133789	0.344213625	0.014965	0.043911	**SWAP70**	23075
ENSG00000212719	0.318091203	0.015004	0.04402	**LINC02693**	339263
ENSG00000100991	-0.218734308	0.015015	0.044043	**TRPC4AP**	26133
ENSG00000103184	3.091102564	0.015018	0.044043	**SEC14L5**	9717
ENSG00000197540	3.861780168	0.015028	0.044063	**GZMM**	3004
ENSG00000119718	0.235868793	0.01504	0.04409	**EIF2B2**	8892
ENSG00000175701	-0.54804495	0.015042	0.044091	**MTLN**	205251
ENSG00000242715	0.892537152	0.015051	0.044108	**CCDC169**	728591
ENSG00000047578	0.384154198	0.015058	0.044121	**KIAA0556**	23247
ENSG00000130303	0.286858579	0.015062	0.044125	**BST2**	684
ENSG00000026297	0.603635744	0.015095	0.044203	**RNASET2**	8635
ENSG00000133874	1.676121916	0.015097	0.044203	**RNF122**	79845
ENSG00000213020	0.485415629	0.015094	0.044203	**ZNF611**	81856
ENSG00000196417	0.582336718	0.015112	0.044238	**ZNF765**	91661
ENSG00000214193	0.959309173	0.015133	0.044292	**SH3D21**	79729
ENSG00000135940	0.247351338	0.015136	0.044293	**COX5B**	1329
ENSG00000085832	0.288683969	0.015145	0.044311	**EPS15**	2060
ENSG00000168528	0.822891106	0.015193	0.044443	**SERINC2**	347735
ENSG00000151468	0.69812396	0.015213	0.044494	**CCDC3**	83643
ENSG00000109586	-0.320276899	0.015222	0.044503	**GALNT7**	51809
ENSG00000279069	-1.416485586	0.01522	0.044503	**NA**	NA
ENSG00000224870	-0.464719158	0.015249	0.044573	**MRPL20-AS**	148413
ENSG00000137274	0.707429307	0.015258	0.044592	**BPHL**	670
ENSG00000148143	-0.415014704	0.015267	0.04461	**ZNF462**	58499
ENSG00000169955	-1.117945298	0.015281	0.044644	**ZNF747**	65988
ENSG00000259969	2.258648816	0.015287	0.044651	**NA**	NA
ENSG00000135473	-0.485377044	0.015293	0.044662	**PAN2**	9924
ENSG00000060138	-0.214491944	0.015318	0.044725	**YBX3**	8531
ENSG00000156587	0.354197587	0.015341	0.044785	**UBE2L6**	9246
ENSG00000183378	-1.272019299	0.015345	0.044787	**OVCH2**	341277
ENSG00000120093	-1.16420023	0.015386	0.0449	**HOXB3**	3213
ENSG00000198363	-0.1680348	0.015412	0.044967	**ASPH**	444
ENSG00000011454	0.319599667	0.015423	0.044991	**GPR21**	2844
ENSG00000011454	0.319599667	0.015423	0.044991	**RABGAP1**	23637
ENSG00000181381	-0.407879589	0.015432	0.045009	**DDX60L**	91351
ENSG00000185414	-0.311066002	0.015447	0.045044	**MRPL30**	51263
ENSG00000087365	-0.158386786	0.01547	0.045102	**SF3B2**	10992
ENSG00000075945	0.347949428	0.015476	0.045114	**KIFAP3**	22920
ENSG00000170166	-2.046533349	0.015491	0.045147	**HOXD4**	3233
ENSG00000240230	-0.57857389	0.015496	0.045147	**COX19**	90639
ENSG00000250562	-2.345114263	0.015496	0.045147	**NA**	NA
ENSG00000059122	0.545628436	0.015561	0.045326	**FLYWCH1**	84256
ENSG00000167065	0.800443255	0.015569	0.045344	**DUSP18**	150290

ENSG00000168763	-0.335616509	0.015574	0.045348	**CNNM3**	26505
ENSG00000167676	1.100381376	0.015594	0.045398	**PLIN4**	729359
ENSG00000037897	-0.822710119	0.015598	0.045402	**METTL1**	4234
ENSG00000182310	-1.116915879	0.015621	0.045448	**SPACA6**	147650
ENSG00000188997	0.471233137	0.01562	0.045448	**KCTD21**	283219
ENSG00000253352	-0.317337591	0.015622	0.045448	**TUG1**	55000
ENSG00000103160	0.379972052	0.015665	0.045565	**HSDL1**	83693
ENSG00000166897	0.997833752	0.015668	0.045565	**ELFN2**	114794
ENSG00000100644	0.15744695	0.015721	0.04571	**HIF1A**	3091
ENSG00000241697	2.026788227	0.015747	0.045776	**TMEFF1**	8577
ENSG00000135655	0.262429154	0.015757	0.04579	**USP15**	9958
ENSG00000150990	-0.375005934	0.015755	0.04579	**DHX37**	57647
ENSG00000187514	-0.226631696	0.015776	0.045838	**PTMA**	5757
ENSG00000173281	0.528544749	0.015785	0.045854	**PPP1R3B**	79660
ENSG00000176714	1.197661646	0.015805	0.045903	**CCDC121**	79635
ENSG00000113494	0.95669923	0.015813	0.04592	**PRLR**	5618
ENSG00000147604	-0.192419074	0.015826	0.045947	**RPL7**	6129
ENSG00000117394	0.26927334	0.015829	0.045949	**SLC2A1**	6513
ENSG00000155329	-0.465252827	0.015842	0.045978	**ZCCHC10**	54819
ENSG00000129473	-0.300758203	0.015851	0.045996	**BCL2L2**	599
ENSG00000133313	-0.219060593	0.015864	0.046025	**CNDP2**	55748
ENSG00000143420	-0.170671926	0.015881	0.04606	**ENSA**	2029
ENSG00000168615	-0.187329089	0.015882	0.04606	**ADAM9**	8754
ENSG00000280239	-0.806735675	0.015889	0.046074	**NA**	NA
ENSG00000114491	-0.345794245	0.015895	0.046074	**UMPS**	7372
ENSG00000156735	0.338383098	0.015895	0.046074	**BAG4**	9530
ENSG00000125753	0.174965549	0.015987	0.04633	**VASP**	7408
ENSG00000174547	-0.320985131	0.016009	0.046388	**MRPL11**	65003
ENSG00000134717	-0.241254395	0.016012	0.046388	**BTF3L4**	91408
ENSG00000004399	0.334418159	0.016038	0.046454	**PLXND1**	23129
ENSG00000100246	0.808818327	0.016047	0.046469	**DNAL4**	10126
ENSG00000136457	1.823065129	0.016049	0.046469	**CHAD**	1101
ENSG00000153250	-0.270336533	0.016061	0.04649	**RBMS1**	5937
ENSG00000249996	-3.409510343	0.016062	0.04649	**NA**	NA
ENSG00000070010	0.271018292	0.016083	0.046533	**UFD1**	7353
ENSG00000167562	0.516792629	0.016083	0.046533	**ZNF701**	55762
ENSG00000188846	-0.161693405	0.016086	0.046533	**RPL14**	9045
ENSG00000066427	-0.399271642	0.016102	0.046571	**ATXN3**	4287
ENSG00000217165	-0.74991792	0.016132	0.046649	**NA**	NA
ENSG00000054654	-0.283699972	0.01615	0.046688	**SYNE2**	23224
ENSG00000120784	-0.568396454	0.016151	0.046688	**ZFP30**	22835
ENSG00000196419	-0.205634859	0.016157	0.046698	**XRCC6**	2547
ENSG00000084710	1.953179157	0.016165	0.046712	**EFR3B**	22979
ENSG00000163626	-0.36654564	0.016178	0.04674	**COX18**	285521
ENSG00000093009	-0.263873462	0.016208	0.046819	**CDC45**	8318
ENSG00000154727	-0.530284781	0.016242	0.046908	**GABPA**	2551
ENSG00000148842	0.52592762	0.016247	0.046914	**CNNM2**	54805
ENSG00000103966	0.30864305	0.016268	0.046968	**EHD4**	30844
ENSG00000172216	0.371891596	0.016274	0.046975	**CEBPB**	1051
ENSG00000162643	2.169302518	0.016284	0.046996	**WDR63**	126820

ENSG00000165983	-0.341089995	0.016289	0.047003	**PTER**	9317
ENSG00000160293	-0.297217776	0.016341	0.047134	**VAV2**	7410
ENSG00000168152	0.669183192	0.016338	0.047134	**THAP9**	79725
ENSG00000163479	0.17618135	0.016349	0.04715	**SSR2**	6746
ENSG00000244274	-0.882433438	0.016364	0.047184	**DBNDD2**	55861
ENSG00000146094	0.60451211	0.01637	0.047194	**DOK3**	79930
ENSG00000169188	-0.208204559	0.016384	0.047225	**APEX2**	27301
ENSG00000133466	-0.835148755	0.016388	0.047227	**C1QTNF6**	114904
ENSG00000275457	-1.410362181	0.01643	0.047339	**NA**	NA
ENSG00000005206	0.34323948	0.016434	0.047343	**SPPL2B**	56928
ENSG00000172086	-0.406259974	0.016437	0.047344	**KRCC1**	51315
ENSG00000092098	0.392351601	0.016443	0.047352	**RNF31**	55072
ENSG00000103245	-0.437293159	0.016453	0.047371	**CIAO3**	64428
ENSG00000203724	-0.846624018	0.01649	0.047471	**C1orf53**	388722
ENSG00000163655	-0.221093154	0.016494	0.047474	**GMPS**	8833
ENSG00000175198	0.559177404	0.016501	0.047482	**PCCA**	5095
ENSG00000245248	1.441336072	0.016503	0.047482	**NA**	NA
ENSG00000244625	-1.605174725	0.016512	0.047498	**NA**	NA
ENSG00000172819	-0.289578201	0.016518	0.047507	**RARG**	5916
ENSG00000135547	1.805295019	0.016522	0.04751	**HEY2**	23493
ENSG00000004660	0.684434699	0.016581	0.047645	**CAMKK1**	84254
ENSG00000105784	1.560169476	0.016573	0.047645	**RUNDC3B**	154661
ENSG00000176124	0.5732905	0.016581	0.047645	**DLEU1**	10301
ENSG00000176124	0.5732905	0.016581	0.047645	**DLEU7-AS1**	1.01E+08
ENSG00000187091	1.764651497	0.016576	0.047645	**PLCD1**	5333
ENSG00000135749	0.347725804	0.016584	0.047647	**PCNX2**	80003
ENSG00000099622	-0.318837069	0.016607	0.047703	**CIRBP**	1153
ENSG00000125864	-0.644865956	0.016623	0.047738	**BFSP1**	631
ENSG00000175229	-1.216095158	0.016628	0.047738	**GAL3ST3**	89792
ENSG00000186472	0.433947374	0.016627	0.047738	**PCLO**	27445
ENSG00000095906	0.273792593	0.016642	0.04777	**NUBP2**	10101
ENSG00000162396	0.585072908	0.01665	0.047785	**PARS2**	25973
ENSG00000157343	1.950103828	0.016661	0.047806	**ARMC12**	221481
ENSG00000187325	-0.391956448	0.016667	0.047815	**TAF9B**	51616
ENSG00000173868	2.330693259	0.01668	0.047844	**PHOSPHO1**	162466
ENSG00000167004	-0.138655215	0.016705	0.047906	**PDIA3**	2923
ENSG00000159714	0.69247604	0.016713	0.047912	**ZDHHC1**	29800
ENSG00000197312	0.256734409	0.016713	0.047912	**DDI2**	84301
ENSG00000182742	-2.357482607	0.016721	0.047927	**HOXB4**	3214
ENSG00000075292	-0.215293635	0.01673	0.047946	**ZNF638**	27332
ENSG00000143977	-0.218522652	0.01675	0.047994	**SNRPG**	6637
ENSG00000169139	-0.311390465	0.016773	0.048052	**UBE2V2**	7336
ENSG00000116604	0.230631869	0.016808	0.048116	**MEF2D**	4209
ENSG00000166454	0.283088601	0.016807	0.048116	**ATMIN**	23300
ENSG00000183943	-0.534829808	0.016811	0.048116	**PRKX**	5613
ENSG00000198740	-0.488942263	0.016811	0.048116	**ZNF652**	22834
ENSG00000261572	2.755838215	0.016804	0.048116	**NA**	NA
ENSG00000103742	-0.73601382	0.016827	0.048153	**IGDCC4**	57722
ENSG00000228463	1.439718561	0.016834	0.048164	**NA**	NA
ENSG00000107201	-0.361898535	0.016841	0.048175	**DDX58**	23586

ENSG00000149150	-0.472219176	0.016847	0.048175	**SLC43A1**	8501
ENSG00000164300	0.406330837	0.016845	0.048175	**SERINC5**	256987
ENSG00000118620	-0.741310727	0.016883	0.048269	**ZNF430**	80264
ENSG00000183208	1.238677138	0.016894	0.048284	**GDPGP1**	390637
ENSG00000269688	-1.893769276	0.016891	0.048284	**NA**	NA
ENSG00000085224	-0.33179671	0.016933	0.048386	**ATRX**	546
ENSG00000175137	0.311593891	0.016966	0.048474	**SH3BP5L**	80851
ENSG00000001629	0.265719328	0.016978	0.0485	**ANKIB1**	54467
ENSG00000187735	0.197524153	0.016994	0.048536	**TCEA1**	6917
ENSG00000146376	0.283149429	0.017003	0.048553	**ARHGAP18**	93663
ENSG00000179981	-0.547219134	0.017016	0.048573	**TSHZ1**	10194
ENSG00000198517	0.505339497	0.017014	0.048573	**MAFK**	7975
ENSG00000196208	1.347190095	0.017071	0.048721	**GREB1**	9687
ENSG00000105426	0.199257778	0.017074	0.048721	**PTPRS**	5802
ENSG00000168491	1.783724605	0.01709	0.048759	**CCDC110**	256309
ENSG00000118412	-0.33425891	0.017094	0.048759	**CASP8AP2**	9994
ENSG00000112531	-0.326083354	0.017108	0.048792	**QKI**	9444
ENSG00000118600	-0.405735779	0.017115	0.048802	**RXYLT1**	10329
ENSG00000274849	-1.644818261	0.017134	0.048849	**NA**	NA
ENSG00000107566	-0.227530975	0.017145	0.048866	**ERLIN1**	10613
ENSG00000121101	1.3508695	0.017149	0.048866	**TEX14**	56155
ENSG00000122068	0.24699039	0.017147	0.048866	**FYTTD1**	84248
ENSG00000105329	0.21888625	0.017157	0.048878	**TGFB1**	7040
ENSG00000143393	0.265303421	0.017166	0.048895	**PI4KB**	5298
ENSG00000164002	-0.49697695	0.01718	0.048925	**EXO5**	64789
ENSG00000106868	-0.525267971	0.017189	0.048943	**SUSD1**	64420
ENSG00000167523	0.395812466	0.017195	0.048952	**SPATA33**	124045
ENSG00000060566	1.673206327	0.017236	0.049059	**CREB3L3**	84699
ENSG00000143061	1.92190614	0.017256	0.0491	**IGSF3**	3321
ENSG00000150459	0.205269615	0.017256	0.0491	**SAP18**	10284
ENSG00000272341	0.931895512	0.017269	0.049119	**NA**	NA
ENSG00000272695	0.506886045	0.017268	0.049119	**GAS6-DT**	1.01E+08
ENSG00000147421	-0.654239999	0.017278	0.049134	**HMBOX1**	79618
ENSG00000115738	1.938637987	0.017284	0.049145	**ID2**	3398
ENSG00000160294	-0.222208611	0.017287	0.049145	**MCM3AP**	8888
ENSG00000100324	-0.346896847	0.017291	0.049146	**TAB1**	10454
ENSG00000196323	-0.287836087	0.017295	0.049148	**ZBTB44**	29068
ENSG00000160062	-0.672024995	0.0173	0.049154	**ZBTB8A**	653121
ENSG00000007866	0.481808809	0.017326	0.049221	**TEAD3**	7005
ENSG00000079931	0.725472231	0.017335	0.049237	**MOXD1**	26002
ENSG00000185669	1.220642832	0.017353	0.049278	**SNAI3**	333929
ENSG00000087095	0.306404218	0.017369	0.049311	**NLK**	51701
ENSG00000151693	-0.300066081	0.017371	0.049311	**ASAP2**	8853
ENSG00000241058	0.432072155	0.017384	0.049341	**NSUN6**	221078
ENSG00000154265	0.657798015	0.017398	0.049371	**ABCA5**	23461
ENSG00000242259	-0.460031346	0.017427	0.049445	**C22orf39**	128977
ENSG00000134996	0.319884643	0.017436	0.04946	**OSTF1**	26578
ENSG00000276234	-1.133076418	0.017447	0.049484	**TADA2A**	6871
ENSG00000137413	-0.342329168	0.017451	0.049488	**TAF8**	129685
ENSG00000169592	-0.366519826	0.017469	0.049528	**INO80E**	283899

ENSG00000185418	0.507990576	0.017481	0.049555	**TARS3**	123283
ENSG00000173611	0.464958524	0.017492	0.049571	**SCAI**	286205
ENSG00000175573	0.274451667	0.017493	0.049571	**C11orf68**	83638
ENSG00000075539	0.172522464	0.017528	0.04966	**FRYL**	285527
ENSG00000158161	0.339469442	0.01755	0.049715	**EYA3**	2140
ENSG00000153006	-0.269148419	0.017556	0.049721	**SREK1IP1**	285672
ENSG00000011376	-0.325138652	0.017563	0.049734	**LARS2**	23395
ENSG00000196739	-0.433385428	0.017576	0.049762	**COL27A1**	85301
ENSG00000005189	-0.832441515	0.017583	0.049771	**REXO5**	81691
ENSG00000116815	-0.503430937	0.017605	0.049826	**CD58**	965
ENSG00000105085	0.386606602	0.017617	0.04985	**MED26**	9441
ENSG00000032742	0.811970805	0.017642	0.0499	**IFT88**	8100
ENSG00000148848	1.047385488	0.01764	0.0499	**ADAM12**	8038
ENSG00000197467	-0.22186332	0.017644	0.0499	**COL13A1**	1305
ENSG00000168522	-0.381292705	0.017659	0.049936	**FNTA**	2339

Supplementary Table 2												
SYMBOL	S1	S2	S3	S4	S5	S6	baseMean	log2FoldChange	lfcSE	pvalue	padj	ENTREZID
ABCD2	0.00	0.00	1.00	5.52	4.32	3.81	4.91	5.89	1.19	8.15E-07	2.64E-05	225
ABLIM2	4.00	3.00	5.00	8.23	6.71	6.15	42.31	2.32	0.57	4.28E-05	3.32E-04	84448
ACSBG1	2.81	2.32	2.00	6.27	4.46	4.00	9.18	2.45	0.54	5.56E-06	8.29E-05	23205
ADA	3.00	3.81	4.09	7.41	5.88	5.67	27.77	2.23	0.42	1.32E-07	8.74E-06	100
APLP1	3.81	4.32	4.95	8.10	6.77	6.44	46.90	2.22	0.45	9.59E-07	2.94E-05	333
RHGEF26-A	1.58	1.00	2.32	5.78	4.52	3.81	6.84	2.93	0.71	3.73E-05	3.01E-04	100507524
BMF	4.00	4.09	4.39	7.95	6.43	5.95	38.54	2.23	0.39	6.72E-09	1.55E-06	90427
C1orf116	2.58	2.00	2.81	5.93	4.70	4.64	11.09	2.48	0.53	2.76E-06	5.29E-05	79098
C2CD4C	1.58	2.32	2.32	6.21	4.52	3.70	8.36	2.75	0.65	2.09E-05	2.00E-04	126567
CCDC114	1.00	2.32	2.00	5.58	4.17	3.81	6.65	2.53	0.63	6.60E-05	4.56E-04	93233
CCDC153	3.32	1.58	3.32	6.51	5.04	4.70	13.34	2.24	0.54	3.62E-05	2.95E-04	283152
CDK18	1.00	0.00	1.00	5.29	3.91	3.00	5.05	4.25	0.99	1.74E-05	1.77E-04	5129
CGNL1	2.32	3.32	3.81	7.03	5.86	5.04	19.89	2.30	0.55	2.97E-05	2.56E-04	84952
CITED1	2.00	1.58	2.58	5.91	4.52	4.17	9.10	2.62	0.63	2.82E-05	2.47E-04	4435
CMTM8	2.58	3.32	3.70	7.02	6.09	5.04	23.11	2.47	0.56	9.92E-06	1.18E-04	152189
CNTN1	4.32	4.32	4.17	7.92	6.78	5.64	38.09	2.15	0.38	2.24E-08	3.37E-06	1272
CYP39A1	3.00	3.70	3.46	6.85	5.55	4.70	18.01	2.10	0.49	1.83E-05	1.83E-04	51302
CYTH4	4.52	4.75	5.32	8.89	7.42	6.89	67.61	2.23	0.38	5.56E-09	1.40E-06	27128
DIRAS1	0.00	0.00	2.00	5.95	4.17	4.32	6.48	4.74	1.06	8.11E-06	1.03E-04	148252
DLG1-AS1	1.00	2.58	1.58	5.83	4.81	4.17	8.37	3.00	0.63	1.67E-06	3.90E-05	100507086
DLX4	2.32	2.58	0.00	5.73	4.09	4.17	7.75	2.38	0.53	8.70E-06	1.08E-04	1748
EAF2	3.00	2.32	2.81	6.44	4.52	4.70	14.23	2.26	0.47	1.27E-06	3.43E-05	55840
EML1	2.00	2.81	3.46	7.25	5.49	5.09	25.24	2.93	0.50	5.86E-09	1.43E-06	2009
ENTPD3	3.58	3.46	4.39	7.89	6.60	5.36	36.78	2.49	0.54	4.33E-06	7.10E-05	956
ERBB3	4.75	4.70	6.04	9.29	7.78	7.03	81.66	2.18	0.53	4.38E-05	3.39E-04	2065
FBXO44	3.46	3.17	4.25	7.53	5.86	5.49	27.33	2.13	0.49	1.13E-05	1.30E-04	93611
FILIP1	2.32	2.58	2.81	6.74	5.29	4.17	12.84	2.77	0.57	1.03E-06	3.08E-05	27145
GHET1	1.00	2.32	1.58	6.02	4.58	4.25	7.84	3.24	0.62	2.20E-07	1.20E-05	102723099
GIPR	3.00	2.00	3.91	7.42	5.70	5.39	21.06	2.51	0.54	3.96E-06	6.72E-05	2696
GOLGA7B	0.00	2.58	3.00	6.75	5.43	4.81	15.17	3.16	0.68	3.52E-06	6.21E-05	401647
HAL	2.58	2.32	2.58	6.15	4.75	4.64	10.96	2.62	0.49	1.00E-07	7.43E-06	3034
HSPA12A	0.00	1.00	2.81	6.74	5.25	4.91	21.67	4.10	0.64	1.31E-10	1.29E-07	259217
HYKK	3.46	4.00	3.17	7.10	5.83	5.43	21.11	2.22	0.37	2.89E-09	9.53E-07	123688
IL2RB	2.00	1.00	2.00	6.11	4.09	4.17	7.82	3.31	0.69	1.48E-06	3.73E-05	3560
INHBA	2.81	2.81	2.32	6.25	4.86	4.09	14.28	2.04	0.46	7.70E-06	9.98E-05	3624
ITGA7	1.00	1.00	2.32	6.09	4.46	3.70	7.54	3.54	0.82	1.71E-05	1.74E-04	3679
KIAA0319	3.58	3.17	3.70	7.12	5.64	4.81	20.95	2.07	0.44	3.03E-06	5.62E-05	9856
KIF21B	2.00	0.00	3.00	6.61	5.25	4.46	15.30	3.33	0.68	8.41E-07	2.70E-05	23046
L1CAM	2.81	3.00	4.09	7.26	6.02	5.09	26.96	2.30	0.52	8.91E-06	1.10E-04	3897
LCA5	3.70	3.00	4.52	7.61	6.25	5.67	32.55	2.10	0.51	3.27E-05	2.74E-04	167691
LCA5L	1.58	1.58	1.58	5.75	3.81	3.81	8.10	2.79	0.60	2.92E-06	5.49E-05	150082
LHX6	2.81	2.32	3.00	6.25	5.17	4.25	13.51	2.35	0.51	3.88E-06	6.63E-05	26468
LINC00173	2.00	1.00	2.58	5.91	4.17	4.17	7.13	2.67	0.67	5.82E-05	4.17E-04	100287569
LLGL2	4.00	3.58	4.91	8.22	6.63	6.07	41.20	2.26	0.53	1.83E-05	1.82E-04	3993

**LRAT**	3.46	2.81	3.32	6.93	5.09	4.86	19.38	2.05	0.40	3.11E-07	1.45E-05	9227
**MAN1C1**	1.00	0.00	1.00	5.25	3.70	3.17	3.98	4.68	1.13	3.31E-05	2.76E-04	57134
**NXPH3**	1.58	2.00	3.17	6.85	5.61	4.75	14.38	3.13	0.62	4.84E-07	1.88E-05	11248
**OTULINL**	4.17	3.58	4.39	8.02	6.29	5.58	37.32	2.07	0.43	1.50E-06	3.74E-05	54491
**PARM1**	4.58	3.70	5.17	8.33	7.08	6.39	53.56	2.18	0.48	6.00E-06	8.66E-05	25849
**PPFIA4**	3.00	2.58	3.46	6.58	4.81	4.95	14.47	2.20	0.54	4.61E-05	3.52E-04	8497
**REEP6**	5.70	5.17	6.43	9.50	8.10	7.52	110.78	2.01	0.49	3.67E-05	2.98E-04	92840
**RGMA**	1.58	2.00	2.32	6.44	4.86	4.25	9.82	3.13	0.60	2.01E-07	1.14E-05	56963
**RIMKLA**	3.81	2.81	3.17	7.50	5.98	4.86	22.56	2.70	0.48	2.36E-08	3.42E-06	284716
**RPLP0P2**	1.00	1.58	2.81	6.02	4.91	4.52	14.67	3.02	0.55	4.12E-08	4.77E-06	113157
**RPS6KA2**	2.32	3.17	3.46	7.28	6.13	5.00	27.57	2.87	0.48	2.39E-09	9.07E-07	6196
**RSPH4A**	0.00	0.00	1.58	5.46	3.81	3.17	4.47	4.27	1.06	5.82E-05	4.17E-04	345895
**SCG2**	4.09	3.32	4.25	7.67	5.83	5.52	26.95	2.03	0.46	8.92E-06	1.10E-04	7857
**SEMA3G**	2.32	2.81	2.58	6.51	5.29	5.04	15.68	2.49	0.40	4.10E-10	2.81E-07	56920
**SLC2A4**	4.00	3.58	5.13	8.69	7.28	6.44	52.79	2.65	0.59	8.01E-06	1.03E-04	6517
**SPINT1**	1.58	2.32	3.81	7.30	5.61	4.91	21.85	2.79	0.64	1.55E-05	1.62E-04	6692
**SYPL2**	3.17	4.25	4.39	7.79	6.32	5.93	37.84	2.19	0.40	4.98E-08	5.31E-06	284612
**TENT5C**	4.32	4.86	4.75	8.30	6.82	6.41	50.96	2.12	0.37	1.26E-08	2.38E-06	54855
**TMEM145**	1.00	1.00	2.32	5.58	4.86	4.00	8.61	3.22	0.68	1.95E-06	4.26E-05	284339
**TMEM52**	2.81	2.58	3.17	6.63	5.09	4.58	15.17	2.41	0.50	1.37E-06	3.58E-05	339456
**TMEM8B**	2.81	2.00	3.46	6.55	5.36	5.00	15.90	2.37	0.53	7.12E-06	9.63E-05	51754
**TNFRSF9**	2.32	1.58	3.46	6.55	4.75	4.81	15.38	2.42	0.57	2.01E-05	1.94E-04	3604
**TTC21A**	2.58	3.00	4.17	7.31	5.83	5.64	23.00	2.44	0.56	1.42E-05	1.52E-04	199223
**TTYH2**	3.00	2.81	3.17	6.95	5.00	4.70	16.61	2.25	0.44	2.31E-07	1.23E-05	94015
**TUBB4A**	1.58	1.58	3.17	6.51	5.09	4.46	12.54	3.04	0.76	5.84E-05	4.18E-04	10382
**VWA1**	4.09	3.58	5.46	8.93	7.36	6.69	58.55	2.51	0.63	5.81E-05	4.17E-04	64856
**WNT11**	0.00	1.58	2.00	5.21	4.39	4.17	6.99	3.43	0.73	3.00E-06	5.60E-05	7481
**ZMYND10**	1.00	1.58	2.00	6.19	4.58	3.91	7.49	3.72	0.82	6.28E-06	8.91E-05	51364
**ZNF540**	2.32	2.32	2.00	6.39	5.00	3.81	11.52	2.70	0.57	2.03E-06	4.38E-05	163255

Supplementary Table 3								
ONTO	ID	Description	GeneRatio	BgRatio	pvalue	p.adjust	qvalue	Count
BP	**GO:0001890**	placenta developmer	25/935	157/20536	4.56E-08	0.00024	0.000216	25
BP	**GO:0001892**	embryonic placenta d	18/935	91/20536	1.23E-07	0.00032	0.000288	18
BP	**GO:0048608**	reproductive structur	46/935	445/20536	2.00E-07	0.00032	0.000288	46
BP	**GO:0061458**	reproductive system	46/935	448/20536	2.43E-07	0.00032	0.000288	46
BP	**GO:0048568**	embryonic organ dev	46/935	466/20536	7.47E-07	0.000785	0.000706	46
MF	**GO:0001228**	DNA-binding transcri	46/934	462/19669	1.83E-06	0.000839	0.000796	46
MF	**GO:0001216**	DNA-binding transcri	46/934	463/19669	1.94E-06	0.000839	0.000796	46
MF	**GO:0019838**	growth factor binding	21/934	149/19669	7.75E-06	0.002237	0.002123	21
BP	**GO:0001655**	urogenital system de	36/935	349/20536	4.40E-06	0.003656	0.00329	36
BP	**GO:0001822**	kidney development	32/935	296/20536	5.61E-06	0.003656	0.00329	32
BP	**GO:0007389**	pattern specification	44/935	473/20536	6.13E-06	0.003656	0.00329	44
BP	**GO:0043627**	response to estrogen	14/935	75/20536	6.26E-06	0.003656	0.00329	14
BP	**GO:0048545**	response to steroid h	40/935	416/20536	7.37E-06	0.003872	0.003484	40
BP	**GO:0045444**	fat cell differentiation	28/935	250/20536	1.10E-05	0.005257	0.00473	28
BP	**GO:0018108**	peptidyl-tyrosine pho	38/935	399/20536	1.54E-05	0.005975	0.005377	38
BP	**GO:0072001**	renal system develop	32/935	311/20536	1.55E-05	0.005975	0.005377	32
BP	**GO:0001503**	ossification	40/935	430/20536	1.61E-05	0.005975	0.005377	40
BP	**GO:0061448**	connective tissue dev	30/935	284/20536	1.71E-05	0.005975	0.005377	30
BP	**GO:0018212**	peptidyl-tyrosine mo	38/935	402/20536	1.82E-05	0.005975	0.005377	38
BP	**GO:0070670**	response to interleuk	9/935	35/20536	1.96E-05	0.006055	0.005449	9
BP	**GO:0001701**	in utero embryonic d	38/935	405/20536	2.15E-05	0.006268	0.00564	38
BP	**GO:0001958**	endochondral ossifica	8/935	28/20536	2.47E-05	0.006485	0.005836	8
BP	**GO:0036075**	replacement ossificat	8/935	28/20536	2.47E-05	0.006485	0.005836	8
BP	**GO:0055093**	response to hyperoxi	7/935	21/20536	2.63E-05	0.006582	0.005922	7
BP	**GO:0050731**	positive regulation of	24/935	212/20536	3.84E-05	0.009182	0.008262	24
CC	**GO:0005938**	cell cortex	32/966	325/21659	2.52E-05	0.010249	0.009646	32
CC	**GO:0031225**	anchored component	22/966	189/21659	3.87E-05	0.010249	0.009646	22
BP	**GO:0044782**	cilium organization	37/935	407/20536	5.38E-05	0.012305	0.011072	37
BP	**GO:0051098**	regulation of binding	37/935	409/20536	5.97E-05	0.012939	0.011643	37
BP	**GO:0048732**	gland development	42/935	488/20536	6.15E-05	0.012939	0.011643	42
BP	**GO:0001578**	microtubule bundle f	14/935	92/20536	6.86E-05	0.013213	0.011889	14
BP	**GO:0006978**	DNA damage respons	6/935	17/20536	7.04E-05	0.013213	0.011889	6
BP	**GO:0061298**	retina vasculature de	6/935	17/20536	7.04E-05	0.013213	0.011889	6
BP	**GO:1901342**	regulation of vasculat	39/935	445/20536	7.58E-05	0.013743	0.012366	39
BP	**GO:0030330**	DNA damage respons	16/935	118/20536	8.95E-05	0.014977	0.013477	16
BP	**GO:0071353**	cellular response to i	8/935	33/20536	9.00E-05	0.014977	0.013477	8
BP	**GO:0045669**	positive regulation of	12/935	72/20536	9.12E-05	0.014977	0.013477	12
BP	**GO:0042770**	signal transduction in	18/935	144/20536	9.82E-05	0.015634	0.014068	18
BP	**GO:0042772**	DNA damage respons	6/935	18/20536	0.000102	0.015694	0.014122	6
BP	**GO:0030278**	regulation of ossificat	24/935	227/20536	0.000114	0.017119	0.015404	24
BP	**GO:0070493**	thrombin-activated re	5/935	12/20536	0.000117	0.017121	0.015406	5
MF	**GO:0004713**	protein tyrosine kinas	19/934	148/19669	7.67E-05	0.016606	0.015764	19
BP	**GO:1903035**	negative regulation o	14/935	97/20536	0.000123	0.017534	0.015778	14
BP	**GO:0031214**	biomineral tissue dev	20/935	174/20536	0.000134	0.01793	0.016134	20
BP	**GO:0110148**	biomineralization	20/935	174/20536	0.000134	0.01793	0.016134	20

BP	**GO:0070482**	response to oxygen le	38/935	442/20536	0.000139	0.01793	0.016134	38
BP	**GO:0072006**	nephron developmen	18/935	148/20536	0.00014	0.01793	0.016134	18
CC	**GO:0016327**	apicolateral plasma m	6/966	19/21659	0.000127	0.018032	0.016972	6
CC	**GO:0030426**	growth cone	20/966	180/21659	0.000163	0.018032	0.016972	20
CC	**GO:0005930**	axoneme	16/966	128/21659	0.000187	0.018032	0.016972	16
CC	**GO:0097014**	ciliary plasm	16/966	129/21659	0.000205	0.018032	0.016972	16
BP	**GO:0034329**	cell junction assembly	38/935	444/20536	0.000152	0.019043	0.017135	38
CC	**GO:0030427**	site of polarized grow	20/966	186/21659	0.000253	0.01914	0.018014	20
BP	**GO:0042476**	odontogenesis	17/935	137/20536	0.000166	0.020299	0.018265	17
CC	**GO:0046658**	anchored component	10/966	60/21659	0.000295	0.019481	0.018335	10
BP	**GO:0051216**	cartilage developmen	23/935	219/20536	0.000173	0.020705	0.018631	23
BP	**GO:0030323**	respiratory tube deve	21/935	192/20536	0.000186	0.021722	0.019546	21
BP	**GO:0050730**	regulation of peptidy	27/935	279/20536	0.000195	0.021722	0.019546	27
BP	**GO:1901888**	regulation of cell junc	23/935	221/20536	0.000198	0.021722	0.019546	23
BP	**GO:0036296**	response to increased	7/935	28/20536	0.000203	0.021722	0.019546	7
BP	**GO:0042730**	fibrinolysis	7/935	28/20536	0.000203	0.021722	0.019546	7
BP	**GO:0030902**	hindbrain developme	19/935	166/20536	0.000207	0.021725	0.019549	19
BP	**GO:0031032**	actomyosin structure	22/935	209/20536	0.00023	0.023292	0.020958	22
BP	**GO:0060711**	labyrinthine layer dev	9/935	47/20536	0.000231	0.023292	0.020958	9
BP	**GO:1904018**	positive regulation of	24/935	238/20536	0.000235	0.023292	0.020958	24
BP	**GO:0045766**	positive regulation of	22/935	211/20536	0.000263	0.025422	0.022875	22
BP	**GO:0043524**	negative regulation o	18/935	156/20536	0.000272	0.025422	0.022875	18
BP	**GO:0070669**	response to interleuk	5/935	14/20536	0.000275	0.025422	0.022875	5
BP	**GO:1901890**	positive regulation of	15/935	117/20536	0.000276	0.025422	0.022875	15
BP	**GO:0090183**	regulation of kidney d	10/935	59/20536	0.000301	0.026526	0.023869	10
BP	**GO:0001539**	cilium or flagellum-de	15/935	118/20536	0.000303	0.026526	0.023869	15
BP	**GO:0060285**	cilium-dependent cell	15/935	118/20536	0.000303	0.026526	0.023869	15
BP	**GO:0045746**	negative regulation o	9/935	49/20536	0.000322	0.027536	0.024778	9
BP	**GO:0007411**	axon guidance	27/935	288/20536	0.000325	0.027536	0.024778	27
BP	**GO:0097485**	neuron projection gu	27/935	289/20536	0.000343	0.027654	0.024884	27
BP	**GO:0045667**	regulation of osteobla	17/935	146/20536	0.000357	0.027654	0.024884	17
BP	**GO:0072073**	kidney epithelium de	17/935	146/20536	0.000357	0.027654	0.024884	17
BP	**GO:0060485**	mesenchyme develop	27/935	290/20536	0.000362	0.027654	0.024884	27
BP	**GO:0061045**	negative regulation o	12/935	83/20536	0.000364	0.027654	0.024884	12
BP	**GO:0070169**	positive regulation of	9/935	50/20536	0.000377	0.027654	0.024884	9
BP	**GO:0110151**	positive regulation of	9/935	50/20536	0.000377	0.027654	0.024884	9
BP	**GO:0043393**	regulation of protein	23/935	231/20536	0.000377	0.027654	0.024884	23
BP	**GO:0030324**	lung development	20/935	188/20536	0.000379	0.027654	0.024884	20
BP	**GO:1903034**	regulation of respons	20/935	188/20536	0.000379	0.027654	0.024884	20
CC	**GO:0043296**	apical junction compl	17/966	152/21659	0.000455	0.026716	0.025145	17
BP	**GO:0060350**	endochondral bone m	11/935	72/20536	0.00039	0.028059	0.025248	11
BP	**GO:0061098**	positive regulation of	10/935	61/20536	0.000398	0.028264	0.025432	10
BP	**GO:0090184**	positive regulation of	8/935	41/20536	0.000449	0.031032	0.027924	8
BP	**GO:0044458**	motile cilium assemb	6/935	23/20536	0.000454	0.031032	0.027924	6
BP	**GO:0045765**	regulation of angioge	34/935	405/20536	0.00047	0.031032	0.027924	34
BP	**GO:0060271**	cilium assembly	33/935	389/20536	0.000471	0.031032	0.027924	33
BP	**GO:0030282**	bone mineralization	15/935	123/20536	0.000476	0.031032	0.027924	15

BP	**GO:0097191**	extrinsic apoptotic si	24/935	250/2053	0.000485	0.031032	0.027924	24
BP	**GO:0070286**	axonemal dynein con	7/935	32/20536	0.000491	0.031032	0.027924	7
BP	**GO:0045778**	positive regulation of	13/935	98/20536	0.000495	0.031032	0.027924	13
BP	**GO:0048041**	focal adhesion assem	12/935	86/20536	0.000507	0.031032	0.027924	12
BP	**GO:0045665**	negative regulation o	23/935	236/2053	0.00051	0.031032	0.027924	23
BP	**GO:0035082**	axoneme assembly	10/935	63/20536	0.00052	0.031032	0.027924	10
BP	**GO:0051893**	regulation of focal ad	10/935	63/20536	0.00052	0.031032	0.027924	10
BP	**GO:0090109**	regulation of cell-sub	10/935	63/20536	0.00052	0.031032	0.027924	10
BP	**GO:0150116**	regulation of cell-sub	10/935	63/20536	0.00052	0.031032	0.027924	10
BP	**GO:0030501**	positive regulation of	8/935	42/20536	0.000532	0.031444	0.028294	8
BP	**GO:0001649**	osteoblast differentia	24/935	252/2053	0.000544	0.031752	0.028571	24
BP	**GO:0032355**	response to estradiol	16/935	138/2053	0.000552	0.031894	0.028699	16
BP	**GO:0032956**	regulation of actin cy	31/935	361/2053	0.000563	0.031929	0.02873	31
BP	**GO:0003002**	regionalization	32/935	377/2053	0.000565	0.031929	0.02873	32
BP	**GO:0030195**	negative regulation o	9/935	53/20536	0.00059	0.03297	0.029667	9
BP	**GO:0060674**	placenta blood vesse	7/935	33/20536	0.000599	0.033142	0.029822	7
BP	**GO:0007409**	axonogenesis	39/935	494/2053	0.000631	0.034521	0.031063	39
BP	**GO:0001938**	positive regulation of	14/935	114/2053	0.000676	0.036439	0.032788	14
BP	**GO:1900047**	negative regulation o	9/935	54/20536	0.000679	0.036439	0.032788	9
BP	**GO:1902903**	regulation of supram	32/935	382/2053	0.000707	0.037509	0.033751	32
BP	**GO:0061299**	retina vasculature m	4/935	10/20536	0.000719	0.037598	0.033832	4
BP	**GO:0070167**	regulation of biomine	13/935	102/2053	0.00073	0.037598	0.033832	13
BP	**GO:0110149**	regulation of biomine	13/935	102/2053	0.00073	0.037598	0.033832	13
BP	**GO:0006904**	vesicle docking involv	8/935	44/20536	0.000738	0.037647	0.033876	8
BP	**GO:0035988**	chondrocyte prolifera	5/935	17/20536	0.000757	0.03781	0.034023	5
BP	**GO:0010810**	regulation of cell-sub	22/935	228/2053	0.000764	0.03781	0.034023	22
BP	**GO:0050678**	regulation of epitheli	33/935	400/2053	0.000765	0.03781	0.034023	33
BP	**GO:0061041**	regulation of wound	17/935	156/2053	0.00077	0.03781	0.034023	17
MF	**GO:0019199**	transmembrane rece	13/934	88/19669	0.000253	0.037825	0.035908	13
MF	**GO:0045499**	chemorepellent activ	7/934	28/19669	0.000262	0.037825	0.035908	7
BP	**GO:0048762**	mesenchymal cell dif	22/935	230/2053	0.000858	0.041712	0.037533	22
BP	**GO:0010039**	response to iron ion	7/935	35/20536	0.000871	0.041712	0.037533	7
BP	**GO:0030193**	regulation of blood c	11/935	79/20536	0.000873	0.041712	0.037533	11
BP	**GO:2001236**	regulation of extrinsi	18/935	172/2053	0.000882	0.041754	0.037571	18
BP	**GO:0060541**	respiratory system de	21/935	216/2053	0.000904	0.042413	0.038165	21
BP	**GO:0060706**	cell differentiation in	6/935	26/20536	0.000921	0.042859	0.038565	6
BP	**GO:0001935**	endothelial cell prolif	20/935	202/2053	0.000946	0.043608	0.039239	20
BP	**GO:1900046**	regulation of hemost	11/935	80/20536	0.000971	0.044392	0.039945	11
BP	**GO:0038084**	vascular endothelial	8/935	46/20536	0.001002	0.044949	0.040446	8
BP	**GO:0001101**	response to acid che	31/935	374/2053	0.001009	0.044949	0.040446	31
BP	**GO:0048339**	paraxial mesoderm d	5/935	18/20536	0.00101	0.044949	0.040446	5
BP	**GO:0050819**	negative regulation o	9/935	57/20536	0.001018	0.044949	0.040446	9
BP	**GO:2001237**	negative regulation o	14/935	119/2053	0.001037	0.045423	0.040873	14
BP	**GO:0002040**	sprouting angiogenes	19/935	189/2053	0.001046	0.045435	0.040884	19
CC	**GO:0005912**	adherens junction	11/966	80/21659	0.000822	0.043489	0.040932	11
BP	**GO:0032970**	regulation of actin fil	33/935	408/2053	0.001069	0.045513	0.040954	33
BP	**GO:0009952**	anterior/posterior pa	22/935	234/2053	0.001076	0.045513	0.040954	22

BP	**GO:0045604**	regulation of epiderm	10/935	69/20536	0.001081	0.045513	0.040954	10
BP	**GO:0050673**	epithelial cell prolifer	36/935	458/20536	0.001082	0.045513	0.040954	36
BP	**GO:0071383**	cellular response to s	24/935	265/20536	0.001103	0.045995	0.041387	24
BP	**GO:0042475**	odontogenesis of der	12/935	94/20536	0.001136	0.047003	0.042294	12
BP	**GO:0045616**	regulation of keratino	8/935	47/20536	0.001161	0.047655	0.042881	8
BP	**GO:0044706**	multi-multicellular or	22/935	236/20536	0.001202	0.048973	0.044067	22
